# Alterations of the oral and gut mycobiome and cytokines during long-term follow-up of COVID-19 convalescents

**DOI:** 10.1038/s41392-023-01417-4

**Published:** 2023-04-17

**Authors:** Zhigang Ren, Shanshuo Liu, Qiong Wang, Benchen Rao, Zhaohai Zeng, Yakun Xu, Haiyu Wang, Hong Luo, Jianjun Gou, Zujiang Yu

**Affiliations:** 1 Department of Infectious Diseases, the First Affiliated Hospital of Zhengzhou University, Zhengzhou, 450052 China; 2 Health Management Center, the First Affiliated Hospital of Zhengzhou University, Zhengzhou, 450052 China; 3 Department of Infectious Diseases, Guangshan County People’s Hospital, Guangshan County, Xinyang, 465450 Henan China

**Keywords:** Microbiology, Predictive markers, Infectious diseases, Immunological disorders, Immunology

**Dear Editor**,

A large number of COVID-19 patients experience body-specific conditions such as fatigue, sleep disturbances, anxiety, depression, and dyspnea after the disease has subsided, which is called “long COVID-19”.^1^ Studies have confirmed reduced microbial diversity, enrichment of opportunistic pathogens and depletion of beneficial symbiotic bacteria in patients with COVID-19.^2,3^ Long-term dysregulation of the microbiota has also been observed in recovered patients.^4^ Nevertheless, long-term fungal microbiota follow-up was still blank. The microecology is closely associated with altered immune status and physical health and may be a potential cause of symptoms. We collected tongue coating, feces, and serum samples from 35 COVID-19 confirmed patients who were recovery (CPR0) and followed up for 1 year (CPR1) and 90 age- and sex-matched healthy controls (HC) for internal transcribed spacer (ITS) sequencing (Supplementary Fig. 1, Supplementary Table S1). The blood routine, liver and kidney function, and cytokines of CPR1 and HC were all within the normal range, but a few indicators were different, and most of them in the HC group were lower (Supplementary Tables S2–3).

During COVID-19 recovery, the α diversity of oral (Fig. 1a) and fecal (Fig. 1b) fungal microbiota increased to varying degrees, but did not fully return to normal levels after 1 year. The Venn diagram indicated the shared and unique operational taxonomy units (OTUs) between the three groups (Fig. 1c, d). In the β diversity analysis, non-metric multidimensional scale (NMDS) analysis and principal coordinate analysis (PCoA) revealed that the oral (Fig. 1e, f) and fecal (Fig. 1g, h) fungal microbiota of CPR1 group was between CPR0 and HC, and tended to converge to the latter, and the difference between groups was statistically significant.

**Fig. 1:**
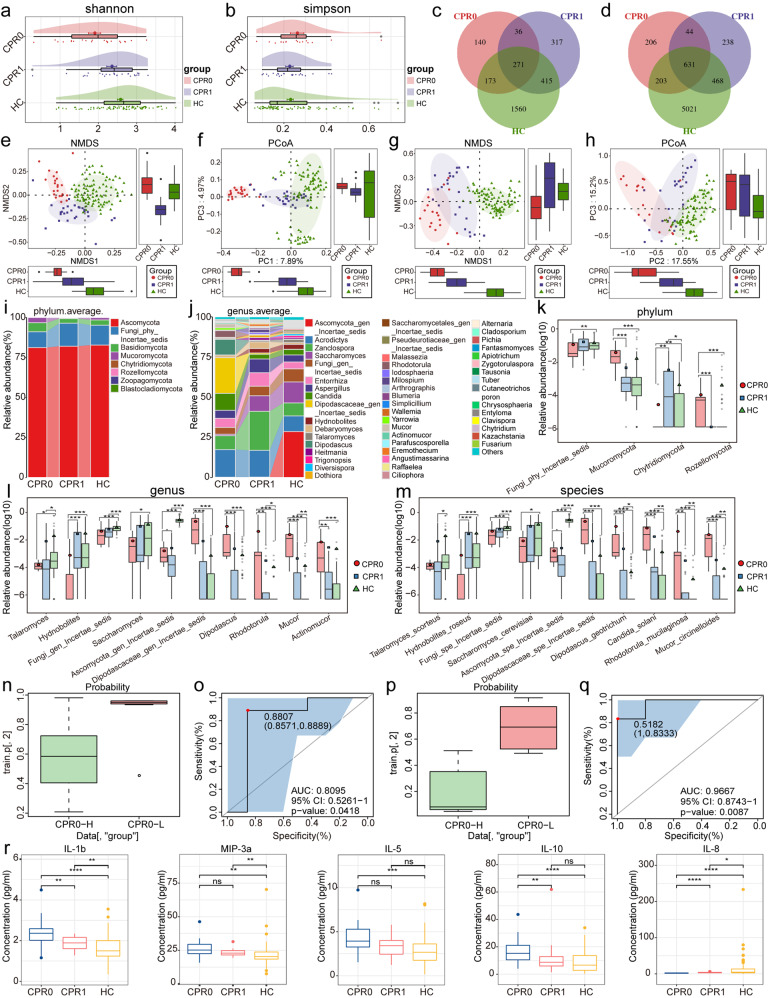
Alterations of the oral and gut mycobiome and cytokines of COVID-19 convalescents after 1-year follow-up. **a** As estimated by the Shannon index, the α diversity of oral mycobiome gradually increased. **b** As estimated by the Simpson index, the α diversity of gut mycobiome gradually increased. **c** With a total of 2912 OTUs found and 271 OTUs shared between the three groups in oral, the HC group had 1560 unique OTUs, significantly higher than the CPR0 and CPR1 groups. **d** 631 OTUs of a total of 6811 OTUs were shared between the three groups, while 5021 OTUs appeared only in the HC group. The NMDS (**e**) and PCoA (**f**) analysis indicated the oral fungal taxonomic composition was conspicuously different in the three groups, and CPR1 gradually approached HC. The NMDS (**g**) and PCoA (**h**) analysis showed the gut fungal taxonomic composition was conspicuously different in the three groups, and CPR1 gradually approached HC. Composition of gut mycobiome at the phylum (**i**) and genus (**j**) levels in different groups. Comparison of fecal mycobiome at the phylum (**k**), genus (**l**) and species (**m**) levels among three groups. (**n**) Compared with the CPR0-H group, the POD value of the CPR0-L group in tongue coating samples increased significantly and achieved great diagnostic results in the training set, with an AUC value of 0.8095 (**o**) (*P* < 0.05). **p** Compared with the CPR0-H group, the POD value of the CPR0-L group in fecal samples increased significantly and achieved great diagnostic results in the training set, with an AUC value of 0.9667 (**q**) (*P* < 0.05). **r** Gradual recovery of the cytokines among three groups. OTUs operational taxonomy units; HC healthy controls, CPR0 confirmed patients recover at discharge, CPR1 confirmed patients recover 1 year, NMDS non-metric multidimensional scale analysis, PCoA principal coordinate analysis, POD probability of disease, AUC area under the curve. Data were represented as mean ± SEM. *P < 0.05, **P < 0.01, ***P < 0.001. ns not significant

When analyzing the average composition and comparison of oral and fecal fungal microbiome at different levels (Supplementary Data S1–10), we found that the dominant phylum of oral and fecal fungi was the same, which were *Ascomycota, Basidiomycota and Mucoromycota* (Fig. 1i). At the genus level, *Zanclospora*, *Aspergillus*, *Acrodictys*, *Entorrhiza* and *Candida* were co-dominant genera, *Malassezia*, *Actinomucor*, *Cladosporium* and *Blumeria* were more abundant in the oral cavity, and *Ascomycota_gen_Incertae_ Sedis* and *Saccharomyces* were more abundant in feces (Fig. 1j). In the between-group differential fungal analysis at the phylum level, the abundance of *Ascomycota* in the oral cavity gradually increased and *Mucoromycota* in feces (Fig. 1k) gradually decreased during disease recovery. At the genus level, there were 21 genera in oral cavity and 11 genera in feces gradually increased, while 8 genera in oral cavity and 11 genera in feces (Fig. 1l) gradually decreased. *Tuber*, *Chrysosphaeria*, and *Angustimassarina* gradually increased in abundance in both environments, while *Actinomucor*, *Rhodotorula*, and *Micor* decreased. At the species level, the abundance of 25 species in the oral cavity and 13 species in the feces gradually increased, and the abundance of 13 species in oral cavity and 13 species in feces (Fig. 1m) gradually decreased. The heatmap can visualize the abundance of different OTUs between three groups of fungal microbes during disease recovery (Supplementary Fig. 2).

Linear discriminant analysis (LDA) effect size (LEfSe) was used to compare the phylogenetic distribution of fungal microorganisms in three groups. We set LDA score (log_10_)>3 to select differential species, and compared with CPR0 and HC, the number of species significantly enriched in tongue samples in CPR1 was the smallest. Nevertheless, the CPR0 group had the largest number of species significantly enriched in fecal samples, more than the CPR1 group and HC group. Interestingly, we found that the CPR0 group had *Dipodascus*, *Candida*, *Rhodotorula*, *Mucor*, *Cladosporium* and *Actinomucor* as the dominant fungi in both tongue and fecal samples (Supplementary Data S11–12). *Candida* causes opportunistic infections when immunocompromised and is the most cited COVID-19-associated fungal infections. *Cladosporium* infections can develop into diseases such as pneumonia if not treated promptly. The MetaCyc database was used to determine possible enrichment pathways for fungal influences on COVID-19. We found that oral and fecal fungi were enriched in tryptophan degradation, guanosine nucleotides degradation, pantothenate and coenzyme A biosynthesis, and sucrose biosynthesis in CPR0 group. Nevertheless, guanosine nucleotides biosynthesis was observed in feces in the CPR1 group. We also found that the amino acid and nucleotide degradation pathways were more enriched in the CPR0 group, while the synthesis pathways were more obvious in the CPR1 group, which was like that in the HC group (Supplementary Data S13–14).

We selected patients with complete clinical information and samples from both CPR0 and CPR1 to establish the prediction model, and finally included 16 patients who provided oral samples and 11 patients who provided fecal samples. After 1-year follow-up, neutralizing antibody inhibition rates below 70% were classified as CPR0-L group, and those above 70% were divided to CPR0-H group. Subsequently, a random forest model was constructed and a five-fold cross-validation was carried out. Finally, 3 oral fungal biomarkers and 18 fecal fungal biomarkers were determined (Supplementary Data S15–16). The probability of disease (POD) index of the CPR0-L group was significantly higher than that of the CPR0-H group in both samples (Fig. 1n, p). The area under the curve (AUC) of the oral prediction model was 0.8095 (95% CI: 0.5261-1, *P* = 0.0418) (Fig. 1o) and the AUC of the fecal prediction model was 0.9667 (95% CI: 0.8743-1, *P* = 0.0087) (Fig. 1q).

Most cytokines, such as IL-1b, MIP-3a, IL-5, and IL-10, tended to decline during disease recovery, while IL-8 concentrations gradually increased (Fig. 1r, Supplementary Data S17). To explore the relationship between fungal flora and immunity during COVID-19 recovery, key oral and fecal fungi and cytokines that showed increasing or decreasing trend among CPR0, CPR1 and HC with statistical difference were selected for Spearman correlation analysis (Supplementary Table S3). In the association analysis between 37 oral OTUs, 23 fecal OTUs and 11 cytokines (Supplementary Fig. 3), IL-8 and MIP-1b were negatively correlated with other cytokines, and were negatively correlated with OTU5, OTU50, OTU67, OTU119, OTU157 in oral fungal microorganisms and OTU5, OTU19, OTU29, OTU69, OTU8088 in fecal fungal microorganisms. In the association analysis of 18 oral fungal OTUs and 8 fecal fungal OTUs with 10 clinical indicators, we found that neutralizing antibody and IgG were negatively correlated with monocytes and total bile acids, and positively correlated with albumin. In addition, OTU5 in oral fungal microorganisms was positively correlated with SARS-CoV-2 binding IgG and IgM during disease recovery. In the association analysis of fungi and bacteria, we found that OTU4, OTU12, OTU19, OTU69, OTU5910 in fecal fungi were negatively correlated with key bacteria (Supplementary Data S18–20). This confirmed that bacteria in the gastrointestinal tract may limit fungal colonization and invasion.

In this study, we reported for the first time that oral and gut fungal microbiome diversity was restored 1 year after discharge from COVID-19 patients, and cytokine concentrations associated with inflammatory storms were gradually reduced. Moreover, the CPR1 neutralizing antibody prediction models based on oral and intestinal fungal flora obtained excellent prediction effects. Spearman correlation analysis also showed significant correlations between oral fungi and bacteria, fecal fungi and bacteria, cytokines, and clinical indicators. However, there are certain shortcomings in this study. Although the predictive model achieves high efficiency, the sample size is small, and large sample validation is required before clinical practice. In addition, the study is observational and requires more in-depth design experiments to validate the findings.

## Supplementary information

SUPPLEMENTAL MATERIAL

## Data Availability

The datasets in this study are available from the corresponding author upon reasonable request. The raw Illumina read data were deposited in the European Bioinformatics Institute European Nucleotide Archive database (PRJNA850097 & PRJNA908275).

## References

[1] Hilpert K, Mikut R (2021). Is there a connection between gut microbiome dysbiosis occurring in COVID-19 patients and post-COVID-19 symptoms?. Front Microbiol..

[2] Ren Z (2021). Alterations in the human oral and gut microbiomes and lipidomics in COVID-19. Gut..

[3] Zuo T (2020). Alterations in fecal fungal microbiome of patients with COVID-19 during time of hospitalization until discharge. Gastroenterology..

[4] Liu Q (2022). Gut microbiota dynamics in a prospective cohort of patients with post-acute COVID-19 syndrome. Gut..

