# Supplementary material for: Alterations of the oral and gut mycobiome and cytokines during long-term follow-up of COVID-19 convalescents
Source: Signal Transduct Target Ther. 2023 Apr 17;8:166. doi: 10.1038/s41392-023-01417-4 (PMC10106887; doi:10.1038/s41392-023-01417-4)
Supplement: Supplementary file 1 — SUPPLEMENTAL MATERIAL [file 41392_2023_1417_MOESM1_ESM.docx]

Supplementary Materials for

**Alterations of the oral and gut mycobiome and cytokines during long-term follow-up of COVID-19 convalescents**

Zhigang Ren ^1†^, Shanshuo Liu ^1†^, Qiong Wang ^2†^, Benchen Rao ^1^, Zhaohai Zeng ^3^, Yakun Xu ^2^, Haiyu Wang ^1^, Hong Luo ^3^, Jianjun Gou ^2*^ and Zujiang Yu ^1*^

^1^ Department of Infectious Diseases, the First Affiliated Hospital of Zhengzhou University, Zhengzhou 450052, China

^2^ Health Management Center, the First Affiliated Hospital of Zhengzhou University, Zhengzhou 450052, China

^3^ Department of Infectious Diseases, Guangshan County People’s Hospital, Guangshan County, Xinyang 465450, Henan, China

^†^ These authors contributed equally to this work.

^*^ Corresponding authors

Correspondence to: Zujiang Yu, Prof., M.D., [johnyuem@zzu.edu.cn](mailto:johnyuem@zzu.edu.cn)

Jianjun Gou, Prof., M.D., goujun64@sohu.com

**This PDF file includes:**

Materials and Methods

Figures S1 to S3

Tables S1 to S3

**Other Supplementary Materials for this manuscript include the following:**

Data S1 to S20

Data S1. Composition and average abundance of oral mycobiome at the phylum level

between three groups

Data S2. Composition and average abundance of oral mycobiome at the genus level between three groups

Data S3. Comparison of oral mycobiome at the phylum level between three groups

Data S4. Comparison of oral mycobiome at the genus level between three groups

Data S5. Comparison of oral mycobiome at the species level between three groups

Data S6. Composition and average abundance of gut mycobiome at the phylum level

between three groups

Data S7. Composition and average abundance of gut mycobiome at the genus level between three groups

Data S8. Comparison of gut mycobiome at the phylum level between three groups

Data S9. Comparison of gut mycobiome at the genus level between three groups

Data S10. Comparison of gut mycobiome at the species level between three groups

Data S11. Statistically significant LDA scores of oral mycobiome at different levels between the three groups

Data S12. Statistically significant LDA scores of gut mycobiome at different levels between the three groups

Data S13. Possible enrichment pathways of oral fungi affecting COVID-19 recovery

identified based on MetaCyc database

Data S14. Possible enrichment pathways of gut fungi affecting COVID-19 recovery identified based on MetaCyc database

Data S15. Optimal biomarkers for prediction models of oral fungi

Data S16. Optimal biomarkers for prediction models of gut fungi

Data S17. Comparison of cytokines between the three groups

Data S18. Spearman correlation analysis of oral fungi, fecal fungi and clinical

Indicators

Data S19. Correlation analysis between oral bacteria and oral fungi

Data S20. Correlation analysis between fecal bacteria and fecal fungi

**Materials and Methods**

Study profile

This study strictly adhered to the Declaration of Helsinki and performed prospective specimen collection and retrospective blinded assessment. It was approved by the Ethics Review Committee of the First Affiliated Hospital of Zhengzhou University (2020-KY-055), and obtained the informed consent signed by all participants. Using reverse transcription polymerase chain reaction (RT-PCR) as the criterion for viral infection and disease recovery, tongue moss samples, stool samples, and serum samples were collected from 35 COVID-19 confirmed patients who were recovery (CPR0) and followed up for 1 year (CPR1). All the patients we selected were in line with the diagnostic criteria and treatment plan for common patients in the fifth and sixth editions of the "Diagnosis and Treatment Plan for Pneumonia Infected with the Novel Coronavirus" issued by the Health Commission of China. Tongue coating samples, stool samples and serum samples from 90 sex- and age-matched healthy controls (HC) were collected at the Health Management Center of the First Affiliated Hospital of Zhengzhou University. None of the participants had received antibiotics or probiotics in the two months prior to enrollment, and had no underlying medical conditions. After rigorous screening, a total of 139 tongue moss, 131 stool and 139 serum samples were included for further analysis. Tongue moss and fecal samples were used for ITS sequencing, and serum samples were detected using cytokine detection kits. Patient data, including demographic, epidemiological characteristics, clinical manifestations, and laboratory results, were derived from medical records and laboratory information systems.

Sample Collection and DNA Extraction

Participants provided a fresh tail stool sample, divided into 3 pieces of 200 mg, from 6 to 8 a.m., and immediately stored in a −80 °C freezer. Participants rinsed their mouths twice with normal saline, scraped the posterior to anterior middle zone of the tongue moss using a throat swab, and immediately placed the swab in a collection tube for storage in a -80 °C freezer. Discard all samples left at room temperature over 2 hours. Serum samples of subjects were collected and divided into three aliquots (200 μL per tube) for routine laboratory testing and cytokine quantification, respectively. DNA extraction was carried out as described in our previous study ^1^.

PCR Amplification

The universal primers ITS3F (5′-GCATCGATGAAGAACGCAGC-3′) and ITS4R

(5′- TCCTCCGCTTATTGATATGC-3′) were used for amplification of the ITS2 region of the fungal ITS gene. Samples were processed using a CPR machine (ABI GeneAmp 9700) in which 1 cycle consists of four reactions：95°C for 3 minutes, 94°C for 30 seconds, 55°C for 30 seconds, and 72°C for 30 seconds. Repeating 35 cycles and at 72 °C for 5 minutes. PCR products were then isolated, extracted, and purified using Agarose gels (Amuhara Biosciences, United City, CA), and quantified by a fluorescence assay kit (Quant-iT PicoGreen, Invitrogen).

Library Construction and Sequencing

The amplified products were purified by the magnetic bead method, and the DNA library was constructed according to the official operating instructions. Then we mixed the different samples at equimolar ratio for single-ended sequencing analysis on the Illumina MiSeq platform (Shanghai Mobio Biomedical Technology, China). The raw Illumina read data were deposited in the European Bioinformatics Institute European Nucleotide Archive database (PRJNA850097 &PRJNA908275).

Operational Taxonomy Unit (OTU) Clustering and Taxonomy Annotation

All samples with equal numbers of random reads were extracted from the raw data using USEARCH (version 11.0.667), and reads with an expected error greater than 1 per base were excluded. Raw sequencing reads were quality filtered and flash merged. OTUs were clustered and identified and removed chimeric sequences using UPARSE version 7.1 (http://drive5.com/uparse) with an identity threshold of 98.5%. Then annotated them with unite v8.3 (https://unite.ut.ee/repository.php). Unclassified Fungi were further performed BLAST against ITS_RefSeq_Fungi version1.1.

Bioinformatics

Alpha diversity indicators (Shannon Index and Simpson Index) were evaluated using Mothur v1.42.1. The Bray-Curtis, weighted and unweighted UniFrac differences were calculated by QIIME. The principal coordinate analysis (PCoA) and non-metric multidimensional scale (NMDS) were used for visualization, and ANOSIM was used to test statistical significance between groups. Linear discriminant analysis (LDA) effect size (LEfSe) was used to detect taxa with differential abundance between groups. And we used PICRUSt2 v2.4.1 (https://github.com/picrust/picrust2/wiki) to predict functional abundance based on ITS rRNA gene sequence.

Identification of the OTU Biomarkers and Construction of Probability of Disease (POD)

We used the Kruskal-Wallis test to determine the significance (*P*<0.05) and selected OTU biomarkers in the oral and fecal fungal microbiota for further analysis. Construct a random forest model and perform a fivefold cross-validation. we defined the POD index as the ratio between the number of randomly generated decision trees that predicted sample as “CPR0-L” and that of “CPR0-H”. The ability to discriminate biomarkers was assessed by plotting the receiver operator characteristic (ROC) curve and calculating the area under the curve (AUC).

Antibody detection

We used SARS-CoV-2 Surrogate Virus Neutralization Test Kit (Nanjing GenScript Biological Co., LTD) ^2^ to detect COVID-19 neutralizing antibodies. The positive control/negative control/diluted samples were mixed with diluted HRP-RBD at 1:1 volume ratio and incubated at 37℃ for 30 min. Then 100 μL of the three mixtures were added to the corresponding wells and incubated at 37℃ for 15 min. Wash the plate with 260 µL of 1× Wash Solution per well for four times. 100μL TMB solution was added to each well and incubated in the dark at 20-25 °C for 15 min. Then add 50 μL Stop Solution to each well to stop the reaction and read immediately. Values ≥30% are certified positive and < 30% are certified negative.

We detected serum anti-SARS-CoV-2 IgG and IgM by direct chemiluminescence microparticle technology. The kit and Flash 3000-C chemiluminescence immunoassay analyzer needed for the test were all sourced from YHLO Biotech Co., Ltd., Shenzhen, China ^3^. The positive verdict value of the kit was 10 U/ml (a value > 10 U/ml was defined as positive, and a value < 10 U/ml was defined as negative). The IgG and IgM level were calculated as log10(value).

Quantitative detection of cytokines

We used the Human High Sensitivity T Cell Magnetic Bead Panel for cytokine quantification in serum samples. After adding 200 µL Wash Buffer into each well of plate, we sealed and mixed it for 10 minutes at room temperature (RT, 20-25°C). Then we added 50 µL control to appropriate wells, 50 µL appropriate matrix to background wells, 25 µL Assay Buffer and 25 µL neat samples to sample wells, 25 µL beads to each well. Seal and wrap the plate and incubate it on a plate shaker overnight (16-18 h) at 4°C. Remove well contents and wash plate 3 times. After that, we added 50 µL of detection antibodies into each well, sealed, covered with foil and incubated with agitation for 1 hour at RT. Add 50 µL Streptavidin-Phycoerythrin to each well containing the 50 µL of detection antibodies. Incubate again with agitation for 30 min, remove the contents and wash 3 times. Rresuspend the beads on a plate shaker for 5 minutes after adding 150 µL Sheath Fluid (or Drive Fluid if using MAGPIX®) to all wells. Run plate on Luminex® 200™, HTS, FLEXMAP 3D® or MAGPIX® with xPONENT® software. Save and analyze the Median Fluorescent Intensity (MFI) data using a 5-parameter logistic or spline curve-fitting method for calculating analyte concentrations in samples and Controls.

Statistical analysis

Normally distributed continuous variables were expressed using means ± standard deviations and statistically compared using the independent t-test. Nonnormally distributed continuous variables were expressed using interquartile range (IQR) and compared using the Mann-Whitney U test. Nonparametric Kruskal-Wallis test was used to compare nonnormally distributed continuous variables in multiple groups. Chi-square test or Fisher's exact test was used for categorical variables. Spearman's rank test was used for correlation analysis. Statistical analysis using SPSS version 26.0 (SPSS Inc., Chicago, IL). A p-value of less than 0.05 was considered a significant difference.

Reference

1 Cui, G. Y. *et al.* Characterization of oral and gut microbiome and plasma metabolomics in COVID-19 patients after 1-year follow-up. *Military Medical Research* **9**, 32 (2022).

2 Tan, C. W. *et al.* A SARS-CoV-2 surrogate virus neutralization test based on antibody-mediated blockage of ACE2-spike protein-protein interaction. *Nature biotechnology* **38**, 1073-1078 (2020).

3 Qian, C. *et al.* Development and multicenter performance evaluation of fully automated SARS-CoV-2 IgM and IgG immunoassays. *Clinical chemistry and laboratory medicine* **58**, 1601-1607 (2020).


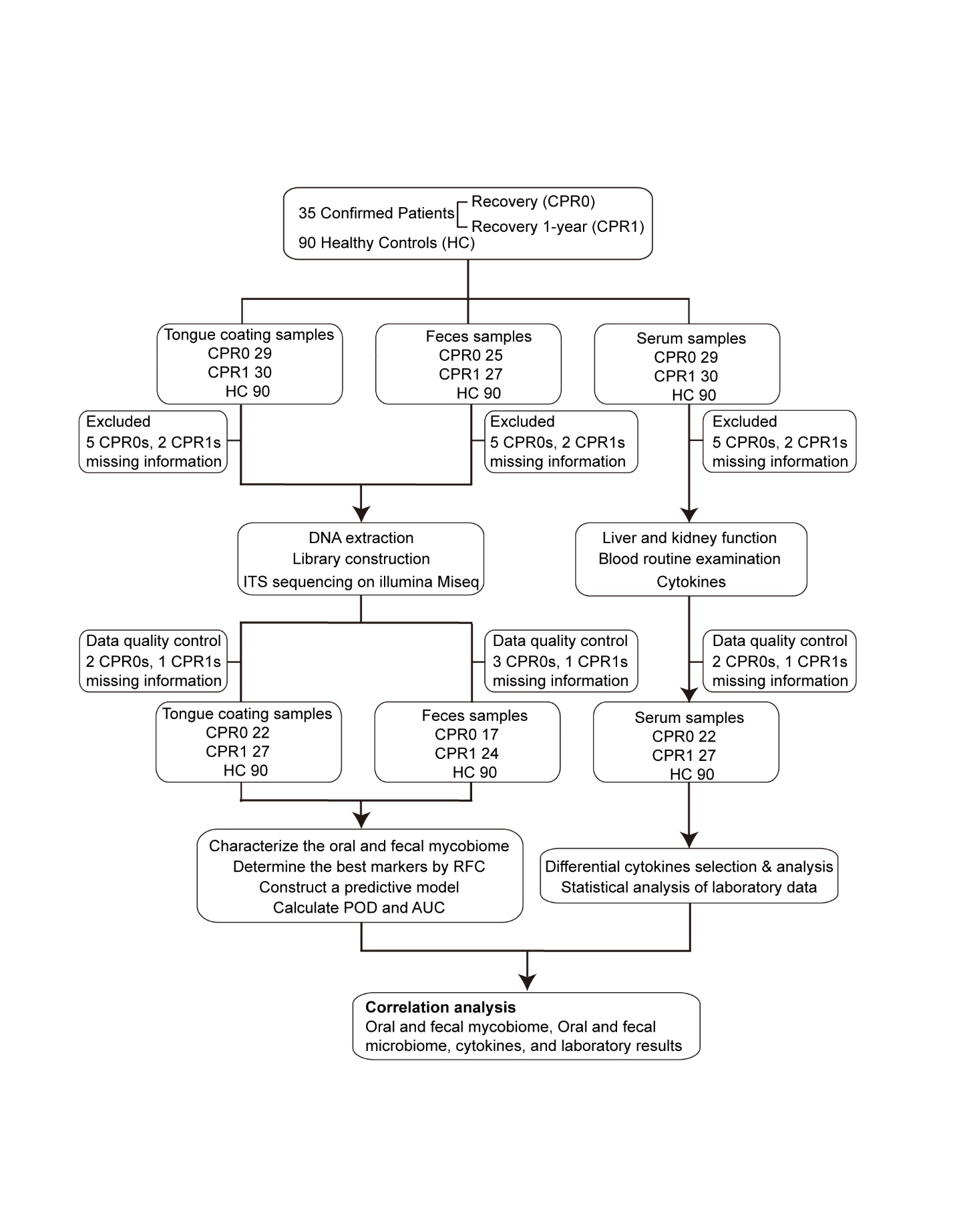


Figure. S1. Study design and flow diagram. 35 confirmed patients were enrolled, and 1-year follow-up was completed after recovery. In addition, we also recruited 90 matched healthy controls (HCs). A total of 440 samples were prospectively collected, including 149 tongue-coating samples, 142 stool samples and 149 plasma samples. After rigorous inclusion and exclusion criteria, 419 samples were included for further analysis. ITS sequencing was conducted on tongue-coating samples and stool samples, and cytokines quantification was carried out on plasma samples. CPR0 confirmed patients recover at discharge, CPR1 confirmed patients recover 1 year, HCs healthy controls, ITS internal transcribed spacer, RFC random forest model, POD probability of disease, AUC area under the curve.

**
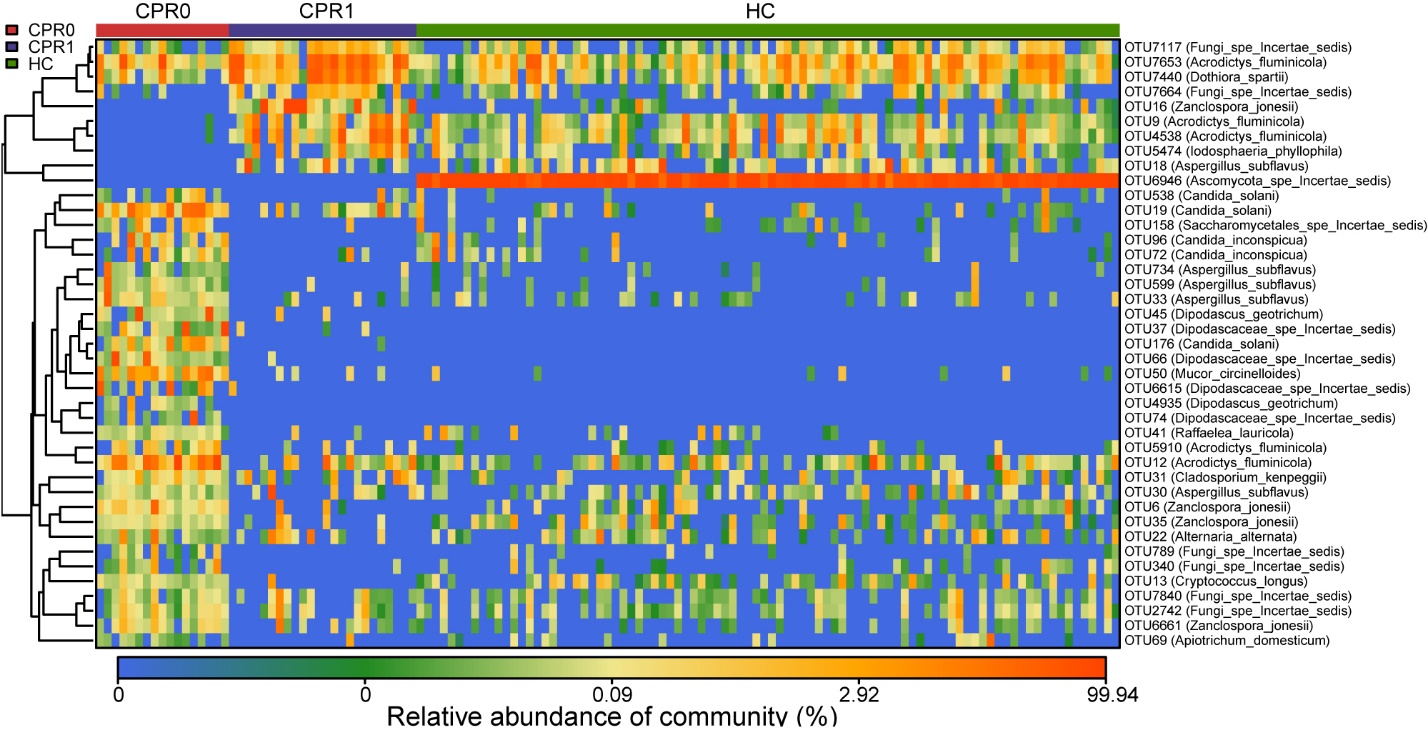
**

**Figure. S2.** **Heatmap displayed the gradual recovery of the differential OTUs in the gut mycobiome.** CPR0 confirmed patients recover at discharge, CPR1 confirmed patients recover 1 year, HC healthy control, OTUs operational taxonomy units.

**
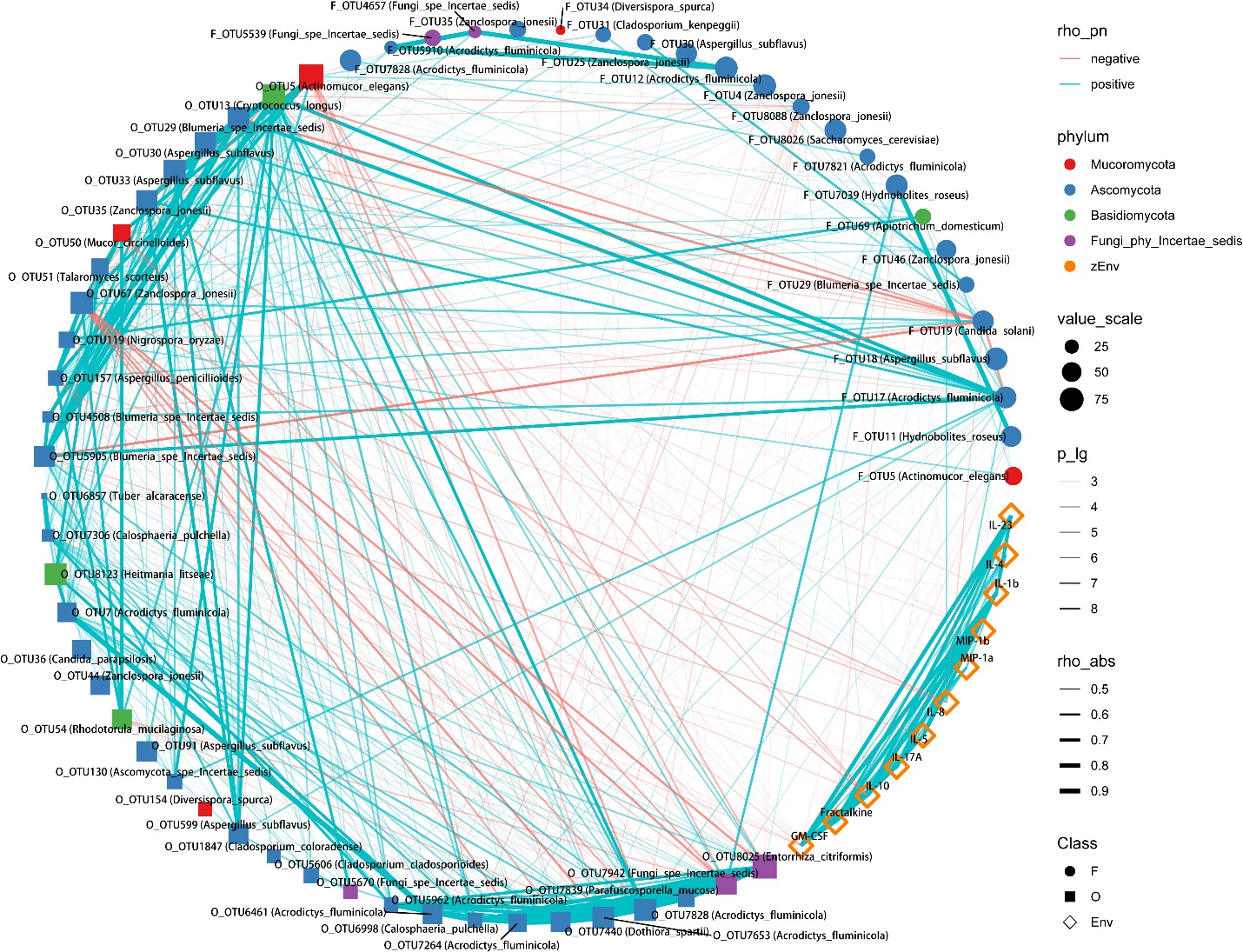
**

**Figure. S3.** **Correlation of oral mycobiome, fecal mycobiome, and cytokines among three groups during disease recovery.** We included mycobiomes and cytokines that showed an increasing or decreasing trend during the recovery process for Spearman’s correlation analysis. The results indicated correlations among 37 oral OTUs, 23 fecal OTUs and 11 cytokines. Red lines indicate negative correlations, blue lines indicate positive correlations, and the width of the lines represents the strength of the correlation (Spearman). The transparency of the lines represented the negative logarithm of the P-value of correlation, translucent lines meant (-log_10_ *P*)>5 and opaque lines meant (-log_10_ *P*)>10. The size of the points indicates the relative abundance of genera and cytokines. The colors of points display the different phyla of the mycobiome. The circle represents the fecal mycobiome, the square represents the oral mycobiome, and the diamond represents cytokines. CPR0 confirmed patients recover at discharge, CPR1 confirmed patients recover 1 year, HC healthy control, OTUs operational taxonomy units.

Table S1. Disease severity and treatment in patients who have recovered from COVID-19.

| Number | Demographic Information | | | | | Clinical Symptoms | | | | | | | |
| --- | --- | --- | --- | --- | --- | --- | --- | --- | --- | --- | --- | --- | --- |
|  | Year | Gender  (Male:1/Fem ale:2) | Height(cm) | Weight (kg) | BMI (kg/m2) | Maximum  temperature  (℃) | Cough  (Yes:1/No:0) | Myalgia (Yes:1/No:0) | Fatigue (Yes:1/No:0) | Expectoration (Yes:1/No:0) | Headache (Yes:1/No:0) | Diarrhea (Yes:1/No:0) |  |
| 7801 | 54 | 2 | NA | NA | NA | 38.2 | 1 | 0 | 0 | 1 | 0 | 0 |  |
| 7802 | 51 | 1 | 172 | 85 | 28.73 | 38.5 | 0 | 0 | 0 | 0 | 0 | 0 |  |
| 7803 | 40 | 1 | 168 | 89 | 26.48 | 39.2 | 1 | 0 | 1 | 1 | 0 | 0 |  |
| 7804 | 58 | 2 | 165 | 55 | 20.20 | 36.8 | 0 | 0 | 0 | 0 | 0 | 0 |  |
| 7805 | 46 | 1 | 176 | 70 | 22.60 | 39.3 | 0 | 0 | 0 | 0 | 0 | 0 |  |
| 7806 | 55 | 2 | 169 | 65 | 22.76 | 39.5 | 0 | 0 | 0 | 0 | 0 | 0 |  |
| 7807 | 45 | 2 | 160 | 73 | 28.52 | 38.2 | 0 | 0 | 0 | 0 | 0 | 0 |  |
| 7808 | 46 | 1 | 168 | 76 | 26.93 | 36.6 | 1 | 0 | 0 | 0 | 0 | 0 |  |
| 7809 | 47 | 1 | 176 | 72 | 23.24 | 37.9 | 0 | 0 | 0 | 0 | 0 | 0 |  |
| 7810 | 41 | 2 | 158 | 58 | 23.23 | 37.5 | 0 | 0 | 0 | 0 | 0 | 0 |  |
| 7811 | 47 | 1 | NA | NA | NA | 37.3 | 1 | 0 | 1 | 1 | 0 | 0 |  |
| 7812 | 40 | 1 | 170 | 61 | 21.11 | 38.8 | 0 | 0 | 0 | 0 | 0 | 0 |  |
| 7813 | 49 | 2 | 159 | 56 | 22.15 | 38.1 | 0 | 0 | 0 | 0 | 0 | 0 |  |
| 7814 | 50 | 2 | 168 | 64 | 22.68 | 37.5 | 0 | 0 | 0 | 0 | 0 | 0 |  |
| 7815 | 56 | 2 | 156 | 62 | 25.48 | 36.8 | 1 | 1 | 1 | 0 | 1 | 0 |  |
| 7816 | 39 | 1 | 165 | 75 | 27.55 | 38.8 | 0 | 0 | 0 | 0 | 0 | 0 |  |
| 7818 | 50 | 1 | 173 | 80 | 26.73 | 37.7 | 0 | 0 | 0 | 0 | 0 | 0 |  |
| 7819 | 44 | 2 | 162 | 62 | 23.62 | 37.6 | 0 | 0 | 0 | 0 | 0 | 0 |  |
| 7820 | 51 | 2 | 160 | 60 | 23.44 | 38.1 | 1 | 0 | 1 | 0 | 1 | 0 |  |
| 7821 | 22 | 1 | 180 | 100 | 30.86 | 37.7 | 0 | 0 | 0 | 0 | 0 | 0 |  |
| 7822 | 56 | 2 | 160 | 60 | 23.44 | 38.1 | 0 | 0 | 0 | 0 | 0 | 0 |  |
| KF001 | 58 | 1 | NA | NA | NA | 38.2 | 1 | 0 | 0 | 1 | 0 | 0 |  |
| KF014 | 69 | 2 | NA | NA | NA | 38.3 | 0 | 0 | 1 | 0 | 0 | 0 |  |
| KF016 | 46 | 1 | NA | NA | NA | 39 | 1 | 1 | 1 | 1 | 1 | 0 |  |
| KF017 | 30 | 1 | NA | NA | NA | 36.4 | 1 | 0 | 0 | 1 | 0 | 0 |  |
| KF019 | 53 | 1 | NA | NA | NA | 37.2 | 1 | 0 | 0 | 1 | 0 | 0 |  |
| KF026 | 38 | 1 | 155 | 50 | 20.81 | 38.7 | 0 | 0 | 0 | 0 | 0 | 0 |  |
| KF031 | 41 | 2 | 167 | 70 | 25.10 | 38.1 | 0 | 0 | 0 | 0 | 0 | 0 |  |

| Number | vital Signs | | | | Imaging Features | Respiratory  Failure  (Yes:1/No:0) | Shock (Yes:1/No:0) | Antiviral Treatment (Yes:1/No:0) | | |
| --- | --- | --- | --- | --- | --- | --- | --- | --- | --- | --- |
|  | Systolic  Pressure  (mmHg) | Diastolic  Pressure  (mmHg) | Heart Rate (/min) | Respiratory  Rate  (/min) | Pneumonia |  |  | Ribavirin | Lopinavir  /Ritonavir Oral Solution | Arbidol |
| 7801 | 136 | 97 | 87 | 19 | Bilateral | 0 | 0 | 1 | 1 | 1 |
| 7802 | 119 | 78 | 96 | 19 | Bilateral | 0 | 0 | 0 | 1 | 1 |
| 7803 | 120 | 87 | 93 | 24 | Bilateral | 0 | 0 | 1 | 1 | 0 |
| 7804 | 120 | 96 | 100 | 20 | Bilateral | 0 | 0 | 0 | 0 | 1 |
| 7805 | 109 | 86 | 76 | 21 | Bilateral | 0 | 0 | 1 | 1 | 0 |
| 7806 | 109 | 87 | 75 | 15 | Bilateral | 0 | 0 | 0 | 1 | 1 |
| 7807 | 120 | 86 | 81 | 21 | Bilateral | 0 | 0 | 1 | 1 | 0 |
| 7808 | 162 | 90 | 79 | 21 | Right Side | 0 | 0 | 1 | 1 | 1 |
| 7809 | 109 | 76 | 78 | 15 | Bilateral | 0 | 0 | 0 | 1 | 1 |
| 7810 | 97 | 69 | 77 | 18 | Bilateral | 0 | 0 | 1 | 0 | 0 |
| 7811 | 160 | 110 | 120 | 24 | Bilateral | 0 | 0 | 1 | 1 | 0 |
| 7812 | 98 | 65 | 69 | 16 | Bilateral | 0 | 0 | 1 | 1 | 0 |
| 7813 | 98 | 67 | 78 | 14 | Bilateral | 0 | 0 | 0 | 1 | 1 |
| 7814 | 130 | 84 | 94 | 24 | Left Side | 0 | 0 | 1 | 1 | 0 |
| 7815 | 124 | 74 | 82 | 20 | Bilateral | 0 | 0 | 1 | 1 | 0 |
| 7816 | 125 | 78 | 86 | 18 | Bilateral | 0 | 0 | 1 | 1 | 1 |
| 7818 | 109 | 87 | 79 | 19 | Bilateral | 0 | 0 | 1 | 1 | 0 |
| 7819 | 100 | 78 | 75 | 17 | Bilateral | 0 | 0 | 0 | 1 | 1 |
| 7820 | 124 | 82 | 100 | 20 | Bilateral | 0 | 0 | 1 | 1 | 1 |
| 7821 | 108 | 78 | 80 | 18 | Bilateral | 0 | 0 | 0 | 1 | 1 |
| 7822 | 128 | 87 | 75 | 19 | Bilateral | 0 | 0 | 0 | 0 | 1 |
| KF001 | 136 | 79 | 72 | 21 | Bilateral | 0 | 0 | 0 | 0 | 1 |
| KF014 | 125 | 80 | 70 | 20 | Bilateral | 0 | 0 | 1 | 1 | 0 |
| KF016 | 118 | 70 | 92 | 20 | Bilateral | 0 | 0 | 0 | 1 | 1 |
| KF017 | 147 | 90 | 88 | 20 | Left Side | 0 | 0 | 1 | 1 | 0 |
| KF019 | 142 | 100 | 122 | 24 | Right Side | 0 | 0 | 0 | 1 | 1 |
| KF026 | 118 | 89 | 87 | 15 | Left Side | 0 | 0 | 0 | 0 | 0 |
| KF031 | 129 | 79 | 87 | 19 | Bilateral | 0 | 0 | 0 | 1 | 1 |

Table S2. Type or paste caption here. Create a page break and paste in the Table above the caption.

|  | CPR0(n=22) | CPR1(n=27) | HC(n=90) | *P* Value |
| --- | --- | --- | --- | --- |
| Age (years) | 47.09±6.06 | 48.44±16.31 | 48.06±6.72 | 0.873 |
| Gender (Male/Female) | 12/10 | 17/10 | 43/43 | 0.496 |
| Neutralizing antibody inhibition rate (%) | 92.57(8.69) | 64.19(40.47) | 0.22(8.00) | 0.001 |
| lgM (mg/ml) | 59.43(143.31) | 1.18(4.29) | 0.28(0.19) | 0.001 |
| lgG (mg/ml) | 168.56(49.44) | 33.01(27.73) | 0.18(0.15) | 0.001 |
| WBC (10^9/L) | - | 6.03±1.58 | 6.20±1.75 | 0.649 |
| RBC (10^12/L) | - | 4.69±0.51 | 4.63±0.50 | 0.618 |
| HGB (g/L) | - | 143.19±15.54 | 140.42±18.07 | 0.475 |
| PLT (10^9/L) | - | 213.07±57.95 | 221.66±72.47 | 0.576 |
| NEU# (10^9/L) | - | 3.52±1.23 | 3.73±1.55 | 0.520 |
| LYM# (10^9/L) | - | 2.06±0.50 | 1.92±0.56 | 0.235 |
| MON# (10^9/L) | - | 0.25±0.06 | 0.39±0.17 | 0.001 |
| EOS# (10^9/L) | - | 0.17±0.19 | 0.12±0.09 | 0.061 |
| BAS# (10^9/L) | - | 0.03±0.02 | 0.03±0.02 | 0.176 |
| NEU% | - | 57.69±6.83 | 58.88±9.24 | 0.547 |
| LYM% | - | 34.79±6.46 | 31.82±7.45 | 0.065 |
| MON% | - | 4.16±0.87 | 6.63±3.50 | 0.001 |
| EOS% | - | 2.78±2.95 | 1.95±1.37 | 0.048 |
| BAS% | - | 0.44±0.24 | 0.52±0.33 | 0.234 |
| HCT (%) | - | 42.82±4.09 | 41.36±6.35 | 0.264 |
| MCV (fL) | - | 91.42±3.44 | 90.35±4.78 | 0.286 |
| MCH (pg) | - | 30.53±1.36 | 30.27±2.14 | 0.543 |
| MCHC (g/L) | - | 334.11±9.84 | 334.74±12.91 | 0.815 |
| RDW-CV (%) | - | 12.44±0.75 | 12.51±1.06 | 0.781 |
| MPV (fL) | - | 11.21±1.06 | 10.93±1.12 | 0.237 |
| PDW (%) | - | 13.79±2.71 | 13.20±2.69 | 0.327 |
| ALT (U/L) | - | 28.15±19.54 | 18.82±10.12 | 0.024 |
| AST (U/L) | - | 21.63±4.71 | 19.82±5.41 | 0.120 |
| AST/ALT | - | 0.97±0.40 | 1.27±0.96 | 0.109 |
| GGT (U/L) | - | 29.26±24.69 | 27.70±30.45 | 0.809 |
| ALP (U/L) | - | 92.22±69.42 | 68.48±17.89 | 0.167 |
| TBIL (μmol/L) | - | 12.76±4.94 | 7.14±4.72 | 0.001 |
| DBIL (μmol/L) | - | 2.08±0.91 | 3.02±1.91 | 0.001 |
| IBIL (μmol/L) | - | 10.68±4.36 | 4.12±2.99 | 0.001 |
| TP (g/L) | - | 73.41±3.29 | 67.12±7.06 | 0.001 |
| ALB (g/L) | - | 48.21±2.24 | 43.15±3.91 | 0.001 |
| GLOB (g/L) | - | 25.20±2.75 | 23.98±3.86 | 0.075 |
| A/G | - | 1.94±0.24 | 1.83±0.23 | 0.036 |
| UREA (mmol/L) | - | 6.23±1.39 | 5.41±1.20 | 0.004 |
| UA (μmol/L) | - | 341.59±100.34 | 289.77±82.50 | 0.008 |
| CREA (μmol/L) | - | 72.76±22.37 | 69.65±20.67 | 0.505 |

**Abbreviation:** WBC: White blood cells, RBC: Red blood cells, HGB: Hemoglobin, PLT: Platelet, NEU: Neutrophils, LYM: Lymphocytes, MON: Monocytes, EOS: Eosnophils, BAS: Basophils, HCT: Hematocrit, MCV: Mean Corpuscular Volume, MCH: Mean Corpuscular Hemoglobin, MCHC: Mean Corpusular Hemoglobin Concerntration, RDW-CV: Red blood cell volume distribution width-coefficient of variation, MPV: Mean Platelet Volume, PDW: Platelet Distribution Width, ALT: Alanine aminotransferase, AST: Aspartate aminotransferase, GGT: Glutamyl transpeptidase, ALP: Alkaline phosphatase, TBIL: Total bilirubin, DBIL: Direct Bilirubin, IBIL: indirect bilirubin, TP: Total protein, ALB: Albumin, GLOB: Globulin, UA: Uric Acid, CREA: Creatinine.

Table S3. The cytokine levels of subjects in three groups.

|  | CPR0(n=22) | CPR1(n=27) | HC(n=90) | P Value |
| --- | --- | --- | --- | --- |
| ITAC | 20.13（9.02） | 23.16（18.18） | 25.27（15.37） | 0.308 |
| GM-CSF | 26.12（8.81） | 13.72（6.11） | 14.96（18.81） | 0.003 |
| Fractalkine | 267.36（53.99） | 224.18（60.41） | 230.73（77.01） | 0.068 |
| IFNy | 20.90（3.88） | 18.41（4.11） | 18.61（9.49） | 0.400 |
| IL-10 | 15.77（10.53） | 8.83（7.92） | 7.23（10.72） | 0.001 |
| MIP-3a | 25.81（6.95） | 22.99（3.49） | 21.25（5.24） | 0.001 |
| IL-12(p70) | 3.20（1.52） | 3.64（1.12） | 3.22（2.31） | 0.556 |
| IL-13 | 3.75（3.13） | 4.71（4.51） | 5.23（3.64） | 0.117 |
| IL-17A | 9.09（3.71） | 5.88（3.76） | 6.61（5.80） | 0.025 |
| IL-1b | 2.45（0.55） | 1.95（0.55） | 1.54（0.76） | 0.001 |
| IL-2 | 4.49（1.66） | 4.49（1.58） | 3.66（1.73） | 0.012 |
| IL-21 | 4.36（2.11） | 2.93（1.82） | 3.11（2.03） | 0.055 |
| IL-4 | 34.55（10.59） | 34.69（9.27） | 31.66（18.72） | 0.424 |
| IL-23 | 214.64（87.40） | 153.02（149.40） | 168.83（221.24） | 0.215 |
| IL-5 | 4.10（2.09） | 3.43（1.69） | 2.75（1.88） | 0.001 |
| IL-6 | 1.60（0.89） | 2.11（1.18） | 1.62（1.63） | 0.376 |
| IL-7 | 18.22（3.52） | 20.75（3.16） | 17.78（4.64） | 0.001 |
| IL-8 | 2.15（1.18） | 3.33(1.49) | 4.64(10.65) | 0.001 |
| MIP-1a | 20.20(4.10) | 17.91(3.18) | 19.14(6.18) | 0.147 |
| MIP-1b | 9.58(4.84) | 19.22(15.04) | 20.03(16.48) | 0.001 |
| TNFa | 6.12(2.17) | 6.15(2.59) | 4.96(1.60) | 0.023 |

| Data S1. Composition and average abundance of oral mycobiome  at the phylum level between three groups | | | |
| --- | --- | --- | --- |
| ID | CPR0 | CPR1 | HC |
| Ascomycota | 0.449825091 | 0.517199704 | 0.717621389 |
| Basidiomycota | 0.187157091 | 0.285022667 | 0.137061378 |
| Fungi_phy_Incertae_sedis | 0.011720409 | 0.193842259 | 0.113571889 |
| Mucoromycota | 0.350779636 | 0.00393537 | 0.030303722 |
| Rozellomycota | 3.38E-05 | 0 | 0.001001889 |
| Zoopagomycota | 2.48E-04 | 0 | 3.73E-04 |
| Chytridiomycota | 2.36E-04 | 0 | 6.71E-05 |

| Data S2. Composition and average abundance of oral mycobiome at the genus level  between three groups | | | |
| --- | --- | --- | --- |
| ID | CPR0 | CPR1 | HC |
| Zanclospora | 0.184707909 | 0.08105363 | 0.210945633 |
| Aspergillus | 0.023146182 | 0.103940815 | 0.104383667 |
| Acrodictys | 0.010992818 | 0.064523074 | 0.101940244 |
| Fungi_gen_Incertae_sedis | 0.010212909 | 0.145799037 | 0.055815311 |
| Malassezia | 0.051561909 | 0.158709963 | 0.033874256 |
| Actinomucor | 0.335696682 | 9.82E-04 | 2.65E-05 |
| Entorrhiza | 0.0015075 | 0.048043222 | 0.057756578 |
| Cladosporium | 0.016880318 | 0.053332593 | 0.035600889 |
| Blumeria | 2.05E-05 | 8.24E-04 | 0.045723011 |
| Candida | 0.062088818 | 0.059688037 | 0.009582456 |
| Cryptococcus | 0 | 2.96E-06 | 0.036513811 |
| Raffaelea | 0.007326955 | 2.35E-04 | 0.030810478 |
| Diversispora | 0.001314227 | 4.16E-04 | 0.030039222 |
| Arthrographis | 2.90E-05 | 5.89E-06 | 0.022962267 |
| Heitmania | 1.92E-05 | 0.009904259 | 0.017578344 |
| Udeniomyces | 0.064740091 | 0.002935556 | 0.003243222 |
| Ascomycota_gen_Incertae_sedis | 0.014233091 | 0.005749741 | 0.014598311 |
| Milospium | 5.67E-04 | 0.011125333 | 0.015415089 |
| Talaromyces | 0.010010227 | 0.004646815 | 0.014155689 |
| Alternaria | 0.013504136 | 0.003827296 | 0.011155422 |
| Wallemia | 2.76E-04 | 0.015740111 | 0.007305711 |
| Pleurotus | 6.44E-04 | 0.01629137 | 0.005658111 |
| Dipodascaceae_gen_Incertae_sedis | 0.015057455 | 0.019001556 | 5.36E-04 |
| Saprochaete | 0 | 0 | 0.008890656 |
| Rhodotorula | 0.031055182 | 0.001761444 | 3.71E-04 |
| Debaryomyces | 0.030914773 | 1.78E-06 | 6.51E-04 |
| Filobasidium | 0.003367045 | 0.022134296 | 5.96E-04 |
| Parafuscosporella | 8.16E-04 | 0.002120815 | 0.007147167 |
| Dothiora | 1.08E-04 | 0.005466556 | 0.005962189 |
| Schizophyllum | 9.43E-04 | 0.019770037 | 1.58E-04 |
| Simplicillium | 0.001620364 | 0.00432737 | 0.004459333 |
| Fusarium | 0.003169727 | 0.005327667 | 0.003044433 |
| Phaeosphaeria | 8.34E-05 | 7.95E-04 | 0.004924044 |
| Aureobasidium | 0.004582136 | 0.010784593 | 5.57E-04 |
| Diaporthe | 9.96E-05 | 0 | 0.004047978 |
| Tuber | 1.31E-04 | 2.14E-04 | 0.003741267 |
| Malasseziaceae_gen_Incertae_sedis | 0.001198682 | 0.004315074 | 0.002103222 |
| Leptospora | 2.36E-04 | 0.008254407 | 0.001092922 |
| Mucor | 0.013755227 | 3.92E-04 | 8.85E-05 |
| Botrytis | 1.40E-04 | 0.007715667 | 0.001182278 |
| Botryosphaeria | 0.011873045 | 6.76E-04 | 3.16E-04 |
| Ascochyta | 8.94E-04 | 0.009693926 | 6.85E-05 |
| Sporobolomyces | 0.006518136 | 1.19E-06 | 0.001138 |
| Calosphaeria | 1.85E-05 | 5.93E-04 | 0.0023836 |
| Bandonia | 4.56E-05 | 0.007030222 | 3.84E-04 |
| Penicillium | 0.001290045 | 0.002380741 | 0.001366133 |
| Symmetrospora | 0 | 0.004859889 | 7.94E-04 |
| Chrysosphaeria | 0 | 1.80E-04 | 0.002012467 |
| Dioszegia | 0.007924636 | 1.04E-05 | 1.05E-04 |
| Togniniella | 0 | 0 | 0.0020321 |
| Others | 0.054679773 | 0.074417037 | 0.0747632 |

| Data S3. Comparison of oral mycobiome at the phylum level between three groups | | | | | |
| --- | --- | --- | --- | --- | --- |
| ID | Ascomycota | Basidiomycota | Fungi_phy_Incertae_sedis | Mucoromycota | Zoopagomycota |
| CPR0.median | 0.4316(0.1587,0.6931) | 0.0727(0.0192,0.3126) | 0.0035(0.0019,0.012) | 0.2751(0.0726,0.5734) | <0.0001(<0.0001,0.0002) |
| CPR0.mean | 0.449825091 | 0.187157091 | 0.011720409 | 0.350779636 | 2.48E-04 |
| CPR0.se | 0.064912274 | 0.04625814 | 0.004616464 | 0.065970207 | 1.17E-04 |
| CPR1.median | 0.5307(0.3261,0.741) | 0.2326(0.1356,0.402) | 0.1782(0.0784,0.2548) | 0.0002(<0.0001,0.0011) | <0.0001(<0.0001,<0.0001) |
| CPR1.mean | 0.517199704 | 0.285022667 | 0.193842259 | 0.00393537 | 0 |
| CPR1.se | 0.04687668 | 0.042686643 | 0.030474451 | 0.002270929 | 0 |
| HC.median | 0.7468(0.5822,0.8954) | 0.0872(0.029,0.1982) | 0.0542(0.0229,0.1532) | 0.0014(0.0006,0.0072) | <0.0001(<0.0001,<0.0001) |
| HC.mean | 0.717621389 | 0.137061378 | 0.113571889 | 0.030303722 | 3.73E-04 |
| HC.se | 0.02017167 | 0.014425092 | 0.014386823 | 0.012611368 | 1.60E-04 |
| p-value | 8.90E-06 | 0.00708398 | 1.08E-09 | 1.43E-11 | 0.003739765 |
| z-score | -4.442285034 | -2.692870383 | -6.097826854 | -6.754901273 | -2.899317054 |
| Sig_mark | *** | ** | *** | *** | ** |
| q-value | 2.08E-05 | 0.009917572 | 3.76E-09 | 1.00E-10 | 0.006544589 |
| fixp | <0.0001 | 0.0071 | <0.0001 | <0.0001 | 0.0037 |
| fixps | ***<0.0001 | ** 0.0071 | ***<0.0001 | ***<0.0001 | ** 0.0037 |

| Data S4. Comparison of oral mycobiome at the genus level between three groups | | | |
| --- | --- | --- | --- |
| ID | CPR0.median | CPR0.mean | CPR0.se |
| Zanclospora | 0.0495(0.0177,0.2225) | 0.184707909 | 0.05838904 |
| Aspergillus | 0.007(0.0025,0.0366) | 0.023146182 | 0.00704291 |
| Acrodictys | 0.0032(0.0015,0.0054) | 0.010992818 | 0.00601761 |
| Fungi_gen_Incertae_sedis | 0.0024(0.0011,0.0088) | 0.010212909 | 0.00464318 |
| Malassezia | 0.0146(0.0026,0.0758) | 0.051561909 | 0.0173357 |
| Actinomucor | 0.2475(0.0687,0.5681) | 0.335696682 | 0.06423864 |
| Entorrhiza | 0.0003(<0.0001,0.0008) | 0.0015075 | 6.99E-04 |
| Blumeria | <0.0001(<0.0001,<0.0001) | 2.05E-05 | 1.28E-05 |
| Cryptococcus | <0.0001(<0.0001,<0.0001) | 0 | 0 |
| Raffaelea | <0.0001(<0.0001,<0.0001) | 0.007326955 | 0.00725507 |
| Diversispora | <0.0001(<0.0001,0.0007) | 0.001314227 | 7.68E-04 |
| Arthrographis | <0.0001(<0.0001,<0.0001) | 2.90E-05 | 2.08E-05 |
| Heitmania | <0.0001(<0.0001,<0.0001) | 1.92E-05 | 8.97E-06 |
| Udeniomyces | 0.0003(<0.0001,0.0007) | 0.064740091 | 0.03298062 |
| Ascomycota_gen_Incertae_sedis | 0.002(0.0005,0.0048) | 0.014233091 | 0.00617141 |
| Milospium | <0.0001(<0.0001,<0.0001) | 5.67E-04 | 5.44E-04 |
| Talaromyces | <0.0001(<0.0001,<0.0001) | 0.010010227 | 0.0099602 |
| Alternaria | 0.0002(<0.0001,0.0028) | 0.013504136 | 0.00728191 |
| Wallemia | <0.0001(<0.0001,<0.0001) | 2.76E-04 | 2.00E-04 |
| Dipodascaceae_gen_Incertae_sedis | 0.0026(0.0006,0.0134) | 0.015057455 | 0.00663647 |
| Saprochaete | <0.0001(<0.0001,<0.0001) | 0 | 0 |
| Rhodotorula | 0.0082(0.001,0.0435) | 0.031055182 | 0.01041936 |
| Debaryomyces | <0.0001(<0.0001,<0.0001) | 0.030914773 | 0.03057571 |
| Parafuscosporella | <0.0001(<0.0001,<0.0001) | 8.16E-04 | 7.60E-04 |
| Dothiora | <0.0001(<0.0001,<0.0001) | 1.08E-04 | 6.35E-05 |
| Simplicillium | <0.0001(<0.0001,<0.0001) | 0.001620364 | 0.0015833 |
| Fusarium | 0.0001(<0.0001,0.0036) | 0.003169727 | 0.00109291 |
| Phaeosphaeria | <0.0001(<0.0001,<0.0001) | 8.34E-05 | 7.48E-05 |
| Aureobasidium | <0.0001(<0.0001,<0.0001) | 0.004582136 | 0.00388055 |
| Diaporthe | <0.0001(<0.0001,<0.0001) | 9.96E-05 | 6.92E-05 |
| Tuber | <0.0001(<0.0001,<0.0001) | 1.31E-04 | 1.12E-04 |
| Malasseziaceae_gen_Incertae_sedis | <0.0001(<0.0001,0.0002) | 0.001198682 | 6.39E-04 |
| Mucor | 0.0061(0.001,0.0157) | 0.013755227 | 0.0037478 |
| Botrytis | <0.0001(<0.0001,<0.0001) | 1.40E-04 | 1.38E-04 |
| Botryosphaeria | 0.0032(<0.0001,0.0135) | 0.011873045 | 0.00536691 |
| Ascochyta | <0.0001(<0.0001,<0.0001) | 8.94E-04 | 5.07E-04 |
| Sporobolomyces | <0.0001(<0.0001,0.0001) | 0.006518136 | 0.00379736 |
| Calosphaeria | <0.0001(<0.0001,<0.0001) | 1.85E-05 | 1.18E-05 |
| Bandonia | <0.0001(<0.0001,<0.0001) | 4.56E-05 | 2.96E-05 |
| Chrysosphaeria | <0.0001(<0.0001,<0.0001) | 0 | 0 |
| Dioszegia | <0.0001(<0.0001,<0.0001) | 0.007924636 | 0.00699225 |
| Togniniella | <0.0001(<0.0001,<0.0001) | 0 | 0 |
| Nigrospora | <0.0001(<0.0001,0.0011) | 0.002627318 | 0.00151986 |
| Bjerkandera | <0.0001(<0.0001,<0.0001) | 3.79E-04 | 3.79E-04 |
| Peniophora | <0.0001(<0.0001,<0.0001) | 0.002392455 | 0.00210755 |
| Kodamaea | <0.0001(<0.0001,<0.0001) | 0 | 0 |
| Saccharomyces | <0.0001(<0.0001,<0.0001) | 7.54E-04 | 6.60E-04 |
| Kazachstania | <0.0001(<0.0001,0.0041) | 0.003499273 | 0.00148498 |
| Angustimassarina | <0.0001(<0.0001,0.0003) | 3.89E-04 | 1.66E-04 |
| Rhexothecium | <0.0001(<0.0001,<0.0001) | 3.95E-06 | 3.95E-06 |
| Bovista | <0.0001(<0.0001,<0.0001) | 0 | 0 |
| Zygotorulaspora | <0.0001(<0.0001,<0.0001) | 3.45E-05 | 2.15E-05 |
| Pleosporales_gen_Incertae_sedis | <0.0001(<0.0001,<0.0001) | 1.31E-04 | 1.27E-04 |
| Gibberella | <0.0001(<0.0001,0.0025) | 0.002459773 | 0.00109524 |
| Coprinellus | <0.0001(<0.0001,<0.0001) | 0 | 0 |
| Cystofilobasidium | <0.0001(<0.0001,<0.0001) | 8.45E-06 | 8.45E-06 |
| Tausonia | <0.0001(<0.0001,<0.0001) | 6.95E-05 | 6.37E-05 |
| Ophiostoma | <0.0001(<0.0001,<0.0001) | 1.15E-05 | 8.05E-06 |
| Amylostereum | <0.0001(<0.0001,<0.0001) | 0 | 0 |
| Neopestalotiopsis | <0.0001(<0.0001,<0.0001) | 0 | 0 |
| Phanerochaete | <0.0001(<0.0001,<0.0001) | 2.42E-04 | 2.42E-04 |
| Diaporthales_gen_Incertae_sedis | <0.0001(<0.0001,<0.0001) | 0 | 0 |
| Tremellomycetes_gen_Incertae_sedis | <0.0001(<0.0001,<0.0001) | 9.06E-05 | 9.06E-05 |
| Fantasmomyces | <0.0001(<0.0001,<0.0001) | 1.48E-05 | 9.25E-06 |
| Trametes | <0.0001(<0.0001,<0.0001) | 2.44E-04 | 2.41E-04 |
| Apiotrichum | <0.0001(<0.0001,<0.0001) | 0 | 0 |
| Yarrowia | <0.0001(<0.0001,<0.0001) | 0 | 0 |
| Leptosporella | <0.0001(<0.0001,<0.0001) | 0.003747909 | 0.00373082 |
| Phyllachora | <0.0001(<0.0001,0.0034) | 0.0022135 | 7.47E-04 |
| Capnodiales_gen_Incertae_sedis | <0.0001(<0.0001,<0.0001) | 0 | 0 |
| Neosetophoma | <0.0001(<0.0001,<0.0001) | 0 | 0 |
| Ustilaginoidea | <0.0001(<0.0001,0.0054) | 0.003362 | 0.00125337 |
| Acremonium | <0.0001(<0.0001,<0.0001) | 2.83E-05 | 2.83E-05 |
| Peroneutypa | <0.0001(<0.0001,<0.0001) | 7.64E-06 | 5.34E-06 |
| Coprinopsis | <0.0001(<0.0001,<0.0001) | 1.51E-04 | 1.51E-04 |
| Auricularia | <0.0001(<0.0001,<0.0001) | 0 | 0 |
| Periconia | <0.0001(<0.0001,<0.0001) | 4.08E-04 | 4.05E-04 |
| Ceriporia | <0.0001(<0.0001,<0.0001) | 0 | 0 |
| Typhula | <0.0001(<0.0001,<0.0001) | 0 | 0 |
| Thielaviopsis | <0.0001(<0.0001,<0.0001) | 0 | 0 |
| Flavodon | <0.0001(<0.0001,<0.0001) | 0 | 0 |
| Dipodascus | <0.0001(<0.0001,0.0003) | 9.32E-04 | 5.71E-04 |
| Exophiala | <0.0001(<0.0001,<0.0001) | 4.21E-05 | 4.21E-05 |
| Novakomyces | <0.0001(<0.0001,<0.0001) | 0.001358818 | 9.47E-04 |
| Trichophaea | <0.0001(<0.0001,<0.0001) | 0.001505727 | 8.14E-04 |
| Tilletiopsis | <0.0001(<0.0001,<0.0001) | 0.001708364 | 0.00137089 |
| Diutina | <0.0001(<0.0001,<0.0001) | 8.65E-05 | 8.65E-05 |
| Cordyceps | <0.0001(<0.0001,<0.0001) | 9.48E-05 | 9.34E-05 |
| Keissleriella | <0.0001(<0.0001,<0.0001) | 0 | 0 |
| Zoophthora | <0.0001(<0.0001,0.0002) | 2.48E-04 | 1.17E-04 |
| Vishniacozyma | <0.0001(<0.0001,<0.0001) | 1.58E-04 | 1.58E-04 |
| Cercospora | <0.0001(<0.0001,<0.0001) | 1.04E-04 | 1.02E-04 |
| Exidia | <0.0001(<0.0001,<0.0001) | 0.001615818 | 0.00161406 |
| Neodevriesia | <0.0001(<0.0001,<0.0001) | 0 | 0 |
| Peniophorella | <0.0001(<0.0001,<0.0001) | 1.04E-05 | 1.04E-05 |
| Acrocalymma | <0.0001(<0.0001,<0.0001) | 0 | 0 |
| Onygenales_gen_Incertae_sedis | <0.0001(<0.0001,<0.0001) | 0.001196545 | 0.00119455 |
| Hypoxylon | <0.0001(<0.0001,<0.0001) | 0 | 0 |
| Tilletia | <0.0001(<0.0001,<0.0001) | 0 | 0 |
| Ciliophora | <0.0001(<0.0001,<0.0001) | 1.09E-05 | 9.33E-06 |
| Agaricales_gen_Incertae_sedis | <0.0001(<0.0001,<0.0001) | 5.48E-04 | 5.43E-04 |
| Filobasidiaceae_gen_Incertae_sedis | <0.0001(<0.0001,<0.0001) | 0 | 0 |
| Rosellinia | <0.0001(<0.0001,<0.0001) | 5.37E-04 | 4.58E-04 |

| Data S4. Comparison of oral mycobiome at the genus level between three groups | | | |
| --- | --- | --- | --- |
| ID | CPR1.median | CPR1.mean | CPR1.se |
| Zanclospora | 0.012(0.0007,0.0254) | 0.08105363 | 0.03914447 |
| Aspergillus | 0.0619(0.0092,0.1736) | 0.103940815 | 0.02048494 |
| Acrodictys | 0.0037(0.0006,0.0769) | 0.064523074 | 0.01957573 |
| Fungi_gen_Incertae_sedis | 0.104(0.0474,0.2228) | 0.145799037 | 0.02538101 |
| Malassezia | 0.1193(0.0422,0.2207) | 0.158709963 | 0.02899643 |
| Actinomucor | <0.0001(<0.0001,0.0004) | 9.82E-04 | 4.45E-04 |
| Entorrhiza | 0.0003(<0.0001,0.0555) | 0.048043222 | 0.02279807 |
| Blumeria | <0.0001(<0.0001,<0.0001) | 8.24E-04 | 4.64E-04 |
| Cryptococcus | <0.0001(<0.0001,<0.0001) | 2.96E-06 | 2.96E-06 |
| Raffaelea | <0.0001(<0.0001,<0.0001) | 2.35E-04 | 2.35E-04 |
| Diversispora | <0.0001(<0.0001,<0.0001) | 4.16E-04 | 2.92E-04 |
| Arthrographis | <0.0001(<0.0001,<0.0001) | 5.89E-06 | 5.89E-06 |
| Heitmania | <0.0001(<0.0001,0.0005) | 0.009904259 | 0.00599868 |
| Udeniomyces | <0.0001(<0.0001,<0.0001) | 0.002935556 | 0.00287655 |
| Ascomycota_gen_Incertae_sedis | <0.0001(<0.0001,0.0002) | 0.005749741 | 0.00309045 |
| Milospium | <0.0001(<0.0001,<0.0001) | 0.011125333 | 0.00849333 |
| Talaromyces | <0.0001(<0.0001,<0.0001) | 0.004646815 | 0.00305295 |
| Alternaria | <0.0001(<0.0001,<0.0001) | 0.003827296 | 0.00266726 |
| Wallemia | <0.0001(<0.0001,<0.0001) | 0.015740111 | 0.01457066 |
| Dipodascaceae_gen_Incertae_sedis | <0.0001(<0.0001,<0.0001) | 0.019001556 | 0.01641529 |
| Saprochaete | <0.0001(<0.0001,<0.0001) | 0 | 0 |
| Rhodotorula | <0.0001(<0.0001,<0.0001) | 0.001761444 | 0.00124025 |
| Debaryomyces | <0.0001(<0.0001,<0.0001) | 1.78E-06 | 1.78E-06 |
| Parafuscosporella | <0.0001(<0.0001,0.0024) | 0.002120815 | 8.77E-04 |
| Dothiora | <0.0001(<0.0001,0.0036) | 0.005466556 | 0.00233852 |
| Simplicillium | 0.0004(<0.0001,0.0018) | 0.00432737 | 0.00256741 |
| Fusarium | <0.0001(<0.0001,<0.0001) | 0.005327667 | 0.00346044 |
| Phaeosphaeria | <0.0001(<0.0001,<0.0001) | 7.95E-04 | 7.95E-04 |
| Aureobasidium | <0.0001(<0.0001,0.0001) | 0.010784593 | 0.01068296 |
| Diaporthe | <0.0001(<0.0001,<0.0001) | 0 | 0 |
| Tuber | <0.0001(<0.0001,0.0003) | 2.14E-04 | 9.11E-05 |
| Malasseziaceae_gen_Incertae_sedis | <0.0001(<0.0001,<0.0001) | 0.004315074 | 0.00431507 |
| Mucor | <0.0001(<0.0001,<0.0001) | 3.92E-04 | 3.68E-04 |
| Botrytis | <0.0001(<0.0001,<0.0001) | 0.007715667 | 0.00527329 |
| Botryosphaeria | <0.0001(<0.0001,<0.0001) | 6.76E-04 | 6.26E-04 |
| Ascochyta | <0.0001(<0.0001,<0.0001) | 0.009693926 | 0.0072724 |
| Sporobolomyces | <0.0001(<0.0001,<0.0001) | 1.19E-06 | 1.19E-06 |
| Calosphaeria | <0.0001(<0.0001,0.0003) | 5.93E-04 | 3.35E-04 |
| Bandonia | <0.0001(<0.0001,0.0002) | 0.007030222 | 0.00463703 |
| Chrysosphaeria | <0.0001(<0.0001,<0.0001) | 1.80E-04 | 1.53E-04 |
| Dioszegia | <0.0001(<0.0001,<0.0001) | 1.04E-05 | 5.71E-06 |
| Togniniella | <0.0001(<0.0001,<0.0001) | 0 | 0 |
| Nigrospora | <0.0001(<0.0001,<0.0001) | 0.002271852 | 0.00222689 |
| Bjerkandera | <0.0001(<0.0001,<0.0001) | 0 | 0 |
| Peniophora | <0.0001(<0.0001,<0.0001) | 0 | 0 |
| Kodamaea | <0.0001(<0.0001,<0.0001) | 0.006127519 | 0.00427332 |
| Saccharomyces | <0.0001(<0.0001,<0.0001) | 1.50E-05 | 9.37E-06 |
| Kazachstania | <0.0001(<0.0001,<0.0001) | 6.43E-04 | 6.11E-04 |
| Angustimassarina | <0.0001(<0.0001,<0.0001) | 4.62E-04 | 2.68E-04 |
| Rhexothecium | <0.0001(<0.0001,<0.0001) | 0 | 0 |
| Bovista | <0.0001(<0.0001,<0.0001) | 3.10E-05 | 1.59E-05 |
| Zygotorulaspora | <0.0001(<0.0001,0.0002) | 5.23E-04 | 3.89E-04 |
| Pleosporales_gen_Incertae_sedis | <0.0001(<0.0001,<0.0001) | 0 | 0 |
| Gibberella | <0.0001(<0.0001,<0.0001) | 0.003155 | 0.00197249 |
| Coprinellus | <0.0001(<0.0001,<0.0001) | 0 | 0 |
| Cystofilobasidium | <0.0001(<0.0001,<0.0001) | 0 | 0 |
| Tausonia | <0.0001(<0.0001,<0.0001) | 0.001839444 | 0.00183944 |
| Ophiostoma | <0.0001(<0.0001,<0.0001) | 2.57E-05 | 1.79E-05 |
| Amylostereum | <0.0001(<0.0001,<0.0001) | 0.004501667 | 0.00448637 |
| Neopestalotiopsis | <0.0001(<0.0001,<0.0001) | 0.004455 | 0.00445308 |
| Phanerochaete | <0.0001(<0.0001,<0.0001) | 0.00423363 | 0.00362616 |
| Diaporthales_gen_Incertae_sedis | <0.0001(<0.0001,<0.0001) | 0 | 0 |
| Tremellomycetes_gen_Incertae_sedis | <0.0001(<0.0001,<0.0001) | 0 | 0 |
| Fantasmomyces | <0.0001(<0.0001,0.0002) | 6.53E-04 | 4.12E-04 |
| Trametes | <0.0001(<0.0001,<0.0001) | 0 | 0 |
| Apiotrichum | <0.0001(<0.0001,<0.0001) | 0 | 0 |
| Yarrowia | <0.0001(<0.0001,<0.0001) | 0.001213222 | 6.87E-04 |
| Leptosporella | <0.0001(<0.0001,<0.0001) | 5.49E-05 | 5.15E-05 |
| Phyllachora | <0.0001(<0.0001,<0.0001) | 0 | 0 |
| Capnodiales_gen_Incertae_sedis | <0.0001(<0.0001,<0.0001) | 0 | 0 |
| Neosetophoma | <0.0001(<0.0001,<0.0001) | 0 | 0 |
| Ustilaginoidea | <0.0001(<0.0001,<0.0001) | 0 | 0 |
| Acremonium | <0.0001(<0.0001,<0.0001) | 3.72E-04 | 3.72E-04 |
| Peroneutypa | <0.0001(<0.0001,<0.0001) | 0.002494667 | 0.00249313 |
| Coprinopsis | <0.0001(<0.0001,<0.0001) | 0 | 0 |
| Auricularia | <0.0001(<0.0001,<0.0001) | 0 | 0 |
| Periconia | <0.0001(<0.0001,<0.0001) | 7.96E-05 | 6.59E-05 |
| Ceriporia | <0.0001(<0.0001,<0.0001) | 6.71E-04 | 6.71E-04 |
| Typhula | <0.0001(<0.0001,<0.0001) | 0 | 0 |
| Thielaviopsis | <0.0001(<0.0001,<0.0001) | 1.36E-04 | 1.36E-04 |
| Flavodon | <0.0001(<0.0001,<0.0001) | 0 | 0 |
| Dipodascus | <0.0001(<0.0001,<0.0001) | 7.11E-05 | 6.93E-05 |
| Exophiala | <0.0001(<0.0001,<0.0001) | 0.001742148 | 0.00173643 |
| Novakomyces | <0.0001(<0.0001,<0.0001) | 6.93E-06 | 5.27E-06 |
| Trichophaea | <0.0001(<0.0001,<0.0001) | 4.00E-04 | 3.97E-04 |
| Tilletiopsis | <0.0001(<0.0001,<0.0001) | 0 | 0 |
| Diutina | <0.0001(<0.0001,<0.0001) | 6.63E-06 | 5.17E-06 |
| Cordyceps | <0.0001(<0.0001,<0.0001) | 7.06E-05 | 4.43E-05 |
| Keissleriella | <0.0001(<0.0001,<0.0001) | 0 | 0 |
| Zoophthora | <0.0001(<0.0001,<0.0001) | 0 | 0 |
| Vishniacozyma | <0.0001(<0.0001,<0.0001) | 0 | 0 |
| Cercospora | <0.0001(<0.0001,<0.0001) | 0 | 0 |
| Exidia | <0.0001(<0.0001,<0.0001) | 0 | 0 |
| Neodevriesia | <0.0001(<0.0001,<0.0001) | 0.001271593 | 0.00105783 |
| Peniophorella | <0.0001(<0.0001,<0.0001) | 0 | 0 |
| Acrocalymma | <0.0001(<0.0001,<0.0001) | 0 | 0 |
| Onygenales_gen_Incertae_sedis | <0.0001(<0.0001,<0.0001) | 0 | 0 |
| Hypoxylon | <0.0001(<0.0001,<0.0001) | 0 | 0 |
| Tilletia | <0.0001(<0.0001,<0.0001) | 0 | 0 |
| Ciliophora | <0.0001(<0.0001,<0.0001) | 0 | 0 |
| Agaricales_gen_Incertae_sedis | <0.0001(<0.0001,<0.0001) | 4.81E-06 | 4.81E-06 |
| Filobasidiaceae_gen_Incertae_sedis | <0.0001(<0.0001,<0.0001) | 0 | 0 |
| Rosellinia | <0.0001(<0.0001,<0.0001) | 0 | 0 |

| Data S4. Comparison of oral mycobiome at the genus level between three groups | | | |
| --- | --- | --- | --- |
| ID | HC.median | HC.mean | HC.se |
| Zanclospora | 0.0755(0.0199,0.3217) | 0.210945633 | 0.02680138 |
| Aspergillus | 0.0268(0.006,0.1122) | 0.104383667 | 0.01782922 |
| Acrodictys | 0.0323(0.0066,0.1606) | 0.101940244 | 0.01409439 |
| Fungi_gen_Incertae_sedis | 0.0428(0.0195,0.063) | 0.055815311 | 0.00713223 |
| Malassezia | 0.0085(0.0016,0.0399) | 0.033874256 | 0.00727867 |
| Actinomucor | <0.0001(<0.0001,<0.0001) | 2.65E-05 | 6.60E-06 |
| Entorrhiza | 0.0033(0.0016,0.0375) | 0.057756578 | 0.01157468 |
| Blumeria | 0.0015(0.0007,0.0063) | 0.045723011 | 0.01306007 |
| Cryptococcus | 0.0093(0.0016,0.0391) | 0.036513811 | 0.00631469 |
| Raffaelea | 0.0004(<0.0001,0.0012) | 0.030810478 | 0.01363513 |
| Diversispora | 0.0013(0.0003,0.0072) | 0.030039222 | 0.01260758 |
| Arthrographis | 0.0003(<0.0001,0.0008) | 0.022962267 | 0.01277248 |
| Heitmania | 0.0008(0.0003,0.0023) | 0.017578344 | 0.00491759 |
| Udeniomyces | <0.0001(<0.0001,<0.0001) | 0.003243222 | 0.00273752 |
| Ascomycota_gen_Incertae_sedis | 0.0018(0.0006,0.0093) | 0.014598311 | 0.00361466 |
| Milospium | <0.0001(<0.0001,0.0007) | 0.015415089 | 0.00983051 |
| Talaromyces | 0.0002(<0.0001,0.001) | 0.014155689 | 0.00457448 |
| Alternaria | 0.0006(0.0001,0.0051) | 0.011155422 | 0.00403571 |
| Wallemia | <0.0001(<0.0001,0.0003) | 0.007305711 | 0.00569663 |
| Dipodascaceae_gen_Incertae_sedis | <0.0001(<0.0001,0.0008) | 5.36E-04 | 1.03E-04 |
| Saprochaete | <0.0001(<0.0001,<0.0001) | 0.008890656 | 0.00863655 |
| Rhodotorula | <0.0001(<0.0001,<0.0001) | 3.71E-04 | 1.58E-04 |
| Debaryomyces | <0.0001(<0.0001,0.0001) | 6.51E-04 | 3.85E-04 |
| Parafuscosporella | 0.0023(0.0003,0.0131) | 0.007147167 | 9.49E-04 |
| Dothiora | 0.0003(0.0001,0.0036) | 0.005962189 | 0.0014697 |
| Simplicillium | 0.0003(0.0001,0.0026) | 0.004459333 | 9.39E-04 |
| Fusarium | <0.0001(<0.0001,0.0002) | 0.003044433 | 0.00120451 |
| Phaeosphaeria | <0.0001(<0.0001,0.0003) | 0.004924044 | 0.00328199 |
| Aureobasidium | <0.0001(<0.0001,<0.0001) | 5.57E-04 | 4.47E-04 |
| Diaporthe | <0.0001(<0.0001,0.0001) | 0.004047978 | 0.00314827 |
| Tuber | 0.0001(<0.0001,0.0007) | 0.003741267 | 0.00193903 |
| Malasseziaceae_gen_Incertae_sedis | <0.0001(<0.0001,0.0002) | 0.002103222 | 6.63E-04 |
| Mucor | <0.0001(<0.0001,<0.0001) | 8.85E-05 | 2.13E-05 |
| Botrytis | <0.0001(<0.0001,<0.0001) | 0.001182278 | 7.22E-04 |
| Botryosphaeria | <0.0001(<0.0001,<0.0001) | 3.16E-04 | 3.02E-04 |
| Ascochyta | <0.0001(<0.0001,<0.0001) | 6.85E-05 | 4.41E-05 |
| Sporobolomyces | <0.0001(<0.0001,<0.0001) | 0.001138 | 5.88E-04 |
| Calosphaeria | 0.0001(<0.0001,0.0017) | 0.0023836 | 4.96E-04 |
| Bandonia | <0.0001(<0.0001,<0.0001) | 3.84E-04 | 3.28E-04 |
| Chrysosphaeria | <0.0001(<0.0001,0.0002) | 0.002012467 | 0.00128631 |
| Dioszegia | <0.0001(<0.0001,<0.0001) | 1.05E-04 | 8.60E-05 |
| Togniniella | <0.0001(<0.0001,<0.0001) | 0.0020321 | 0.00117772 |
| Nigrospora | <0.0001(<0.0001,<0.0001) | 6.81E-04 | 4.83E-04 |
| Bjerkandera | <0.0001(<0.0001,<0.0001) | 0.0019042 | 0.00180368 |
| Peniophora | <0.0001(<0.0001,<0.0001) | 0.001255978 | 8.09E-04 |
| Kodamaea | <0.0001(<0.0001,<0.0001) | 0 | 0 |
| Saccharomyces | <0.0001(<0.0001,0.0003) | 0.001554967 | 6.08E-04 |
| Kazachstania | <0.0001(<0.0001,<0.0001) | 6.92E-04 | 5.17E-04 |
| Angustimassarina | <0.0001(<0.0001,0.001) | 0.001486744 | 3.89E-04 |
| Rhexothecium | <0.0001(<0.0001,0.0002) | 0.001690344 | 0.00112444 |
| Bovista | <0.0001(<0.0001,0.0004) | 0.0016021 | 7.96E-04 |
| Zygotorulaspora | <0.0001(<0.0001,0.0002) | 0.001421178 | 8.06E-04 |
| Pleosporales_gen_Incertae_sedis | <0.0001(<0.0001,<0.0001) | 0.0015339 | 0.00105416 |
| Gibberella | <0.0001(<0.0001,<0.0001) | 1.58E-05 | 7.01E-06 |
| Coprinellus | <0.0001(<0.0001,<0.0001) | 0.001527922 | 9.05E-04 |
| Cystofilobasidium | <0.0001(<0.0001,<0.0001) | 0.001523222 | 0.00112588 |
| Tausonia | <0.0001(<0.0001,<0.0001) | 9.16E-04 | 5.47E-04 |
| Ophiostoma | <0.0001(<0.0001,0.0002) | 0.001457878 | 7.01E-04 |
| Amylostereum | <0.0001(<0.0001,<0.0001) | 0 | 0 |
| Neopestalotiopsis | <0.0001(<0.0001,<0.0001) | 0 | 0 |
| Phanerochaete | <0.0001(<0.0001,<0.0001) | 8.00E-07 | 8.00E-07 |
| Diaporthales_gen_Incertae_sedis | <0.0001(<0.0001,<0.0001) | 0.001322878 | 9.79E-04 |
| Tremellomycetes_gen_Incertae_sedis | <0.0001(<0.0001,<0.0001) | 0.001179233 | 0.00116005 |
| Fantasmomyces | <0.0001(<0.0001,0.0005) | 9.68E-04 | 3.05E-04 |
| Trametes | <0.0001(<0.0001,<0.0001) | 0.001069289 | 5.28E-04 |
| Apiotrichum | <0.0001(<0.0001,0.0001) | 0.001092511 | 5.70E-04 |
| Yarrowia | <0.0001(<0.0001,<0.0001) | 6.10E-04 | 3.63E-04 |
| Leptosporella | <0.0001(<0.0001,<0.0001) | 3.53E-06 | 3.53E-06 |
| Phyllachora | <0.0001(<0.0001,<0.0001) | 3.33E-04 | 1.76E-04 |
| Capnodiales_gen_Incertae_sedis | <0.0001(<0.0001,<0.0001) | 8.48E-04 | 7.09E-04 |
| Neosetophoma | <0.0001(<0.0001,<0.0001) | 8.45E-04 | 6.08E-04 |
| Ustilaginoidea | <0.0001(<0.0001,<0.0001) | 1.43E-05 | 5.25E-06 |
| Acremonium | <0.0001(<0.0001,<0.0001) | 7.05E-04 | 5.02E-04 |
| Peroneutypa | <0.0001(<0.0001,<0.0001) | 0 | 0 |
| Coprinopsis | <0.0001(<0.0001,<0.0001) | 7.10E-04 | 4.51E-04 |
| Auricularia | <0.0001(<0.0001,<0.0001) | 7.14E-04 | 4.40E-04 |
| Periconia | <0.0001(<0.0001,<0.0001) | 5.47E-04 | 2.63E-04 |
| Ceriporia | <0.0001(<0.0001,<0.0001) | 4.18E-04 | 3.07E-04 |
| Typhula | <0.0001(<0.0001,<0.0001) | 5.93E-04 | 5.08E-04 |
| Thielaviopsis | <0.0001(<0.0001,<0.0001) | 5.47E-04 | 3.46E-04 |
| Flavodon | <0.0001(<0.0001,<0.0001) | 5.84E-04 | 5.41E-04 |
| Dipodascus | <0.0001(<0.0001,0.0004) | 3.12E-04 | 6.47E-05 |
| Exophiala | <0.0001(<0.0001,<0.0001) | 0 | 0 |
| Novakomyces | <0.0001(<0.0001,<0.0001) | 1.78E-04 | 1.51E-04 |
| Trichophaea | <0.0001(<0.0001,<0.0001) | 1.26E-05 | 1.04E-05 |
| Tilletiopsis | <0.0001(<0.0001,<0.0001) | 7.91E-05 | 6.66E-05 |
| Diutina | <0.0001(<0.0001,<0.0001) | 4.73E-04 | 2.04E-04 |
| Cordyceps | <0.0001(<0.0001,<0.0001) | 4.29E-04 | 3.69E-04 |
| Keissleriella | <0.0001(<0.0001,<0.0001) | 4.52E-04 | 4.37E-04 |
| Zoophthora | <0.0001(<0.0001,<0.0001) | 3.73E-04 | 1.60E-04 |
| Vishniacozyma | <0.0001(<0.0001,<0.0001) | 3.78E-04 | 2.72E-04 |
| Cercospora | <0.0001(<0.0001,<0.0001) | 3.80E-04 | 1.79E-04 |
| Exidia | <0.0001(<0.0001,<0.0001) | 0 | 0 |
| Neodevriesia | <0.0001(<0.0001,<0.0001) | 0 | 0 |
| Peniophorella | <0.0001(<0.0001,<0.0001) | 3.77E-04 | 3.60E-04 |
| Acrocalymma | <0.0001(<0.0001,<0.0001) | 3.34E-04 | 3.20E-04 |
| Onygenales_gen_Incertae_sedis | <0.0001(<0.0001,<0.0001) | 4.13E-06 | 4.13E-06 |
| Hypoxylon | <0.0001(<0.0001,<0.0001) | 2.74E-04 | 2.57E-04 |
| Tilletia | <0.0001(<0.0001,<0.0001) | 2.66E-04 | 1.54E-04 |
| Ciliophora | <0.0001(<0.0001,<0.0001) | 2.11E-04 | 1.37E-04 |
| Agaricales_gen_Incertae_sedis | <0.0001(<0.0001,<0.0001) | 0 | 0 |
| Filobasidiaceae_gen_Incertae_sedis | <0.0001(<0.0001,<0.0001) | 1.34E-04 | 1.13E-04 |
| Rosellinia | <0.0001(<0.0001,<0.0001) | 0 | 0 |

| Data S4. Comparison of oral mycobiome at the genus level between three groups | | | | | |
| --- | --- | --- | --- | --- | --- |
| ID | p-value | z-score | Sig_mark | q-value | fixp |
| Zanclospora | 5.07E-05 | -4.052587706 | *** | 4.84E-04 | <0.0001 |
| Aspergillus | 0.011511091 | -2.526788155 | * | 0.04748325 | 0.0115 |
| Acrodictys | 1.85E-06 | -4.769348696 | *** | 2.68E-05 | <0.0001 |
| Fungi_gen_Incertae_sedis | 1.54E-10 | -6.401030294 | *** | 5.87E-09 | <0.0001 |
| Malassezia | 3.47E-05 | -4.140345146 | *** | 3.60E-04 | <0.0001 |
| Actinomucor | 1.70E-17 | -8.51241211 | *** | 3.09E-15 | <0.0001 |
| Entorrhiza | 1.25E-06 | -4.847892124 | *** | 1.97E-05 | <0.0001 |
| Blumeria | 3.29E-16 | -8.162334406 | *** | 3.98E-14 | <0.0001 |
| Cryptococcus | 3.96E-22 | -9.672250912 | *** | 1.44E-19 | <0.0001 |
| Raffaelea | 9.20E-11 | -6.479509777 | *** | 4.18E-09 | <0.0001 |
| Diversispora | 1.62E-10 | -6.39387835 | *** | 5.87E-09 | <0.0001 |
| Arthrographis | 6.93E-11 | -6.522109185 | *** | 3.60E-09 | <0.0001 |
| Heitmania | 2.22E-12 | -7.019625496 | *** | 1.62E-10 | <0.0001 |
| Udeniomyces | 5.24E-05 | -4.044653836 | *** | 4.88E-04 | <0.0001 |
| Ascomycota_gen_Incertae_sedis | 1.76E-06 | -4.779387147 | *** | 2.66E-05 | <0.0001 |
| Milospium | 1.06E-04 | -3.876677457 | *** | 9.61E-04 | 0.0001 |
| Talaromyces | 8.88E-09 | -5.750836643 | *** | 2.30E-07 | <0.0001 |
| Alternaria | 3.37E-07 | -5.101485812 | *** | 5.89E-06 | <0.0001 |
| Wallemia | 0.004540577 | -2.837939613 | ** | 0.02377828 | 0.0045 |
| Dipodascaceae_gen_Incertae_sedis | 3.63E-07 | -5.08759967 | *** | 5.98E-06 | <0.0001 |
| Saprochaete | 0.010837385 | -2.547898967 | * | 0.04521806 | 0.0108 |
| Rhodotorula | 4.76E-12 | -6.912689277 | *** | 2.88E-10 | <0.0001 |
| Debaryomyces | 2.49E-04 | -3.662824316 | *** | 0.00201223 | 0.0002 |
| Parafuscosporella | 1.70E-09 | -6.024046532 | *** | 5.61E-08 | <0.0001 |
| Dothiora | 2.78E-06 | -4.68633648 | *** | 3.88E-05 | <0.0001 |
| Simplicillium | 1.63E-05 | -4.309858956 | *** | 1.91E-04 | <0.0001 |
| Fusarium | 0.04344426 | -2.019413492 | * | 0.12616213 | 0.0434 |
| Phaeosphaeria | 0.007014995 | -2.69613159 | ** | 0.03223346 | 0.007 |
| Aureobasidium | 0.033221199 | -2.129399777 | * | 0.10872871 | 0.0332 |
| Diaporthe | 1.54E-04 | -3.784263166 | *** | 0.00133242 | 0.0002 |
| Tuber | 3.65E-05 | -4.128656168 | *** | 3.68E-04 | <0.0001 |
| Malasseziaceae_gen_Incertae_sedis | 8.56E-06 | -4.450635611 | *** | 1.07E-04 | <0.0001 |
| Mucor | 2.03E-12 | -7.032739006 | *** | 1.62E-10 | <0.0001 |
| Botrytis | 0.018601074 | -2.353430714 | * | 0.0699386 | 0.0186 |
| Botryosphaeria | 4.19E-09 | -5.876374459 | *** | 1.27E-07 | <0.0001 |
| Ascochyta | 0.043116768 | -2.022577089 | * | 0.12616213 | 0.0431 |
| Sporobolomyces | 0.002130058 | -3.071472463 | ** | 0.01299943 | 0.0021 |
| Calosphaeria | 6.61E-08 | -5.401474463 | *** | 1.50E-06 | <0.0001 |
| Bandonia | 0.032575807 | -2.137272834 | * | 0.1084864 | 0.0326 |
| Chrysosphaeria | 3.41E-07 | -5.099499065 | *** | 5.89E-06 | <0.0001 |
| Dioszegia | 0.01445073 | -2.445859511 | * | 0.05787996 | 0.0145 |
| Togniniella | 0.001325003 | -3.210508589 | ** | 0.00845725 | 0.0013 |
| Nigrospora | 0.003860859 | -2.88931195 | ** | 0.02123472 | 0.0039 |
| Bjerkandera | 9.46E-04 | -3.306002629 | *** | 0.00648176 | 0.0009 |
| Peniophora | 0.003228232 | -2.945125998 | ** | 0.01868231 | 0.0032 |
| Kodamaea | 0.015325959 | -2.424583476 | * | 0.05918429 | 0.0153 |
| Saccharomyces | 2.74E-05 | -4.19371999 | *** | 3.02E-04 | <0.0001 |
| Kazachstania | 0.027123206 | -2.209740079 | * | 0.09288419 | 0.0271 |
| Angustimassarina | 0.005481374 | -2.777293023 | ** | 0.02587094 | 0.0055 |
| Rhexothecium | 5.95E-06 | -4.528212349 | *** | 7.94E-05 | <0.0001 |
| Bovista | 1.65E-07 | -5.235461915 | *** | 3.43E-06 | <0.0001 |
| Zygotorulaspora | 0.005362376 | -2.784418721 | ** | 0.02587094 | 0.0054 |
| Pleosporales_gen_Incertae_sedis | 3.36E-05 | -4.147726265 | *** | 3.59E-04 | <0.0001 |
| Gibberella | 0.03150191 | -2.150674383 | * | 0.10588142 | 0.0315 |
| Coprinellus | 3.95E-05 | -4.110357357 | *** | 3.88E-04 | <0.0001 |
| Cystofilobasidium | 0.003242384 | -2.943772392 | ** | 0.01868231 | 0.0032 |
| Tausonia | 0.021036703 | -2.307325107 | * | 0.07486591 | 0.021 |
| Ophiostoma | 8.99E-06 | -4.440059345 | *** | 1.09E-04 | <0.0001 |
| Amylostereum | 2.08E-04 | -3.709204769 | *** | 0.00171527 | 0.0002 |
| Neopestalotiopsis | 0.015325959 | -2.424583476 | * | 0.05918429 | 0.0153 |
| Phanerochaete | 0.008332437 | -2.638293728 | ** | 0.03688628 | 0.0083 |
| Diaporthales_gen_Incertae_sedis | 0.020992871 | -2.308112665 | * | 0.07486591 | 0.021 |
| Tremellomycetes_gen_Incertae_sedis | 0.014509853 | -2.444386987 | * | 0.05787996 | 0.0145 |
| Fantasmomyces | 5.19E-04 | -3.47054224 | *** | 0.00369697 | 0.0005 |
| Trametes | 0.001327998 | -3.209859725 | ** | 0.00845725 | 0.0013 |
| Apiotrichum | 1.70E-07 | -5.22931058 | *** | 3.43E-06 | <0.0001 |
| Yarrowia | 0.001212895 | -3.235830267 | ** | 0.00815335 | 0.0012 |
| Leptosporella | 6.12E-06 | -4.522100825 | *** | 7.94E-05 | <0.0001 |
| Phyllachora | 1.52E-04 | -3.787360578 | *** | 0.00133242 | 0.0002 |
| Capnodiales_gen_Incertae_sedis | 0.010837008 | -2.547911125 | * | 0.04521806 | 0.0108 |
| Neosetophoma | 0.010837385 | -2.547898967 | * | 0.04521806 | 0.0108 |
| Ustilaginoidea | 2.84E-07 | -5.133495645 | *** | 5.43E-06 | <0.0001 |
| Acremonium | 0.002148667 | -3.068874745 | ** | 0.01299943 | 0.0021 |
| Peroneutypa | 0.022035342 | -2.289758121 | * | 0.07765854 | 0.022 |
| Coprinopsis | 0.010143944 | -2.570883513 | * | 0.04436448 | 0.0101 |
| Auricularia | 0.005487578 | -2.776925362 | ** | 0.02587094 | 0.0055 |
| Periconia | 0.033247622 | -2.129080248 | * | 0.10872871 | 0.0332 |
| Ceriporia | 0.00566331 | -2.766663941 | ** | 0.02635617 | 0.0057 |
| Typhula | 6.31E-04 | -3.417919401 | *** | 0.00440499 | 0.0006 |
| Thielaviopsis | 0.037862014 | -2.076345436 | * | 0.11874679 | 0.0379 |
| Flavodon | 0.020992871 | -2.308112665 | * | 0.07486591 | 0.021 |
| Dipodascus | 0.005122598 | -2.799221555 | ** | 0.02547264 | 0.0051 |
| Exophiala | 0.048383289 | -1.973986406 | * | 0.13678467 | 0.0484 |
| Novakomyces | 0.040630992 | -2.047276005 | * | 0.11991098 | 0.0406 |
| Trichophaea | 0.011973625 | -2.512920683 | * | 0.04883625 | 0.012 |
| Tilletiopsis | 0.037187889 | -2.083695338 | * | 0.11874679 | 0.0372 |
| Diutina | 2.84E-04 | -3.629872107 | *** | 0.00223767 | 0.0003 |
| Cordyceps | 0.036465882 | -2.091694177 | * | 0.11818853 | 0.0365 |
| Keissleriella | 0.020992871 | -2.308112665 | * | 0.07486591 | 0.021 |
| Zoophthora | 0.003739765 | -2.899317054 | ** | 0.02088515 | 0.0037 |
| Vishniacozyma | 0.001898688 | -3.105638298 | ** | 0.01188317 | 0.0019 |
| Cercospora | 0.007450214 | -2.676020268 | ** | 0.03380535 | 0.0075 |
| Exidia | 0.004716353 | -2.825794345 | ** | 0.02377828 | 0.0047 |
| Neodevriesia | 0.015325959 | -2.424583476 | * | 0.05918429 | 0.0153 |
| Peniophorella | 0.048609429 | -1.972001552 | * | 0.13678467 | 0.0486 |
| Acrocalymma | 0.010837385 | -2.547898967 | * | 0.04521806 | 0.0108 |
| Onygenales_gen_Incertae_sedis | 0.049155174 | -1.967243249 | * | 0.13725637 | 0.0492 |
| Hypoxylon | 0.029010983 | -2.183337251 | * | 0.09842044 | 0.029 |
| Tilletia | 4.32E-04 | -3.519759727 | *** | 0.00319987 | 0.0004 |
| Ciliophora | 0.046155926 | -1.993964578 | * | 0.13297302 | 0.0462 |
| Agaricales_gen_Incertae_sedis | 0.0267255 | -2.215503849 | * | 0.09239387 | 0.0267 |
| Filobasidiaceae_gen_Incertae_sedis | 0.005487775 | -2.77691369 | ** | 0.02587094 | 0.0055 |
| Rosellinia | 3.06E-04 | -3.610560757 | *** | 0.00231061 | 0.0003 |

| Data S5. Comparison of oral mycobiome at the species level between three groups | | | |
| --- | --- | --- | --- |
| ID | CPR0.median | CPR0.mean | CPR0.se |
| Zanclospora_jonesii | 0.0491(0.0176,0.2219) | 0.184597091 | 0.058382515 |
| Acrodictys_fluminicola | 0.0032(0.0015,0.0054) | 0.010992818 | 0.006017614 |
| Aspergillus_subflavus | 0.0003(<0.0001,0.0015) | 0.004864091 | 0.002670321 |
| Fungi_spe_Incertae_sedis | 0.0024(0.0011,0.0088) | 0.010212909 | 0.004643185 |
| Actinomucor_elegans | 0.2475(0.0687,0.5681) | 0.335696682 | 0.064238636 |
| Entorrhiza_citriformis | 0.0002(<0.0001,0.0007) | 0.001461182 | 7.02E-04 |
| Malassezia_restricta | 0.0093(0.0012,0.0441) | 0.024374318 | 0.007168128 |
| Blumeria_spe_Incertae_sedis | <0.0001(<0.0001,<0.0001) | 2.05E-05 | 1.28E-05 |
| Cryptococcus_longus | <0.0001(<0.0001,<0.0001) | 0 | 0 |
| Raffaelea_lauricola | <0.0001(<0.0001,<0.0001) | 0.007326955 | 0.007255072 |
| Diversispora_spurca | <0.0001(<0.0001,0.0007) | 0.001314227 | 7.68E-04 |
| Arthrographis_grakistii | <0.0001(<0.0001,<0.0001) | 2.90E-05 | 2.08E-05 |
| Heitmania_litseae | <0.0001(<0.0001,<0.0001) | 1.92E-05 | 8.97E-06 |
| Ascomycota_spe_Incertae_sedis | 0.002(0.0005,0.0048) | 0.014233091 | 0.006171405 |
| Milospium_graphideorum | <0.0001(<0.0001,<0.0001) | 5.67E-04 | 5.44E-04 |
| Aspergillus_penicillioides | 0.0035(0.0008,0.0075) | 0.010384227 | 0.003511077 |
| Candida_albicans | 0.0004(<0.0001,0.0026) | 0.059654864 | 0.045986385 |
| Alternaria_alternata | 0.0002(<0.0001,0.0028) | 0.013428 | 0.007238126 |
| Talaromyces_scorteus | <0.0001(<0.0001,<0.0001) | 0.010008045 | 0.009960309 |
| Candida_tropicalis | <0.0001(<0.0001,<0.0001) | 3.60E-05 | 3.60E-05 |
| Udeniomyces_megalosporus | 0.0002(<0.0001,0.0003) | 0.035201591 | 0.018325899 |
| Dipodascaceae_spe_Incertae_sedis | 0.0026(0.0006,0.0134) | 0.015057455 | 0.006636473 |
| Cladosporium_halotolerans | 0.0003(<0.0001,0.0054) | 0.005705273 | 0.002273287 |
| Saprochaete_gigas | <0.0001(<0.0001,<0.0001) | 0 | 0 |
| Rhodotorula_mucilaginosa | 0.0082(0.001,0.0435) | 0.030954909 | 0.010425493 |
| Debaryomyces_prosopidis | <0.0001(<0.0001,<0.0001) | 0.030914773 | 0.030575711 |
| Parafuscosporella_mucosa | <0.0001(<0.0001,<0.0001) | 8.16E-04 | 7.60E-04 |
| Dothiora_spartii | <0.0001(<0.0001,<0.0001) | 1.08E-04 | 6.35E-05 |
| Udeniomyces_pyricola | <0.0001(<0.0001,0.0002) | 0.0295385 | 0.019828131 |
| Aspergillus_amstelodami | <0.0001(<0.0001,<0.0001) | 6.46E-04 | 6.01E-04 |
| Candida_parapsilosis | <0.0001(<0.0001,<0.0001) | 2.90E-05 | 2.07E-05 |
| Simplicillium_sympodiophorum | <0.0001(<0.0001,<0.0001) | 3.33E-05 | 1.99E-05 |
| Wallemia_spe_Incertae_sedis | <0.0001(<0.0001,<0.0001) | 1.84E-04 | 1.84E-04 |
| Phaeosphaeria_oryzae | <0.0001(<0.0001,<0.0001) | 8.34E-05 | 7.48E-05 |
| Aureobasidium_pullulans | <0.0001(<0.0001,<0.0001) | 0.004582136 | 0.003880547 |
| Candida_spe_Incertae_sedis | <0.0001(<0.0001,<0.0001) | 4.18E-04 | 2.96E-04 |
| Aspergillus_restrictus | <0.0001(<0.0001,0.0002) | 9.74E-04 | 4.70E-04 |
| Aspergillus_spe_Incertae_sedis | 0.0002(<0.0001,0.0013) | 0.001202818 | 3.93E-04 |
| Tuber_alcaracense | <0.0001(<0.0001,<0.0001) | 1.31E-04 | 1.12E-04 |
| Cladosporium_cladosporioides | <0.0001(<0.0001,0.0007) | 6.95E-04 | 2.58E-04 |
| Diaporthe_spe_Incertae_sedis | <0.0001(<0.0001,<0.0001) | 9.96E-05 | 6.92E-05 |
| Cladosporium_sphaerospermum | <0.0001(<0.0001,<0.0001) | 8.41E-04 | 5.73E-04 |
| Malasseziaceae_spe_Incertae_sedis | <0.0001(<0.0001,0.0002) | 0.001198682 | 6.39E-04 |
| Botrytis_cinerea | <0.0001(<0.0001,<0.0001) | 1.40E-04 | 1.38E-04 |
| Botryosphaeria_spe_Incertae_sedis | 0.0032(<0.0001,0.0135) | 0.011873045 | 0.005366906 |
| Mucor_circinelloides | 0.0052(0.0005,0.0121) | 0.012376273 | 0.003619451 |
| Calosphaeria_pulchella | <0.0001(<0.0001,<0.0001) | 1.85E-05 | 1.18E-05 |
| Bandonia_marina | <0.0001(<0.0001,<0.0001) | 4.56E-05 | 2.96E-05 |
| Talaromyces_funiculosus | <0.0001(<0.0001,<0.0001) | 0 | 0 |
| Chrysosphaeria_jan-nelii | <0.0001(<0.0001,<0.0001) | 0 | 0 |
| Dioszegia_zsoltii_var._yunnanensis | <0.0001(<0.0001,<0.0001) | 0.007924636 | 0.006992248 |
| Togniniella_acerosa | <0.0001(<0.0001,<0.0001) | 0 | 0 |
| Nigrospora_oryzae | <0.0001(<0.0001,0.0011) | 0.002627318 | 0.001519859 |
| Aspergillus_versicolor | 0.0002(<0.0001,0.0013) | 0.002857455 | 0.001439429 |
| Bjerkandera_mikrofumosa | <0.0001(<0.0001,<0.0001) | 0 | 0 |
| Kodamaea_ohmeri | <0.0001(<0.0001,<0.0001) | 0 | 0 |
| Saccharomyces_cerevisiae | <0.0001(<0.0001,<0.0001) | 7.54E-04 | 6.60E-04 |
| Kazachstania_heterogenica | <0.0001(<0.0001,0.0041) | 0.003499273 | 0.001484984 |
| Angustimassarina_camporesii | <0.0001(<0.0001,0.0003) | 3.89E-04 | 1.66E-04 |
| Rhexothecium_globosum | <0.0001(<0.0001,<0.0001) | 3.95E-06 | 3.95E-06 |
| Sporobolomyces_spe_Incertae_sedis | <0.0001(<0.0001,0.0001) | 0.006369 | 0.003682758 |
| Bovista_psammophila | <0.0001(<0.0001,<0.0001) | 0 | 0 |
| Zygotorulaspora_danielsina | <0.0001(<0.0001,<0.0001) | 3.45E-05 | 2.15E-05 |
| Pleosporales_spe_Incertae_sedis | <0.0001(<0.0001,<0.0001) | 1.31E-04 | 1.27E-04 |
| Coprinellus_spe_Incertae_sedis | <0.0001(<0.0001,<0.0001) | 0 | 0 |
| Cystofilobasidium_macerans | <0.0001(<0.0001,<0.0001) | 0 | 0 |
| Tausonia_pullulans | <0.0001(<0.0001,<0.0001) | 6.95E-05 | 6.37E-05 |
| Ophiostoma_acarorum | <0.0001(<0.0001,<0.0001) | 4.68E-06 | 4.68E-06 |
| Candida_zeylanoides | <0.0001(<0.0001,<0.0001) | 4.06E-04 | 3.20E-04 |
| Amylostereum_orientale | <0.0001(<0.0001,<0.0001) | 0 | 0 |
| Neopestalotiopsis_foedans | <0.0001(<0.0001,<0.0001) | 0 | 0 |
| Diaporthales_spe_Incertae_sedis | <0.0001(<0.0001,<0.0001) | 0 | 0 |
| Gibberella_fujikuroi | <0.0001(<0.0001,0.0025) | 0.002459773 | 0.001095237 |
| Tremellomycetes_spe_Incertae_sedis | <0.0001(<0.0001,<0.0001) | 9.06E-05 | 9.06E-05 |
| Fantasmomyces_hyalinus | <0.0001(<0.0001,<0.0001) | 1.48E-05 | 9.25E-06 |
| Pleurotus_eryngii | <0.0001(<0.0001,<0.0001) | 0 | 0 |
| Phanerochaete_chrysosporium | <0.0001(<0.0001,<0.0001) | 2.39E-04 | 2.39E-04 |
| Apiotrichum_domesticum | <0.0001(<0.0001,<0.0001) | 0 | 0 |
| Cladosporium_coloradense | <0.0001(<0.0001,<0.0001) | 3.70E-05 | 3.70E-05 |
| Leptosporella_bambusae | <0.0001(<0.0001,<0.0001) | 0.003747909 | 0.003730819 |
| Phyllachora_cynodonticola | <0.0001(<0.0001,0.0034) | 0.0022135 | 7.47E-04 |
| Capnodiales_spe_Incertae_sedis | <0.0001(<0.0001,<0.0001) | 0 | 0 |
| Ustilaginoidea_virens | <0.0001(<0.0001,0.0054) | 0.003362 | 0.001253369 |
| Malassezia_slooffiae | <0.0001(<0.0001,<0.0001) | 0 | 0 |
| Peroneutypa_scoparia | <0.0001(<0.0001,<0.0001) | 7.64E-06 | 5.34E-06 |
| Naganishia_globosa | <0.0001(<0.0001,<0.0001) | 0 | 0 |
| Peniophora_pseudoversicolor | <0.0001(<0.0001,<0.0001) | 0.002207227 | 0.002108651 |
| Talaromyces_spe_Incertae_sedis | <0.0001(<0.0001,<0.0001) | 0 | 0 |
| Coprinopsis_urticicola | <0.0001(<0.0001,<0.0001) | 0 | 0 |
| Typhula_micans | <0.0001(<0.0001,<0.0001) | 0 | 0 |
| Thielaviopsis_euricoi | <0.0001(<0.0001,<0.0001) | 0 | 0 |
| Flavodon_spe_Incertae_sedis | <0.0001(<0.0001,<0.0001) | 0 | 0 |
| Yarrowia_spe_Incertae_sedis | <0.0001(<0.0001,<0.0001) | 0 | 0 |
| Dipodascus_geotrichum | <0.0001(<0.0001,0.0003) | 9.30E-04 | 5.71E-04 |
| Cutaneotrichosporon_curvatus | <0.0001(<0.0001,<0.0001) | 0 | 0 |
| Exophiala_halophila | <0.0001(<0.0001,<0.0001) | 0 | 0 |
| Novakomyces_olei | <0.0001(<0.0001,<0.0001) | 0.001358818 | 9.47E-04 |
| Trichophaea_saccata | <0.0001(<0.0001,<0.0001) | 0.001505727 | 8.14E-04 |
| Tilletiopsis_washingtonensis | <0.0001(<0.0001,<0.0001) | 0.001708364 | 0.00137089 |
| Auricularia_auricula-judae | <0.0001(<0.0001,<0.0001) | 0 | 0 |
| Diutina_sipiczkii | <0.0001(<0.0001,<0.0001) | 0 | 0 |
| Keissleriella_quadriseptata | <0.0001(<0.0001,<0.0001) | 0 | 0 |
| Candida_inconspicua | <0.0001(<0.0001,0.0003) | 0.001525773 | 9.79E-04 |
| Zoophthora_radicans | <0.0001(<0.0001,0.0002) | 2.48E-04 | 1.17E-04 |
| Ceriporia_lacerata | <0.0001(<0.0001,<0.0001) | 0 | 0 |
| Vishniacozyma_spe_Incertae_sedis | <0.0001(<0.0001,<0.0001) | 1.58E-04 | 1.58E-04 |
| Cercospora_canescens | <0.0001(<0.0001,<0.0001) | 1.04E-04 | 1.02E-04 |
| Exidia_pithya | <0.0001(<0.0001,<0.0001) | 0.001615818 | 0.001614057 |
| Simplicillium_spe_Incertae_sedis | <0.0001(<0.0001,<0.0001) | 0.001587091 | 0.001584759 |
| Neodevriesia_lagerstroemiae | <0.0001(<0.0001,<0.0001) | 0 | 0 |
| Peniophorella_spe_Incertae_sedis | <0.0001(<0.0001,<0.0001) | 0 | 0 |
| Acrocalymma_fici | <0.0001(<0.0001,<0.0001) | 0 | 0 |
| Aspergillus_coremiiformis | <0.0001(<0.0001,<0.0001) | 0 | 0 |
| Onygenales_spe_Incertae_sedis | <0.0001(<0.0001,<0.0001) | 0.001196545 | 0.001194547 |
| Diaporthe_caulivora | <0.0001(<0.0001,<0.0001) | 0 | 0 |
| Hypoxylon_perforatum | <0.0001(<0.0001,<0.0001) | 0 | 0 |
| Zasmidium_fructigenum | <0.0001(<0.0001,<0.0001) | 0 | 0 |
| Tilletia_spe_Incertae_sedis | <0.0001(<0.0001,<0.0001) | 0 | 0 |
| Ciliophora_spe_Incertae_sedis | <0.0001(<0.0001,<0.0001) | 1.09E-05 | 9.33E-06 |
| Yarrowia_bubula | <0.0001(<0.0001,<0.0001) | 0 | 0 |
| Peniophora_cinerea | <0.0001(<0.0001,<0.0001) | 0 | 0 |
| Agaricales_spe_Incertae_sedis | <0.0001(<0.0001,<0.0001) | 5.48E-04 | 5.43E-04 |
| [Cryptococcus]_ater | <0.0001(<0.0001,<0.0001) | 0 | 0 |
| Rosellinia_australiensis | <0.0001(<0.0001,<0.0001) | 5.37E-04 | 4.58E-04 |

| Data S5. Comparison of oral mycobiome at the species level between three groups | | | |
| --- | --- | --- | --- |
| ID | CPR1.median | CPR1.mean | CPR1.se |
| Zanclospora_jonesii | 0.012(0.0005,0.0254) | 0.081040185 | 0.039145513 |
| Acrodictys_fluminicola | 0.0037(0.0006,0.0769) | 0.064523074 | 0.019575732 |
| Aspergillus_subflavus | 0.0016(0.0003,0.0375) | 0.043961407 | 0.015671467 |
| Fungi_spe_Incertae_sedis | 0.104(0.0474,0.2228) | 0.145799037 | 0.025381007 |
| Actinomucor_elegans | <0.0001(<0.0001,0.0004) | 9.82E-04 | 4.45E-04 |
| Entorrhiza_citriformis | 0.0003(<0.0001,0.0555) | 0.048043222 | 0.022798066 |
| Malassezia_restricta | 0.0839(0.0128,0.1516) | 0.123201 | 0.02892802 |
| Blumeria_spe_Incertae_sedis | <0.0001(<0.0001,<0.0001) | 8.24E-04 | 4.64E-04 |
| Cryptococcus_longus | <0.0001(<0.0001,<0.0001) | 2.96E-06 | 2.96E-06 |
| Raffaelea_lauricola | <0.0001(<0.0001,<0.0001) | 2.35E-04 | 2.35E-04 |
| Diversispora_spurca | <0.0001(<0.0001,<0.0001) | 4.16E-04 | 2.92E-04 |
| Arthrographis_grakistii | <0.0001(<0.0001,<0.0001) | 5.89E-06 | 5.89E-06 |
| Heitmania_litseae | <0.0001(<0.0001,0.0005) | 0.009904259 | 0.005998678 |
| Ascomycota_spe_Incertae_sedis | <0.0001(<0.0001,0.0002) | 0.005749741 | 0.003090452 |
| Milospium_graphideorum | <0.0001(<0.0001,<0.0001) | 0.011125333 | 0.008493331 |
| Aspergillus_penicillioides | <0.0001(<0.0001,0.0146) | 0.033978481 | 0.014914548 |
| Candida_albicans | <0.0001(<0.0001,0.0004) | 0.00266763 | 0.001776717 |
| Alternaria_alternata | <0.0001(<0.0001,<0.0001) | 0.003788148 | 0.002642726 |
| Talaromyces_scorteus | <0.0001(<0.0001,<0.0001) | 0.001351815 | 6.17E-04 |
| Candida_tropicalis | <0.0001(<0.0001,0.0009) | 0.039011778 | 0.036814205 |
| Udeniomyces_megalosporus | <0.0001(<0.0001,<0.0001) | 0.002912593 | 0.002877358 |
| Dipodascaceae_spe_Incertae_sedis | <0.0001(<0.0001,<0.0001) | 0.019001556 | 0.016415285 |
| Cladosporium_halotolerans | <0.0001(<0.0001,<0.0001) | 0.002478778 | 0.001547243 |
| Saprochaete_gigas | <0.0001(<0.0001,<0.0001) | 0 | 0 |
| Rhodotorula_mucilaginosa | <0.0001(<0.0001,<0.0001) | 0.001746296 | 0.001240976 |
| Debaryomyces_prosopidis | <0.0001(<0.0001,<0.0001) | 1.78E-06 | 1.78E-06 |
| Parafuscosporella_mucosa | <0.0001(<0.0001,0.0024) | 0.002120815 | 8.77E-04 |
| Dothiora_spartii | <0.0001(<0.0001,0.0036) | 0.005466556 | 0.002338519 |
| Udeniomyces_pyricola | <0.0001(<0.0001,<0.0001) | 2.30E-05 | 1.65E-05 |
| Aspergillus_amstelodami | <0.0001(<0.0001,<0.0001) | 0.006308444 | 0.003750043 |
| Candida_parapsilosis | <0.0001(<0.0001,<0.0001) | 0.004429778 | 0.002896172 |
| Simplicillium_sympodiophorum | 0.0003(<0.0001,0.0018) | 0.004319111 | 0.002567903 |
| Wallemia_spe_Incertae_sedis | <0.0001(<0.0001,<0.0001) | 5.53E-04 | 5.53E-04 |
| Phaeosphaeria_oryzae | <0.0001(<0.0001,<0.0001) | 7.95E-04 | 7.95E-04 |
| Aureobasidium_pullulans | <0.0001(<0.0001,0.0001) | 0.010784593 | 0.010682964 |
| Candida_spe_Incertae_sedis | <0.0001(<0.0001,<0.0001) | 0.01024263 | 0.004661782 |
| Aspergillus_restrictus | <0.0001(<0.0001,0.0031) | 0.011567593 | 0.006617147 |
| Aspergillus_spe_Incertae_sedis | <0.0001(<0.0001,0.0008) | 0.006669556 | 0.003355974 |
| Tuber_alcaracense | <0.0001(<0.0001,0.0003) | 2.14E-04 | 9.11E-05 |
| Cladosporium_cladosporioides | <0.0001(<0.0001,0.003) | 0.002223259 | 6.61E-04 |
| Diaporthe_spe_Incertae_sedis | <0.0001(<0.0001,<0.0001) | 0 | 0 |
| Cladosporium_sphaerospermum | <0.0001(<0.0001,<0.0001) | 0.002679704 | 0.002032167 |
| Malasseziaceae_spe_Incertae_sedis | <0.0001(<0.0001,<0.0001) | 0.004315074 | 0.004315074 |
| Botrytis_cinerea | <0.0001(<0.0001,<0.0001) | 0.007715667 | 0.005273289 |
| Botryosphaeria_spe_Incertae_sedis | <0.0001(<0.0001,<0.0001) | 6.76E-04 | 6.26E-04 |
| Mucor_circinelloides | <0.0001(<0.0001,<0.0001) | 3.86E-04 | 3.68E-04 |
| Calosphaeria_pulchella | <0.0001(<0.0001,0.0003) | 5.93E-04 | 3.35E-04 |
| Bandonia_marina | <0.0001(<0.0001,0.0002) | 0.007030222 | 0.004637031 |
| Talaromyces_funiculosus | <0.0001(<0.0001,<0.0001) | 0 | 0 |
| Chrysosphaeria_jan-nelii | <0.0001(<0.0001,<0.0001) | 1.80E-04 | 1.53E-04 |
| Dioszegia_zsoltii_var._yunnanensis | <0.0001(<0.0001,<0.0001) | 1.04E-05 | 5.71E-06 |
| Togniniella_acerosa | <0.0001(<0.0001,<0.0001) | 0 | 0 |
| Nigrospora_oryzae | <0.0001(<0.0001,<0.0001) | 0.002271852 | 0.002226886 |
| Aspergillus_versicolor | <0.0001(<0.0001,<0.0001) | 0.001253444 | 7.89E-04 |
| Bjerkandera_mikrofumosa | <0.0001(<0.0001,<0.0001) | 0 | 0 |
| Kodamaea_ohmeri | <0.0001(<0.0001,<0.0001) | 0.006127519 | 0.004273324 |
| Saccharomyces_cerevisiae | <0.0001(<0.0001,<0.0001) | 1.50E-05 | 9.37E-06 |
| Kazachstania_heterogenica | <0.0001(<0.0001,<0.0001) | 6.43E-04 | 6.11E-04 |
| Angustimassarina_camporesii | <0.0001(<0.0001,<0.0001) | 4.62E-04 | 2.68E-04 |
| Rhexothecium_globosum | <0.0001(<0.0001,<0.0001) | 0 | 0 |
| Sporobolomyces_spe_Incertae_sedis | <0.0001(<0.0001,<0.0001) | 1.19E-06 | 1.19E-06 |
| Bovista_psammophila | <0.0001(<0.0001,<0.0001) | 3.10E-05 | 1.59E-05 |
| Zygotorulaspora_danielsina | <0.0001(<0.0001,0.0002) | 5.23E-04 | 3.89E-04 |
| Pleosporales_spe_Incertae_sedis | <0.0001(<0.0001,<0.0001) | 0 | 0 |
| Coprinellus_spe_Incertae_sedis | <0.0001(<0.0001,<0.0001) | 0 | 0 |
| Cystofilobasidium_macerans | <0.0001(<0.0001,<0.0001) | 0 | 0 |
| Tausonia_pullulans | <0.0001(<0.0001,<0.0001) | 0.001839444 | 0.001839444 |
| Ophiostoma_acarorum | <0.0001(<0.0001,<0.0001) | 1.41E-05 | 1.41E-05 |
| Candida_zeylanoides | <0.0001(<0.0001,<0.0001) | 0.002696148 | 0.002696148 |
| Amylostereum_orientale | <0.0001(<0.0001,<0.0001) | 0.004501667 | 0.004486367 |
| Neopestalotiopsis_foedans | <0.0001(<0.0001,<0.0001) | 0.004455 | 0.004453077 |
| Diaporthales_spe_Incertae_sedis | <0.0001(<0.0001,<0.0001) | 0 | 0 |
| Gibberella_fujikuroi | <0.0001(<0.0001,<0.0001) | 0.002183444 | 0.001763508 |
| Tremellomycetes_spe_Incertae_sedis | <0.0001(<0.0001,<0.0001) | 0 | 0 |
| Fantasmomyces_hyalinus | <0.0001(<0.0001,0.0002) | 6.53E-04 | 4.12E-04 |
| Pleurotus_eryngii | <0.0001(<0.0001,<0.0001) | 0 | 0 |
| Phanerochaete_chrysosporium | <0.0001(<0.0001,<0.0001) | 0.003604407 | 0.003595489 |
| Apiotrichum_domesticum | <0.0001(<0.0001,<0.0001) | 0 | 0 |
| Cladosporium_coloradense | <0.0001(<0.0001,<0.0001) | 3.23E-04 | 2.57E-04 |
| Leptosporella_bambusae | <0.0001(<0.0001,<0.0001) | 5.49E-05 | 5.15E-05 |
| Phyllachora_cynodonticola | <0.0001(<0.0001,<0.0001) | 0 | 0 |
| Capnodiales_spe_Incertae_sedis | <0.0001(<0.0001,<0.0001) | 0 | 0 |
| Ustilaginoidea_virens | <0.0001(<0.0001,<0.0001) | 0 | 0 |
| Malassezia_slooffiae | <0.0001(<0.0001,<0.0001) | 0.00274963 | 0.001937114 |
| Peroneutypa_scoparia | <0.0001(<0.0001,<0.0001) | 0.002494667 | 0.002493129 |
| Naganishia_globosa | <0.0001(<0.0001,<0.0001) | 0 | 0 |
| Peniophora_pseudoversicolor | <0.0001(<0.0001,<0.0001) | 0 | 0 |
| Talaromyces_spe_Incertae_sedis | <0.0001(<0.0001,<0.0001) | 0.002385296 | 0.002064921 |
| Coprinopsis_urticicola | <0.0001(<0.0001,<0.0001) | 0 | 0 |
| Typhula_micans | <0.0001(<0.0001,<0.0001) | 0 | 0 |
| Thielaviopsis_euricoi | <0.0001(<0.0001,<0.0001) | 1.36E-04 | 1.36E-04 |
| Flavodon_spe_Incertae_sedis | <0.0001(<0.0001,<0.0001) | 0 | 0 |
| Yarrowia_spe_Incertae_sedis | <0.0001(<0.0001,<0.0001) | 4.43E-04 | 4.43E-04 |
| Dipodascus_geotrichum | <0.0001(<0.0001,<0.0001) | 7.11E-05 | 6.93E-05 |
| Cutaneotrichosporon_curvatus | <0.0001(<0.0001,<0.0001) | 0.001767926 | 0.001761744 |
| Exophiala_halophila | <0.0001(<0.0001,<0.0001) | 0.001742148 | 0.001736426 |
| Novakomyces_olei | <0.0001(<0.0001,<0.0001) | 6.93E-06 | 5.27E-06 |
| Trichophaea_saccata | <0.0001(<0.0001,<0.0001) | 4.00E-04 | 3.97E-04 |
| Tilletiopsis_washingtonensis | <0.0001(<0.0001,<0.0001) | 0 | 0 |
| Auricularia_auricula-judae | <0.0001(<0.0001,<0.0001) | 0 | 0 |
| Diutina_sipiczkii | <0.0001(<0.0001,<0.0001) | 6.63E-06 | 5.17E-06 |
| Keissleriella_quadriseptata | <0.0001(<0.0001,<0.0001) | 0 | 0 |
| Candida_inconspicua | <0.0001(<0.0001,<0.0001) | 0 | 0 |
| Zoophthora_radicans | <0.0001(<0.0001,<0.0001) | 0 | 0 |
| Ceriporia_lacerata | <0.0001(<0.0001,<0.0001) | 0 | 0 |
| Vishniacozyma_spe_Incertae_sedis | <0.0001(<0.0001,<0.0001) | 0 | 0 |
| Cercospora_canescens | <0.0001(<0.0001,<0.0001) | 0 | 0 |
| Exidia_pithya | <0.0001(<0.0001,<0.0001) | 0 | 0 |
| Simplicillium_spe_Incertae_sedis | <0.0001(<0.0001,<0.0001) | 8.26E-06 | 8.26E-06 |
| Neodevriesia_lagerstroemiae | <0.0001(<0.0001,<0.0001) | 0.001271593 | 0.001057834 |
| Peniophorella_spe_Incertae_sedis | <0.0001(<0.0001,<0.0001) | 0 | 0 |
| Acrocalymma_fici | <0.0001(<0.0001,<0.0001) | 0 | 0 |
| Aspergillus_coremiiformis | <0.0001(<0.0001,<0.0001) | 0 | 0 |
| Onygenales_spe_Incertae_sedis | <0.0001(<0.0001,<0.0001) | 0 | 0 |
| Diaporthe_caulivora | <0.0001(<0.0001,<0.0001) | 0 | 0 |
| Hypoxylon_perforatum | <0.0001(<0.0001,<0.0001) | 0 | 0 |
| Zasmidium_fructigenum | <0.0001(<0.0001,<0.0001) | 8.40E-04 | 5.95E-04 |
| Tilletia_spe_Incertae_sedis | <0.0001(<0.0001,<0.0001) | 0 | 0 |
| Ciliophora_spe_Incertae_sedis | <0.0001(<0.0001,<0.0001) | 0 | 0 |
| Yarrowia_bubula | <0.0001(<0.0001,<0.0001) | 0 | 0 |
| Peniophora_cinerea | <0.0001(<0.0001,<0.0001) | 0 | 0 |
| Agaricales_spe_Incertae_sedis | <0.0001(<0.0001,<0.0001) | 4.81E-06 | 4.81E-06 |
| [Cryptococcus]_ater | <0.0001(<0.0001,<0.0001) | 0 | 0 |
| Rosellinia_australiensis | <0.0001(<0.0001,<0.0001) | 0 | 0 |

| Data S5. Comparison of oral mycobiome at the species level between three groups | | | |
| --- | --- | --- | --- |
| ID | HC.median | HC.mean | HC.se |
| Zanclospora_jonesii | 0.0754(0.0199,0.3216) | 0.210876989 | 0.026794968 |
| Acrodictys_fluminicola | 0.0323(0.0066,0.1606) | 0.101940244 | 0.01409439 |
| Aspergillus_subflavus | 0.0124(0.0028,0.0971) | 0.093119233 | 0.018038496 |
| Fungi_spe_Incertae_sedis | 0.0428(0.0195,0.063) | 0.055815311 | 0.007132234 |
| Actinomucor_elegans | <0.0001(<0.0001,<0.0001) | 2.65E-05 | 6.60E-06 |
| Entorrhiza_citriformis | 0.0033(0.0016,0.0375) | 0.057662778 | 0.01154618 |
| Malassezia_restricta | 0.0055(0.0009,0.0206) | 0.025656122 | 0.006822908 |
| Blumeria_spe_Incertae_sedis | 0.0015(0.0007,0.0063) | 0.045594189 | 0.013059388 |
| Cryptococcus_longus | 0.0093(0.0016,0.0391) | 0.036513811 | 0.006314694 |
| Raffaelea_lauricola | 0.0004(<0.0001,0.0011) | 0.030690811 | 0.013637806 |
| Diversispora_spurca | 0.0013(0.0003,0.0072) | 0.030039222 | 0.012607578 |
| Arthrographis_grakistii | 0.0003(<0.0001,0.0008) | 0.022962267 | 0.012772478 |
| Heitmania_litseae | 0.0008(0.0003,0.0023) | 0.017578344 | 0.004917585 |
| Ascomycota_spe_Incertae_sedis | 0.0018(0.0006,0.0093) | 0.014598311 | 0.00361466 |
| Milospium_graphideorum | <0.0001(<0.0001,0.0007) | 0.015415089 | 0.009830505 |
| Aspergillus_penicillioides | <0.0001(<0.0001,0.0005) | 0.003030567 | 0.001286047 |
| Candida_albicans | <0.0001(<0.0001,0.0002) | 1.45E-04 | 3.64E-05 |
| Alternaria_alternata | 0.0006(0.0001,0.0047) | 0.0109662 | 0.003991309 |
| Talaromyces_scorteus | 0.0001(<0.0001,0.0002) | 0.011110522 | 0.004211647 |
| Candida_tropicalis | <0.0001(<0.0001,<0.0001) | 0.001913378 | 0.001869266 |
| Udeniomyces_megalosporus | <0.0001(<0.0001,<0.0001) | 0.003243222 | 0.002737524 |
| Dipodascaceae_spe_Incertae_sedis | <0.0001(<0.0001,0.0008) | 5.36E-04 | 1.03E-04 |
| Cladosporium_halotolerans | 0.0002(<0.0001,0.0015) | 0.007325122 | 0.001853324 |
| Saprochaete_gigas | <0.0001(<0.0001,<0.0001) | 0.008890656 | 0.008636547 |
| Rhodotorula_mucilaginosa | <0.0001(<0.0001,<0.0001) | 2.03E-04 | 6.87E-05 |
| Debaryomyces_prosopidis | <0.0001(<0.0001,0.0001) | 6.51E-04 | 3.85E-04 |
| Parafuscosporella_mucosa | 0.0023(0.0003,0.0131) | 0.007147167 | 9.49E-04 |
| Dothiora_spartii | 0.0003(0.0001,0.0036) | 0.005962189 | 0.001469701 |
| Udeniomyces_pyricola | <0.0001(<0.0001,<0.0001) | 0 | 0 |
| Aspergillus_amstelodami | 0.0001(<0.0001,0.0003) | 0.004219267 | 0.001709655 |
| Candida_parapsilosis | <0.0001(<0.0001,0.0001) | 0.004690056 | 0.001932289 |
| Simplicillium_sympodiophorum | 0.0003(0.0001,0.0025) | 0.004307056 | 9.34E-04 |
| Wallemia_spe_Incertae_sedis | <0.0001(<0.0001,0.0002) | 0.005011122 | 0.003898453 |
| Phaeosphaeria_oryzae | <0.0001(<0.0001,0.0003) | 0.0049226 | 0.003282017 |
| Aureobasidium_pullulans | <0.0001(<0.0001,<0.0001) | 5.53E-04 | 4.47E-04 |
| Candida_spe_Incertae_sedis | <0.0001(<0.0001,0.0002) | 0.001284056 | 7.18E-04 |
| Aspergillus_restrictus | <0.0001(<0.0001,<0.0001) | 3.46E-04 | 2.02E-04 |
| Aspergillus_spe_Incertae_sedis | <0.0001(<0.0001,<0.0001) | 0.001605456 | 5.98E-04 |
| Tuber_alcaracense | 0.0001(<0.0001,0.0007) | 0.003741267 | 0.00193903 |
| Cladosporium_cladosporioides | 0.0004(<0.0001,0.0022) | 0.002994311 | 8.82E-04 |
| Diaporthe_spe_Incertae_sedis | <0.0001(<0.0001,0.0001) | 0.003761822 | 0.003145895 |
| Cladosporium_sphaerospermum | <0.0001(<0.0001,<0.0001) | 0.002683211 | 9.90E-04 |
| Malasseziaceae_spe_Incertae_sedis | <0.0001(<0.0001,0.0002) | 0.002103222 | 6.63E-04 |
| Botrytis_cinerea | <0.0001(<0.0001,<0.0001) | 0.001182278 | 7.22E-04 |
| Botryosphaeria_spe_Incertae_sedis | <0.0001(<0.0001,<0.0001) | 3.16E-04 | 3.02E-04 |
| Mucor_circinelloides | <0.0001(<0.0001,<0.0001) | 8.85E-05 | 2.13E-05 |
| Calosphaeria_pulchella | 0.0001(<0.0001,0.0017) | 0.0023836 | 4.96E-04 |
| Bandonia_marina | <0.0001(<0.0001,<0.0001) | 3.84E-04 | 3.28E-04 |
| Talaromyces_funiculosus | <0.0001(<0.0001,<0.0001) | 0.002152444 | 0.001631295 |
| Chrysosphaeria_jan-nelii | <0.0001(<0.0001,0.0002) | 0.002012467 | 0.001286314 |
| Dioszegia_zsoltii_var._yunnanensis | <0.0001(<0.0001,<0.0001) | 1.05E-04 | 8.60E-05 |
| Togniniella_acerosa | <0.0001(<0.0001,<0.0001) | 0.0020321 | 0.001177724 |
| Nigrospora_oryzae | <0.0001(<0.0001,<0.0001) | 6.75E-04 | 4.83E-04 |
| Aspergillus_versicolor | <0.0001(<0.0001,<0.0001) | 7.86E-04 | 2.44E-04 |
| Bjerkandera_mikrofumosa | <0.0001(<0.0001,<0.0001) | 0.001853256 | 0.001803947 |
| Kodamaea_ohmeri | <0.0001(<0.0001,<0.0001) | 0 | 0 |
| Saccharomyces_cerevisiae | <0.0001(<0.0001,0.0003) | 0.001554967 | 6.08E-04 |
| Kazachstania_heterogenica | <0.0001(<0.0001,<0.0001) | 6.88E-04 | 5.17E-04 |
| Angustimassarina_camporesii | <0.0001(<0.0001,0.001) | 0.001486744 | 3.89E-04 |
| Rhexothecium_globosum | <0.0001(<0.0001,0.0002) | 0.001690344 | 0.001124441 |
| Sporobolomyces_spe_Incertae_sedis | <0.0001(<0.0001,<0.0001) | 8.11E-05 | 7.99E-05 |
| Bovista_psammophila | <0.0001(<0.0001,0.0004) | 0.001593456 | 7.96E-04 |
| Zygotorulaspora_danielsina | <0.0001(<0.0001,0.0002) | 0.001421178 | 8.06E-04 |
| Pleosporales_spe_Incertae_sedis | <0.0001(<0.0001,<0.0001) | 0.0015339 | 0.001054156 |
| Coprinellus_spe_Incertae_sedis | <0.0001(<0.0001,<0.0001) | 0.001527922 | 9.05E-04 |
| Cystofilobasidium_macerans | <0.0001(<0.0001,<0.0001) | 0.001497833 | 0.00112603 |
| Tausonia_pullulans | <0.0001(<0.0001,<0.0001) | 9.16E-04 | 5.47E-04 |
| Ophiostoma_acarorum | <0.0001(<0.0001,0.0002) | 0.0014574 | 7.01E-04 |
| Candida_zeylanoides | <0.0001(<0.0001,<0.0001) | 5.00E-04 | 3.24E-04 |
| Amylostereum_orientale | <0.0001(<0.0001,<0.0001) | 0 | 0 |
| Neopestalotiopsis_foedans | <0.0001(<0.0001,<0.0001) | 0 | 0 |
| Diaporthales_spe_Incertae_sedis | <0.0001(<0.0001,<0.0001) | 0.001322878 | 9.79E-04 |
| Gibberella_fujikuroi | <0.0001(<0.0001,<0.0001) | 1.51E-05 | 6.94E-06 |
| Tremellomycetes_spe_Incertae_sedis | <0.0001(<0.0001,<0.0001) | 0.001179233 | 0.001160052 |
| Fantasmomyces_hyalinus | <0.0001(<0.0001,0.0005) | 9.68E-04 | 3.05E-04 |
| Pleurotus_eryngii | <0.0001(<0.0001,<0.0001) | 0.001147778 | 8.42E-04 |
| Phanerochaete_chrysosporium | <0.0001(<0.0001,<0.0001) | 0 | 0 |
| Apiotrichum_domesticum | <0.0001(<0.0001,0.0001) | 0.001091722 | 5.70E-04 |
| Cladosporium_coloradense | <0.0001(<0.0001,<0.0001) | 8.96E-04 | 7.69E-04 |
| Leptosporella_bambusae | <0.0001(<0.0001,<0.0001) | 3.53E-06 | 3.53E-06 |
| Phyllachora_cynodonticola | <0.0001(<0.0001,<0.0001) | 3.33E-04 | 1.76E-04 |
| Capnodiales_spe_Incertae_sedis | <0.0001(<0.0001,<0.0001) | 8.48E-04 | 7.09E-04 |
| Ustilaginoidea_virens | <0.0001(<0.0001,<0.0001) | 1.43E-05 | 5.25E-06 |
| Malassezia_slooffiae | <0.0001(<0.0001,<0.0001) | 0 | 0 |
| Peroneutypa_scoparia | <0.0001(<0.0001,<0.0001) | 0 | 0 |
| Naganishia_globosa | <0.0001(<0.0001,<0.0001) | 7.44E-04 | 6.35E-04 |
| Peniophora_pseudoversicolor | <0.0001(<0.0001,<0.0001) | 1.80E-04 | 1.78E-04 |
| Talaromyces_spe_Incertae_sedis | <0.0001(<0.0001,<0.0001) | 0 | 0 |
| Coprinopsis_urticicola | <0.0001(<0.0001,<0.0001) | 6.51E-04 | 4.48E-04 |
| Typhula_micans | <0.0001(<0.0001,<0.0001) | 5.93E-04 | 5.08E-04 |
| Thielaviopsis_euricoi | <0.0001(<0.0001,<0.0001) | 5.47E-04 | 3.46E-04 |
| Flavodon_spe_Incertae_sedis | <0.0001(<0.0001,<0.0001) | 5.84E-04 | 5.41E-04 |
| Yarrowia_spe_Incertae_sedis | <0.0001(<0.0001,<0.0001) | 4.28E-04 | 3.25E-04 |
| Dipodascus_geotrichum | <0.0001(<0.0001,0.0004) | 3.12E-04 | 6.47E-05 |
| Cutaneotrichosporon_curvatus | <0.0001(<0.0001,<0.0001) | 0 | 0 |
| Exophiala_halophila | <0.0001(<0.0001,<0.0001) | 0 | 0 |
| Novakomyces_olei | <0.0001(<0.0001,<0.0001) | 1.78E-04 | 1.51E-04 |
| Trichophaea_saccata | <0.0001(<0.0001,<0.0001) | 1.26E-05 | 1.04E-05 |
| Tilletiopsis_washingtonensis | <0.0001(<0.0001,<0.0001) | 7.91E-05 | 6.66E-05 |
| Auricularia_auricula-judae | <0.0001(<0.0001,<0.0001) | 4.84E-04 | 3.79E-04 |
| Diutina_sipiczkii | <0.0001(<0.0001,<0.0001) | 4.61E-04 | 2.04E-04 |
| Keissleriella_quadriseptata | <0.0001(<0.0001,<0.0001) | 4.52E-04 | 4.37E-04 |
| Candida_inconspicua | <0.0001(<0.0001,<0.0001) | 6.40E-05 | 1.39E-05 |
| Zoophthora_radicans | <0.0001(<0.0001,<0.0001) | 3.73E-04 | 1.60E-04 |
| Ceriporia_lacerata | <0.0001(<0.0001,<0.0001) | 4.18E-04 | 3.07E-04 |
| Vishniacozyma_spe_Incertae_sedis | <0.0001(<0.0001,<0.0001) | 3.78E-04 | 2.72E-04 |
| Cercospora_canescens | <0.0001(<0.0001,<0.0001) | 3.80E-04 | 1.79E-04 |
| Exidia_pithya | <0.0001(<0.0001,<0.0001) | 0 | 0 |
| Simplicillium_spe_Incertae_sedis | <0.0001(<0.0001,<0.0001) | 0 | 0 |
| Neodevriesia_lagerstroemiae | <0.0001(<0.0001,<0.0001) | 0 | 0 |
| Peniophorella_spe_Incertae_sedis | <0.0001(<0.0001,<0.0001) | 3.77E-04 | 3.60E-04 |
| Acrocalymma_fici | <0.0001(<0.0001,<0.0001) | 3.34E-04 | 3.20E-04 |
| Aspergillus_coremiiformis | <0.0001(<0.0001,<0.0001) | 3.33E-04 | 2.15E-04 |
| Onygenales_spe_Incertae_sedis | <0.0001(<0.0001,<0.0001) | 4.13E-06 | 4.13E-06 |
| Diaporthe_caulivora | <0.0001(<0.0001,<0.0001) | 2.86E-04 | 1.95E-04 |
| Hypoxylon_perforatum | <0.0001(<0.0001,<0.0001) | 2.74E-04 | 2.57E-04 |
| Zasmidium_fructigenum | <0.0001(<0.0001,<0.0001) | 0 | 0 |
| Tilletia_spe_Incertae_sedis | <0.0001(<0.0001,<0.0001) | 2.31E-04 | 1.52E-04 |
| Ciliophora_spe_Incertae_sedis | <0.0001(<0.0001,<0.0001) | 2.11E-04 | 1.37E-04 |
| Yarrowia_bubula | <0.0001(<0.0001,<0.0001) | 1.78E-04 | 1.67E-04 |
| Peniophora_cinerea | <0.0001(<0.0001,<0.0001) | 1.42E-04 | 1.30E-04 |
| Agaricales_spe_Incertae_sedis | <0.0001(<0.0001,<0.0001) | 0 | 0 |
| [Cryptococcus]_ater | <0.0001(<0.0001,<0.0001) | 1.34E-04 | 1.13E-04 |
| Rosellinia_australiensis | <0.0001(<0.0001,<0.0001) | 0 | 0 |

| Data S5. Comparison of oral mycobiome at the species level between three groups | | | | | |
| --- | --- | --- | --- | --- | --- |
| ID | p-value | z-score | Sig_mark | q-value | fixp |
| Zanclospora_jonesii | 5.17E-05 | -4.047806603 | *** | 5.76E-04 | <0.0001 |
| Acrodictys_fluminicola | 1.85E-06 | -4.769348696 | *** | 3.03E-05 | <0.0001 |
| Aspergillus_subflavus | 3.30E-07 | -5.105657947 | *** | 7.60E-06 | <0.0001 |
| Fungi_spe_Incertae_sedis | 1.54E-10 | -6.401030294 | *** | 7.70E-09 | <0.0001 |
| Actinomucor_elegans | 1.70E-17 | -8.51241211 | *** | 2.98E-15 | <0.0001 |
| Entorrhiza_citriformis | 8.26E-07 | -4.929033173 | *** | 1.49E-05 | <0.0001 |
| Malassezia_restricta | 5.36E-04 | -3.461964659 | *** | 0.00460645 | 0.0005 |
| Blumeria_spe_Incertae_sedis | 3.29E-16 | -8.162334406 | *** | 4.30E-14 | <0.0001 |
| Cryptococcus_longus | 3.96E-22 | -9.672250912 | *** | 2.07E-19 | <0.0001 |
| Raffaelea_lauricola | 2.32E-09 | -5.973907398 | *** | 9.34E-08 | <0.0001 |
| Diversispora_spurca | 1.62E-10 | -6.39387835 | *** | 7.70E-09 | <0.0001 |
| Arthrographis_grakistii | 6.93E-11 | -6.522109185 | *** | 4.04E-09 | <0.0001 |
| Heitmania_litseae | 2.22E-12 | -7.019625496 | *** | 1.67E-10 | <0.0001 |
| Ascomycota_spe_Incertae_sedis | 1.76E-06 | -4.779387147 | *** | 2.97E-05 | <0.0001 |
| Milospium_graphideorum | 1.06E-04 | -3.876677457 | *** | 0.0011324 | 0.0001 |
| Aspergillus_penicillioides | 1.00E-04 | -3.889853087 | *** | 0.001095 | 0.0001 |
| Candida_albicans | 5.47E-06 | -4.546029145 | *** | 8.18E-05 | <0.0001 |
| Alternaria_alternata | 3.49E-07 | -5.095005426 | *** | 7.60E-06 | <0.0001 |
| Talaromyces_scorteus | 3.96E-06 | -4.613363686 | *** | 6.11E-05 | <0.0001 |
| Candida_tropicalis | 0.00116733 | -3.246744066 | ** | 0.0094105 | 0.0012 |
| Udeniomyces_megalosporus | 2.39E-04 | -3.673794577 | *** | 0.00227678 | 0.0002 |
| Dipodascaceae_spe_Incertae_sedis | 3.63E-07 | -5.08759967 | *** | 7.60E-06 | <0.0001 |
| Cladosporium_halotolerans | 3.28E-05 | -4.153081772 | *** | 4.00E-04 | <0.0001 |
| Saprochaete_gigas | 0.01083739 | -2.547898967 | * | 0.05567441 | 0.0108 |
| Rhodotorula_mucilaginosa | 4.31E-13 | -7.245555349 | *** | 4.51E-11 | <0.0001 |
| Debaryomyces_prosopidis | 2.49E-04 | -3.662824316 | *** | 0.00233414 | 0.0002 |
| Parafuscosporella_mucosa | 1.70E-09 | -6.024046532 | *** | 7.43E-08 | <0.0001 |
| Dothiora_spartii | 2.78E-06 | -4.68633648 | *** | 4.42E-05 | <0.0001 |
| Udeniomyces_pyricola | 1.35E-12 | -7.088880531 | *** | 1.18E-10 | <0.0001 |
| Aspergillus_amstelodami | 3.01E-05 | -4.172554214 | *** | 3.76E-04 | <0.0001 |
| Candida_parapsilosis | 0.00102808 | -3.28272848 | ** | 0.00855098 | 0.001 |
| Simplicillium_sympodiophorum | 5.98E-07 | -4.991737899 | *** | 1.16E-05 | <0.0001 |
| Wallemia_spe_Incertae_sedis | 1.45E-04 | -3.798660019 | *** | 0.00152464 | 0.0001 |
| Phaeosphaeria_oryzae | 0.01228613 | -2.503817631 | * | 0.06190322 | 0.0123 |
| Aureobasidium_pullulans | 0.01348282 | -2.470755007 | * | 0.06728567 | 0.0135 |
| Candida_spe_Incertae_sedis | 0.02127492 | -2.303069763 | * | 0.08381998 | 0.0213 |
| Aspergillus_restrictus | 0.00304182 | -2.96348032 | ** | 0.02070014 | 0.003 |
| Aspergillus_spe_Incertae_sedis | 0.0345355 | -2.113763155 | * | 0.12394933 | 0.0345 |
| Tuber_alcaracense | 3.65E-05 | -4.128656168 | *** | 4.25E-04 | <0.0001 |
| Cladosporium_cladosporioides | 0.01936331 | -2.338461707 | * | 0.0818256 | 0.0194 |
| Diaporthe_spe_Incertae_sedis | 0.00203323 | -3.085335271 | ** | 0.0150058 | 0.002 |
| Cladosporium_sphaerospermum | 0.02888979 | -2.184987184 | * | 0.10557126 | 0.0289 |
| Malasseziaceae_spe_Incertae_sedis | 8.56E-06 | -4.450635611 | *** | 1.18E-04 | <0.0001 |
| Botrytis_cinerea | 0.01860107 | -2.353430714 | * | 0.08027005 | 0.0186 |
| Botryosphaeria_spe_Incertae_sedis | 4.19E-09 | -5.876374459 | *** | 1.57E-07 | <0.0001 |
| Mucor_circinelloides | 1.07E-11 | -6.797132091 | *** | 6.99E-10 | <0.0001 |
| Calosphaeria_pulchella | 6.61E-08 | -5.401474463 | *** | 1.92E-06 | <0.0001 |
| Bandonia_marina | 0.03257581 | -2.137272834 | * | 0.11772223 | 0.0326 |
| Talaromyces_funiculosus | 7.26E-07 | -4.954369105 | *** | 1.36E-05 | <0.0001 |
| Chrysosphaeria_jan-nelii | 3.41E-07 | -5.099499065 | *** | 7.60E-06 | <0.0001 |
| Dioszegia_zsoltii_var._yunnanensis | 0.01445073 | -2.445859511 | * | 0.06748574 | 0.0145 |
| Togniniella_acerosa | 0.001325 | -3.210508589 | ** | 0.01006234 | 0.0013 |
| Nigrospora_oryzae | 0.00113471 | -3.254803107 | ** | 0.00929044 | 0.0011 |
| Aspergillus_versicolor | 0.00433138 | -2.85296174 | ** | 0.0276786 | 0.0043 |
| Bjerkandera_mikrofumosa | 0.00272423 | -2.997254698 | ** | 0.01878285 | 0.0027 |
| Kodamaea_ohmeri | 0.01532596 | -2.424583476 | * | 0.06748574 | 0.0153 |
| Saccharomyces_cerevisiae | 2.74E-05 | -4.19371999 | *** | 3.51E-04 | <0.0001 |
| Kazachstania_heterogenica | 0.02272284 | -2.278062769 | * | 0.08819828 | 0.0227 |
| Angustimassarina_camporesii | 0.00548137 | -2.777293023 | ** | 0.03059143 | 0.0055 |
| Rhexothecium_globosum | 5.95E-06 | -4.528212349 | *** | 8.66E-05 | <0.0001 |
| Sporobolomyces_spe_Incertae_sedis | 9.24E-09 | -5.744085541 | *** | 3.03E-07 | <0.0001 |
| Bovista_psammophila | 3.05E-07 | -5.120235561 | *** | 7.60E-06 | <0.0001 |
| Zygotorulaspora_danielsina | 0.00536238 | -2.784418721 | ** | 0.03059143 | 0.0054 |
| Pleosporales_spe_Incertae_sedis | 3.36E-05 | -4.147726265 | *** | 4.00E-04 | <0.0001 |
| Coprinellus_spe_Incertae_sedis | 3.95E-05 | -4.110357357 | *** | 4.50E-04 | <0.0001 |
| Cystofilobasidium_macerans | 0.00272423 | -2.997254698 | ** | 0.01878285 | 0.0027 |
| Tausonia_pullulans | 0.0210367 | -2.307325107 | * | 0.08350934 | 0.021 |
| Ophiostoma_acarorum | 5.49E-07 | -5.008281663 | *** | 1.11E-05 | <0.0001 |
| Candida_zeylanoides | 0.00349577 | -2.920404513 | ** | 0.02318715 | 0.0035 |
| Amylostereum_orientale | 2.08E-04 | -3.709204769 | *** | 0.00201751 | 0.0002 |
| Neopestalotiopsis_foedans | 0.01532596 | -2.424583476 | * | 0.06748574 | 0.0153 |
| Diaporthales_spe_Incertae_sedis | 0.02099287 | -2.308112665 | * | 0.08350934 | 0.021 |
| Gibberella_fujikuroi | 0.0425832 | -2.027775202 | * | 0.13859376 | 0.0426 |
| Tremellomycetes_spe_Incertae_sedis | 0.01450985 | -2.444386987 | * | 0.06748574 | 0.0145 |
| Fantasmomyces_hyalinus | 5.19E-04 | -3.47054224 | *** | 0.00453617 | 0.0005 |
| Pleurotus_eryngii | 0.00272403 | -2.997277256 | ** | 0.01878285 | 0.0027 |
| Phanerochaete_chrysosporium | 0.00941639 | -2.596554573 | ** | 0.05034889 | 0.0094 |
| Apiotrichum_domesticum | 1.70E-07 | -5.22931058 | *** | 4.69E-06 | <0.0001 |
| Cladosporium_coloradense | 0.04373102 | -2.016659869 | * | 0.14145096 | 0.0437 |
| Leptosporella_bambusae | 6.12E-06 | -4.522100825 | *** | 8.67E-05 | <0.0001 |
| Phyllachora_cynodonticola | 1.52E-04 | -3.787360578 | *** | 0.00156436 | 0.0002 |
| Capnodiales_spe_Incertae_sedis | 0.01083701 | -2.547911125 | * | 0.05567441 | 0.0108 |
| Ustilaginoidea_virens | 2.84E-07 | -5.133495645 | *** | 7.45E-06 | <0.0001 |
| Malassezia_slooffiae | 0.01532596 | -2.424583476 | * | 0.06748574 | 0.0153 |
| Peroneutypa_scoparia | 0.02203534 | -2.289758121 | * | 0.08616805 | 0.022 |
| Naganishia_globosa | 0.02901195 | -2.18332411 | * | 0.10557126 | 0.029 |
| Peniophora_pseudoversicolor | 0.0022053 | -3.061093313 | ** | 0.01604971 | 0.0022 |
| Talaromyces_spe_Incertae_sedis | 0.01532596 | -2.424583476 | * | 0.06748574 | 0.0153 |
| Coprinopsis_urticicola | 0.00548758 | -2.776925362 | ** | 0.03059143 | 0.0055 |
| Typhula_micans | 6.31E-04 | -3.417919401 | *** | 0.00533312 | 0.0006 |
| Thielaviopsis_euricoi | 0.03786201 | -2.076345436 | * | 0.13152529 | 0.0379 |
| Flavodon_spe_Incertae_sedis | 0.02099287 | -2.308112665 | * | 0.08350934 | 0.021 |
| Yarrowia_spe_Incertae_sedis | 0.00790748 | -2.655995134 | ** | 0.04271667 | 0.0079 |
| Dipodascus_geotrichum | 0.0051226 | -2.799221555 | ** | 0.0298249 | 0.0051 |
| Cutaneotrichosporon_curvatus | 0.01532596 | -2.424583476 | * | 0.06748574 | 0.0153 |
| Exophiala_halophila | 0.01532596 | -2.424583476 | * | 0.06748574 | 0.0153 |
| Novakomyces_olei | 0.04063099 | -2.047276005 | * | 0.1330665 | 0.0406 |
| Trichophaea_saccata | 0.01197363 | -2.512920683 | * | 0.06091437 | 0.012 |
| Tilletiopsis_washingtonensis | 0.03718789 | -2.083695338 | * | 0.13152529 | 0.0372 |
| Auricularia_auricula-judae | 0.02099216 | -2.308125437 | * | 0.08350934 | 0.021 |
| Diutina_sipiczkii | 1.87E-04 | -3.735577947 | *** | 0.00187574 | 0.0002 |
| Keissleriella_quadriseptata | 0.02099287 | -2.308112665 | * | 0.08350934 | 0.021 |
| Candida_inconspicua | 0.00508758 | -2.801435542 | ** | 0.0298249 | 0.0051 |
| Zoophthora_radicans | 0.00373977 | -2.899317054 | ** | 0.02449546 | 0.0037 |
| Ceriporia_lacerata | 0.001325 | -3.210508589 | ** | 0.01006234 | 0.0013 |
| Vishniacozyma_spe_Incertae_sedis | 0.00189869 | -3.105638298 | ** | 0.01421304 | 0.0019 |
| Cercospora_canescens | 0.00745021 | -2.676020268 | ** | 0.04109381 | 0.0075 |
| Exidia_pithya | 0.00471635 | -2.825794345 | ** | 0.02808374 | 0.0047 |
| Simplicillium_spe_Incertae_sedis | 0.0267255 | -2.215503849 | * | 0.10002973 | 0.0267 |
| Neodevriesia_lagerstroemiae | 0.01532596 | -2.424583476 | * | 0.06748574 | 0.0153 |
| Peniophorella_spe_Incertae_sedis | 0.01511927 | -2.429509461 | * | 0.06748574 | 0.0151 |
| Acrocalymma_fici | 0.01083739 | -2.547898967 | * | 0.05567441 | 0.0108 |
| Aspergillus_coremiiformis | 0.02901195 | -2.18332411 | * | 0.10557126 | 0.029 |
| Onygenales_spe_Incertae_sedis | 0.04915517 | -1.967243249 | * | 0.15705678 | 0.0492 |
| Diaporthe_caulivora | 0.01511927 | -2.429509461 | * | 0.06748574 | 0.0151 |
| Hypoxylon_perforatum | 0.02901098 | -2.183337251 | * | 0.10557126 | 0.029 |
| Zasmidium_fructigenum | 0.01532596 | -2.424583476 | * | 0.06748574 | 0.0153 |
| Tilletia_spe_Incertae_sedis | 0.00132485 | -3.21054147 | ** | 0.01006234 | 0.0013 |
| Ciliophora_spe_Incertae_sedis | 0.04615593 | -1.993964578 | * | 0.14837856 | 0.0462 |
| Yarrowia_bubula | 0.02099287 | -2.308112665 | * | 0.08350934 | 0.021 |
| Peniophora_cinerea | 0.02099287 | -2.308112665 | * | 0.08350934 | 0.021 |
| Agaricales_spe_Incertae_sedis | 0.0267255 | -2.215503849 | * | 0.10002973 | 0.0267 |
| [Cryptococcus]_ater | 0.00548777 | -2.77691369 | ** | 0.03059143 | 0.0055 |
| Rosellinia_australiensis | 3.06E-04 | -3.610560757 | *** | 0.00276036 | 0.0003 |

| Data S6. Composition and average abundance of gut mycobiome at the phylum level between three groups | | | |
| --- | --- | --- | --- |
| ID | CPR0 | CPR1 | HC |
| Ascomycota | 0.811384647 | 0.820096792 | 0.827027767 |
| Fungi_phy_Incertae_sedis | 0.099265882 | 0.143662125 | 0.124301244 |
| Basidiomycota | 0.055963235 | 0.02880725 | 0.033201589 |
| Mucoromycota | 0.033205235 | 0.00419375 | 0.0145814 |
| Chytridiomycota | 2.58E-05 | 0.0031195 | 4.03E-04 |
| Rozellomycota | 1.03E-04 | 0 | 3.78E-04 |
| Zoopagomycota | 5.26E-05 | 1.21E-04 | 6.83E-05 |
| Blastocladiomycota | 0 | 0 | 3.89E-05 |

| Data S7. Composition and average abundance of gut mycobiome at the genus level between three groups | | | |
| --- | --- | --- | --- |
| ID | CPR0 | CPR1 | HC |
| Ascomycota_gen_Incertae_sedis | 0.001602647 | 0.002546125 | 0.286155867 |
| Acrodictys | 0.172191235 | 0.166309 | 0.097041478 |
| Zanclospora | 0.086692882 | 0.243307583 | 0.080829956 |
| Saccharomyces | 0.008514824 | 0.096199333 | 0.130613367 |
| Fungi_gen_Incertae_sedis | 0.042711294 | 0.059496542 | 0.080916211 |
| Entorrhiza | 0.056554588 | 0.084165583 | 0.043385033 |
| Aspergillus | 0.049584412 | 0.085012833 | 0.043377756 |
| Candida | 0.105924647 | 0.029513208 | 0.034928922 |
| Dipodascaceae_gen_Incertae_sedis | 0.221913412 | 0.008293458 | 7.24E-04 |
| Hydnobolites | 7.64E-04 | 0.027388833 | 0.029797167 |
| Debaryomyces | 0.015605059 | 0.041530958 | 0.011872856 |
| Talaromyces | 1.56E-04 | 0.008053375 | 0.019577689 |
| Dipodascus | 0.097269 | 0.002322667 | 7.91E-04 |
| Heitmania | 5.55E-04 | 0.015636333 | 0.009285044 |
| Trigonopsis | 1.25E-05 | 7.50E-06 | 0.0129861 |
| Diversispora | 0.001523294 | 6.93E-04 | 0.011931589 |
| Dothiora | 0.003143235 | 0.015555667 | 0.007140344 |
| Saccharomycetales_gen_Incertae_sedis | 0.005992353 | 0.001170083 | 0.008114011 |
| Pseudeurotiaceae_gen_Incertae_sedis | 0 | 0 | 0.009337656 |
| Malassezia | 0.001490412 | 0.003393917 | 0.007377822 |
| Rhodotorula | 0.041707647 | 3.16E-04 | 1.07E-04 |
| Iodosphaeria | 0 | 0.010973208 | 0.0044895 |
| Milospium | 8.18E-06 | 0.019479208 | 8.68E-04 |
| Arthrographis | 6.68E-05 | 4.13E-05 | 0.005839178 |
| Blumeria | 5.13E-04 | 0.008163333 | 0.003523111 |
| Simplicillium | 0.002225176 | 0.004053333 | 0.003988711 |
| Wallemia | 5.75E-04 | 9.29E-05 | 0.005115678 |
| Yarrowia | 0.013988647 | 2.99E-04 | 0.002224367 |
| Mucor | 0.024573059 | 4.48E-04 | 1.31E-04 |
| Actinomucor | 0.007108882 | 0.002879167 | 0.002279356 |
| Parafuscosporella | 0.003686706 | 0.003501375 | 0.002192256 |
| Eremothecium | 9.35E-05 | 0.0139465 | 2.42E-05 |
| Angustimassarina | 3.22E-04 | 0.0012035 | 0.002507067 |
| Raffaelea | 5.76E-04 | 0.006910292 | 7.16E-04 |
| Ciliophora | 0 | 1.16E-05 | 0.002504467 |
| Alternaria | 0.002211412 | 7.33E-04 | 0.001625489 |
| Cladosporium | 0.002832294 | 0.0027045 | 9.24E-04 |
| Pichia | 1.95E-04 | 1.19E-04 | 0.001848922 |
| Fantasmomyces | 8.36E-04 | 0.002027292 | 0.001207211 |
| Apiotrichum | 4.62E-05 | 4.03E-04 | 0.001772811 |
| Zygotorulaspora | 0.001762235 | 0.001293333 | 0.001199422 |
| Tausonia | 7.19E-05 | 1.32E-04 | 0.001801233 |
| Tuber | 1.55E-04 | 0.001033375 | 0.001290267 |
| Cutaneotrichosporon | 1.48E-04 | 0.004755792 | 2.34E-04 |
| Chrysosphaeria | 0 | 2.22E-04 | 0.001306222 |
| Entyloma | 0.006937765 | 2.46E-05 | 2.70E-05 |
| Clavispora | 2.22E-05 | 0 | 0.001332478 |
| Chytridium | 2.58E-05 | 0.0031195 | 4.00E-04 |
| Kazachstania | 4.29E-04 | 0.001329542 | 7.48E-04 |
| Fusarium | 4.15E-04 | 0.00156925 | 6.13E-04 |
| Others | 0.016266294 | 0.017618542 | 0.020974789 |

| Data S8. Comparison of gut mycobiome at the phylum level  between three groups | | | | |
| --- | --- | --- | --- | --- |
| ID | Fungi_phy_Incertae_sedis | Mucoromycota | Chytridiomycota | Rozellomycota |
| CPR0.median | 0.0297(0.0192,0.1275) | 0.019(0.008,0.0442) | <0.0001(<0.0001,<0.0001) | <0.0001(<0.0001,<0.0001) |
| CPR0.mean | 0.099265882 | 0.033205235 | 2.58E-05 | 1.03E-04 |
| CPR0.se | 0.033135451 | 0.009947401 | 2.58E-05 | 6.15E-05 |
| CPR1.median | 0.0726(0.0435,0.2127) | 0.0005(0.0002,0.0011) | 0.0002(<0.0001,0.0026) | <0.0001(<0.0001,<0.0001) |
| CPR1.mean | 0.143662125 | 0.00419375 | 0.0031195 | 0 |
| CPR1.se | 0.032869915 | 0.003268933 | 0.001868019 | 0 |
| HC.median | 0.0841(0.0534,0.1277) | 0.0004(<0.0001,0.0011) | <0.0001(<0.0001,0.0001) | <0.0001(<0.0001,<0.0001) |
| HC.mean | 0.124301244 | 0.0145814 | 4.03E-04 | 3.78E-04 |
| HC.se | 0.011803062 | 0.007597828 | 1.27E-04 | 2.73E-04 |
| p-value | 0.028251234 | 1.65E-06 | 0.001835719 | 2.11E-07 |
| z-score | -2.193779932 | -4.791792517 | -3.115598768 | -5.189212634 |
| Sig_mark | * | *** | ** | *** |
| q-value | 0.056502468 | 6.61E-06 | 0.00489525 | 1.69E-06 |
| fixp | 0.0283 | <0.0001 | 0.0018 | <0.0001 |
| fixps | * 0.0283 | ***<0.0001 | ** 0.0018 | ***<0.0001 |

| Data S9. Comparison of gut mycobiome at the genus level between three groups | | | |
| --- | --- | --- | --- |
| ID | CPR0.median | CPR0.mean | CPR0.se |
| Ascomycota_gen_Incertae_sedis | 0.0006(0.0003,0.0027) | 0.001602647 | 4.87E-04 |
| Zanclospora | 0.0596(0.0264,0.1083) | 0.086692882 | 0.019851495 |
| Saccharomyces | 0.0034(0.0002,0.0149) | 0.008514824 | 0.002924222 |
| Fungi_gen_Incertae_sedis | 0.0207(0.0049,0.0538) | 0.042711294 | 0.014212868 |
| Entorrhiza | 0.008(0.0017,0.0234) | 0.056554588 | 0.028328732 |
| Candida | 0.0491(0.007,0.1539) | 0.105924647 | 0.031040425 |
| Dipodascaceae_gen_Incertae_sedis | 0.0539(0.0053,0.3745) | 0.221913412 | 0.062577334 |
| Hydnobolites | <0.0001(<0.0001,<0.0001) | 7.64E-04 | 6.92E-04 |
| Debaryomyces | 0.0034(0.0001,0.0308) | 0.015605059 | 0.005517021 |
| Talaromyces | 0.0001(<0.0001,0.0002) | 1.56E-04 | 2.37E-05 |
| Dipodascus | 0.0012(0.0007,0.0247) | 0.097269 | 0.0491193 |
| Heitmania | 0.0002(<0.0001,0.0004) | 5.55E-04 | 3.39E-04 |
| Trigonopsis | <0.0001(<0.0001,<0.0001) | 1.25E-05 | 9.35E-06 |
| Dothiora | 0.0002(0.0001,0.0006) | 0.003143235 | 0.001701147 |
| Saccharomycetales_gen_Incertae_sedis | 0.0025(<0.0001,0.007) | 0.005992353 | 0.002508712 |
| Pseudeurotiaceae_gen_Incertae_sedis | <0.0001(<0.0001,<0.0001) | 0 | 0 |
| Rhodotorula | 0.0007(<0.0001,0.0013) | 0.041707647 | 0.041001542 |
| Iodosphaeria | <0.0001(<0.0001,<0.0001) | 0 | 0 |
| Milospium | <0.0001(<0.0001,<0.0001) | 8.18E-06 | 5.89E-06 |
| Arthrographis | <0.0001(<0.0001,<0.0001) | 6.68E-05 | 2.86E-05 |
| Blumeria | 0.0004(0.0003,0.0006) | 5.13E-04 | 8.52E-05 |
| Wallemia | 0.0002(<0.0001,0.0004) | 5.75E-04 | 3.77E-04 |
| Mucor | 0.0127(0.0006,0.0249) | 0.024573059 | 0.009854202 |
| Actinomucor | 0.0005(<0.0001,0.0067) | 0.007108882 | 0.003928425 |
| Parafuscosporella | 0.0011(0.0005,0.0015) | 0.003686706 | 0.00163938 |
| Eremothecium | <0.0001(<0.0001,<0.0001) | 9.35E-05 | 6.19E-05 |
| Angustimassarina | <0.0001(<0.0001,0.0002) | 3.22E-04 | 1.91E-04 |
| Raffaelea | 0.0005(0.0004,0.0008) | 5.76E-04 | 6.83E-05 |
| Ciliophora | <0.0001(<0.0001,<0.0001) | 0 | 0 |
| Alternaria | 0.0001(<0.0001,0.0003) | 0.002211412 | 0.001428443 |
| Cladosporium | 0.0008(0.0003,0.0012) | 0.002832294 | 0.001427592 |
| Fantasmomyces | <0.0001(<0.0001,0.0005) | 8.36E-04 | 3.96E-04 |
| Apiotrichum | <0.0001(<0.0001,<0.0001) | 4.62E-05 | 9.17E-06 |
| Tuber | <0.0001(<0.0001,<0.0001) | 1.55E-04 | 7.97E-05 |
| Cutaneotrichosporon | <0.0001(<0.0001,<0.0001) | 1.48E-04 | 1.18E-04 |
| Chrysosphaeria | <0.0001(<0.0001,<0.0001) | 0 | 0 |
| Entyloma | <0.0001(<0.0001,0.0002) | 0.006937765 | 0.004716166 |
| Chytridium | <0.0001(<0.0001,<0.0001) | 2.58E-05 | 2.58E-05 |
| Fusarium | <0.0001(<0.0001,<0.0001) | 4.15E-04 | 3.16E-04 |
| Cryptococcus | 0.0003(0.0002,0.0003) | 2.90E-04 | 3.65E-05 |
| Calosphaeria | 0.0001(<0.0001,0.0005) | 0.001372176 | 6.89E-04 |
| Diplodia | <0.0001(<0.0001,<0.0001) | 0 | 0 |
| Schizophyllum | <0.0001(<0.0001,<0.0001) | 3.29E-06 | 3.29E-06 |
| Auricularia | <0.0001(<0.0001,<0.0001) | 0 | 0 |
| Issatchenkia | <0.0001(<0.0001,0.0006) | 9.86E-04 | 6.91E-04 |
| Penicillium | <0.0001(<0.0001,0.0001) | 3.18E-04 | 2.39E-04 |
| Acremonium | <0.0001(<0.0001,<0.0001) | 0.002307412 | 0.002246035 |
| Pleurotus | <0.0001(<0.0001,0.0001) | 1.03E-04 | 4.00E-05 |
| Rozellomycota_gen_Incertae_sedis | <0.0001(<0.0001,<0.0001) | 5.27E-05 | 1.41E-05 |
| Ustilaginoidea | <0.0001(<0.0001,<0.0001) | 0.001556824 | 0.001540443 |
| Mrakia | <0.0001(<0.0001,<0.0001) | 0.001392588 | 0.00138728 |
| Diaporthe | <0.0001(<0.0001,<0.0001) | 0.001359765 | 0.001340848 |
| Tetracladium | <0.0001(<0.0001,<0.0001) | 0 | 0 |
| Paraphoma | <0.0001(<0.0001,<0.0001) | 0 | 0 |
| Leptospora | <0.0001(<0.0001,<0.0001) | 4.28E-05 | 1.63E-05 |
| Acrodontium | <0.0001(<0.0001,<0.0001) | 0 | 0 |

| Data S9. Comparison of gut mycobiome at the genus level between three groups | | | |
| --- | --- | --- | --- |
| ID | CPR1.median | CPR1.mean | CPR1.se |
| Ascomycota_gen_Incertae_sedis | 0.0002(<0.0001,0.0007) | 0.002546125 | 0.00142119 |
| Zanclospora | 0.1064(0.0185,0.4387) | 0.243307583 | 0.054922831 |
| Saccharomyces | 0.001(0.0003,0.1187) | 0.096199333 | 0.03486446 |
| Fungi_gen_Incertae_sedis | 0.0379(0.0162,0.0728) | 0.059496542 | 0.015053855 |
| Entorrhiza | 0.0418(0.0177,0.1098) | 0.084165583 | 0.022977144 |
| Candida | 0.0017(0.0002,0.0168) | 0.029513208 | 0.017610435 |
| Dipodascaceae_gen_Incertae_sedis | <0.0001(<0.0001,0.0003) | 0.008293458 | 0.007641268 |
| Hydnobolites | 0.0007(<0.0001,0.0369) | 0.027388833 | 0.010118923 |
| Debaryomyces | 0.0009(0.0002,0.0128) | 0.041530958 | 0.025784903 |
| Talaromyces | <0.0001(<0.0001,0.0004) | 0.008053375 | 0.007045947 |
| Dipodascus | <0.0001(<0.0001,<0.0001) | 0.002322667 | 0.001688246 |
| Heitmania | 0.0008(0.0002,0.0047) | 0.015636333 | 0.01052983 |
| Trigonopsis | <0.0001(<0.0001,<0.0001) | 7.50E-06 | 7.50E-06 |
| Dothiora | 0.0067(0.0014,0.0307) | 0.015555667 | 0.004212284 |
| Saccharomycetales_gen_Incertae_sedis | <0.0001(<0.0001,0.0002) | 0.001170083 | 7.26E-04 |
| Pseudeurotiaceae_gen_Incertae_sedis | <0.0001(<0.0001,<0.0001) | 0 | 0 |
| Rhodotorula | <0.0001(<0.0001,<0.0001) | 3.16E-04 | 2.03E-04 |
| Iodosphaeria | <0.0001(<0.0001,0.0047) | 0.010973208 | 0.004615154 |
| Milospium | <0.0001(<0.0001,0.0006) | 0.019479208 | 0.018788332 |
| Arthrographis | <0.0001(<0.0001,<0.0001) | 4.13E-05 | 1.64E-05 |
| Blumeria | <0.0001(<0.0001,0.0004) | 0.008163333 | 0.006371913 |
| Wallemia | <0.0001(<0.0001,<0.0001) | 9.29E-05 | 4.97E-05 |
| Mucor | <0.0001(<0.0001,<0.0001) | 4.48E-04 | 3.26E-04 |
| Actinomucor | <0.0001(<0.0001,<0.0001) | 0.002879167 | 0.002789442 |
| Parafuscosporella | 0.0021(0.0009,0.0049) | 0.003501375 | 8.08E-04 |
| Eremothecium | <0.0001(<0.0001,<0.0001) | 0.0139465 | 0.011768265 |
| Angustimassarina | 0.0005(0.0001,0.0014) | 0.0012035 | 4.64E-04 |
| Raffaelea | <0.0001(<0.0001,<0.0001) | 0.006910292 | 0.006652735 |
| Ciliophora | <0.0001(<0.0001,<0.0001) | 1.16E-05 | 1.16E-05 |
| Alternaria | <0.0001(<0.0001,0.0001) | 7.33E-04 | 3.33E-04 |
| Cladosporium | 0.0001(<0.0001,0.0016) | 0.0027045 | 0.00135067 |
| Fantasmomyces | 0.0007(0.0002,0.0026) | 0.002027292 | 6.64E-04 |
| Apiotrichum | <0.0001(<0.0001,<0.0001) | 4.03E-04 | 4.02E-04 |
| Tuber | 0.0004(0.0001,0.0017) | 0.001033375 | 2.65E-04 |
| Cutaneotrichosporon | <0.0001(<0.0001,<0.0001) | 0.004755792 | 0.004711904 |
| Chrysosphaeria | <0.0001(<0.0001,<0.0001) | 2.22E-04 | 1.43E-04 |
| Entyloma | <0.0001(<0.0001,<0.0001) | 2.46E-05 | 2.24E-05 |
| Chytridium | 0.0002(<0.0001,0.0026) | 0.0031195 | 0.001868019 |
| Fusarium | <0.0001(<0.0001,0.0008) | 0.00156925 | 7.76E-04 |
| Cryptococcus | <0.0001(<0.0001,<0.0001) | 9.50E-05 | 7.85E-05 |
| Calosphaeria | 0.0005(0.0001,0.0013) | 9.11E-04 | 2.42E-04 |
| Diplodia | <0.0001(<0.0001,<0.0001) | 0 | 0 |
| Schizophyllum | <0.0001(<0.0001,<0.0001) | 0 | 0 |
| Auricularia | <0.0001(<0.0001,<0.0001) | 1.57E-05 | 1.38E-05 |
| Issatchenkia | <0.0001(<0.0001,<0.0001) | 0.001056417 | 0.001055678 |
| Penicillium | <0.0001(<0.0001,<0.0001) | 1.75E-04 | 1.53E-04 |
| Acremonium | <0.0001(<0.0001,<0.0001) | 1.66E-04 | 1.22E-04 |
| Pleurotus | <0.0001(<0.0001,0.0001) | 8.31E-04 | 5.13E-04 |
| Rozellomycota_gen_Incertae_sedis | <0.0001(<0.0001,<0.0001) | 0 | 0 |
| Ustilaginoidea | <0.0001(<0.0001,<0.0001) | 1.13E-06 | 1.13E-06 |
| Mrakia | <0.0001(<0.0001,<0.0001) | 3.25E-06 | 3.25E-06 |
| Diaporthe | <0.0001(<0.0001,<0.0001) | 0 | 0 |
| Tetracladium | <0.0001(<0.0001,<0.0001) | 8.79E-04 | 8.73E-04 |
| Paraphoma | <0.0001(<0.0001,<0.0001) | 8.70E-04 | 7.92E-04 |
| Leptospora | <0.0001(<0.0001,<0.0001) | 1.75E-04 | 1.75E-04 |
| Acrodontium | <0.0001(<0.0001,<0.0001) | 4.94E-04 | 4.92E-04 |

| Data S9. Comparison of gut mycobiome at the genus level between three groups | | | |
| --- | --- | --- | --- |
| ID | HC.median | HC.mean | HC.se |
| Ascomycota_gen_Incertae_sedis | 0.2529(0.1987,0.3192) | 0.286155867 | 0.014734265 |
| Zanclospora | 0.0215(0.004,0.0986) | 0.080829956 | 0.01406146 |
| Saccharomyces | 0.0134(0.0007,0.1823) | 0.130613367 | 0.021928774 |
| Fungi_gen_Incertae_sedis | 0.0689(0.0475,0.097) | 0.080916211 | 0.004827277 |
| Entorrhiza | 0.0044(0.0005,0.0451) | 0.043385033 | 0.007949247 |
| Candida | 0.003(0.0003,0.02) | 0.034928922 | 0.010424901 |
| Dipodascaceae_gen_Incertae_sedis | <0.0001(<0.0001,<0.0001) | 7.24E-04 | 3.28E-04 |
| Hydnobolites | 0.0005(<0.0001,0.0054) | 0.029797167 | 0.00966847 |
| Debaryomyces | <0.0001(<0.0001,0.0004) | 0.011872856 | 0.008986662 |
| Talaromyces | 0.0003(<0.0001,0.0013) | 0.019577689 | 0.009367828 |
| Dipodascus | <0.0001(<0.0001,<0.0001) | 7.91E-04 | 2.93E-04 |
| Heitmania | 0.0008(<0.0001,0.0049) | 0.009285044 | 0.002763651 |
| Trigonopsis | <0.0001(<0.0001,0.0003) | 0.0129861 | 0.007856359 |
| Dothiora | 0.0005(<0.0001,0.0056) | 0.007140344 | 0.001504987 |
| Saccharomycetales_gen_Incertae_sedis | <0.0001(<0.0001,<0.0001) | 0.008114011 | 0.003928305 |
| Pseudeurotiaceae_gen_Incertae_sedis | <0.0001(<0.0001,<0.0001) | 0.009337656 | 0.009279614 |
| Rhodotorula | <0.0001(<0.0001,<0.0001) | 1.07E-04 | 9.34E-05 |
| Iodosphaeria | <0.0001(<0.0001,0.0001) | 0.0044895 | 0.00196775 |
| Milospium | <0.0001(<0.0001,0.0001) | 8.68E-04 | 4.49E-04 |
| Arthrographis | <0.0001(<0.0001,0.0002) | 0.005839178 | 0.004169621 |
| Blumeria | <0.0001(<0.0001,0.0003) | 0.003523111 | 0.00212671 |
| Wallemia | <0.0001(<0.0001,<0.0001) | 0.005115678 | 0.003015236 |
| Mucor | <0.0001(<0.0001,<0.0001) | 1.31E-04 | 7.86E-05 |
| Actinomucor | <0.0001(<0.0001,<0.0001) | 0.002279356 | 0.002127329 |
| Parafuscosporella | 0.0008(<0.0001,0.0024) | 0.002192256 | 3.88E-04 |
| Eremothecium | <0.0001(<0.0001,<0.0001) | 2.42E-05 | 2.39E-05 |
| Angustimassarina | 0.0002(<0.0001,0.002) | 0.002507067 | 0.001172194 |
| Raffaelea | <0.0001(<0.0001,<0.0001) | 7.16E-04 | 2.91E-04 |
| Ciliophora | <0.0001(<0.0001,<0.0001) | 0.002504467 | 0.002425395 |
| Alternaria | <0.0001(<0.0001,<0.0001) | 0.001625489 | 9.41E-04 |
| Cladosporium | <0.0001(<0.0001,<0.0001) | 9.24E-04 | 3.12E-04 |
| Fantasmomyces | <0.0001(<0.0001,0.0008) | 0.001207211 | 2.94E-04 |
| Apiotrichum | <0.0001(<0.0001,<0.0001) | 0.001772811 | 0.001739553 |
| Tuber | <0.0001(<0.0001,0.0005) | 0.001290267 | 9.02E-04 |
| Cutaneotrichosporon | <0.0001(<0.0001,<0.0001) | 2.34E-04 | 1.53E-04 |
| Chrysosphaeria | <0.0001(<0.0001,<0.0001) | 0.001306222 | 0.001169272 |
| Entyloma | <0.0001(<0.0001,<0.0001) | 2.70E-05 | 2.06E-05 |
| Chytridium | <0.0001(<0.0001,0.0001) | 4.00E-04 | 1.27E-04 |
| Fusarium | <0.0001(<0.0001,<0.0001) | 6.13E-04 | 2.99E-04 |
| Cryptococcus | <0.0001(<0.0001,<0.0001) | 0.001020544 | 3.22E-04 |
| Calosphaeria | <0.0001(<0.0001,0.0006) | 5.96E-04 | 1.57E-04 |
| Diplodia | <0.0001(<0.0001,<0.0001) | 0.0010858 | 0.001074575 |
| Schizophyllum | <0.0001(<0.0001,<0.0001) | 8.23E-04 | 7.44E-04 |
| Auricularia | <0.0001(<0.0001,<0.0001) | 7.67E-04 | 4.25E-04 |
| Issatchenkia | <0.0001(<0.0001,<0.0001) | 2.20E-04 | 2.06E-04 |
| Penicillium | <0.0001(<0.0001,<0.0001) | 4.90E-04 | 2.81E-04 |
| Acremonium | <0.0001(<0.0001,<0.0001) | 6.08E-05 | 5.35E-05 |
| Pleurotus | <0.0001(<0.0001,<0.0001) | 1.66E-04 | 9.48E-05 |
| Rozellomycota_gen_Incertae_sedis | <0.0001(<0.0001,<0.0001) | 3.66E-04 | 2.73E-04 |
| Ustilaginoidea | <0.0001(<0.0001,<0.0001) | 0 | 0 |
| Mrakia | <0.0001(<0.0001,<0.0001) | 0 | 0 |
| Diaporthe | <0.0001(<0.0001,<0.0001) | 1.72E-06 | 1.72E-06 |
| Tetracladium | <0.0001(<0.0001,<0.0001) | 3.08E-06 | 3.08E-06 |
| Paraphoma | <0.0001(<0.0001,<0.0001) | 0 | 0 |
| Leptospora | <0.0001(<0.0001,<0.0001) | 1.54E-04 | 1.53E-04 |
| Acrodontium | <0.0001(<0.0001,<0.0001) | 0 | 0 |

| Data S9. Comparison of gut mycobiome at the genus level between three groups | | | | | |
| --- | --- | --- | --- | --- | --- |
| ID | p-value | z-score | Sig_mark | q-value | fixp |
| Ascomycota_gen_Incertae_sedis | 4.99E-19 | -8.91239 | *** | 1.39E-16 | <0.0001 |
| Zanclospora | 0.0071376 | -2.690353 | ** | 0.0301709 | 0.0071 |
| Saccharomyces | 0.0210999 | -2.306193 | * | 0.0698305 | 0.0211 |
| Fungi_gen_Incertae_sedis | 7.19E-06 | -4.487919 | *** | 7.14E-05 | <0.0001 |
| Entorrhiza | 0.0162153 | -2.404032 | * | 0.0556526 | 0.0162 |
| Candida | 0.0019527 | -3.097336 | ** | 0.0092008 | 0.002 |
| Dipodascaceae_gen_Incertae_sedis | 3.38E-11 | -6.628878 | *** | 1.34E-09 | <0.0001 |
| Hydnobolites | 1.38E-05 | -4.346812 | *** | 1.32E-04 | <0.0001 |
| Debaryomyces | 1.48E-06 | -4.813751 | *** | 1.79E-05 | <0.0001 |
| Talaromyces | 0.0142417 | -2.45111 | * | 0.0506991 | 0.0142 |
| Dipodascus | 1.01E-10 | -6.465503 | *** | 3.51E-09 | <0.0001 |
| Heitmania | 0.0454997 | -2.000005 | * | 0.1160451 | 0.0455 |
| Trigonopsis | 1.18E-07 | -5.295865 | *** | 2.06E-06 | <0.0001 |
| Dothiora | 0.0024613 | -3.028054 | ** | 0.0110363 | 0.0025 |
| Saccharomycetales_gen_Incertae_sedis | 0.0071629 | -2.689175 | ** | 0.0301709 | 0.0072 |
| Pseudeurotiaceae_gen_Incertae_sedis | 0.0124319 | -2.499641 | * | 0.0454747 | 0.0124 |
| Rhodotorula | 5.38E-08 | -5.438226 | *** | 1.15E-06 | <0.0001 |
| Iodosphaeria | 1.96E-05 | -4.26938 | *** | 1.70E-04 | <0.0001 |
| Milospium | 8.77E-04 | -3.327345 | *** | 0.0051861 | 0.0009 |
| Arthrographis | 0.0276365 | -2.202408 | * | 0.0883097 | 0.0276 |
| Blumeria | 0.001157 | -3.249267 | ** | 0.0058742 | 0.0012 |
| Wallemia | 0.0138499 | -2.461134 | * | 0.0500034 | 0.0138 |
| Mucor | 1.84E-15 | -7.951622 | *** | 1.28E-13 | <0.0001 |
| Actinomucor | 3.64E-06 | -4.630717 | *** | 3.90E-05 | <0.0001 |
| Parafuscosporella | 0.0414823 | -2.038677 | * | 0.1067785 | 0.0415 |
| Eremothecium | 5.97E-10 | -6.191129 | *** | 1.85E-08 | <0.0001 |
| Angustimassarina | 0.0343783 | -2.115606 | * | 0.0926172 | 0.0344 |
| Raffaelea | 5.98E-08 | -5.419446 | *** | 1.19E-06 | <0.0001 |
| Ciliophora | 0.0285863 | -2.189145 | * | 0.0903067 | 0.0286 |
| Alternaria | 0.002201 | -3.061682 | ** | 0.0100749 | 0.0022 |
| Cladosporium | 1.20E-06 | -4.855292 | *** | 1.52E-05 | <0.0001 |
| Fantasmomyces | 0.0144073 | -2.446945 | * | 0.0506991 | 0.0144 |
| Apiotrichum | 4.96E-05 | -4.057447 | *** | 3.54E-04 | <0.0001 |
| Tuber | 8.28E-04 | -3.343189 | *** | 0.0050053 | 0.0008 |
| Cutaneotrichosporon | 0.0383757 | -2.070819 | * | 0.0997052 | 0.0384 |
| Chrysosphaeria | 0.0201891 | -2.322816 | * | 0.0676212 | 0.0202 |
| Entyloma | 5.09E-12 | -6.902929 | *** | 2.36E-10 | <0.0001 |
| Chytridium | 0.0018104 | -3.119697 | ** | 0.0086773 | 0.0018 |
| Fusarium | 0.0380704 | -2.074096 | * | 0.0997052 | 0.0381 |
| Cryptococcus | 2.34E-07 | -5.17018 | *** | 3.42E-06 | <0.0001 |
| Calosphaeria | 0.0156734 | -2.416432 | * | 0.0544652 | 0.0157 |
| Diplodia | 0.0293544 | -2.178694 | * | 0.0906755 | 0.0294 |
| Schizophyllum | 0.046163 | -1.9939 | * | 0.1166664 | 0.0462 |
| Auricularia | 0.0253715 | -2.235698 | * | 0.0820149 | 0.0254 |
| Issatchenkia | 0.0022107 | -3.060365 | ** | 0.0100749 | 0.0022 |
| Penicillium | 0.0200725 | -2.32499 | * | 0.0676212 | 0.0201 |
| Acremonium | 0.0001418 | -3.805052 | *** | 9.61E-04 | 0.0001 |
| Pleurotus | 0.0081873 | -2.644247 | ** | 0.0339712 | 0.0082 |
| Rozellomycota_gen_Incertae_sedis | 7.30E-08 | -5.383531 | *** | 1.35E-06 | <0.0001 |
| Ustilaginoidea | 4.87E-04 | -3.48778 | *** | 0.0030773 | 0.0005 |
| Mrakia | 5.60E-04 | -3.45051 | *** | 0.0034566 | 0.0006 |
| Diaporthe | 4.17E-08 | -5.483483 | *** | 9.66E-07 | <0.0001 |
| Tetracladium | 0.0014822 | -3.17814 | ** | 0.0072291 | 0.0015 |
| Paraphoma | 0.0011239 | -3.25751 | ** | 0.0058742 | 0.0011 |
| Leptospora | 1.91E-05 | -4.275443 | *** | 1.70E-04 | <0.0001 |
| Acrodontium | 0.0111912 | -2.536671 | * | 0.041482 | 0.0112 |

| Data S10. Comparison of gut mycobiome at the species level between three groups | | | |
| --- | --- | --- | --- |
| ID | CPR0.median | CPR0.mean | CPR0.se |
| Ascomycota_spe_Incertae_sedis | 0.0006(0.0003,0.0027) | 0.001602647 | 4.87E-04 |
| Zanclospora_jonesii | 0.0596(0.0264,0.1082) | 0.086623353 | 0.019845514 |
| Saccharomyces_cerevisiae | 0.0034(0.0002,0.0149) | 0.008514824 | 0.002924222 |
| Fungi_spe_Incertae_sedis | 0.0207(0.0049,0.0538) | 0.042711294 | 0.014212868 |
| Entorrhiza_citriformis | 0.008(0.0017,0.0234) | 0.056554588 | 0.028328732 |
| Dipodascaceae_spe_Incertae_sedis | 0.0539(0.0053,0.3745) | 0.221913412 | 0.062577334 |
| Hydnobolites_roseus | <0.0001(<0.0001,<0.0001) | 7.64E-04 | 6.92E-04 |
| Debaryomyces_prosopidis | 0.0034(0.0001,0.0308) | 0.015605059 | 0.005517021 |
| Talaromyces_scorteus | 0.0001(<0.0001,0.0002) | 1.56E-04 | 2.37E-05 |
| Candida_solani | 0.0248(0.0018,0.0895) | 0.073366412 | 0.029306878 |
| Dipodascus_geotrichum | 0.0012(0.0007,0.0247) | 0.095981588 | 0.048824741 |
| Heitmania_litseae | 0.0002(<0.0001,0.0004) | 5.55E-04 | 3.39E-04 |
| Trigonopsis_californica | <0.0001(<0.0001,<0.0001) | 1.25E-05 | 9.35E-06 |
| Dothiora_spartii | 0.0002(0.0001,0.0006) | 0.003143235 | 0.001701147 |
| Saccharomycetales_spe_Incertae_sedis | 0.0025(<0.0001,0.007) | 0.005992353 | 0.002508712 |
| Pseudeurotiaceae_spe_Incertae_sedis | <0.0001(<0.0001,<0.0001) | 0 | 0 |
| Rhodotorula_mucilaginosa | 0.0007(<0.0001,0.0013) | 0.041707647 | 0.041001542 |
| Iodosphaeria_phyllophila | <0.0001(<0.0001,<0.0001) | 0 | 0 |
| Candida_inconspicua | 0.0042(<0.0001,0.0126) | 0.018764941 | 0.00907285 |
| Milospium_graphideorum | <0.0001(<0.0001,<0.0001) | 8.18E-06 | 5.89E-06 |
| Arthrographis_grakistii | <0.0001(<0.0001,<0.0001) | 6.68E-05 | 2.86E-05 |
| Blumeria_spe_Incertae_sedis | 0.0004(0.0003,0.0006) | 5.05E-04 | 8.30E-05 |
| Mucor_circinelloides | 0.0127(0.0006,0.0242) | 0.024315588 | 0.009808528 |
| Actinomucor_elegans | 0.0005(<0.0001,0.0067) | 0.007108882 | 0.003928425 |
| Parafuscosporella_mucosa | 0.0011(0.0005,0.0015) | 0.003686706 | 0.00163938 |
| Candida_tropicalis | <0.0001(<0.0001,0.0011) | 9.31E-04 | 4.03E-04 |
| Eremothecium_coryli | <0.0001(<0.0001,<0.0001) | 5.65E-05 | 5.21E-05 |
| Angustimassarina_camporesii | <0.0001(<0.0001,0.0002) | 3.22E-04 | 1.91E-04 |
| Raffaelea_lauricola | 0.0005(0.0004,0.0008) | 5.76E-04 | 6.83E-05 |
| Ciliophora_spe_Incertae_sedis | <0.0001(<0.0001,<0.0001) | 0 | 0 |
| Alternaria_alternata | 0.0001(<0.0001,0.0003) | 0.002154 | 0.001418544 |
| Wallemia_spe_Incertae_sedis | 0.0001(<0.0001,0.0002) | 1.13E-04 | 2.67E-05 |
| Fantasmomyces_hyalinus | <0.0001(<0.0001,0.0005) | 8.36E-04 | 3.96E-04 |
| Apiotrichum_domesticum | <0.0001(<0.0001,<0.0001) | 4.62E-05 | 9.17E-06 |
| Yarrowia_deformans | <0.0001(<0.0001,<0.0001) | 0.003070176 | 0.002073954 |
| Tuber_alcaracense | <0.0001(<0.0001,<0.0001) | 1.55E-04 | 7.97E-05 |
| Cladosporium_kenpeggii | 0.0005(0.0002,0.0007) | 0.002471824 | 0.00132725 |
| Chrysosphaeria_jan-nelii | <0.0001(<0.0001,<0.0001) | 0 | 0 |
| Chytridium_olla | <0.0001(<0.0001,<0.0001) | 2.58E-05 | 2.58E-05 |
| Entyloma_calendulae | <0.0001(<0.0001,0.0002) | 0.006189824 | 0.004539238 |
| Calosphaeria_pulchella | 0.0001(<0.0001,0.0005) | 0.001372176 | 6.89E-04 |
| Diplodia_spe_Incertae_sedis | <0.0001(<0.0001,<0.0001) | 0 | 0 |
| Cutaneotrichosporon_smithiae | <0.0001(<0.0001,<0.0001) | 3.21E-05 | 3.21E-05 |
| Cryptococcus_longus | 0.0003(0.0002,0.0003) | 2.90E-04 | 3.65E-05 |
| Candida_vespimorsuum | <0.0001(<0.0001,<0.0001) | 4.24E-04 | 3.82E-04 |
| Schizophyllum_commune | <0.0001(<0.0001,<0.0001) | 3.29E-06 | 3.29E-06 |
| Auricularia_auricula-judae | <0.0001(<0.0001,<0.0001) | 0 | 0 |
| Issatchenkia_orientalis | <0.0001(<0.0001,0.0006) | 9.86E-04 | 6.91E-04 |
| Eremothecium_ashbyi | <0.0001(<0.0001,<0.0001) | 3.71E-05 | 3.71E-05 |
| Aspergillus_versicolor | <0.0001(<0.0001,<0.0001) | 0.002223059 | 0.002155154 |
| Aspergillus_amstelodami | <0.0001(<0.0001,<0.0001) | 4.04E-05 | 9.42E-06 |
| Penicillium_spe_Incertae_sedis | <0.0001(<0.0001,<0.0001) | 5.01E-05 | 2.02E-05 |
| Acremonium_cavaraeanum | <0.0001(<0.0001,<0.0001) | 0.002268176 | 0.00224825 |
| Wickerhamiella_pararugosa | <0.0001(<0.0001,<0.0001) | 0 | 0 |
| Candida_saitoana | <0.0001(<0.0001,<0.0001) | 7.06E-05 | 4.41E-05 |
| Rozellomycota_spe_Incertae_sedis | <0.0001(<0.0001,<0.0001) | 5.27E-05 | 1.41E-05 |
| Cladosporium_cladosporioides | <0.0001(<0.0001,<0.0001) | 1.86E-04 | 1.16E-04 |
| Ustilaginoidea_virens | <0.0001(<0.0001,<0.0001) | 0.001556824 | 0.001540443 |
| Pleurotus_ostreatus | <0.0001(<0.0001,<0.0001) | 3.15E-05 | 1.41E-05 |
| Mrakia_frigida | <0.0001(<0.0001,<0.0001) | 0.001392588 | 0.00138728 |
| Diaporthe_caulivora | <0.0001(<0.0001,<0.0001) | 0.001359765 | 0.001340848 |
| Tetracladium_spe_Incertae_sedis | <0.0001(<0.0001,<0.0001) | 0 | 0 |
| Paraphoma_spe_Incertae_sedis | <0.0001(<0.0001,<0.0001) | 0 | 0 |
| Cutaneotrichosporon_curvatus | <0.0001(<0.0001,<0.0001) | 0 | 0 |
| Leptospora_rubella | <0.0001(<0.0001,<0.0001) | 4.28E-05 | 1.63E-05 |
| Entyloma_linariae | <0.0001(<0.0001,<0.0001) | 7.48E-04 | 7.07E-04 |
| Acrodontium_crateriforme | <0.0001(<0.0001,<0.0001) | 0 | 0 |

| Data S10. Comparison of gut mycobiome at the species level between three groups | | | |
| --- | --- | --- | --- |
| ID | CPR1.median | CPR1.mean | CPR1.se |
| Ascomycota_spe_Incertae_sedis | 0.0002(<0.0001,0.0007) | 0.002546125 | 0.00142119 |
| Zanclospora_jonesii | 0.1042(0.0185,0.4387) | 0.242807125 | 0.054976187 |
| Saccharomyces_cerevisiae | 0.001(0.0003,0.1187) | 0.096199333 | 0.03486446 |
| Fungi_spe_Incertae_sedis | 0.0379(0.0162,0.0728) | 0.059496542 | 0.015053855 |
| Entorrhiza_citriformis | 0.0418(0.0177,0.1098) | 0.084154042 | 0.022976375 |
| Dipodascaceae_spe_Incertae_sedis | <0.0001(<0.0001,0.0003) | 0.008293458 | 0.007641268 |
| Hydnobolites_roseus | 0.0007(<0.0001,0.0369) | 0.027388833 | 0.010118923 |
| Debaryomyces_prosopidis | 0.0009(0.0002,0.0128) | 0.041530958 | 0.025784903 |
| Talaromyces_scorteus | <0.0001(<0.0001,0.0003) | 0.008029458 | 0.007047088 |
| Candida_solani | <0.0001(<0.0001,0.0001) | 0.017502667 | 0.017294601 |
| Dipodascus_geotrichum | <0.0001(<0.0001,<0.0001) | 0.002272083 | 0.001674651 |
| Heitmania_litseae | 0.0008(0.0002,0.0047) | 0.015636333 | 0.01052983 |
| Trigonopsis_californica | <0.0001(<0.0001,<0.0001) | 7.50E-06 | 7.50E-06 |
| Dothiora_spartii | 0.0067(0.0014,0.0307) | 0.015555667 | 0.004212284 |
| Saccharomycetales_spe_Incertae_sedis | <0.0001(<0.0001,0.0002) | 0.001170083 | 7.26E-04 |
| Pseudeurotiaceae_spe_Incertae_sedis | <0.0001(<0.0001,<0.0001) | 0 | 0 |
| Rhodotorula_mucilaginosa | <0.0001(<0.0001,<0.0001) | 3.16E-04 | 2.03E-04 |
| Iodosphaeria_phyllophila | <0.0001(<0.0001,0.0047) | 0.010973208 | 0.004615154 |
| Candida_inconspicua | <0.0001(<0.0001,<0.0001) | 3.19E-04 | 3.17E-04 |
| Milospium_graphideorum | <0.0001(<0.0001,0.0006) | 0.019479208 | 0.018788332 |
| Arthrographis_grakistii | <0.0001(<0.0001,<0.0001) | 4.13E-05 | 1.64E-05 |
| Blumeria_spe_Incertae_sedis | <0.0001(<0.0001,0.0004) | 0.008163333 | 0.006371913 |
| Mucor_circinelloides | <0.0001(<0.0001,<0.0001) | 3.46E-04 | 3.21E-04 |
| Actinomucor_elegans | <0.0001(<0.0001,<0.0001) | 0.002879167 | 0.002789442 |
| Parafuscosporella_mucosa | 0.0021(0.0009,0.0049) | 0.003501375 | 8.08E-04 |
| Candida_tropicalis | <0.0001(<0.0001,<0.0001) | 1.03E-05 | 6.08E-06 |
| Eremothecium_coryli | <0.0001(<0.0001,<0.0001) | 0.011704583 | 0.011683368 |
| Angustimassarina_camporesii | 0.0005(0.0001,0.0014) | 0.0012035 | 4.64E-04 |
| Raffaelea_lauricola | <0.0001(<0.0001,<0.0001) | 0.006910292 | 0.006652735 |
| Ciliophora_spe_Incertae_sedis | <0.0001(<0.0001,<0.0001) | 1.16E-05 | 1.16E-05 |
| Alternaria_alternata | <0.0001(<0.0001,0.0001) | 7.26E-04 | 3.30E-04 |
| Wallemia_spe_Incertae_sedis | <0.0001(<0.0001,<0.0001) | 1.05E-05 | 9.51E-06 |
| Fantasmomyces_hyalinus | 0.0007(0.0002,0.0026) | 0.002027292 | 6.64E-04 |
| Apiotrichum_domesticum | <0.0001(<0.0001,<0.0001) | 1.09E-04 | 1.09E-04 |
| Yarrowia_deformans | <0.0001(<0.0001,<0.0001) | 2.28E-05 | 2.28E-05 |
| Tuber_alcaracense | 0.0004(0.0001,0.0017) | 0.001033375 | 2.65E-04 |
| Cladosporium_kenpeggii | <0.0001(<0.0001,0.0006) | 0.001471208 | 9.97E-04 |
| Chrysosphaeria_jan-nelii | <0.0001(<0.0001,<0.0001) | 2.22E-04 | 1.43E-04 |
| Chytridium_olla | 0.0002(<0.0001,0.0026) | 0.0031195 | 0.001868019 |
| Entyloma_calendulae | <0.0001(<0.0001,<0.0001) | 1.67E-05 | 1.45E-05 |
| Calosphaeria_pulchella | 0.0005(0.0001,0.0013) | 9.11E-04 | 2.42E-04 |
| Diplodia_spe_Incertae_sedis | <0.0001(<0.0001,<0.0001) | 0 | 0 |
| Cutaneotrichosporon_smithiae | <0.0001(<0.0001,<0.0001) | 0.003606208 | 0.003599079 |
| Cryptococcus_longus | <0.0001(<0.0001,<0.0001) | 7.72E-05 | 7.72E-05 |
| Candida_vespimorsuum | <0.0001(<0.0001,<0.0001) | 2.66E-04 | 1.67E-04 |
| Schizophyllum_commune | <0.0001(<0.0001,<0.0001) | 0 | 0 |
| Auricularia_auricula-judae | <0.0001(<0.0001,<0.0001) | 1.57E-05 | 1.38E-05 |
| Issatchenkia_orientalis | <0.0001(<0.0001,<0.0001) | 0.001056417 | 0.001055678 |
| Eremothecium_ashbyi | <0.0001(<0.0001,<0.0001) | 0.002241917 | 0.00206701 |
| Aspergillus_versicolor | <0.0001(<0.0001,<0.0001) | 1.60E-04 | 1.60E-04 |
| Aspergillus_amstelodami | <0.0001(<0.0001,<0.0001) | 2.66E-04 | 1.35E-04 |
| Penicillium_spe_Incertae_sedis | <0.0001(<0.0001,<0.0001) | 1.55E-04 | 1.43E-04 |
| Acremonium_cavaraeanum | <0.0001(<0.0001,<0.0001) | 0 | 0 |
| Wickerhamiella_pararugosa | <0.0001(<0.0001,<0.0001) | 0 | 0 |
| Candida_saitoana | <0.0001(<0.0001,<0.0001) | 1.39E-05 | 1.05E-05 |
| Rozellomycota_spe_Incertae_sedis | <0.0001(<0.0001,<0.0001) | 0 | 0 |
| Cladosporium_cladosporioides | <0.0001(<0.0001,<0.0001) | 9.06E-04 | 7.40E-04 |
| Ustilaginoidea_virens | <0.0001(<0.0001,<0.0001) | 1.13E-06 | 1.13E-06 |
| Pleurotus_ostreatus | <0.0001(<0.0001,<0.0001) | 7.99E-04 | 5.10E-04 |
| Mrakia_frigida | <0.0001(<0.0001,<0.0001) | 3.25E-06 | 3.25E-06 |
| Diaporthe_caulivora | <0.0001(<0.0001,<0.0001) | 0 | 0 |
| Tetracladium_spe_Incertae_sedis | <0.0001(<0.0001,<0.0001) | 8.79E-04 | 8.73E-04 |
| Paraphoma_spe_Incertae_sedis | <0.0001(<0.0001,<0.0001) | 8.70E-04 | 7.92E-04 |
| Cutaneotrichosporon_curvatus | <0.0001(<0.0001,<0.0001) | 6.82E-04 | 6.79E-04 |
| Leptospora_rubella | <0.0001(<0.0001,<0.0001) | 0 | 0 |
| Entyloma_linariae | <0.0001(<0.0001,<0.0001) | 7.96E-06 | 7.96E-06 |
| Acrodontium_crateriforme | <0.0001(<0.0001,<0.0001) | 4.94E-04 | 4.92E-04 |

| Data S10. Comparison of gut mycobiome at the species level between three groups | | | |
| --- | --- | --- | --- |
| ID | HC.median | HC.mean | HC.se |
| Ascomycota_spe_Incertae_sedis | 0.2529(0.1987,0.3192) | 0.286155867 | 0.014734265 |
| Zanclospora_jonesii | 0.0211(0.004,0.0986) | 0.080584911 | 0.014053619 |
| Saccharomyces_cerevisiae | 0.0134(0.0007,0.1823) | 0.130607167 | 0.021927843 |
| Fungi_spe_Incertae_sedis | 0.0689(0.0475,0.097) | 0.080916211 | 0.004827277 |
| Entorrhiza_citriformis | 0.0044(0.0005,0.0451) | 0.043286411 | 0.007936388 |
| Dipodascaceae_spe_Incertae_sedis | <0.0001(<0.0001,<0.0001) | 7.24E-04 | 3.28E-04 |
| Hydnobolites_roseus | 0.0005(<0.0001,0.0054) | 0.029797167 | 0.00966847 |
| Debaryomyces_prosopidis | <0.0001(<0.0001,0.0004) | 0.011872856 | 0.008986662 |
| Talaromyces_scorteus | 0.0002(<0.0001,0.0008) | 0.019442956 | 0.009370493 |
| Candida_solani | <0.0001(<0.0001,<0.0001) | 0.001304778 | 6.67E-04 |
| Dipodascus_geotrichum | <0.0001(<0.0001,<0.0001) | 4.45E-05 | 1.62E-05 |
| Heitmania_litseae | 0.0008(<0.0001,0.0049) | 0.009285044 | 0.002763651 |
| Trigonopsis_californica | <0.0001(<0.0001,0.0003) | 0.0129861 | 0.007856359 |
| Dothiora_spartii | 0.0005(<0.0001,0.0056) | 0.007140344 | 0.001504987 |
| Saccharomycetales_spe_Incertae_sedis | <0.0001(<0.0001,<0.0001) | 0.008114011 | 0.003928305 |
| Pseudeurotiaceae_spe_Incertae_sedis | <0.0001(<0.0001,<0.0001) | 0.009337656 | 0.009279614 |
| Rhodotorula_mucilaginosa | <0.0001(<0.0001,<0.0001) | 1.35E-05 | 9.17E-06 |
| Iodosphaeria_phyllophila | <0.0001(<0.0001,0.0001) | 0.0044895 | 0.00196775 |
| Candida_inconspicua | <0.0001(<0.0001,<0.0001) | 0.003035656 | 0.002776744 |
| Milospium_graphideorum | <0.0001(<0.0001,0.0001) | 8.68E-04 | 4.49E-04 |
| Arthrographis_grakistii | <0.0001(<0.0001,0.0002) | 0.005839178 | 0.004169621 |
| Blumeria_spe_Incertae_sedis | <0.0001(<0.0001,0.0003) | 0.003523111 | 0.00212671 |
| Mucor_circinelloides | <0.0001(<0.0001,<0.0001) | 8.15E-05 | 5.09E-05 |
| Actinomucor_elegans | <0.0001(<0.0001,<0.0001) | 0.002279356 | 0.002127329 |
| Parafuscosporella_mucosa | 0.0008(<0.0001,0.0024) | 0.002192256 | 3.88E-04 |
| Candida_tropicalis | <0.0001(<0.0001,<0.0001) | 0.003378189 | 0.003350675 |
| Eremothecium_coryli | <0.0001(<0.0001,<0.0001) | 2.42E-05 | 2.39E-05 |
| Angustimassarina_camporesii | 0.0002(<0.0001,0.002) | 0.002507067 | 0.001172194 |
| Raffaelea_lauricola | <0.0001(<0.0001,<0.0001) | 7.09E-04 | 2.91E-04 |
| Ciliophora_spe_Incertae_sedis | <0.0001(<0.0001,<0.0001) | 0.002504467 | 0.002425395 |
| Alternaria_alternata | <0.0001(<0.0001,<0.0001) | 0.001572911 | 9.03E-04 |
| Wallemia_spe_Incertae_sedis | <0.0001(<0.0001,<0.0001) | 0.0019941 | 0.001410454 |
| Fantasmomyces_hyalinus | <0.0001(<0.0001,0.0008) | 0.001207211 | 2.94E-04 |
| Apiotrichum_domesticum | <0.0001(<0.0001,<0.0001) | 0.001772811 | 0.001739553 |
| Yarrowia_deformans | <0.0001(<0.0001,<0.0001) | 0.001083889 | 8.18E-04 |
| Tuber_alcaracense | <0.0001(<0.0001,0.0005) | 0.001290267 | 9.02E-04 |
| Cladosporium_kenpeggii | <0.0001(<0.0001,<0.0001) | 6.25E-04 | 2.53E-04 |
| Chrysosphaeria_jan-nelii | <0.0001(<0.0001,<0.0001) | 0.001306222 | 0.001169272 |
| Chytridium_olla | <0.0001(<0.0001,0.0001) | 4.00E-04 | 1.27E-04 |
| Entyloma_calendulae | <0.0001(<0.0001,<0.0001) | 2.70E-05 | 2.06E-05 |
| Calosphaeria_pulchella | <0.0001(<0.0001,0.0006) | 5.96E-04 | 1.57E-04 |
| Diplodia_spe_Incertae_sedis | <0.0001(<0.0001,<0.0001) | 0.0010858 | 0.001074575 |
| Cutaneotrichosporon_smithiae | <0.0001(<0.0001,<0.0001) | 1.07E-04 | 1.06E-04 |
| Cryptococcus_longus | <0.0001(<0.0001,<0.0001) | 9.86E-04 | 3.22E-04 |
| Candida_vespimorsuum | <0.0001(<0.0001,0.0002) | 8.76E-04 | 4.81E-04 |
| Schizophyllum_commune | <0.0001(<0.0001,<0.0001) | 8.23E-04 | 7.44E-04 |
| Auricularia_auricula-judae | <0.0001(<0.0001,<0.0001) | 6.87E-04 | 4.19E-04 |
| Issatchenkia_orientalis | <0.0001(<0.0001,<0.0001) | 2.20E-04 | 2.06E-04 |
| Eremothecium_ashbyi | <0.0001(<0.0001,<0.0001) | 0 | 0 |
| Aspergillus_versicolor | <0.0001(<0.0001,<0.0001) | 1.04E-04 | 8.06E-05 |
| Aspergillus_amstelodami | <0.0001(<0.0001,<0.0001) | 3.59E-04 | 1.86E-04 |
| Penicillium_spe_Incertae_sedis | <0.0001(<0.0001,<0.0001) | 3.82E-04 | 2.80E-04 |
| Acremonium_cavaraeanum | <0.0001(<0.0001,<0.0001) | 0 | 0 |
| Wickerhamiella_pararugosa | <0.0001(<0.0001,<0.0001) | 4.09E-04 | 3.29E-04 |
| Candida_saitoana | <0.0001(<0.0001,<0.0001) | 3.80E-04 | 3.75E-04 |
| Rozellomycota_spe_Incertae_sedis | <0.0001(<0.0001,<0.0001) | 3.66E-04 | 2.73E-04 |
| Cladosporium_cladosporioides | <0.0001(<0.0001,<0.0001) | 3.08E-05 | 1.32E-05 |
| Ustilaginoidea_virens | <0.0001(<0.0001,<0.0001) | 0 | 0 |
| Pleurotus_ostreatus | <0.0001(<0.0001,<0.0001) | 7.54E-05 | 4.13E-05 |
| Mrakia_frigida | <0.0001(<0.0001,<0.0001) | 0 | 0 |
| Diaporthe_caulivora | <0.0001(<0.0001,<0.0001) | 1.72E-06 | 1.72E-06 |
| Tetracladium_spe_Incertae_sedis | <0.0001(<0.0001,<0.0001) | 3.08E-06 | 3.08E-06 |
| Paraphoma_spe_Incertae_sedis | <0.0001(<0.0001,<0.0001) | 0 | 0 |
| Cutaneotrichosporon_curvatus | <0.0001(<0.0001,<0.0001) | 3.00E-07 | 3.00E-07 |
| Leptospora_rubella | <0.0001(<0.0001,<0.0001) | 1.54E-04 | 1.53E-04 |
| Entyloma_linariae | <0.0001(<0.0001,<0.0001) | 0 | 0 |
| Acrodontium_crateriforme | <0.0001(<0.0001,<0.0001) | 0 | 0 |

| Data S10. Comparison of gut mycobiome at the species level between three groups | | | | | |
| --- | --- | --- | --- | --- | --- |
| ID | p-value | z-score | Sig_mark | q-value | fixp |
| Ascomycota_spe_Incertae_sedis | 4.99E-19 | -8.91239 | *** | 2.14E-16 | <0.0001 |
| Zanclospora_jonesii | 0.0069369 | -2.699857 | ** | 0.0327026 | 0.0069 |
| Saccharomyces_cerevisiae | 0.0214462 | -2.300036 | * | 0.0766701 | 0.0214 |
| Fungi_spe_Incertae_sedis | 7.19E-06 | -4.487919 | *** | 7.71E-05 | <0.0001 |
| Entorrhiza_citriformis | 0.0159361 | -2.410375 | * | 0.0589362 | 0.0159 |
| Dipodascaceae_spe_Incertae_sedis | 3.38E-11 | -6.628878 | *** | 1.45E-09 | <0.0001 |
| Hydnobolites_roseus | 1.38E-05 | -4.346812 | *** | 1.45E-04 | <0.0001 |
| Debaryomyces_prosopidis | 1.48E-06 | -4.813751 | *** | 1.93E-05 | <0.0001 |
| Talaromyces_scorteus | 0.0157844 | -2.413862 | * | 0.0588828 | 0.0158 |
| Candida_solani | 7.22E-11 | -6.515942 | *** | 2.82E-09 | <0.0001 |
| Dipodascus_geotrichum | 2.52E-14 | -7.620733 | *** | 1.80E-12 | <0.0001 |
| Heitmania_litseae | 0.0454997 | -2.000005 | * | 0.1259314 | 0.0455 |
| Trigonopsis_californica | 1.18E-07 | -5.295865 | *** | 1.88E-06 | <0.0001 |
| Dothiora_spartii | 0.0024613 | -3.028054 | ** | 0.0125704 | 0.0025 |
| Saccharomycetales_spe_Incertae_sedis | 0.0071629 | -2.689175 | ** | 0.0334008 | 0.0072 |
| Pseudeurotiaceae_spe_Incertae_sedis | 0.0124319 | -2.499641 | * | 0.0476187 | 0.0124 |
| Rhodotorula_mucilaginosa | 3.88E-10 | -6.258894 | *** | 1.28E-08 | <0.0001 |
| Iodosphaeria_phyllophila | 1.96E-05 | -4.26938 | *** | 1.96E-04 | <0.0001 |
| Candida_inconspicua | 1.33E-09 | -6.063833 | *** | 3.17E-08 | <0.0001 |
| Milospium_graphideorum | 8.77E-04 | -3.327345 | *** | 0.0061662 | 0.0009 |
| Arthrographis_grakistii | 0.0276365 | -2.202408 | * | 0.0963906 | 0.0276 |
| Blumeria_spe_Incertae_sedis | 0.0012178 | -3.234687 | ** | 0.0069656 | 0.0012 |
| Mucor_circinelloides | 3.13E-17 | -8.44164 | *** | 4.47E-15 | <0.0001 |
| Actinomucor_elegans | 3.64E-06 | -4.630717 | *** | 4.11E-05 | <0.0001 |
| Parafuscosporella_mucosa | 0.0414823 | -2.038677 | * | 0.1163131 | 0.0415 |
| Candida_tropicalis | 0.0013665 | -3.201629 | ** | 0.0076135 | 0.0014 |
| Eremothecium_coryli | 3.67E-08 | -5.50595 | *** | 6.85E-07 | <0.0001 |
| Angustimassarina_camporesii | 0.0343783 | -2.115606 | * | 0.1000466 | 0.0344 |
| Raffaelea_lauricola | 2.77E-08 | -5.55551 | *** | 5.94E-07 | <0.0001 |
| Ciliophora_spe_Incertae_sedis | 0.0285863 | -2.189145 | * | 0.0983864 | 0.0286 |
| Alternaria_alternata | 0.0022045 | -3.061207 | ** | 0.0115656 | 0.0022 |
| Wallemia_spe_Incertae_sedis | 0.0015171 | -3.171383 | ** | 0.0082387 | 0.0015 |
| Fantasmomyces_hyalinus | 0.0144073 | -2.446945 | * | 0.0546967 | 0.0144 |
| Apiotrichum_domesticum | 2.12E-05 | -4.251629 | *** | 2.07E-04 | <0.0001 |
| Yarrowia_deformans | 0.0391556 | -2.062548 | * | 0.1105115 | 0.0392 |
| Tuber_alcaracense | 8.28E-04 | -3.343189 | *** | 0.0059217 | 0.0008 |
| Cladosporium_kenpeggii | 2.67E-06 | -4.694696 | *** | 3.18E-05 | <0.0001 |
| Chrysosphaeria_jan-nelii | 0.0201891 | -2.322816 | * | 0.0727824 | 0.0202 |
| Chytridium_olla | 0.0018104 | -3.119697 | ** | 0.0097081 | 0.0018 |
| Entyloma_calendulae | 1.55E-10 | -6.399922 | *** | 5.56E-09 | <0.0001 |
| Calosphaeria_pulchella | 0.0156734 | -2.416432 | * | 0.0588828 | 0.0157 |
| Diplodia_spe_Incertae_sedis | 0.0293544 | -2.178694 | * | 0.0983864 | 0.0294 |
| Cutaneotrichosporon_smithiae | 3.26E-04 | -3.593943 | *** | 0.0024952 | 0.0003 |
| Cryptococcus_longus | 2.30E-08 | -5.58742 | *** | 5.20E-07 | <0.0001 |
| Candida_vespimorsuum | 0.0483243 | -1.974506 | * | 0.1320453 | 0.0483 |
| Schizophyllum_commune | 0.046163 | -1.9939 | * | 0.1269481 | 0.0462 |
| Auricularia_auricula-judae | 0.0336822 | -2.123856 | * | 0.1000466 | 0.0337 |
| Issatchenkia_orientalis | 0.0022107 | -3.060365 | ** | 0.0115656 | 0.0022 |
| Eremothecium_ashbyi | 6.98E-04 | -3.39044 | *** | 0.0050739 | 0.0007 |
| Aspergillus_versicolor | 3.50E-05 | -4.138193 | *** | 3.08E-04 | <0.0001 |
| Aspergillus_amstelodami | 0.0022587 | -3.053924 | ** | 0.0116745 | 0.0023 |
| Penicillium_spe_Incertae_sedis | 0.0104945 | -2.559096 | * | 0.0436457 | 0.0105 |
| Acremonium_cavaraeanum | 2.11E-11 | -6.698246 | *** | 1.01E-09 | <0.0001 |
| Wickerhamiella_pararugosa | 0.0293554 | -2.178681 | * | 0.0983864 | 0.0294 |
| Candida_saitoana | 0.0339247 | -2.120966 | * | 0.1000466 | 0.0339 |
| Rozellomycota_spe_Incertae_sedis | 7.30E-08 | -5.383531 | *** | 1.25E-06 | <0.0001 |
| Cladosporium_cladosporioides | 0.0025033 | -3.022948 | ** | 0.0126341 | 0.0025 |
| Ustilaginoidea_virens | 4.87E-04 | -3.48778 | *** | 0.0036657 | 0.0005 |
| Pleurotus_ostreatus | 0.023412 | -2.266644 | * | 0.0830061 | 0.0234 |
| Mrakia_frigida | 5.60E-04 | -3.45051 | *** | 0.0041386 | 0.0006 |
| Diaporthe_caulivora | 4.17E-08 | -5.483483 | *** | 7.45E-07 | <0.0001 |
| Tetracladium_spe_Incertae_sedis | 0.0014822 | -3.17814 | ** | 0.0081523 | 0.0015 |
| Paraphoma_spe_Incertae_sedis | 0.0011239 | -3.25751 | ** | 0.0067374 | 0.0011 |
| Cutaneotrichosporon_curvatus | 0.011649 | -2.522603 | * | 0.0450218 | 0.0116 |
| Leptospora_rubella | 2.74E-06 | -4.689499 | *** | 3.18E-05 | <0.0001 |
| Entyloma_linariae | 8.36E-10 | -6.137888 | *** | 2.11E-08 | <0.0001 |
| Acrodontium_crateriforme | 0.0111912 | -2.536671 | * | 0.0436457 | 0.0112 |

| Data S11. Statistically significant LDA scores of oral mycobiome at different levels between the three groups | | | | |
| --- | --- | --- | --- | --- |
| Biomaker_names | Logarithm value | Groups | LDA_value | P_value |
| k__Fungi | 5.705672357 | CPR0 | 5.27690536 | 1.64E-09 |
| k__Fungi.p__Ascomycota.c__Dothideomycetes.o__Botryosphaeriales.f__Botryosphaeriaceae.g__Botryosphaeria | 3.787170284 | CPR0 | 3.554469317 | 2.94E-09 |
| p__Ascomycota.c__Sordariomycetes.o__Sordariomycetes_ord_Incertae_sedis.f__Sordariomycetes_fam_Incertae_sedis.g__Acrodictys | 4.421592004 | HC | 4.003618278 | 0.00065413 |
| p__Ascomycota.c__Dothideomycetes.o__Eremomycetales.f__Eremomycetaceae.g__Arthrographis.s__Arthrographis_grakistii | 3.794461978 | HC | 3.527358764 | 2.06E-10 |
| p__Ascomycota.c__Sordariomycetes.o__Ophiostomatales.f__Ophiostomataceae | 3.944401132 | HC | 3.641085816 | 1.53E-13 |
| p__Mucoromycota.c__Glomeromycetes | 3.884064676 | HC | 3.587312404 | 1.43E-09 |
| k__Fungi.p__Ascomycota.c__Dothideomycetes.o__Pleosporales.f__Pleosporaceae.g__Alternaria.s__Alternaria_alternata | 3.946171041 | CPR0 | 3.598837669 | 1.23E-06 |
| k__Fungi.p__Basidiomycota.c__Malasseziomycetes.o__Malasseziales.f__Malasseziaceae.g__Malassezia.s__Malassezia_restricta | 4.879488791 | CPR1 | 4.485597162 | 0.000105363 |
| k__Fungi.p__Basidiomycota.c__Exobasidiomycetes | 3.115954637 | CPR0 | 3.070651174 | 3.85E-07 |
| k__Fungi.p__Ascomycota.c__Leotiomycetes | 4.263483963 | HC | 3.919131169 | 5.41E-10 |
| p__Mucoromycota.c__Glomeromycetes.o__Diversisporales.f__Diversisporaceae.g__Diversispora | 3.883993496 | HC | 3.605184989 | 6.07E-10 |
| p__Ascomycota.c__Eurotiomycetes.o__Eurotiales.f__Trichocomaceae.g__Talaromyces | 3.581045139 | HC | 3.172655002 | 7.53E-08 |
| k__Fungi.p__Basidiomycota.c__Tremellomycetes.o__Cystofilobasidiales.f__Mrakiaceae | 4.673070058 | CPR0 | 4.408329269 | 1.13E-05 |
| p__Ascomycota.c__Eurotiomycetes.o__Eurotiales.f__Trichocomaceae | 3.581045139 | HC | 3.163679802 | 7.53E-08 |
| k__Fungi.p__Ascomycota.c__Leotiomycetes.o__Erysiphales.f__Erysiphaceae.g__Blumeria.s__Blumeria_spe_Incertae_sedis | 4.24070532 | HC | 3.905448215 | 2.66E-15 |
| k__Fungi.p__Ascomycota.c__Eurotiomycetes.o__Eurotiales | 4.496432562 | CPR1 | 4.084875227 | 0.002463901 |
| k__Fungi.p__Basidiomycota.c__Microbotryomycetes.o__Sporidiobolales | 4.342660572 | CPR0 | 4.042276023 | 2.72E-08 |
| p__Ascomycota.c__Sordariomycetes.o__Chaetosphaeriales.f__Chaetosphaeriaceae | 4.775913955 | CPR0 | 4.209104622 | 0.000168369 |
| p__Ascomycota.c__Sordariomycetes.o__Ophiostomatales.f__Ophiostomataceae.g__Chrysosphaeria.s__Chrysosphaeria_jan_nelii | 2.831329092 | HC | 3.031454374 | 3.68E-07 |
| k__Fungi.p__Basidiomycota.c__Tremellomycetes.o__Cystofilobasidiales | 4.673130358 | CPR0 | 4.404731511 | 2.55E-05 |
| p__Mucoromycota.c__Mucoromycetes.o__Mucorales.f__Mucoraceae.g__Mucor | 2.559584224 | CPR0 | 3.392272527 | 1.65E-18 |
| p__Ascomycota.c__Sordariomycetes.o__Calosphaeriales.f__Calosphaeriaceae | 3.036192366 | HC | 3.235276893 | 3.76E-09 |
| k__Fungi.p__Basidiomycota.c__Agaricomycetes.o__Agaricales.f__Lycoperdaceae.g__Bovista | 2.632176359 | HC | 3.070718892 | 1.50E-07 |
| p__Fungi_phy_Incertae_sedis.c__Entorrhizomycetes.o__Entorrhizales | 4.170637316 | HC | 3.827443743 | 2.81E-05 |
| k__Fungi.p__Mucoromycota.c__Mucoromycetes.o__Mucorales.f__Mucoraceae.g__Mucor.s__Mucor_circinelloides | 3.851672383 | CPR0 | 3.599754279 | 4.22E-12 |
| k__Fungi.p__Basidiomycota.c__Agaricomycetes.o__Agaricales.f__Lycoperdaceae | 2.632176359 | HC | 3.065669541 | 1.50E-07 |
| k__Fungi.p__Ascomycota.c__Eurotiomycetes | 4.608060257 | CPR1 | 4.131248073 | 0.000991713 |
| k__Fungi.p__Ascomycota.c__Dothideomycetes.o__Botryosphaeriales.f__Botryosphaeriaceae.g__Botryosphaeria.s__Botryosphaeria_spe_Incertae_sedis | 3.787170284 | CPR0 | 3.543434163 | 2.94E-09 |
| p__Fungi_phy_Incertae_sedis.c__Entorrhizomycetes.o__Entorrhizales.f__Entorrhizaceae.g__Entorrhiza | 4.170637316 | HC | 3.827443743 | 2.81E-05 |
| k__Fungi.p__Basidiomycota.c__Tremellomycetes.o__Cystofilobasidiales.f__Mrakiaceae.g__Udeniomyces | 4.667597007 | CPR0 | 4.404969498 | 1.10E-05 |
| k__Fungi.p__Mucoromycota.c__Mucoromycetes.o__Mucorales.f__Mucoraceae | 5.368007015 | CPR0 | 5.059553607 | 7.64E-16 |
| p__Basidiomycota.c__Microbotryomycetes.o__Microbotryomycetes_ord_Incertae_sedis.f__Microbotryomycetes_fam_Incertae_sedis | 3.660201888 | HC | 3.388583091 | 9.79E-12 |
| p__Fungi_phy_Incertae_sedis | 4.86960732 | HC | 4.525330274 | 2.81E-05 |
| k__Fungi.p__Fungi_phy_Incertae_sedis.c__Fungi_cla_Incertae_sedis.o__Fungi_ord_Incertae_sedis.f__Fungi_fam_Incertae_sedis | 4.917638516 | CPR1 | 4.586282771 | 4.00E-11 |
| k__Fungi.p__Ascomycota.c__Saccharomycetes.o__Saccharomycetales.f__Dipodascaceae.g__Dipodascus.s__Dipodascus_geotrichum | 2.827285619 | CPR0 | 3.640016852 | 0.003966775 |
| k__Fungi.p__Ascomycota.c__Sordariomycetes.o__Trichosphaeriales.f__Trichosphaeriaceae.g__Nigrospora | 3.225659816 | CPR1 | 3.044742411 | 0.0022237 |
| k__Fungi.p__Ascomycota.c__Leotiomycetes.o__Erysiphales.f__Erysiphaceae | 4.243747103 | HC | 3.906133101 | 7.66E-12 |
| k__Fungi.p__Ascomycota.c__Leotiomycetes.o__Erysiphales.f__Erysiphaceae.g__Blumeria | 4.242329328 | HC | 3.905199255 | 2.66E-15 |
| k__Fungi.p__Ascomycota.c__Eurotiomycetes.o__Eurotiales.f__Aspergillaceae.g__Aspergillus.s__Aspergillus_penicillioides | 4.156669675 | CPR1 | 3.793015685 | 2.30E-05 |
| p__Mucoromycota.c__Mucoromycetes.o__Mucorales | 2.559584224 | CPR0 | 3.391523302 | 1.65E-18 |
| p__Ascomycota.c__Dothideomycetes.o__Dothideales | 3.762075843 | CPR1 | 3.41101946 | 3.08E-05 |
| k__Fungi.p__Ascomycota.c__Saccharomycetes.o__Saccharomycetales.f__Dipodascaceae.g__Dipodascus | 2.828260104 | CPR0 | 3.616100412 | 0.003817712 |
| p__Ascomycota.c__Ascomycota_cla_Incertae_sedis.o__Ascomycota_ord_Incertae_sedis.f__Ascomycota_fam_Incertae_sedis.g__Milospium | 3.705942661 | CPR1 | 3.300235132 | 0.000265705 |
| p__Ascomycota.c__Ascomycota_cla_Incertae_sedis.o__Ascomycota_ord_Incertae_sedis | 3.968652101 | HC | 3.418439318 | 0.000965006 |
| k__Fungi.p__Basidiomycota.c__Microbotryomycetes.o__Sporidiobolales.f__Sporidiobolaceae | 4.342660572 | CPR0 | 4.042962126 | 2.72E-08 |
| p__Ascomycota.c__Eurotiomycetes.o__Eurotiales.f__Trichocomaceae.g__Talaromyces.s__Talaromyces_scorteus | 3.41785878 | HC | 3.168074868 | 2.43E-05 |
| p__Basidiomycota | 4.398275988 | CPR1 | 4.101721105 | 2.88E-07 |
| p__Ascomycota.c__Dothideomycetes.o__Eremomycetales.f__Eremomycetaceae.g__Arthrographis | 3.794461978 | HC | 3.524227317 | 2.06E-10 |
| p__Ascomycota.c__Dothideomycetes.o__Pleosporales | 3.087209962 | HC | 3.113621109 | 0.001101292 |
| k__Fungi.p__Ascomycota.c__Dothideomycetes.o__Botryosphaeriales | 3.787170284 | CPR0 | 3.580737249 | 5.04E-08 |
| p__Ascomycota.c__Ascomycota_cla_Incertae_sedis.o__Ascomycota_ord_Incertae_sedis.f__Ascomycota_fam_Incertae_sedis | 3.968652101 | HC | 3.418439318 | 0.000965006 |
| k__Fungi.p__Basidiomycota.c__Malasseziomycetes.o__Malasseziales | 4.974479245 | CPR1 | 4.579606094 | 1.52E-06 |
| k__Fungi.p__Ascomycota.c__Ascomycota_cla_Incertae_sedis.o__Ascomycota_ord_Incertae_sedis.f__Ascomycota_fam_Incertae_sedis.g__Ascomycota_gen_Incertae_sedis | 3.799331948 | CPR0 | 3.347491038 | 2.60E-06 |
| k__Fungi.p__Fungi_phy_Incertae_sedis.c__Fungi_cla_Incertae_sedis.o__Fungi_ord_Incertae_sedis | 4.917638516 | CPR1 | 4.586282771 | 4.00E-11 |
| k__Fungi.p__Fungi_phy_Incertae_sedis | 4.98612047 | CPR1 | 4.649954795 | 2.08E-10 |
| k__Fungi.p__Ascomycota.c__Sordariomycetes.o__Hypocreales.f__Hypocreales_fam_Incertae_sedis.g__Ustilaginoidea.s__Ustilaginoidea_virens | 3.317557437 | CPR0 | 3.359997017 | 2.25E-07 |
| p__Ascomycota.c__Ascomycota_cla_Incertae_sedis.o__Ascomycota_ord_Incertae_sedis.f__Ascomycota_fam_Incertae_sedis.g__Milospium.s__Milospium_graphideorum | 3.705942661 | CPR1 | 3.300023203 | 0.000265705 |
| k__Fungi.p__Basidiomycota.c__Tremellomycetes.o__Tremellales | 4.15684846 | HC | 3.845845689 | 2.88E-16 |
| k__Fungi.p__Ascomycota.c__Sordariomycetes.o__Trichosphaeriales | 3.225659816 | CPR1 | 3.095958583 | 0.0022237 |
| k__Fungi.p__Ascomycota.c__Dothideomycetes.o__Pleosporales.f__Pleosporaceae.g__Alternaria | 3.948710907 | CPR0 | 3.596770579 | 1.23E-06 |
| p__Ascomycota.c__Sordariomycetes.o__Chaetosphaeriales.f__Chaetosphaeriaceae.g__Zanclospora.s__Zanclospora_jonesii | 4.775510034 | CPR0 | 4.208522376 | 0.000168369 |
| k__Fungi.p__Fungi_phy_Incertae_sedis.c__Fungi_cla_Incertae_sedis | 4.917638516 | CPR1 | 4.586282771 | 4.00E-11 |
| k__Fungi.p__Basidiomycota.c__Malasseziomycetes.o__Malasseziales.f__Malasseziaceae.g__Malassezia | 4.969204143 | CPR1 | 4.577525582 | 4.33E-06 |
| k__Fungi.p__Ascomycota.c__Dothideomycetes.o__Pleosporales.f__Pleosporaceae | 3.948710907 | CPR0 | 3.603615516 | 1.06E-06 |
| p__Mucoromycota | 4.58303468 | HC | 4.270378827 | 5.09E-09 |
| k__Fungi.p__Ascomycota.c__Eurotiomycetes.o__Eurotiales.f__Aspergillaceae.g__Aspergillus.s__Aspergillus_versicolor | 3.222631704 | CPR0 | 3.208656257 | 0.001622063 |
| p__Ascomycota | 5.863516748 | HC | 5.102711314 | 4.36E-06 |
| p__Ascomycota.c__Sordariomycetes.o__Hypocreales.f__Cordycipitaceae | 3.307328538 | CPR1 | 3.120821772 | 0.00617933 |
| p__Ascomycota.c__Sordariomycetes.o__Fuscosporellales.f__Fuscosporellaceae.g__Parafuscosporella.s__Parafuscosporella_mucosa | 3.250521169 | HC | 3.001771119 | 1.76E-08 |
| k__Fungi.p__Ascomycota.c__Leotiomycetes.o__Helotiales | 3.570244611 | CPR1 | 3.317565499 | 0.001682276 |
| p__Basidiomycota.c__Microbotryomycetes | 4.26226188 | HC | 3.974345378 | 9.79E-12 |
| k__Fungi.p__Ascomycota.c__Dothideomycetes.o__Pleosporales.f__Phaeosphaeriaceae | 3.583762405 | HC | 3.312059452 | 0.002446376 |
| p__Mucoromycota.c__Glomeromycetes.o__Diversisporales.f__Diversisporaceae.g__Diversispora.s__Diversispora_spurca | 3.883993496 | HC | 3.605796366 | 6.07E-10 |
| k__Fungi.p__Ascomycota.c__Ascomycota_cla_Incertae_sedis.o__Ascomycota_ord_Incertae_sedis | 4.100361943 | CPR0 | 3.64206197 | 2.60E-06 |
| k__Fungi.p__Mucoromycota.c__Mucoromycetes | 5.368013947 | CPR0 | 5.059473967 | 1.55E-14 |
| k__Fungi.p__Mucoromycota.c__Mucoromycetes.o__Mucorales.f__Mucoraceae.g__Actinomucor.s__Actinomucor_elegans | 5.353269638 | CPR0 | 5.044983673 | 1.46E-17 |
| k__Fungi.p__Ascomycota.c__Ascomycota_cla_Incertae_sedis.o__Ascomycota_ord_Incertae_sedis.f__Ascomycota_fam_Incertae_sedis | 4.100361943 | CPR0 | 3.64206197 | 2.60E-06 |
| p__Ascomycota.c__Sordariomycetes.o__Chaetosphaeriales | 4.792849041 | CPR0 | 4.233429782 | 0.000144292 |
| k__Fungi.p__Basidiomycota.c__Microbotryomycetes | 4.34282422 | CPR0 | 3.967579374 | 2.62E-05 |
| k__Fungi.p__Ascomycota.c__Sordariomycetes.o__Trichosphaeriales.f__Trichosphaeriaceae | 3.225659816 | CPR1 | 3.046961846 | 0.0022237 |
| p__Ascomycota.c__Sordariomycetes.o__Chaetosphaeriales.f__Chaetosphaeriaceae.g__Zanclospora.s__Zanclospora_aurea | 1.744224342 | CPR0 | 4.182693674 | 0.001414446 |
| k__Fungi.p__Ascomycota.c__Eurotiomycetes.o__Eurotiales.f__Aspergillaceae.g__Aspergillus.s__Aspergillus_amstelodami | 3.565610983 | CPR1 | 3.307541886 | 7.09E-05 |
| k__Fungi.p__Basidiomycota.c__Agaricomycetes.o__Agaricales.f__Psathyrellaceae | 2.876294577 | HC | 3.092353537 | 9.90E-08 |
| k__Fungi.p__Ascomycota.c__Sordariomycetes.o__Hypocreales.f__Nectriaceae.g__Fusarium | 3.352515226 | CPR1 | 3.137426832 | 0.030005038 |
| k__Fungi.p__Ascomycota.c__Sordariomycetes | 4.710203322 | CPR1 | 4.203316434 | 0.004797381 |
| p__Ascomycota.c__Sordariomycetes.o__Chaetosphaeriales.f__Chaetosphaeriaceae.g__Zanclospora | 4.775913955 | CPR0 | 4.209104622 | 0.000168369 |
| p__Ascomycota.c__Sordariomycetes.o__Ophiostomatales.f__Ophiostomataceae.g__Ophiostoma.s__Ophiostoma_acarorum | 2.646329043 | HC | 3.185646315 | 6.67E-07 |
| p__Mucoromycota.c__Mucoromycetes | 2.559584224 | CPR0 | 3.384457669 | 1.65E-18 |
| p__Ascomycota.c__Sordariomycetes.o__Sordariomycetes_ord_Incertae_sedis.f__Sordariomycetes_fam_Incertae_sedis.g__Acrodictys.s__Acrodictys_fluminicola | 4.421592004 | HC | 4.003618278 | 0.00065413 |
| p__Fungi_phy_Incertae_sedis.c__Entorrhizomycetes.o__Entorrhizales.f__Entorrhizaceae | 4.170637316 | HC | 3.827443743 | 2.81E-05 |
| p__Ascomycota.c__Sordariomycetes.o__Hypocreales | 3.722849168 | CPR1 | 3.348782441 | 0.003110656 |
| k__Fungi.p__Mucoromycota.c__Mucoromycetes.o__Mucorales.f__Mucoraceae.g__Actinomucor | 5.353269638 | CPR0 | 5.044983654 | 1.46E-17 |
| k__Fungi.p__Ascomycota.c__Sordariomycetes.o__Hypocreales.f__Hypocreales_fam_Incertae_sedis.g__Ustilaginoidea | 3.317557437 | CPR0 | 3.359932206 | 2.25E-07 |
| p__Ascomycota.c__Sordariomycetes.o__Ophiostomatales.f__Ophiostomataceae.g__Chrysosphaeria | 2.831329092 | HC | 3.022764066 | 3.68E-07 |
| k__Fungi.p__Ascomycota.c__Saccharomycetes.o__Saccharomycetales.f__Dipodascaceae.g__Dipodascaceae_gen_Incertae_sedis.s__Dipodascaceae_spe_Incertae_sedis | 3.990945097 | CPR1 | 3.703166548 | 7.03E-08 |
| k__Fungi.p__Mucoromycota | 5.369499226 | CPR0 | 5.058178987 | 1.65E-11 |
| p__Mucoromycota.c__Mucoromycetes.o__Mucorales.f__Mucoraceae.g__Mucor.s__Mucor_pseudolusitanicus | 2.559584224 | CPR0 | 3.38674262 | 1.65E-18 |
| k__Fungi.p__Basidiomycota.c__Microbotryomycetes.o__Sporidiobolales.f__Sporidiobolaceae.g__Rhodotorula | 4.234028203 | CPR0 | 3.932196797 | 2.77E-12 |
| k__Fungi.p__Ascomycota.c__Saccharomycetes.o__Saccharomycetales | 4.98626805 | CPR0 | 4.626465519 | 0.001110749 |
| k__Fungi.p__Ascomycota.c__Saccharomycetes | 4.98626805 | CPR0 | 4.626465519 | 0.001110749 |
| k__Fungi.p__Basidiomycota.c__Tremellomycetes.o__Trichosporonales.f__Trichosporonaceae | 3.179795455 | CPR1 | 3.00370797 | 0.000217855 |
| p__Ascomycota.c__Sordariomycetes.o__Fuscosporellales.f__Fuscosporellaceae.g__Parafuscosporella | 3.250521169 | HC | 3.080517122 | 1.76E-08 |
| k__Fungi.p__Ascomycota.c__Eurotiomycetes.o__Eurotiales.f__Aspergillaceae | 4.479560176 | CPR1 | 4.086283244 | 0.000692849 |
| k__Fungi.p__Ascomycota.c__Sordariomycetes.o__Trichosphaeriales.f__Trichosphaeriaceae.g__Nigrospora.s__Nigrospora_oryzae | 3.225659816 | CPR1 | 3.05006699 | 0.00064497 |
| k__Fungi.p__Ascomycota.c__Dothideomycetes.o__Dothideales | 3.791615213 | CPR1 | 3.437979675 | 0.000236617 |
| p__Ascomycota.c__Sordariomycetes.o__Ophiostomatales.f__Ophiostomataceae.g__Ophiostoma | 2.646473956 | HC | 3.207356936 | 1.23E-05 |
| k__Fungi.p__Basidiomycota.c__Agaricomycetes.o__Polyporales | 3.340577137 | CPR1 | 3.003252581 | 9.57E-05 |
| k__Fungi.p__Ascomycota.c__Sordariomycetes.o__Diaporthales | 3.255881406 | HC | 3.139479735 | 7.89E-07 |
| p__Basidiomycota.c__Microbotryomycetes.o__Microbotryomycetes_ord_Incertae_sedis.f__Microbotryomycetes_fam_Incertae_sedis.g__Heitmania | 3.660201888 | HC | 3.382618234 | 9.79E-12 |
| p__Ascomycota.c__Sordariomycetes.o__Sordariomycetes_ord_Incertae_sedis | 4.425956436 | HC | 4.00727649 | 0.000892217 |
| k__Fungi.p__Ascomycota.c__Saccharomycetes.o__Saccharomycetales.f__Saccharomycetaceae | 3.398100247 | CPR0 | 3.213071696 | 0.008529486 |
| p__Basidiomycota.c__Agaricomycetes | 2.907448869 | HC | 3.147157486 | 1.10E-05 |
| k__Fungi.p__Basidiomycota.c__Tremellomycetes | 4.753785679 | CPR0 | 4.354146534 | 0.022789885 |
| p__Ascomycota.c__Dothideomycetes.o__Eremomycetales | 3.833682752 | HC | 3.535458568 | 1.78E-16 |
| k__Fungi.p__Ascomycota.c__Saccharomycetes.o__Saccharomycetales.f__Saccharomycetales_fam_Incertae_sedis.g__Candida | 4.754660897 | CPR0 | 4.489488382 | 0.046868952 |
| k__Fungi.p__Ascomycota.c__Dothideomycetes.o__Capnodiales.f__Cladosporiaceae.g__Cladosporium.s__Cladosporium_halotolerans | 3.481407672 | CPR0 | 3.108599653 | 2.85E-05 |
| p__Mucoromycota.c__Glomeromycetes.o__Diversisporales | 3.883993496 | HC | 3.606302275 | 6.07E-10 |
| p__Ascomycota.c__Ascomycota_cla_Incertae_sedis | 3.968652101 | HC | 3.418439318 | 0.000965006 |
| p__Basidiomycota.c__Microbotryomycetes.o__Microbotryomycetes_ord_Incertae_sedis | 3.660201888 | HC | 3.38333602 | 9.79E-12 |
| p__Fungi_phy_Incertae_sedis.c__Entorrhizomycetes.o__Entorrhizales.f__Entorrhizaceae.g__Entorrhiza.s__Entorrhiza_citriformis | 4.169955858 | HC | 3.828377023 | 1.83E-05 |
| k__Fungi.p__Basidiomycota.c__Tremellomycetes.o__Tremellales.f__Tremellaceae.g__Cryptococcus | 4.129206623 | HC | 3.839935906 | 3.96E-22 |
| p__Ascomycota.c__Sordariomycetes.o__Fuscosporellales.f__Fuscosporellaceae | 3.250521169 | HC | 3.089364488 | 1.76E-08 |
| k__Fungi.p__Ascomycota.c__Ascomycota_cla_Incertae_sedis.o__Ascomycota_ord_Incertae_sedis.f__Ascomycota_fam_Incertae_sedis.g__Ascomycota_gen_Incertae_sedis.s__Ascomycota_spe_Incertae_sedis | 3.799331948 | CPR0 | 3.347491038 | 2.60E-06 |
| p__Ascomycota.c__Dothideomycetes | 4.531059402 | HC | 4.150101422 | 7.55E-06 |
| p__Mucoromycota.c__Glomeromycetes.o__Diversisporales.f__Diversisporaceae | 3.883993496 | HC | 3.60586827 | 6.07E-10 |
| k__Fungi.p__Basidiomycota.c__Tremellomycetes.o__Cystofilobasidiales.f__Mrakiaceae.g__Udeniomyces.s__Udeniomyces_pyricola | 4.312302704 | CPR0 | 4.066714592 | 1.64E-12 |
| p__Ascomycota.c__Sordariomycetes.o__Ophiostomatales | 3.944401132 | HC | 3.641894466 | 1.53E-13 |
| k__Fungi.p__Basidiomycota.c__Tremellomycetes.o__Tremellales.f__Tremellaceae | 4.129235209 | HC | 3.839988265 | 4.72E-22 |
| k__Fungi.p__Ascomycota.c__Leotiomycetes.o__Erysiphales | 4.243747103 | HC | 3.906178432 | 7.66E-12 |
| p__Fungi_phy_Incertae_sedis.c__Entorrhizomycetes | 4.170637316 | HC | 3.827443743 | 2.81E-05 |
| p__Ascomycota.c__Sordariomycetes.o__Hypocreales.f__Cordycipitaceae.g__Simplicillium.s__Simplicillium_sympodiophorum | 3.201437292 | CPR1 | 3.198888628 | 4.45E-06 |
| p__Ascomycota.c__Sordariomycetes.o__Calosphaeriales.f__Calosphaeriaceae.g__Calosphaeria.s__Calosphaeria_pulchella | 2.804599253 | HC | 3.173679146 | 2.98E-07 |
| p__Ascomycota.c__Sordariomycetes.o__Ophiostomatales.f__Ophiostomataceae.g__Raffaelea | 3.885186079 | HC | 3.585570319 | 2.00E-10 |
| p__Ascomycota.c__Sordariomycetes.o__Calosphaeriales.f__Calosphaeriaceae.g__Calosphaeria | 2.804599253 | HC | 3.166803714 | 2.98E-07 |
| k__Fungi.p__Basidiomycota.c__Tremellomycetes.o__Tremellales.f__Tremellaceae.g__Cryptococcus.s__Cryptococcus_longus | 4.129206623 | HC | 3.839935625 | 3.96E-22 |
| k__Fungi.p__Mucoromycota.c__Mucoromycetes.o__Mucorales.f__Mucoraceae.g__Mucor | 3.891295058 | CPR0 | 3.628270996 | 9.96E-13 |
| k__Fungi.p__Basidiomycota.c__Malasseziomycetes | 4.974479245 | CPR1 | 4.579606094 | 1.52E-06 |
| p__Ascomycota.c__Sordariomycetes.o__Ophiostomatales.f__Ophiostomataceae.g__Raffaelea.s__Raffaelea_lauricola | 3.882744712 | HC | 3.585094251 | 4.55E-09 |
| k__Fungi.p__Ascomycota.c__Eurotiomycetes.o__Eurotiales.f__Aspergillaceae.g__Aspergillus | 4.439052555 | CPR1 | 4.066331744 | 0.000352635 |
| k__Fungi.p__Ascomycota.c__Saccharomycetes.o__Saccharomycetales.f__Dipodascaceae | 4.016098313 | CPR1 | 3.729889728 | 1.35E-05 |
| p__Ascomycota.c__Sordariomycetes.o__Hypocreales.f__Cordycipitaceae.g__Simplicillium | 3.202745921 | CPR1 | 3.120098486 | 0.000126828 |
| k__Fungi.p__Ascomycota.c__Saccharomycetes.o__Saccharomycetales.f__Saccharomycetales_fam_Incertae_sedis.g__Candida.s__Candida_albicans | 4.744092848 | CPR0 | 4.439558548 | 1.80E-07 |
| k__Fungi.p__Basidiomycota.c__Malasseziomycetes.o__Malasseziales.f__Malasseziaceae | 4.974479245 | CPR1 | 4.579606094 | 1.52E-06 |
| p__Ascomycota.c__Dothideomycetes.o__Eremomycetales.f__Eremomycetaceae | 3.833682752 | HC | 3.534452966 | 1.78E-16 |
| k__Fungi.p__Fungi_phy_Incertae_sedis.c__Fungi_cla_Incertae_sedis.o__Fungi_ord_Incertae_sedis.f__Fungi_fam_Incertae_sedis.g__Fungi_gen_Incertae_sedis | 4.917638516 | CPR1 | 4.586282771 | 4.00E-11 |
| k__Fungi.p__Ascomycota.c__Sordariomycetes.o__Hypocreales.f__Hypocreales_fam_Incertae_sedis | 3.345138186 | CPR0 | 3.33897181 | 9.80E-05 |
| p__Ascomycota.c__Sordariomycetes.o__Sordariomycetes_ord_Incertae_sedis.f__Sordariomycetes_fam_Incertae_sedis | 4.425956436 | HC | 4.00727649 | 0.000892217 |
| k__Fungi.p__Mucoromycota.c__Mucoromycetes.o__Mucorales | 5.368013947 | CPR0 | 5.059479978 | 1.55E-14 |
| k__Fungi.p__Ascomycota.c__Saccharomycetes.o__Saccharomycetales.f__Saccharomycetales_fam_Incertae_sedis.g__Candida.s__Candida_parapsilosis | 3.251901925 | CPR1 | 3.036474538 | 0.003423166 |
| p__Ascomycota.c__Sordariomycetes.o__Fuscosporellales | 3.250521169 | HC | 3.177361019 | 1.76E-08 |
| p__Ascomycota.c__Eurotiomycetes.o__Eurotiales.f__Aspergillaceae.g__Aspergillus.s__Aspergillus_subflavus | 4.406234253 | HC | 4.05754914 | 1.57E-05 |
| k__Fungi.p__Basidiomycota.c__Microbotryomycetes.o__Sporidiobolales.f__Sporidiobolaceae.g__Rhodotorula.s__Rhodotorula_mucilaginosa | 4.233291641 | CPR0 | 3.933002106 | 2.69E-13 |
| k__Fungi.p__Ascomycota.c__Ascomycota_cla_Incertae_sedis | 4.100361943 | CPR0 | 3.64206197 | 2.60E-06 |
| k__Fungi.p__Fungi_phy_Incertae_sedis.c__Fungi_cla_Incertae_sedis.o__Fungi_ord_Incertae_sedis.f__Fungi_fam_Incertae_sedis.g__Fungi_gen_Incertae_sedis.s__Fungi_spe_Incertae_sedis | 4.917638516 | CPR1 | 4.586282771 | 4.00E-11 |
| p__Ascomycota.c__Sordariomycetes.o__Calosphaeriales | 3.036192366 | HC | 3.197163228 | 3.76E-09 |
| p__Mucoromycota.c__Mucoromycetes.o__Mucorales.f__Mucoraceae | 2.559584224 | CPR0 | 3.38547857 | 1.65E-18 |
| k__Fungi.p__Ascomycota.c__Dothideomycetes.o__Botryosphaeriales.f__Botryosphaeriaceae | 3.787170284 | CPR0 | 3.557861189 | 2.94E-09 |
| k__Fungi.p__Ascomycota.c__Saccharomycetes.o__Saccharomycetales.f__Dipodascaceae.g__Dipodascaceae_gen_Incertae_sedis | 3.990945097 | CPR1 | 3.721240004 | 7.03E-08 |
| p__Basidiomycota.c__Microbotryomycetes.o__Microbotryomycetes_ord_Incertae_sedis.f__Microbotryomycetes_fam_Incertae_sedis.g__Heitmania.s__Heitmania_litseae | 3.660201888 | HC | 3.381761615 | 9.79E-12 |
| k__Fungi.p__Basidiomycota.c__Tremellomycetes.o__Cystofilobasidiales.f__Mrakiaceae.g__Udeniomyces.s__Udeniomyces_megalosporus | 4.414797976 | CPR0 | 4.162361609 | 4.95E-05 |
| k__Fungi.p__Ascomycota.c__Sordariomycetes.o__Hypocreales.f__Nectriaceae | 3.566617146 | CPR0 | 3.246083202 | 0.013429271 |

| Data S12. Statistically significant LDA scores of gut mycobiome at different levels between the three groups | | | | |
| --- | --- | --- | --- | --- |
| Biomaker_names | Logarithm value | Groups | LDA_value | P_value |
| k__Fungi | 5.53841052 | CPR0 | 5.102065096 | 5.17E-05 |
| p__Ascomycota.c__Saccharomycetes.o__Saccharomycetales.f__Trigonopsidaceae.g__Trigonopsis.s__Trigonopsis_californica | 3.603487554 | HC | 3.402700252 | 1.40E-07 |
| p__Ascomycota.c__Pezizomycetes.o__Pezizales | 3.95520541 | CPR1 | 3.605683541 | 0.00036353 |
| p__Ascomycota.c__Dothideomycetes.o__Dothideales.f__Dothioraceae.g__Dothiora | 3.670201016 | CPR1 | 3.270072433 | 0.006318616 |
| p__Ascomycota.c__Sordariomycetes.o__Sordariomycetes_ord_Incertae_sedis.f__Sordariomycetes_fam_Incertae_sedis.g__Acrodictys | 4.736789285 | CPR0 | 4.15872348 | 0.019892368 |
| k__Fungi.p__Ascomycota.c__Dothideomycetes.o__Dothideales | 3.671703159 | CPR1 | 3.271700613 | 0.009992537 |
| k__Fungi.p__Ascomycota.c__Saccharomycetes.o__Saccharomycetales.f__Saccharomycetaceae.g__Eremothecium | 3.599110197 | CPR1 | 3.345730893 | 6.48E-10 |
| k__Fungi.p__Ascomycota.c__Saccharomycetes.o__Saccharomycetales.f__Saccharomycetales_fam_Incertae_sedis.g__Candida.s__Candida_inconspicua | 3.950676988 | CPR0 | 3.657330245 | 1.23E-09 |
| k__Fungi.p__Ascomycota.c__Saccharomycetes.o__Saccharomycetales.f__Saccharomycetales_fam_Incertae_sedis | 4.830322275 | CPR0 | 4.446894957 | 0.001318091 |
| k__Fungi.p__Ascomycota.c__Leotiomycetes.o__Thelebolales.f__Pseudeurotiaceae | 3.862972886 | HC | 3.56422458 | 2.78E-08 |
| k__Fungi.p__Basidiomycota.c__Exobasidiomycetes | 3.371370929 | CPR0 | 3.104934419 | 1.44E-09 |
| k__Fungi.p__Ascomycota.c__Leotiomycetes | 3.929494226 | HC | 3.608845404 | 0.048405092 |
| k__Fungi.p__Ascomycota.c__Leotiomycetes.o__Erysiphales.f__Erysiphaceae.g__Blumeria.s__Blumeria_spe_Incertae_sedis | 3.391897487 | CPR1 | 3.064286374 | 0.000393278 |
| k__Fungi.p__Basidiomycota.c__Microbotryomycetes.o__Sporidiobolales | 4.403939649 | CPR0 | 4.260117066 | 2.15E-05 |
| p__Ascomycota.c__Sordariomycetes.o__Chaetosphaeriales.f__Chaetosphaeriaceae | 4.77597801 | CPR1 | 4.304248729 | 0.001608943 |
| k__Fungi.p__Basidiomycota.c__Tremellomycetes.o__Trichosporonales.f__Trichosporonaceae.g__Apiotrichum | 2.709291084 | HC | 3.182319586 | 2.48E-05 |
| k__Fungi.p__Mucoromycota.c__Mucoromycetes.o__Mucorales.f__Mucoraceae.g__Mucor.s__Mucor_circinelloides | 4.143467342 | CPR0 | 3.824620898 | 2.74E-17 |
| k__Fungi.p__Mucoromycota.c__Mucoromycetes.o__Mucorales.f__Mucoraceae | 4.257279933 | CPR0 | 3.931953178 | 1.79E-12 |
| k__Fungi.p__Fungi_phy_Incertae_sedis.c__Fungi_cla_Incertae_sedis.o__Fungi_ord_Incertae_sedis.f__Fungi_fam_Incertae_sedis | 4.393618144 | HC | 3.736819066 | 5.28E-06 |
| k__Fungi.p__Ascomycota.c__Saccharomycetes.o__Saccharomycetales.f__Dipodascaceae.g__Dipodascus.s__Dipodascus_geotrichum | 4.734681842 | CPR0 | 4.419581948 | 1.25E-14 |
| k__Fungi.p__Basidiomycota.c__Microbotryomycetes.o__Sporidiobolales.f__Sporidiobolaceae.g__Rhodotorula.s__Rhodotorula_mucilaginosa | 4.403766986 | CPR0 | 4.262707819 | 3.68E-10 |
| k__Fungi.p__Ascomycota.c__Leotiomycetes.o__Erysiphales.f__Erysiphaceae | 3.391897487 | CPR1 | 3.0644973 | 0.000461324 |
| k__Fungi.p__Ascomycota.c__Leotiomycetes.o__Erysiphales.f__Erysiphaceae.g__Blumeria | 3.391897487 | CPR1 | 3.064459177 | 0.000382731 |
| p__Ascomycota.c__Dothideomycetes.o__Dothideales | 4.147322271 | CPR1 | 3.746629736 | 0.006318616 |
| p__Ascomycota.c__Saccharomycetes.o__Saccharomycetales | 4.111211174 | HC | 3.884930095 | 0.005551285 |
| p__Ascomycota.c__Ascomycota_cla_Incertae_sedis.o__Ascomycota_ord_Incertae_sedis.f__Ascomycota_fam_Incertae_sedis.g__Milospium | 3.639488867 | CPR1 | 3.099142222 | 0.00123119 |
| k__Fungi.p__Ascomycota.c__Saccharomycetes.o__Saccharomycetales.f__Saccharomycetaceae.g__Saccharomyces.s__Saccharomyces_cerevisiae | 4.748511665 | HC | 4.436224591 | 0.048844492 |
| k__Fungi.p__Basidiomycota.c__Wallemiomycetes.o__Wallemiales | 3.275890319 | HC | 3.003311305 | 0.009259085 |
| k__Fungi.p__Ascomycota.c__Saccharomycetes.o__Saccharomycetales.f__Saccharomycetaceae.g__Saccharomyces | 4.748531308 | HC | 4.436245741 | 0.048844492 |
| p__Ascomycota.c__Pezizomycetes | 3.95520541 | CPR1 | 3.605683541 | 0.00036353 |
| p__Ascomycota.c__Ascomycota_cla_Incertae_sedis.o__Ascomycota_ord_Incertae_sedis | 4.968205713 | HC | 4.662822565 | 7.76E-18 |
| p__Ascomycota.c__Saccharomycetes.o__Saccharomycetales.f__Trigonopsidaceae | 3.603487554 | HC | 3.402705323 | 1.40E-07 |
| k__Fungi.p__Basidiomycota.c__Microbotryomycetes.o__Sporidiobolales.f__Sporidiobolaceae | 4.403939649 | CPR0 | 4.260190058 | 2.15E-05 |
| p__Ascomycota.c__Eurotiomycetes.o__Eurotiales.f__Trichocomaceae.g__Talaromyces.s__Talaromyces_scorteus | 3.702873141 | HC | 3.425629408 | 0.022482688 |
| p__Ascomycota.c__Sordariomycetes.o__Xylariales.f__Iodosphaeriaceae.g__Iodosphaeria.s__Iodosphaeria_phyllophila | 3.415915064 | CPR1 | 3.114257222 | 2.02E-05 |
| p__Ascomycota.c__Ascomycota_cla_Incertae_sedis.o__Ascomycota_ord_Incertae_sedis.f__Ascomycota_fam_Incertae_sedis | 4.968205713 | HC | 4.662822565 | 7.76E-18 |
| k__Fungi.p__Ascomycota.c__Ascomycota_cla_Incertae_sedis.o__Ascomycota_ord_Incertae_sedis.f__Ascomycota_fam_Incertae_sedis.g__Ascomycota_gen_Incertae_sedis | 4.96668507 | HC | 4.662124043 | 4.15E-19 |
| k__Fungi.p__Fungi_phy_Incertae_sedis.c__Fungi_cla_Incertae_sedis.o__Fungi_ord_Incertae_sedis | 4.393618144 | HC | 3.736819066 | 5.28E-06 |
| k__Fungi.p__Ascomycota.c__Dothideomycetes.o__Capnodiales.f__Cladosporiaceae.g__Cladosporium.s__Cladosporium_coloradense | 1.51852311 | CPR0 | 3.355725255 | 2.91E-15 |
| k__Fungi.p__Basidiomycota.c__Exobasidiomycetes.o__Entylomatales.f__Entylomataceae | 3.363528513 | CPR0 | 3.138290086 | 4.66E-12 |
| p__Ascomycota.c__Ascomycota_cla_Incertae_sedis.o__Ascomycota_ord_Incertae_sedis.f__Ascomycota_fam_Incertae_sedis.g__Milospium.s__Milospium_graphideorum | 3.639488867 | CPR1 | 3.099175518 | 0.00123119 |
| k__Fungi.p__Basidiomycota.c__Wallemiomycetes | 3.275890319 | HC | 3.003486838 | 0.009259085 |
| p__Ascomycota.c__Pezizomycetes.o__Pezizales.f__Pezizaceae.g__Hydnobolites | 3.938880231 | CPR1 | 3.593888968 | 2.58E-05 |
| p__Ascomycota.c__Sordariomycetes.o__Chaetosphaeriales.f__Chaetosphaeriaceae.g__Zanclospora.s__Zanclospora_jonesii | 4.774832972 | CPR1 | 4.303738676 | 0.001608943 |
| k__Fungi.p__Fungi_phy_Incertae_sedis.c__Fungi_cla_Incertae_sedis | 4.393618144 | HC | 3.736819066 | 5.28E-06 |
| k__Fungi.p__Ascomycota.c__Saccharomycetes.o__Saccharomycetales.f__Debaryomycetaceae | 4.264692016 | CPR1 | 3.848130802 | 2.87E-05 |
| p__Basidiomycota.c__Agaricomycetes.o__Russulales.f__Russulaceae.g__Russula.s__Russula_xerophila | 1.687248183 | CPR1 | 3.067362804 | 0.001855105 |
| k__Fungi.p__Ascomycota.c__Saccharomycetes.o__Saccharomycetales.f__Saccharomycetales_fam_Incertae_sedis.g__Saccharomycetales_gen_Incertae_sedis | 3.558 | CPR0 | 3.201180395 | 0.005499377 |
| k__Fungi.p__Ascomycota.c__Saccharomycetes | 5.441017818 | CPR0 | 5.003007212 | 0.003498831 |
| p__Ascomycota | 5.875223412 | CPR1 | 4.993338763 | 0.007600038 |
| p__Ascomycota.c__Saccharomycetes.o__Saccharomycetales.f__Trigonopsidaceae.g__Trigonopsis | 3.603487554 | HC | 3.40274019 | 1.40E-07 |
| p__Ascomycota.c__Sordariomycetes.o__Xylariales.f__Iodosphaeriaceae.g__Iodosphaeria | 3.415915064 | CPR1 | 3.114257289 | 2.02E-05 |
| k__Fungi.p__Mucoromycota.c__Mucoromycetes.o__Mucorales.f__Mucoraceae.g__Actinomucor.s__Actinomucor_elegans | 3.606344827 | CPR0 | 3.27670384 | 2.08E-06 |
| p__Ascomycota.c__Sordariomycetes.o__Chaetosphaeriales | 4.77597801 | CPR1 | 4.304228349 | 0.001608943 |
| p__Ascomycota.c__Dothideomycetes.o__Dothideales.f__Dothioraceae | 3.670201016 | CPR1 | 3.270072433 | 0.006318616 |
| p__Ascomycota.c__Pezizomycetes.o__Pezizales.f__Pezizaceae.g__Hydnobolites.s__Hydnobolites_roseus | 3.938880231 | CPR1 | 3.594797813 | 2.58E-05 |
| p__Ascomycota.c__Sordariomycetes.o__Chaetosphaeriales.f__Chaetosphaeriaceae.g__Zanclospora | 4.77597801 | CPR1 | 4.304248729 | 0.001608943 |
| k__Fungi.p__Ascomycota.c__Dothideomycetes.o__Pleosporales.f__Didymosphaeriaceae.g__Didymosphaeriaceae_gen_Incertae_sedis.s__Didymosphaeriaceae_spe_Incertae_sedis | 1.467650172 | CPR0 | 3.190709872 | 2.44E-16 |
| p__Ascomycota.c__Sordariomycetes.o__Sordariomycetes_ord_Incertae_sedis.f__Sordariomycetes_fam_Incertae_sedis.g__Acrodictys.s__Acrodictys_fluminicola | 4.736789285 | CPR0 | 4.15872348 | 0.019892368 |
| k__Fungi.p__Basidiomycota.c__Exobasidiomycetes.o__Entylomatales | 3.369998243 | CPR0 | 3.107237816 | 2.96E-09 |
| k__Fungi.p__Mucoromycota.c__Mucoromycetes.o__Mucorales.f__Mucoraceae.g__Actinomucor | 3.606344827 | CPR0 | 3.279344674 | 2.08E-06 |
| k__Fungi.p__Ascomycota.c__Saccharomycetes.o__Saccharomycetales.f__Dipodascaceae.g__Dipodascaceae_gen_Incertae_sedis.s__Dipodascaceae_spe_Incertae_sedis | 5.133203832 | CPR0 | 4.83882873 | 4.05E-11 |
| k__Fungi.p__Mucoromycota | 4.278009676 | CPR0 | 3.95218722 | 1.73E-06 |
| p__Ascomycota.c__Saccharomycetes | 4.111211174 | HC | 3.884930095 | 0.005551285 |
| k__Fungi.p__Basidiomycota.c__Microbotryomycetes.o__Sporidiobolales.f__Sporidiobolaceae.g__Rhodotorula | 4.403766986 | CPR0 | 4.261722043 | 5.35E-08 |
| k__Fungi.p__Ascomycota.c__Saccharomycetes.o__Saccharomycetales.f__Saccharomycetales_fam_Incertae_sedis.g__Candida.s__Candida_tropicalis | 3.213690878 | HC | 3.096402034 | 0.00117322 |
| k__Fungi.p__Ascomycota.c__Saccharomycetes.o__Saccharomycetales | 5.441017818 | CPR0 | 5.003007212 | 0.003498831 |
| p__Ascomycota.c__Pezizomycetes.o__Pezizales.f__Pezizaceae | 3.938893232 | CPR1 | 3.591025343 | 0.00025511 |
| k__Fungi.p__Basidiomycota.c__Wallemiomycetes.o__Wallemiales.f__Wallemiaceae | 3.275890319 | HC | 3.00315558 | 0.009259085 |
| k__Fungi.p__Basidiomycota.c__Exobasidiomycetes.o__Entylomatales.f__Entylomataceae.g__Entyloma | 3.363528513 | CPR0 | 3.141143451 | 4.66E-12 |
| k__Fungi.p__Ascomycota.c__Saccharomycetes.o__Saccharomycetales.f__Debaryomycetaceae.g__Debaryomyces | 4.262156601 | CPR1 | 3.850750873 | 5.30E-07 |
| p__Ascomycota.c__Sordariomycetes.o__Sordariomycetes_ord_Incertae_sedis | 4.739253701 | CPR0 | 4.158103458 | 0.020884877 |
| k__Fungi.p__Ascomycota.c__Saccharomycetes.o__Saccharomycetales.f__Dipodascaceae.g__Dipodascus | 4.741341018 | CPR0 | 4.424632313 | 7.17E-11 |
| k__Fungi.p__Ascomycota.c__Dothideomycetes.o__Pleosporales.f__Didymosphaeriaceae.g__Didymosphaeriaceae_gen_Incertae_sedis | 1.467650172 | CPR0 | 3.20358474 | 2.44E-16 |
| k__Fungi.p__Ascomycota.c__Saccharomycetes.o__Saccharomycetales.f__Saccharomycetales_fam_Incertae_sedis.g__Candida | 4.802939581 | CPR0 | 4.417177358 | 0.000731831 |
| k__Fungi.p__Ascomycota.c__Ascomycota_cla_Incertae_sedis.o__Ascomycota_ord_Incertae_sedis.f__Ascomycota_fam_Incertae_sedis | 5.267715065 | HC | 4.963148777 | 4.15E-19 |
| p__Ascomycota.c__Ascomycota_cla_Incertae_sedis | 4.968205713 | HC | 4.662822565 | 7.76E-18 |
| k__Fungi.p__Ascomycota.c__Saccharomycetes.o__Saccharomycetales.f__Saccharomycetales_fam_Incertae_sedis.g__Saccharomycetales_gen_Incertae_sedis.s__Saccharomycetales_spe_Incertae_sedis | 3.558 | CPR0 | 3.201276331 | 0.005499377 |
| k__Fungi.p__Ascomycota.c__Dothideomycetes.o__Pleosporales.f__Didymosphaeriaceae | 1.467650172 | CPR0 | 3.145491068 | 2.28E-14 |
| k__Fungi.p__Mucoromycota.c__Mucoromycetes.o__Mucorales.f__Mucoraceae.g__Mucor | 4.147482576 | CPR0 | 3.828167777 | 1.70E-15 |
| k__Fungi.p__Ascomycota.c__Ascomycota_cla_Incertae_sedis.o__Ascomycota_ord_Incertae_sedis.f__Ascomycota_fam_Incertae_sedis.g__Ascomycota_gen_Incertae_sedis.s__Ascomycota_spe_Incertae_sedis | 4.96668507 | HC | 4.662124043 | 4.15E-19 |
| p__Ascomycota.c__Sordariomycetes | 5.04859799 | CPR1 | 4.491645752 | 1.59E-07 |
| p__Ascomycota.c__Dothideomycetes | 4.191616635 | HC | 3.77372671 | 0.011413875 |
| p__Ascomycota.c__Sordariomycetes.o__Xylariales | 3.893036319 | CPR1 | 3.590756753 | 2.02E-05 |
| k__Fungi.p__Ascomycota.c__Ascomycota_cla_Incertae_sedis.o__Ascomycota_ord_Incertae_sedis | 5.267715065 | HC | 4.963148777 | 4.15E-19 |
| k__Fungi.p__Basidiomycota.c__Exobasidiomycetes.o__Entylomatales.f__Entylomataceae.g__Entyloma.s__Entyloma_calendulae | 3.327424891 | CPR0 | 3.223503947 | 1.33E-10 |
| k__Fungi.p__Mucoromycota.c__Mucoromycetes | 4.257279933 | CPR0 | 3.930017051 | 5.08E-11 |
| k__Fungi.p__Basidiomycota.c__Wallemiomycetes.o__Wallemiales.f__Wallemiaceae.g__Wallemia | 3.275890319 | HC | 3.003214865 | 0.009259085 |
| k__Fungi.p__Ascomycota.c__Sordariomycetes | 4.033069468 | CPR0 | 3.604283095 | 0.004227709 |
| k__Fungi.p__Basidiomycota.c__Tremellomycetes.o__Trichosporonales.f__Trichosporonaceae.g__Apiotrichum.s__Apiotrichum_domesticum | 2.709291084 | HC | 3.113290287 | 1.04E-05 |
| k__Fungi.p__Ascomycota.c__Saccharomycetes.o__Saccharomycetales.f__Saccharomycetales_fam_Incertae_sedis.g__Candida.s__Candida_solani | 4.68759967 | CPR0 | 4.369989013 | 6.25E-11 |
| k__Fungi.p__Ascomycota.c__Saccharomycetes.o__Saccharomycetales.f__Dipodascaceae | 5.292773269 | CPR0 | 4.988018186 | 1.43E-08 |
| k__Fungi.p__Ascomycota.c__Saccharomycetes.o__Saccharomycetales.f__Debaryomycetaceae.g__Debaryomyces.s__Debaryomyces_prosopidis | 4.262156601 | CPR1 | 3.850750873 | 5.30E-07 |
| k__Fungi.p__Ascomycota.c__Leotiomycetes.o__Erysiphales | 3.391897487 | CPR1 | 3.0644973 | 0.000461324 |
| k__Fungi.p__Ascomycota.c__Sordariomycetes.o__Xylariales | 3.416799582 | CPR1 | 3.108487046 | 0.007575233 |
| k__Fungi.p__Ascomycota | 5.597105898 | CPR0 | 4.903377021 | 0.024567756 |
| k__Fungi.p__Fungi_phy_Incertae_sedis.c__Fungi_cla_Incertae_sedis.o__Fungi_ord_Incertae_sedis.f__Fungi_fam_Incertae_sedis.g__Fungi_gen_Incertae_sedis | 4.393618144 | HC | 3.736819066 | 5.28E-06 |
| p__Ascomycota.c__Sordariomycetes.o__Sordariomycetes_ord_Incertae_sedis.f__Sordariomycetes_fam_Incertae_sedis | 4.739253701 | CPR0 | 4.158103458 | 0.020884877 |
| k__Fungi.p__Mucoromycota.c__Mucoromycetes.o__Mucorales | 4.257279933 | CPR0 | 3.927688342 | 5.08E-11 |
| p__Ascomycota.c__Dothideomycetes.o__Dothideales.f__Dothioraceae.g__Dothiora.s__Dothiora_spartii | 3.670201016 | CPR1 | 3.270072433 | 0.006318616 |
| k__Fungi.p__Ascomycota.c__Ascomycota_cla_Incertae_sedis | 5.267715065 | HC | 4.963148777 | 4.15E-19 |
| k__Fungi.p__Fungi_phy_Incertae_sedis.c__Fungi_cla_Incertae_sedis.o__Fungi_ord_Incertae_sedis.f__Fungi_fam_Incertae_sedis.g__Fungi_gen_Incertae_sedis.s__Fungi_spe_Incertae_sedis | 4.393618144 | HC | 3.736819066 | 5.28E-06 |
| p__Ascomycota.c__Sordariomycetes.o__Xylariales.f__Iodosphaeriaceae | 3.415915064 | CPR1 | 3.11425734 | 2.02E-05 |
| k__Fungi.p__Ascomycota.c__Saccharomycetes.o__Saccharomycetales.f__Dipodascaceae.g__Dipodascaceae_gen_Incertae_sedis | 5.133203832 | CPR0 | 4.838874371 | 4.05E-11 |

| Data S13. Possible enrichment pathways of oral fungi affecting COVID-19 recovery identified based on MetaCyc database | | | | |
| --- | --- | --- | --- | --- |
| Biomaker_names | Logarithm value | Groups | LDA_value | P_value |
| COLANSYN_PWY_colanic_acid_building_blocks_biosynthesis | 4.407689223 | CPR0 | 3.89659162 | 0.003576 |
| GLYOXYLATE_BYPASS_glyoxylate_cycle | 5.494748566 | CPR1 | 4.16220312 | 4.70E-05 |
| GLYCOCAT_PWY_glycogen_degradation_I_bacterial | 4.394935261 |  |  | - |
| PWY_5920_superpathway_of_heme_biosynthesis_from_glycine | 5.23154944 | CPR1 | 4.23995227 | 2.27E-06 |
| COA_PWY_coenzyme_A_biosynthesis_I | 5.212762746 |  |  | - |
| PWY_5667_CDP_diacylglycerol_biosynthesis_I | 5.151730413 | HC | 4.25163038 | 1.00E-08 |
| PWY_7209_superpathway_of_pyrimidine_ribonucleosides_degradation | 4.68525579 | CPR0 | 4.17388229 | 0.000114 |
| PWY_7385_13_propanediol_biosynthesis_engineered | 5.093279392 |  |  | - |
| GLUCOSE1PMETAB_PWY_glucose_and_glucose_1_phosphate_degradation | 4.170920261 |  |  | - |
| PWY_7219_adenosine_ribonucleotides_de_novo_biosynthesis | 5.406097508 |  |  | - |
| PWY_6737_starch_degradation_V | 4.305810481 |  |  | - |
| PWY_6121_5_aminoimidazole_ribonucleotide_biosynthesis_I | 4.797928849 |  |  | - |
| PWY_7007_methyl_ketone_biosynthesis | 5.400209441 | HC | 4.34891281 | 0.000207 |
| PWY_621_sucrose_degradation_III_sucrose_invertase | 4.536471751 |  |  | - |
| POLYAMINSYN3_PWY_superpathway_of_polyamine_biosynthesis_II | 2.992411554 |  |  | - |
| PWY_5189_tetrapyrrole_biosynthesis_II_from_glycine | 5.231642737 | CPR1 | 4.35562512 | 1.98E-08 |
| PANTOSYN_PWY_pantothenate_and_coenzyme_A_biosynthesis_I | 4.625872014 | CPR0 | 4.16887263 | 8.08E-05 |
| PWY0_166_superpathway_of_pyrimidine_deoxyribonucleotides_ de_novo_biosynthesis_E__coli | 5.316056587 | HC | 4.59561137 | 1.53E-09 |
| VALSYN_PWY_L_valine_biosynthesis | 5.341592573 | CPR1 | 4.15241392 | 0.000143 |
| PWY_7220_adenosine_deoxyribonucleotides_de_novo_biosynthesis_II | 5.505053715 | HC | 4.50601719 | 0.00013 |
| PWY_6277_superpathway_of_5_aminoimidazole_ribonucleotide_biosynthesis | 4.75933955 |  |  | - |
| PWY_922_mevalonate_pathway_I | 5.236357833 | CPR1 | 3.93732279 | 0.025408 |
| GLYCOGENSYNTH_PWY_glycogen_biosynthesis_I_from_ADP_D_Glucose | 4.055302497 | CPR0 | 3.72548185 | 0.001306 |
| PWY_7208_superpathway_of_pyrimidine_nucleobases_salvage | 5.298381485 |  |  | - |
| PWY_7198_pyrimidine_deoxyribonucleotides_de_novo_biosynthesis_IV | 5.019024615 |  |  | - |
| PWY_7228_superpathway_of_guanosine_nucleotides_de_novo_biosynthesis_I | 5.321675185 |  |  | - |
| HSERMETANA_PWY_L_methionine_biosynthesis_III | 4.802573422 | HC | 4.49504718 | 2.37E-17 |
| GLUTORN_PWY_L_ornithine_biosynthesis | 3.348563211 |  |  | - |
| PWY_5989_stearate_biosynthesis_II_bacteria_and_plants | 4.459883693 | CPR0 | 3.99294745 | 6.36E-05 |
| PWY_7222_guanosine_deoxyribonucleotides_de_novo_biosynthesis_II | 5.505053715 | HC | 4.50601719 | 0.00013 |
| THRESYN_PWY_superpathway_of_L_threonine_biosynthesis | 5.263098192 |  |  | - |
| PHOSLIPSYN_PWY_superpathway_of_phospholipid_biosynthesis_I_bacteria | 4.266989525 |  |  | - |
| PWY_7431_aromatic_biogenic_amine_degradation_bacteria | 5.062431362 |  |  | - |
| PANTO_PWY_phosphopantothenate_biosynthesis_I | 5.275512999 |  |  | - |
| PWY_6126_superpathway_of_adenosine_nucleotides_de_novo_biosynthesis_II | 5.381797013 |  |  | - |
| PWY_4984_urea_cycle | 5.221606747 |  |  | - |
| TCA_TCA_cycle_I_prokaryotic | 5.218116 |  |  | - |
| PWY_7347_sucrose_biosynthesis_III | 4.08222588 | CPR0 | 3.92882332 | 1.95E-06 |
| PWY_7663_gondoate_biosynthesis_anaerobic | 4.369587049 |  |  | - |
| PWY_7210_pyrimidine_deoxyribonucleotides_biosynthesis_from_CTP | 5.08254583 |  |  | - |
| PWY_7196_superpathway_of_pyrimidine_ribonucleosides_salvage | 4.85652268 | HC | 4.55107186 | 2.28E-17 |
| TYRFUMCAT_PWY_L_tyrosine_degradation_I | 4.806856135 | HC | 4.14690324 | 0.003132 |
| PWY_7328_superpathway_of_UDP_glucose_derived_O_antigen_building_blocks_biosynthesis | 4.12229133 | CPR0 | 3.78935842 | 0.000284 |
| PWY_7111_pyruvate_fermentation_to_isobutanol_engineered | 5.391030396 | CPR1 | 4.19308083 | 0.00057 |
| PWY_7229_superpathway_of_adenosine_nucleotides_de_novo_biosynthesis_I | 5.383947001 |  |  | - |
| FAO_PWY_fatty_acid_beta_oxidation_I | 5.538557932 | CPR1 | 4.40060946 | 0.01 |
| HEME_BIOSYNTHESIS_II_heme_biosynthesis_I_aerobic | 5.233914767 | CPR1 | 3.99462788 | 0.026248 |
| PWY_5651_L_tryptophan_degradation_to_2_amino_3_carboxymuconate_semialdehyde | 4.778161493 | CPR0 | 4.37577948 | 8.44E-08 |
| PWY_3781_aerobic_respiration_I_cytochrome_c | 5.843876868 | CPR1 | 4.67145174 | 4.43E-05 |
| LEU_DEG2_PWY_L_leucine_degradation_I | 4.768542467 | HC | 4.15543623 | 0.001998 |
| PWY_6609_adenine_and_adenosine_salvage_III | 5.145885173 | CPR0 | 4.19259365 | 0.006681 |
| PWY4FS_8_phosphatidylglycerol_biosynthesis_II_non_plastidic | 4.076816379 |  |  | - |
| PWY_6608_guanosine_nucleotides_degradation_III | 5.217113703 | CPR0 | 4.18824032 | 0.036398 |
| PWY_5705_allantoin_degradation_to_glyoxylate_III | 3.840632048 | CPR1 | 3.7863706 | 0.000764 |
| PWY_5695_urate_biosynthesis_inosine_5_phosphate_degradation | 5.181655075 |  |  | - |
| NAD_BIOSYNTHESIS_II_NAD_salvage_pathway_II | 4.950833682 |  |  | - |
| PWY_6125_superpathway_of_guanosine_nucleotides_de_novo_biosynthesis_II | 5.301286049 |  |  | - |
| SER_GLYSYN_PWY_superpathway_of_L_serine_and_glycine_biosynthesis_I | 5.319053922 | CPR1 | 3.9289724 | 0.002448 |
| ARGSYNBSUB_PWY_L_arginine_biosynthesis_II_acetyl_cycle | 3.297085166 |  |  | - |
| PWY_7197_pyrimidine_deoxyribonucleotide_phosphorylation | 5.261931332 | HC | 4.41814906 | 9.21E-10 |
| SUCSYN_PWY_sucrose_biosynthesis_I_from_photosynthesis | 4.382491457 | CPR0 | 4.01502126 | 2.52E-06 |
| PWY_6122_5_aminoimidazole_ribonucleotide_biosynthesis_II | 4.75933955 |  |  | - |
| SO4ASSIM_PWY_sulfate_reduction_I_assimilatory | 5.131705522 | CPR0 | 4.30037925 | 1.64E-06 |
| PWY_7221_guanosine_ribonucleotides_de_novo_biosynthesis | 5.271527749 |  |  | - |
| ARGSYN_PWY_L_arginine_biosynthesis_I_via_L_ornithine | 3.892041508 |  |  | - |
| PWY0_1319_CDP_diacylglycerol_biosynthesis_II | 5.151730413 | HC | 4.25163038 | 1.00E-08 |
| PWY_7323_superpathway_of_GDP_mannose_derived_O_antigen_building_blocks_biosynthesis | 5.108306476 | HC | 4.5638473 | 8.18E-07 |
| PWY_5659_GDP_mannose_biosynthesis | 5.390543195 |  |  | - |
| PENTOSE_P_PWY_pentose_phosphate_pathway | 5.28300466 | CPR1 | 3.93804759 | 0.002819 |
| FASYN_ELONG_PWY_fatty_acid_elongation_saturated | 5.394231741 | CPR1 | 4.71877822 | 0.000739 |
| GLUCONEO_PWY_gluconeogenesis_I | 3.391450189 |  |  | - |
| PWY4FS_7_phosphatidylglycerol_biosynthesis_I_plastidic | 4.076816379 |  |  | - |
| PWY_6317_galactose_degradation_I_Leloir_pathway | 5.08160227 |  |  | - |
| LIPASYN_PWY_phospholipases | 4.332909646 | CPR0 | 3.95089435 | 5.23E-05 |
| P221_PWY_octane_oxidation | 4.840923367 | CPR1 | 4.22161578 | 0.00096 |
| ANAGLYCOLYSIS_PWY_glycolysis_III_from_glucose | 5.204633519 |  |  | - |
| PWY_6545_pyrimidine_deoxyribonucleotides_de_novo_biosynthesis_III | 5.089172209 |  |  | - |
| CALVIN_PWY_Calvin_Benson_Bassham_cycle | 5.351383531 | CPR1 | 4.31112043 | 1.31E-08 |
| TRNA_CHARGING_PWY_tRNA_charging | 5.388974686 | CPR1 | 4.07161693 | 0.042191 |
| PWY_7184_pyrimidine_deoxyribonucleotides_de_novo_biosynthesis_I | 5.344515788 | HC | 4.5601046 | 1.02E-09 |
| NONOXIPENT_PWY_pentose_phosphate_pathway_non_oxidative_branch | 5.372019229 | CPR1 | 4.1108493 | 3.04E-05 |

| Data S14. Possible enrichment pathways of gut fungi affecting COVID-19 recovery identified based on MetaCyc database | | | | |
| --- | --- | --- | --- | --- |
| Biomaker_names | Logarithm value | Groups | LDA_value | P_value |
| COLANSYN_PWY_colanic_acid_building_blocks_biosynthesis | 4.092114326 |  |  | - |
| GLYOXYLATE_BYPASS_glyoxylate_cycle | 5.54732153 |  |  | - |
| GLYCOCAT_PWY_glycogen_degradation_I_bacterial | 4.188612984 |  |  | - |
| PWY_5920_superpathway_of_heme_biosynthesis_from_glycine | 5.249973744 |  |  | - |
| COA_PWY_coenzyme_A_biosynthesis_I | 5.238132605 |  |  | - |
| PWY_5667_CDP_diacylglycerol_biosynthesis_I | 5.210005324 |  |  | - |
| PWY_7209_superpathway_of_pyrimidine_ribonucleosides_degradation | 4.313046418 |  |  | - |
| PWY_7385_13_propanediol_biosynthesis_engineered | 5.220323614 |  |  | - |
| GLUCOSE1PMETAB_PWY_glucose_and_glucose_1_phosphate_degradation | 4.017010904 |  |  | - |
| PWY_7219_adenosine_ribonucleotides_de_novo_biosynthesis | 5.438358804 |  |  | - |
| PWY_6737_starch_degradation_V | 4.121795576 |  |  | - |
| PWY_6121_5_aminoimidazole_ribonucleotide_biosynthesis_I | 4.261412947 |  |  | - |
| PWY_7007_methyl_ketone_biosynthesis | 5.111886935 |  |  | - |
| PWY_621_sucrose_degradation_III_sucrose_invertase | 4.270997928 |  |  | - |
| POLYAMINSYN3_PWY_superpathway_of_polyamine_biosynthesis_II | 2.672331872 |  |  | - |
| PWY_5189_tetrapyrrole_biosynthesis_II_from_glycine | 5.219090833 |  |  | - |
| PANTOSYN_PWY_pantothenate_and_coenzyme_A_biosynthesis_I | 4.842932934 | CPR0 | 4.528177 | #### |
| PWY0_166_superpathway_of_pyrimidine_deoxyribonucleotides_de_novo_ biosynthesis_E__coli | 5.322778168 |  |  | - |
| VALSYN_PWY_L_valine_biosynthesis | 5.311946363 |  |  | - |
| PWY_7220_adenosine_deoxyribonucleotides_de_novo_biosynthesis_II | 5.4908249 |  |  | - |
| PWY_6277_superpathway_of_5_aminoimidazole_ribonucleotide_biosynthesis | 4.20370705 |  |  | - |
| PWY_922_mevalonate_pathway_I | 5.252521842 |  |  | - |
| GLYCOGENSYNTH_PWY_glycogen_biosynthesis_I_from_ADP_D_Glucose | 4.044683953 |  |  | - |
| PWY_7208_superpathway_of_pyrimidine_nucleobases_salvage | 5.301331877 |  |  | - |
| PWY_7198_pyrimidine_deoxyribonucleotides_de_novo_biosynthesis_IV | 5.226195544 |  |  | - |
| PWY_7228_superpathway_of_guanosine_nucleotides_de_novo_biosynthesis_I | 5.342024464 | CPR1 | 4.079682 | 0.026 |
| HSERMETANA_PWY_L_methionine_biosynthesis_III | 4.702263979 |  |  | - |
| GLUTORN_PWY_L_ornithine_biosynthesis | 3.425574598 | HC | 3.91993 | #### |
| PWY_5989_stearate_biosynthesis_II_bacteria_and_plants | 4.278005591 |  |  | - |
| PWY_7222_guanosine_deoxyribonucleotides_de_novo_biosynthesis_II | 5.4908249 |  |  | - |
| THRESYN_PWY_superpathway_of_L_threonine_biosynthesis | 5.280132079 |  |  | - |
| PHOSLIPSYN_PWY_superpathway_of_phospholipid_biosynthesis_I_bacteria | 3.986118398 |  |  | - |
| PWY_7431_aromatic_biogenic_amine_degradation_bacteria | 4.877397647 | HC | 4.33754 | 0.023 |
| PANTO_PWY_phosphopantothenate_biosynthesis_I | 5.343893822 |  |  | - |
| PWY_6126_superpathway_of_adenosine_nucleotides_de_novo_biosynthesis_II | 5.417038753 |  |  | - |
| PWY_4984_urea_cycle | 5.240460602 |  |  | - |
| TCA_TCA_cycle_I_prokaryotic | 5.016813446 |  |  | - |
| PWY_7347_sucrose_biosynthesis_III | 3.652131683 | CPR0 | 4.044135 | 0.014 |
| PWY_7663_gondoate_biosynthesis_anaerobic | 4.237696997 |  |  | - |
| PWY_7210_pyrimidine_deoxyribonucleotides_biosynthesis_from_CTP | 5.266052469 |  |  | - |
| PWY_7196_superpathway_of_pyrimidine_ribonucleosides_salvage | 4.768435589 |  |  | - |
| TYRFUMCAT_PWY_L_tyrosine_degradation_I | 4.676048012 |  |  | - |
| PWY_7328_superpathway_of_UDP_glucose_derived_O_antigen_building _blocks_biosynthesis | 3.684791227 |  |  | - |
| PWY_7111_pyruvate_fermentation_to_isobutanol_engineered | 5.380750706 |  |  | - |
| PWY_7229_superpathway_of_adenosine_nucleotides_de_novo_biosynthesis_I | 5.424602131 |  |  | - |
| FAO_PWY_fatty_acid_beta_oxidation_I | 5.242469398 |  |  | - |
| HEME_BIOSYNTHESIS_II_heme_biosynthesis_I_aerobic | 5.293810874 |  |  | - |
| PWY_5651_L_tryptophan_degradation_to_2_amino_3_carboxymuconate_ semialdehyde | 4.822717625 | CPR0 | 4.492835 | #### |
| PWY_3781_aerobic_respiration_I_cytochrome_c | 5.918986764 |  |  | - |
| LEU_DEG2_PWY_L_leucine_degradation_I | 4.561705424 | HC | 4.092735 | 0.001 |
| PWY_6609_adenine_and_adenosine_salvage_III | 5.216689181 |  |  | - |
| PWY4FS_8_phosphatidylglycerol_biosynthesis_II_non_plastidic | 3.824178469 |  |  | - |
| PWY_6608_guanosine_nucleotides_degradation_III | 5.277682334 | CPR0 | 4.540068 | #### |
| PWY_5705_allantoin_degradation_to_glyoxylate_III | 3.436623441 |  |  | - |
| PWY_5695_urate_biosynthesis_inosine_5_phosphate_degradation | 5.241318733 | CPR0 | 4.473681 | #### |
| NAD_BIOSYNTHESIS_II_NAD_salvage_pathway_II | 4.856240786 | CPR0 | 4.271375 | 0.018 |
| PWY_6125_superpathway_of_guanosine_nucleotides_de_novo_biosynthesis_II | 5.323847483 | CPR1 | 4.082638 | 0.039 |
| SER_GLYSYN_PWY_superpathway_of_L_serine_and_glycine_biosynthesis_I | 5.325437243 |  |  | - |
| ARGSYNBSUB_PWY_L_arginine_biosynthesis_II_acetyl_cycle | 3.999611293 | HC | 3.618224 | #### |
| PWY_7197_pyrimidine_deoxyribonucleotide_phosphorylation | 5.274911768 |  |  | - |
| SUCSYN_PWY_sucrose_biosynthesis_I_from_photosynthesis | 3.967188869 | CPR0 | 3.931106 | 0.021 |
| PWY_6122_5_aminoimidazole_ribonucleotide_biosynthesis_II | 4.20370705 |  |  | - |
| SO4ASSIM_PWY_sulfate_reduction_I_assimilatory | 5.210017061 |  |  | - |
| PWY_7221_guanosine_ribonucleotides_de_novo_biosynthesis | 5.279189046 | CPR1 | 3.863723 | 0.026 |
| ARGSYN_PWY_L_arginine_biosynthesis_I_via_L_ornithine | 3.958153032 | HC | 3.756725 | #### |
| PWY0_1319_CDP_diacylglycerol_biosynthesis_II | 5.210005324 |  |  | - |
| PWY_7323_superpathway_of_GDP_mannose_derived_O_antigen_building_ blocks_biosynthesis | 5.000640033 |  |  | - |
| PWY_5659_GDP_mannose_biosynthesis | 5.417105976 |  |  | - |
| PENTOSE_P_PWY_pentose_phosphate_pathway | 5.295992332 |  |  | - |
| FASYN_ELONG_PWY_fatty_acid_elongation_saturated | 5.012726913 |  |  | - |
| GLUCONEO_PWY_gluconeogenesis_I | 3.052470327 |  |  | - |
| PWY4FS_7_phosphatidylglycerol_biosynthesis_I_plastidic | 3.824178469 |  |  | - |
| PWY_6317_galactose_degradation_I_Leloir_pathway | 5.070273041 | CPR0 | 4.298882 | 0.014 |
| LIPASYN_PWY_phospholipases | 3.683491006 |  |  | - |
| P221_PWY_octane_oxidation | 4.677794892 | CPR0 | 4.198281 | 0.002 |
| ANAGLYCOLYSIS_PWY_glycolysis_III_from_glucose | 5.254144936 |  |  | - |
| PWY_6545_pyrimidine_deoxyribonucleotides_de_novo_biosynthesis_III | 5.275827195 |  |  | - |
| CALVIN_PWY_Calvin_Benson_Bassham_cycle | 5.283612775 |  |  | - |
| TRNA_CHARGING_PWY_tRNA_charging | 5.424663189 |  |  | - |
| PWY_7184_pyrimidine_deoxyribonucleotides_de_novo_biosynthesis_I | 5.352661795 |  |  | - |
| NONOXIPENT_PWY_pentose_phosphate_pathway_non_oxidative_branch | 5.332886711 |  |  | - |

| Data S15. Optimal biomarkers for prediction models of oral fungi | | | |
| --- | --- | --- | --- |
| OTU ID | OTU88 (Pleurotus_ostreatus) | OTU96 (Candida_inconspicua) | OTU419 (Zanclospora_jonesii) |
| CPR0_1O | 0 | 0.015502 | 6.12E-04 |
| CPR0_5O | 6.90E-05 | 0 | 0 |
| CPR0_6O | 6.50E-05 | 4.68E-04 | 0 |
| CPR0_9O | 0.001885 | 0 | 1.06E-04 |
| CPR0_13O | 0.001418 | 1.29E-04 | 0 |
| CPR0_15O | 0 | 0 | 0 |
| CPR0_19O | 0.006051 | 0 | 0.00346 |
| CPR0_2O | 1.31E-04 | 0 | 0 |
| CPR0_3O | 0 | 0 | 0 |
| CPR0_4O | 0 | 0 | 0 |
| CPR0_11O | 0 | 0 | 0 |
| CPR0_12O | 0 | 0 | 0 |
| CPR0_16O | 0 | 0 | 0 |
| CPR0_18O | 0 | 0 | 0 |
| CPR0_20O | 0 | 0 | 0 |
| CPR0_21O | 0 | 0 | 0 |

| Data S16. Optimal biomarkers for prediction models of gut fungi | | | | | | | | | | | |
| --- | --- | --- | --- | --- | --- | --- | --- | --- | --- | --- | --- |
| OTU ID | CPR0_1F | CPR0_5F | CPR0_9F | CPR0_13F | CPR0_15F | CPR0_2F | CPR0_3F | CPR0_4F | CPR0_11F | CPR0_12F | CPR0_16F |
| OTU4 (Zanclospora_jonesii) | 0.003231 | 0.006429 | 0.010605 | 0.023846 | 0.005288 | 0.054334 | 0.070892 | 0.029637 | 0.057024 | 0.016496 | 0.093047 |
| OTU12 (Acrodictys_fluminicola) | 2.84E-04 | 0.030593 | 0.003122 | 8.76E-04 | 0.001943 | 0.390193 | 0.082625 | 0.07072 | 0.0175 | 0.287303 | 0.025885 |
| OTU205 (Yarrowia_spe_Incertae_sedis) | 0.16853 | 0 | 6.28E-04 | 1.83E-04 | 0 | 0 | 0 | 0 | 0 | 0 | 0 |
| OTU235 (Issatchenkia_orientalis) | 0 | 0.002488 | 8.46E-04 | 0 | 1.70E-05 | 0 | 0 | 0 | 0 | 0 | 0 |
| OTU340 (Fungi_spe_Incertae_sedis) | 0 | 7.30E-05 | 0 | 2.20E-04 | 0 | 0.030568 | 4.05E-04 | 0 | 0.002949 | 3.83E-04 | 0.001345 |
| OTU1500 (Saccharomyces_cerevisiae) | 0 | 4.40E-05 | 0 | 0 | 0 | 0 | 2.59E-04 | 5.60E-05 | 0.001428 | 4.04E-04 | 3.86E-04 |
| OTU2168 (Acrodictys_fluminicola) | 0 | 7.83E-04 | 1.69E-04 | 0 | 9.80E-05 | 0.007528 | 0.001672 | 0.001256 | 5.54E-04 | 0.006212 | 4.88E-04 |
| OTU2742 (Fungi_spe_Incertae_sedis) | 0 | 1.89E-04 | 1.38E-04 | 5.84E-04 | 1.44E-04 | 0.001142 | 0.001313 | 4.42E-04 | 0.00152 | 1.54E-04 | 0.001708 |
| OTU2916 (Parafuscosporella_mucosa) | 0 | 8.30E-05 | 0 | 0 | 1.70E-05 | 0.001449 | 4.20E-04 | 2.48E-04 | 1.96E-04 | 3.75E-04 | 1.96E-04 |
| OTU3730 (Simplicillium_sympodiophorum) | 0 | 0 | 8.60E-05 | 0 | 0 | 2.24E-04 | 2.71E-04 | 1.29E-04 | 0 | 0.001132 | 1.94E-04 |
| OTU4126 (Saccharomyces_cerevisiae) | 0 | 4.80E-05 | 0 | 0 | 0 | 0 | 5.34E-04 | 1.23E-04 | 0.002617 | 2.84E-04 | 5.67E-04 |
| OTU4657 (Fungi_spe_Incertae_sedis) | 5.30E-05 | 4.20E-04 | 1.53E-04 | 5.70E-05 | 4.40E-05 | 0.01089 | 0.002677 | 0.0018 | 3.87E-04 | 0.005996 | 5.95E-04 |
| OTU5910 (Acrodictys_fluminicola) | 0 | 8.17E-04 | 0 | 0 | 4.40E-05 | 0.014764 | 0.002511 | 0.00223 | 6.75E-04 | 0.010768 | 0.001767 |
| OTU6661 (Zanclospora_jonesii) | 6.00E-05 | 1.28E-04 | 2.34E-04 | 4.38E-04 | 1.47E-04 | 0.001264 | 0.001553 | 8.22E-04 | 0.001039 | 4.50E-04 | 0.002475 |
| OTU6768 (Aspergillus_subflavus) | 0 | 0 | 0 | 0 | 0 | 0 | 1.15E-04 | 4.06E-04 | 0 | 0.002475 | 2.25E-04 |
| OTU7078 (Simplicillium_sympodiophorum) | 0.001946 | 0 | 0 | 5.70E-05 | 1.17E-04 | 0 | 0 | 0 | 0 | 0 | 0 |
| OTU7313 (Acrodictys_fluminicola) | 0 | 0 | 0 | 0 | 0 | 1.64E-04 | 6.30E-05 | 4.70E-04 | 0 | 0.005074 | 0.001209 |
| OTU7840 (Fungi_spe_Incertae_sedis) | 0 | 1.54E-04 | 7.90E-05 | 2.25E-04 | 0 | 4.16E-04 | 5.40E-04 | 4.40E-04 | 4.88E-04 | 1.08E-04 | 0.001146 |

| Data S17. Comparison of cytokines between the three groups | | | | | | |
| --- | --- | --- | --- | --- | --- | --- |
| ID | CPR0.median | CPR0.mean | CPR0.se | CPR1.median | CPR1.mean | CPR1.se |
| Fractalkine | 256.615(234.7375,284.9225) | 260.3290909 | 12.73502898 | 223.28(197.23,249.56) | 238.7944444 | 18.54672481 |
| IL-23 | 212.95(161.7175,257.325) | 212.5081818 | 18.4336912 | 149.23(99.71,229.89) | 179.3722222 | 21.01522074 |
| IL-4 | 33.835(30.1225,40.1625) | 35.61409091 | 2.164509448 | 34.69(30.705,40.17) | 34.58 | 1.698143615 |
| ITAC | 20.185(17.5225,24.94) | 24.92863636 | 3.020934196 | 24.17(19.35,37.72) | 38.43296296 | 6.472442632 |
| GM-CSF | 25.295(17.6025,28.7875) | 26.41636364 | 2.661278502 | 13.22(9.975,15.99) | 15.34888889 | 1.749393247 |
| MIP-3a | 25.155(22.4975,29.48) | 26.04318182 | 1.436467436 | 22.89(21.56,24.75) | 23.32703704 | 0.477826219 |
| MIP-1a | 20.11(17.445,21.735) | 19.81 | 1.078255566 | 17.84(16.02,19.02) | 17.66444444 | 0.434559191 |
| MIP-1b | 10.9(7.6875,12.385) | 12.00909091 | 1.592781187 | 20.27(15.415,27.765) | 24.36814815 | 2.821007025 |
| IFNy | 20.85(18.3225,21.9225) | 20.13363636 | 1.227985635 | 18.41(16.49,20.515) | 18.55740741 | 0.591903731 |
| IL-7 | 18.22(16.5575,19.44) | 18.00409091 | 0.555880667 | 20.39(18.94,22.055) | 20.56555556 | 0.551904585 |
| IL-10 | 15.28(10.47,21.085) | 17.05454545 | 1.970882384 | 8.74(6.03,12.86) | 11.45740741 | 2.141670514 |
| IL-8 | 2.125(1.57,2.5825) | 2.122272727 | 0.153308296 | 3.32(2.76,4.19) | 3.577407407 | 0.235980462 |
| IL-17A | 8.755(8.01,10.9125) | 9.636363636 | 0.89961982 | 5.92(5.03,8.495) | 7.107777778 | 0.548140169 |
| TNFa | 5.905(4.7025,6.9) | 5.94 | 0.35904515 | 6.1(4.805,7.145) | 6.06962963 | 0.279390098 |
| IL-13 | 3.6(2.0425,5.3725) | 4.070909091 | 0.557386802 | 4.54(3.16,7.35) | 5.490740741 | 0.580582262 |
| IL-2 | 4.18(3.215,5.0375) | 4.092272727 | 0.301938135 | 4.49(3.585,5.11) | 4.418888889 | 0.219871326 |
| IL-21 | 4.175(3.275,5.28) | 4.43 | 0.409972547 | 2.81(2.52,4.305) | 3.327407407 | 0.22764657 |
| IL-12(p70) | 3.1(2.08,3.69) | 3.334545455 | 0.461566469 | 3.62(2.905,4.02) | 4.597777778 | 1.342592035 |
| IL-5 | 3.93(3.2675,5.2775) | 4.225909091 | 0.376544243 | 3.41(2.45,4.005) | 3.257407407 | 0.207238382 |
| IL-6 | 1.51(1.1375,2.1225) | 2.091363636 | 0.575437833 | 2.09(1.34,2.495) | 2.767777778 | 0.946643092 |
| IL-1b | 2.365(2.02,2.59) | 2.365 | 0.16111187 | 1.89(1.61,2.16) | 1.865185185 | 0.063237378 |

| Data S17. Comparison of cytokines between the three groups | | | | | | | | |
| --- | --- | --- | --- | --- | --- | --- | --- | --- |
| ID | HC.median | HC.mean | HC.se | p-value | z-score | Sig_mark | q-value | fixp |
| Fractalkine | 227.825(184.7975,264.19) | 229.3414444 | 7.228247297 | 0.068065444 | -1.82457331 |  | 0.109951871 | 0.0681 |
| IL-23 | 149.92(65.3825,285.0025) | 218.5084444 | 24.29058938 | 0.195089401 | -1.295669419 |  | 0.256253824 | 0.1951 |
| IL-4 | 31.345(21.9025,42.575) | 33.53377778 | 1.650836935 | 0.372305994 | -0.892162211 |  | 0.411496098 | 0.3723 |
| ITAC | 25.19(17.0975,32.66) | 27.27955556 | 1.487336935 | 0.307757909 | -1.019937909 |  | 0.380171535 | 0.3078 |
| GM-CSF | 14.585(7.1325,25.86) | 25.83433333 | 4.288787003 | 0.003114633 | -2.956191877 | ** | 0.008175911 | 0.0031 |
| MIP-3a | 20.425(18.48,23.7825) | 21.74277778 | 0.771312742 | 3.38E-04 | -3.584295846 | *** | 0.001182961 | 0.0003 |
| MIP-1a | 19.08(16.1275,22.5675) | 23.12 | 1.503266074 | 0.170533124 | -1.370492792 |  | 0.255799686 | 0.1705 |
| MIP-1b | 20.295(12.4325,28.75) | 22.47488889 | 1.42496358 | 9.71E-05 | -3.897791426 | *** | 6.80E-04 | <0.0001 |
| IFNy | 18.155(13.995,23.48) | 18.735 | 0.692073947 | 0.40010336 | -0.841436652 |  | 0.420108528 | 0.4001 |
| IL-7 | 17.765(14.8725,19.945) | 16.97066667 | 0.447983129 | 1.82E-04 | -3.742781359 | *** | 8.53E-04 | 0.0002 |
| IL-10 | 6.61(2.7375,13.6325) | 9.201555556 | 0.816277625 | 2.03E-04 | -3.714998595 | *** | 8.53E-04 | 0.0002 |
| IL-8 | 4.725(2.855,13.525) | 13.56955556 | 2.908783289 | 5.92E-08 | -5.42109928 | *** | 1.24E-06 | <0.0001 |
| IL-17A | 6.45(4.64,10.32) | 7.689111111 | 0.446671827 | 0.025330249 | -2.236328675 | * | 0.048357749 | 0.0253 |
| TNFa | 4.96(4.3525,5.995) | 5.309111111 | 0.161371281 | 0.023310881 | -2.268300773 | * | 0.048357749 | 0.0233 |
| IL-13 | 5.035(2.82,6.72) | 5.456777778 | 0.537938262 | 0.195241009 | -1.29522968 |  | 0.256253824 | 0.1952 |
| IL-2 | 3.575(2.695,4.4675) | 3.714888889 | 0.176332398 | 0.012345758 | -2.502104213 | * | 0.028806768 | 0.0123 |
| IL-21 | 3.075(2.3175,4.285) | 3.496 | 0.195163544 | 0.049388824 | -1.965219618 | * | 0.086430443 | 0.0494 |
| IL-12(p70) | 3.13(1.9425,4.19) | 3.355777778 | 0.208545883 | 0.555868137 | -0.588989769 |  | 0.555868137 | 0.5559 |
| IL-5 | 2.67(1.77,3.61) | 2.768555556 | 0.16089121 | 6.02E-04 | -3.430850715 | *** | 0.001805075 | 0.0006 |
| IL-6 | 1.585(0.87,2.5) | 2.091555556 | 0.278954796 | 0.365443344 | -0.905041609 |  | 0.411496098 | 0.3654 |
| IL-1b | 1.49(1.24,1.9975) | 1.610777778 | 0.06130341 | 5.13E-06 | -4.559452005 | *** | 5.39E-05 | <0.0001 |

| Data S18. Spearman correlation analysis of oral fungi, fecal fungi and clinical indicators | | | | | | | | | |
| --- | --- | --- | --- | --- | --- | --- | --- | --- | --- |
| from | to | rho | p | rho_pn | rho_abs | rank | p_0.05 | p_0.01 | p_lg |
| O_OTU35 (Zanclospora_jonesii) | O_OTU5 (Actinomucor_elegans) | 0.052721 | 0.6005526 | positive | 0.05272072 | 0 | 0 | 0 | 0.22145 |
| O_OTU50 (Mucor_circinelloides) | O_OTU5 (Actinomucor_elegans) | 0.558982 | 1.2383E-09 | positive | 0.55898209 | 0.5 | 1 | 2 | 7.9493 |
| O_OTU79 (Phaeosphaeria_oryzae) | O_OTU5 (Actinomucor_elegans) | -0.235625 | 0.01769071 | negative | 0.23562513 | 0 | 1 | 1 | 1.75225 |
| O_OTU91 (Aspergillus_subflavus) | O_OTU5 (Actinomucor_elegans) | -0.166091 | 0.09692048 | negative | 0.1660912 | 0 | 0 | 0 | 1.01358 |
| F_OTU17 (Acrodictys_fluminicola) | O_OTU5 (Actinomucor_elegans) | -0.113364 | 0.25900133 | negative | 0.11336386 | 0 | 0 | 0 | 0.5867 |
| F_OTU69 (Apiotrichum_domesticum) | O_OTU5 (Actinomucor_elegans) | -0.27481 | 0.00541607 | negative | 0.27480968 | 0 | 1 | 2 | 2.26631 |
| F_OTU3128 (Sporothrix_dombeyi) | O_OTU5 (Actinomucor_elegans) | -0.132156 | 0.18770226 | negative | 0.1321561 | 0 | 0 | 0 | 0.72653 |
| F_OTU5539 (Fungi_spe_Incertae_sedis) | O_OTU5 (Actinomucor_elegans) | -0.459779 | 1.3188E-06 | negative | 0.45977948 | 0.3 | 1 | 2 | 5.87655 |
| F_OTU7821 (Acrodictys_fluminicola) | O_OTU5 (Actinomucor_elegans) | -0.233214 | 0.01892217 | negative | 0.23321406 | 0 | 1 | 1 | 1.72303 |
| F_OTU7828 (Acrodictys_fluminicola) | O_OTU5 (Actinomucor_elegans) | -0.359011 | 0.00022709 | negative | 0.35901051 | 0.3 | 1 | 2 | 3.64379 |
| O_OTU13 (Cryptococcus_longus) | O_OTU5 (Actinomucor_elegans) | -0.283243 | 0.00410285 | negative | 0.28324262 | 0 | 1 | 2 | 2.38691 |
| O_OTU29 (Blumeria_spe_Incertae_sedis) | O_OTU5 (Actinomucor_elegans) | -0.177831 | 0.07521688 | negative | 0.17783062 | 0 | 0 | 0 | 1.12368 |
| O_OTU30 (Aspergillus_subflavus) | O_OTU5 (Actinomucor_elegans) | -0.128275 | 0.20111364 | negative | 0.12827452 | 0 | 0 | 0 | 0.69656 |
| O_OTU33 (Aspergillus_subflavus) | O_OTU5 (Actinomucor_elegans) | 0.054356 | 0.5892834 | positive | 0.0543564 | 0 | 0 | 0 | 0.22968 |
| O_OTU44 (Zanclospora_jonesii) | O_OTU5 (Actinomucor_elegans) | -0.296416 | 0.00261407 | negative | 0.29641627 | 0 | 1 | 2 | 2.58268 |
| O_OTU95 (Zanclospora_jonesii) | O_OTU5 (Actinomucor_elegans) | -0.305554 | 0.00188854 | negative | 0.30555378 | 0.3 | 1 | 2 | 2.72387 |
| O_OTU154 (Diversispora_spurca) | O_OTU5 (Actinomucor_elegans) | -0.326144 | 0.00087344 | negative | 0.32614389 | 0.3 | 1 | 2 | 3.05876 |
| O_OTU599 (Aspergillus_subflavus) | O_OTU5 (Actinomucor_elegans) | 0.311743 | 0.00150635 | positive | 0.31174307 | 0.3 | 1 | 2 | 2.82207 |
| O_OTU1734 (Acrodictys_fluminicola) | O_OTU5 (Actinomucor_elegans) | -0.218117 | 0.02843603 | negative | 0.21811693 | 0 | 1 | 1 | 1.54613 |
| O_OTU5905 (Blumeria_spe_Incertae_sedis) | O_OTU5 (Actinomucor_elegans) | -0.2122 | 0.03314122 | negative | 0.21220045 | 0 | 1 | 1 | 1.47963 |
| O_OTU5962 (Acrodictys_fluminicola) | O_OTU5 (Actinomucor_elegans) | -0.16564 | 0.09784364 | negative | 0.16564016 | 0 | 0 | 0 | 1.00947 |
| O_OTU7942 (Fungi_spe_Incertae_sedis) | O_OTU5 (Actinomucor_elegans) | -0.228676 | 0.02144101 | negative | 0.22867605 | 0 | 1 | 1 | 1.66875 |
| O_OTU8123 (Heitmania_litseae) | O_OTU5 (Actinomucor_elegans) | -0.208711 | 0.03621233 | negative | 0.20871085 | 0 | 1 | 1 | 1.44114 |
| Neutralizing_antibody | O_OTU5 (Actinomucor_elegans) | 0.266806 | 0.00699539 | positive | 0.26680568 | 0 | 1 | 2 | 2.15519 |
| lgM | O_OTU5 (Actinomucor_elegans) | 0.200771 | 0.04409856 | positive | 0.20077066 | 0 | 1 | 1 | 1.35558 |
| lgG | O_OTU5 (Actinomucor_elegans) | 0.257314 | 0.00938543 | positive | 0.25731441 | 0 | 1 | 2 | 2.02755 |
| MON# | O_OTU5 (Actinomucor_elegans) | 0.200671 | 0.04420576 | positive | 0.20067117 | 0 | 1 | 1 | 1.35452 |
| MON_p.c. | O_OTU5 (Actinomucor_elegans) | 0.160728 | 0.10835058 | positive | 0.1607283 | 0 | 0 | 0 | 0.96517 |
| IBIL | O_OTU5 (Actinomucor_elegans) | 0.292003 | 0.00304732 | positive | 0.29200278 | 0 | 1 | 2 | 2.51608 |
| TP | O_OTU5 (Actinomucor_elegans) | 0.476058 | 4.8627E-07 | positive | 0.47605804 | 0.3 | 1 | 2 | 6.30428 |
| ALB | O_OTU5 (Actinomucor_elegans) | 0.491403 | 1.8089E-07 | positive | 0.49140344 | 0.3 | 1 | 2 | 6.71921 |
| GLOB | O_OTU5 (Actinomucor_elegans) | 0.356416 | 0.00025394 | positive | 0.35641567 | 0.3 | 1 | 2 | 3.59525 |
| TBA | O_OTU5 (Actinomucor_elegans) | -0.445095 | 3.1067E-06 | negative | 0.44509544 | 0.3 | 1 | 2 | 5.50631 |
| F_OTU3924 (Saccharomyces_cerevisiae) | O_OTU5 (Actinomucor_elegans) | 0.134987 | 0.17834004 | positive | 0.13498682 | 0 | 0 | 0 | 0.74875 |
| F_OTU6998 (Calosphaeria_pulchella) | O_OTU5 (Actinomucor_elegans) | -0.086571 | 0.38934024 | negative | 0.08657085 | 0 | 0 | 0 | 0.40967 |
| O_OTU50 (Mucor_circinelloides) | O_OTU35 (Zanclospora_jonesii) | 0.419864 | 1.2378E-05 | positive | 0.41986389 | 0.3 | 1 | 2 | 4.907 |
| O_OTU79 (Phaeosphaeria_oryzae) | O_OTU35 (Zanclospora_jonesii) | -0.098336 | 0.32790623 | negative | 0.09833629 | 0 | 0 | 0 | 0.48425 |
| O_OTU91 (Aspergillus_subflavus) | O_OTU35 (Zanclospora_jonesii) | 0.207002 | 0.0378009 | positive | 0.20700241 | 0 | 1 | 1 | 1.4225 |
| F_OTU17 (Acrodictys_fluminicola) | O_OTU35 (Zanclospora_jonesii) | 0.467833 | 8.1021E-07 | positive | 0.46783315 | 0.3 | 1 | 2 | 6.08608 |
| F_OTU69 (Apiotrichum_domesticum) | O_OTU35 (Zanclospora_jonesii) | -0.1503 | 0.13355469 | negative | 0.15029997 | 0 | 0 | 0 | 0.87434 |
| F_OTU3128 (Sporothrix_dombeyi) | O_OTU35 (Zanclospora_jonesii) | 0.187695 | 0.06016676 | positive | 0.18769464 | 0 | 0 | 0 | 1.22064 |
| F_OTU5539 (Fungi_spe_Incertae_sedis) | O_OTU35 (Zanclospora_jonesii) | 0.187813 | 0.06000241 | positive | 0.18781295 | 0 | 0 | 0 | 1.22183 |
| F_OTU7821 (Acrodictys_fluminicola) | O_OTU35 (Zanclospora_jonesii) | -0.13463 | 0.17950213 | negative | 0.13462964 | 0 | 0 | 0 | 0.74593 |
| F_OTU7828 (Acrodictys_fluminicola) | O_OTU35 (Zanclospora_jonesii) | -0.181467 | 0.0693499 | negative | 0.18146729 | 0 | 0 | 0 | 1.15895 |
| O_OTU13 (Cryptococcus_longus) | O_OTU35 (Zanclospora_jonesii) | 0.427348 | 8.3091E-06 | positive | 0.42734848 | 0.3 | 1 | 2 | 5.07993 |
| O_OTU29 (Blumeria_spe_Incertae_sedis) | O_OTU35 (Zanclospora_jonesii) | 0.248617 | 0.01217714 | positive | 0.24861748 | 0 | 1 | 1 | 1.91445 |
| O_OTU30 (Aspergillus_subflavus) | O_OTU35 (Zanclospora_jonesii) | 0.211074 | 0.03410759 | positive | 0.21107407 | 0 | 1 | 1 | 1.46715 |
| O_OTU33 (Aspergillus_subflavus) | O_OTU35 (Zanclospora_jonesii) | 0.278026 | 0.00487662 | positive | 0.27802587 | 0 | 1 | 2 | 2.31188 |
| O_OTU44 (Zanclospora_jonesii) | O_OTU35 (Zanclospora_jonesii) | 0.188702 | 0.05877857 | positive | 0.18870239 | 0 | 0 | 0 | 1.23078 |
| O_OTU95 (Zanclospora_jonesii) | O_OTU35 (Zanclospora_jonesii) | 0.204405 | 0.04032796 | positive | 0.2044047 | 0 | 1 | 1 | 1.39439 |
| O_OTU154 (Diversispora_spurca) | O_OTU35 (Zanclospora_jonesii) | 0.037171 | 0.71210036 | positive | 0.0371705 | 0 | 0 | 0 | 0.14746 |
| O_OTU599 (Aspergillus_subflavus) | O_OTU35 (Zanclospora_jonesii) | 0.459112 | 1.3723E-06 | positive | 0.45911184 | 0.3 | 1 | 2 | 5.85938 |
| O_OTU1734 (Acrodictys_fluminicola) | O_OTU35 (Zanclospora_jonesii) | 0.023137 | 0.81835803 | positive | 0.02313669 | 0 | 0 | 0 | 0.08706 |
| O_OTU5905 (Blumeria_spe_Incertae_sedis) | O_OTU35 (Zanclospora_jonesii) | 0.224521 | 0.02399394 | positive | 0.22452137 | 0 | 1 | 1 | 1.6199 |
| O_OTU5962 (Acrodictys_fluminicola) | O_OTU35 (Zanclospora_jonesii) | 0.270577 | 0.00620653 | positive | 0.27057724 | 0 | 1 | 2 | 2.20715 |
| O_OTU7942 (Fungi_spe_Incertae_sedis) | O_OTU35 (Zanclospora_jonesii) | 0.127738 | 0.20301891 | positive | 0.12773837 | 0 | 0 | 0 | 0.69246 |
| O_OTU8123 (Heitmania_litseae) | O_OTU35 (Zanclospora_jonesii) | 0.286626 | 0.00366165 | positive | 0.28662566 | 0 | 1 | 2 | 2.43632 |
| Neutralizing_antibody | O_OTU35 (Zanclospora_jonesii) | -0.303256 | 0.0020514 | negative | 0.30325626 | 0.3 | 1 | 2 | 2.68795 |
| lgM | O_OTU35 (Zanclospora_jonesii) | -0.250903 | 0.0113809 | negative | 0.25090325 | 0 | 1 | 1 | 1.94382 |
| lgG | O_OTU35 (Zanclospora_jonesii) | -0.28105 | 0.00441361 | negative | 0.28104993 | 0 | 1 | 2 | 2.35521 |
| MON# | O_OTU35 (Zanclospora_jonesii) | 0.52702 | 1.4985E-08 | positive | 0.52702043 | 0.5 | 1 | 2 | 7.60232 |
| MON_p.c. | O_OTU35 (Zanclospora_jonesii) | 0.498361 | 1.1368E-07 | positive | 0.4983614 | 0.3 | 1 | 2 | 6.9077 |
| IBIL | O_OTU35 (Zanclospora_jonesii) | -0.330756 | 0.00072937 | negative | 0.33075619 | 0.3 | 1 | 2 | 3.13705 |
| TP | O_OTU35 (Zanclospora_jonesii) | 0.069853 | 0.48760241 | positive | 0.06985329 | 0 | 0 | 0 | 0.31193 |
| ALB | O_OTU35 (Zanclospora_jonesii) | -0.120314 | 0.2307406 | negative | 0.12031441 | 0 | 0 | 0 | 0.63688 |
| GLOB | O_OTU35 (Zanclospora_jonesii) | 0.208947 | 0.03599722 | positive | 0.208947 | 0 | 1 | 1 | 1.44373 |
| TBA | O_OTU35 (Zanclospora_jonesii) | 0.062891 | 0.53210362 | positive | 0.06289096 | 0 | 0 | 0 | 0.274 |
| F_OTU3924 (Saccharomyces_cerevisiae) | O_OTU35 (Zanclospora_jonesii) | 0.280652 | 0.00447216 | positive | 0.28065228 | 0 | 1 | 2 | 2.34948 |
| F_OTU6998 (Calosphaeria_pulchella) | O_OTU35 (Zanclospora_jonesii) | -0.347987 | 0.00036271 | negative | 0.34798734 | 0.3 | 1 | 2 | 3.44042 |
| O_OTU79 (Phaeosphaeria_oryzae) | O_OTU50 (Mucor_circinelloides) | -0.198687 | 0.04638947 | negative | 0.19868716 | 0 | 1 | 1 | 1.33358 |
| O_OTU91 (Aspergillus_subflavus) | O_OTU50 (Mucor_circinelloides) | -0.076647 | 0.44616505 | negative | 0.07664676 | 0 | 0 | 0 | 0.3505 |
| F_OTU17 (Acrodictys_fluminicola) | O_OTU50 (Mucor_circinelloides) | 0.237522 | 0.01677088 | positive | 0.23752245 | 0 | 1 | 1 | 1.77544 |
| F_OTU69 (Apiotrichum_domesticum) | O_OTU50 (Mucor_circinelloides) | -0.10481 | 0.29690588 | negative | 0.1048099 | 0 | 0 | 0 | 0.52738 |
| F_OTU3128 (Sporothrix_dombeyi) | O_OTU50 (Mucor_circinelloides) | -0.135503 | 0.17667053 | negative | 0.13550296 | 0 | 0 | 0 | 0.75284 |
| F_OTU5539 (Fungi_spe_Incertae_sedis) | O_OTU50 (Mucor_circinelloides) | -0.294081 | 0.00283589 | negative | 0.29408077 | 0 | 1 | 2 | 2.54731 |
| F_OTU7821 (Acrodictys_fluminicola) | O_OTU50 (Mucor_circinelloides) | -0.432151 | 6.4016E-06 | negative | 0.43215118 | 0.3 | 1 | 2 | 5.19303 |
| F_OTU7828 (Acrodictys_fluminicola) | O_OTU50 (Mucor_circinelloides) | -0.429512 | 7.3918E-06 | negative | 0.42951183 | 0.3 | 1 | 2 | 5.13067 |
| O_OTU13 (Cryptococcus_longus) | O_OTU50 (Mucor_circinelloides) | -0.020173 | 0.84130191 | negative | 0.02017254 | 0 | 0 | 0 | 0.07505 |
| O_OTU29 (Blumeria_spe_Incertae_sedis) | O_OTU50 (Mucor_circinelloides) | 0.127306 | 0.20456304 | positive | 0.12730649 | 0 | 0 | 0 | 0.68917 |
| O_OTU30 (Aspergillus_subflavus) | O_OTU50 (Mucor_circinelloides) | 0.192735 | 0.05348341 | positive | 0.19273484 | 0 | 0 | 0 | 1.27178 |
| O_OTU33 (Aspergillus_subflavus) | O_OTU50 (Mucor_circinelloides) | 0.356541 | 0.00025258 | positive | 0.35654063 | 0.3 | 1 | 2 | 3.59758 |
| O_OTU44 (Zanclospora_jonesii) | O_OTU50 (Mucor_circinelloides) | -0.238299 | 0.01640673 | negative | 0.23829851 | 0 | 1 | 1 | 1.78498 |
| O_OTU95 (Zanclospora_jonesii) | O_OTU50 (Mucor_circinelloides) | -0.114807 | 0.25294693 | negative | 0.11480716 | 0 | 0 | 0 | 0.59697 |
| O_OTU154 (Diversispora_spurca) | O_OTU50 (Mucor_circinelloides) | -0.253482 | 0.01053753 | negative | 0.25348216 | 0 | 1 | 1 | 1.97726 |
| O_OTU599 (Aspergillus_subflavus) | O_OTU50 (Mucor_circinelloides) | 0.66516 | 3.2863E-14 | positive | 0.66515963 | 0.5 | 1 | 2 | 8 |
| O_OTU1734 (Acrodictys_fluminicola) | O_OTU50 (Mucor_circinelloides) | -0.054986 | 0.58496997 | negative | 0.05498621 | 0 | 0 | 0 | 0.23287 |
| O_OTU5905 (Blumeria_spe_Incertae_sedis) | O_OTU50 (Mucor_circinelloides) | 0.149415 | 0.13588372 | positive | 0.149415 | 0 | 0 | 0 | 0.86683 |
| O_OTU5962 (Acrodictys_fluminicola) | O_OTU50 (Mucor_circinelloides) | 0.00032 | 0.99746851 | positive | 0.00031968 | 0 | 0 | 0 | 0.0011 |
| O_OTU7942 (Fungi_spe_Incertae_sedis) | O_OTU50 (Mucor_circinelloides) | -0.191625 | 0.05489949 | negative | 0.19162545 | 0 | 0 | 0 | 1.26043 |
| O_OTU8123 (Heitmania_litseae) | O_OTU50 (Mucor_circinelloides) | -0.008916 | 0.92948293 | negative | 0.00891643 | 0 | 0 | 0 | 0.03176 |
| Neutralizing_antibody | O_OTU50 (Mucor_circinelloides) | -0.096007 | 0.33954773 | negative | 0.09600723 | 0 | 0 | 0 | 0.4691 |
| lgM | O_OTU50 (Mucor_circinelloides) | 0.017734 | 0.860279 | positive | 0.01773391 | 0 | 0 | 0 | 0.06536 |
| lgG | O_OTU50 (Mucor_circinelloides) | -0.019802 | 0.84418078 | negative | 0.01980188 | 0 | 0 | 0 | 0.07356 |
| MON# | O_OTU50 (Mucor_circinelloides) | 0.446918 | 2.7992E-06 | positive | 0.44691818 | 0.3 | 1 | 2 | 5.55141 |
| MON_p.c. | O_OTU50 (Mucor_circinelloides) | 0.512023 | 4.4288E-08 | positive | 0.5120225 | 0.5 | 1 | 2 | 7.26529 |
| IBIL | O_OTU50 (Mucor_circinelloides) | 0.10125 | 0.31370512 | positive | 0.1012502 | 0 | 0 | 0 | 0.50348 |
| TP | O_OTU50 (Mucor_circinelloides) | 0.352023 | 0.00030617 | positive | 0.35202277 | 0.3 | 1 | 2 | 3.51402 |
| ALB | O_OTU50 (Mucor_circinelloides) | 0.270405 | 0.00624082 | positive | 0.27040471 | 0 | 1 | 2 | 2.20476 |
| GLOB | O_OTU50 (Mucor_circinelloides) | 0.351549 | 0.00031236 | positive | 0.35154936 | 0.3 | 1 | 2 | 3.50533 |
| TBA | O_OTU50 (Mucor_circinelloides) | -0.122618 | 0.22186968 | negative | 0.1226179 | 0 | 0 | 0 | 0.6539 |
| F_OTU3924 (Saccharomyces_cerevisiae) | O_OTU50 (Mucor_circinelloides) | 0.338232 | 0.00054134 | positive | 0.33823161 | 0.3 | 1 | 2 | 3.26652 |
| F_OTU6998 (Calosphaeria_pulchella) | O_OTU50 (Mucor_circinelloides) | -0.309415 | 0.001641 | negative | 0.30941506 | 0.3 | 1 | 2 | 2.78489 |
| O_OTU91 (Aspergillus_subflavus) | O_OTU79 (Phaeosphaeria_oryzae) | 0.043784 | 0.66373938 | positive | 0.0437841 | 0 | 0 | 0 | 0.178 |
| F_OTU17 (Acrodictys_fluminicola) | O_OTU79 (Phaeosphaeria_oryzae) | -0.104269 | 0.29942087 | negative | 0.10426874 | 0 | 0 | 0 | 0.52372 |
| F_OTU69 (Apiotrichum_domesticum) | O_OTU79 (Phaeosphaeria_oryzae) | 0.468767 | 7.6509E-07 | positive | 0.46876695 | 0.3 | 1 | 2 | 6.11065 |
| F_OTU3128 (Sporothrix_dombeyi) | O_OTU79 (Phaeosphaeria_oryzae) | 0.187471 | 0.06047884 | positive | 0.1874707 | 0 | 0 | 0 | 1.2184 |
| F_OTU5539 (Fungi_spe_Incertae_sedis) | O_OTU79 (Phaeosphaeria_oryzae) | 0.182586 | 0.0676216 | positive | 0.18258591 | 0 | 0 | 0 | 1.16991 |
| F_OTU7821 (Acrodictys_fluminicola) | O_OTU79 (Phaeosphaeria_oryzae) | -0.030235 | 0.76406984 | negative | 0.03023463 | 0 | 0 | 0 | 0.11687 |
| F_OTU7828 (Acrodictys_fluminicola) | O_OTU79 (Phaeosphaeria_oryzae) | -0.012853 | 0.89849412 | negative | 0.01285254 | 0 | 0 | 0 | 0.04648 |
| O_OTU13 (Cryptococcus_longus) | O_OTU79 (Phaeosphaeria_oryzae) | 0.217124 | 0.02918385 | positive | 0.2171237 | 0 | 1 | 1 | 1.53486 |
| O_OTU29 (Blumeria_spe_Incertae_sedis) | O_OTU79 (Phaeosphaeria_oryzae) | 0.212778 | 0.03265481 | positive | 0.2127779 | 0 | 1 | 1 | 1.48605 |
| O_OTU30 (Aspergillus_subflavus) | O_OTU79 (Phaeosphaeria_oryzae) | 0.185077 | 0.06389869 | positive | 0.18507655 | 0 | 0 | 0 | 1.19451 |
| O_OTU33 (Aspergillus_subflavus) | O_OTU79 (Phaeosphaeria_oryzae) | 0.059104 | 0.55713082 | positive | 0.05910406 | 0 | 0 | 0 | 0.25404 |
| O_OTU44 (Zanclospora_jonesii) | O_OTU79 (Phaeosphaeria_oryzae) | 0.094917 | 0.34508406 | positive | 0.09491731 | 0 | 0 | 0 | 0.46208 |
| O_OTU95 (Zanclospora_jonesii) | O_OTU79 (Phaeosphaeria_oryzae) | 0.126123 | 0.20883851 | positive | 0.12612271 | 0 | 0 | 0 | 0.68019 |
| O_OTU154 (Diversispora_spurca) | O_OTU79 (Phaeosphaeria_oryzae) | 0.478262 | 4.2313E-07 | positive | 0.47826227 | 0.3 | 1 | 2 | 6.36338 |
| O_OTU599 (Aspergillus_subflavus) | O_OTU79 (Phaeosphaeria_oryzae) | -0.235955 | 0.01752795 | negative | 0.23595454 | 0 | 1 | 1 | 1.75627 |
| O_OTU1734 (Acrodictys_fluminicola) | O_OTU79 (Phaeosphaeria_oryzae) | -0.010242 | 0.91903362 | negative | 0.01024188 | 0 | 0 | 0 | 0.03667 |
| O_OTU5905 (Blumeria_spe_Incertae_sedis) | O_OTU79 (Phaeosphaeria_oryzae) | 0.209301 | 0.03567641 | positive | 0.2093014 | 0 | 1 | 1 | 1.44762 |
| O_OTU5962 (Acrodictys_fluminicola) | O_OTU79 (Phaeosphaeria_oryzae) | 0.003872 | 0.96934813 | positive | 0.0038717 | 0 | 0 | 0 | 0.01352 |
| O_OTU7942 (Fungi_spe_Incertae_sedis) | O_OTU79 (Phaeosphaeria_oryzae) | 0.1107 | 0.2704328 | positive | 0.11070019 | 0 | 0 | 0 | 0.56794 |
| O_OTU8123 (Heitmania_litseae) | O_OTU79 (Phaeosphaeria_oryzae) | 0.359015 | 0.00022705 | positive | 0.35901459 | 0.3 | 1 | 2 | 3.64386 |
| Neutralizing_antibody | O_OTU79 (Phaeosphaeria_oryzae) | -0.24924 | 0.01195549 | negative | 0.24924033 | 0 | 1 | 1 | 1.92243 |
| lgM | O_OTU79 (Phaeosphaeria_oryzae) | -0.119618 | 0.23347059 | negative | 0.11961803 | 0 | 0 | 0 | 0.63177 |
| lgG | O_OTU79 (Phaeosphaeria_oryzae) | -0.325986 | 0.00087878 | negative | 0.32598647 | 0.3 | 1 | 2 | 3.05611 |
| MON# | O_OTU79 (Phaeosphaeria_oryzae) | -0.0772 | 0.44287958 | negative | 0.07720004 | 0 | 0 | 0 | 0.35371 |
| MON_p.c. | O_OTU79 (Phaeosphaeria_oryzae) | -0.0791 | 0.43170206 | negative | 0.07909991 | 0 | 0 | 0 | 0.36482 |
| IBIL | O_OTU79 (Phaeosphaeria_oryzae) | -0.203592 | 0.04114721 | negative | 0.20359174 | 0 | 1 | 1 | 1.38566 |
| TP | O_OTU79 (Phaeosphaeria_oryzae) | -0.367579 | 0.00015594 | negative | 0.36757881 | 0.3 | 1 | 2 | 3.80702 |
| ALB | O_OTU79 (Phaeosphaeria_oryzae) | -0.379582 | 9.0466E-05 | negative | 0.37958177 | 0.3 | 1 | 2 | 4.04347 |
| GLOB | O_OTU79 (Phaeosphaeria_oryzae) | -0.312794 | 0.00144889 | negative | 0.31279437 | 0.3 | 1 | 2 | 2.83896 |
| TBA | O_OTU79 (Phaeosphaeria_oryzae) | 0.093128 | 0.35429275 | positive | 0.09312847 | 0 | 0 | 0 | 0.45064 |
| F_OTU3924 (Saccharomyces_cerevisiae) | O_OTU79 (Phaeosphaeria_oryzae) | -0.202934 | 0.04182048 | negative | 0.20293369 | 0 | 1 | 1 | 1.37861 |
| F_OTU6998 (Calosphaeria_pulchella) | O_OTU79 (Phaeosphaeria_oryzae) | -0.005539 | 0.95615697 | negative | 0.0055393 | 0 | 0 | 0 | 0.01947 |
| F_OTU17 (Acrodictys_fluminicola) | O_OTU91 (Aspergillus_subflavus) | 0.102671 | 0.30692538 | positive | 0.10267145 | 0 | 0 | 0 | 0.51297 |
| F_OTU69 (Apiotrichum_domesticum) | O_OTU91 (Aspergillus_subflavus) | -0.16345 | 0.10242513 | negative | 0.16344995 | 0 | 0 | 0 | 0.98959 |
| F_OTU3128 (Sporothrix_dombeyi) | O_OTU91 (Aspergillus_subflavus) | 0.401931 | 3.0974E-05 | positive | 0.40193087 | 0.3 | 1 | 2 | 4.50886 |
| F_OTU5539 (Fungi_spe_Incertae_sedis) | O_OTU91 (Aspergillus_subflavus) | 0.360372 | 0.00021407 | positive | 0.36037242 | 0.3 | 1 | 2 | 3.66943 |
| F_OTU7821 (Acrodictys_fluminicola) | O_OTU91 (Aspergillus_subflavus) | 0.203492 | 0.04124905 | positive | 0.20349163 | 0 | 1 | 1 | 1.38459 |
| F_OTU7828 (Acrodictys_fluminicola) | O_OTU91 (Aspergillus_subflavus) | 0.111663 | 0.26626365 | positive | 0.11166264 | 0 | 0 | 0 | 0.57469 |
| O_OTU13 (Cryptococcus_longus) | O_OTU91 (Aspergillus_subflavus) | 0.310333 | 0.00158668 | positive | 0.31033261 | 0.3 | 1 | 2 | 2.79951 |
| O_OTU29 (Blumeria_spe_Incertae_sedis) | O_OTU91 (Aspergillus_subflavus) | 0.334661 | 0.00062476 | positive | 0.33466118 | 0.3 | 1 | 2 | 3.20428 |
| O_OTU30 (Aspergillus_subflavus) | O_OTU91 (Aspergillus_subflavus) | 0.303214 | 0.00205452 | positive | 0.30321388 | 0.3 | 1 | 2 | 2.68729 |
| O_OTU33 (Aspergillus_subflavus) | O_OTU91 (Aspergillus_subflavus) | 0.050712 | 0.61451945 | positive | 0.0507123 | 0 | 0 | 0 | 0.21146 |
| O_OTU44 (Zanclospora_jonesii) | O_OTU91 (Aspergillus_subflavus) | 0.521985 | 2.1688E-08 | positive | 0.52198468 | 0.5 | 1 | 2 | 7.4991 |
| O_OTU95 (Zanclospora_jonesii) | O_OTU91 (Aspergillus_subflavus) | 0.412114 | 1.8519E-05 | positive | 0.41211359 | 0.3 | 1 | 2 | 4.73215 |
| O_OTU154 (Diversispora_spurca) | O_OTU91 (Aspergillus_subflavus) | 0.359942 | 0.00021811 | positive | 0.35994164 | 0.3 | 1 | 2 | 3.6613 |
| O_OTU599 (Aspergillus_subflavus) | O_OTU91 (Aspergillus_subflavus) | 0.175829 | 0.07861226 | positive | 0.17582913 | 0 | 0 | 0 | 1.10451 |
| O_OTU1734 (Acrodictys_fluminicola) | O_OTU91 (Aspergillus_subflavus) | 0.266748 | 0.00700804 | positive | 0.26674835 | 0 | 1 | 2 | 2.1544 |
| O_OTU5905 (Blumeria_spe_Incertae_sedis) | O_OTU91 (Aspergillus_subflavus) | 0.272764 | 0.00578628 | positive | 0.27276356 | 0 | 1 | 2 | 2.2376 |
| O_OTU5962 (Acrodictys_fluminicola) | O_OTU91 (Aspergillus_subflavus) | 0.162857 | 0.10369434 | positive | 0.16285682 | 0 | 0 | 0 | 0.98424 |
| O_OTU7942 (Fungi_spe_Incertae_sedis) | O_OTU91 (Aspergillus_subflavus) | 0.130429 | 0.19358721 | positive | 0.13042896 | 0 | 0 | 0 | 0.71312 |
| O_OTU8123 (Heitmania_litseae) | O_OTU91 (Aspergillus_subflavus) | 0.221723 | 0.02585593 | positive | 0.22172326 | 0 | 1 | 1 | 1.58744 |
| Neutralizing_antibody | O_OTU91 (Aspergillus_subflavus) | -0.164622 | 0.09995344 | negative | 0.16462177 | 0 | 0 | 0 | 1.0002 |
| lgM | O_OTU91 (Aspergillus_subflavus) | -0.148936 | 0.13715665 | negative | 0.14893619 | 0 | 0 | 0 | 0.86278 |
| lgG | O_OTU91 (Aspergillus_subflavus) | -0.233139 | 0.01896159 | negative | 0.23313914 | 0 | 1 | 1 | 1.72213 |
| MON# | O_OTU91 (Aspergillus_subflavus) | -0.058756 | 0.55945913 | negative | 0.058756 | 0 | 0 | 0 | 0.25223 |
| MON_p.c. | O_OTU91 (Aspergillus_subflavus) | -0.082225 | 0.41367181 | negative | 0.08222484 | 0 | 0 | 0 | 0.38334 |
| IBIL | O_OTU91 (Aspergillus_subflavus) | -0.362005 | 0.00019937 | negative | 0.36200533 | 0.3 | 1 | 2 | 3.70032 |
| TP | O_OTU91 (Aspergillus_subflavus) | -0.305053 | 0.00192304 | negative | 0.30505253 | 0.3 | 1 | 2 | 2.71601 |
| ALB | O_OTU91 (Aspergillus_subflavus) | -0.383866 | 7.4103E-05 | negative | 0.3838655 | 0.3 | 1 | 2 | 4.1301 |
| GLOB | O_OTU91 (Aspergillus_subflavus) | -0.235829 | 0.01759001 | negative | 0.23582863 | 0 | 1 | 1 | 1.75473 |
| TBA | O_OTU91 (Aspergillus_subflavus) | 0.231854 | 0.0196491 | positive | 0.23185357 | 0 | 1 | 1 | 1.70666 |
| F_OTU3924 (Saccharomyces_cerevisiae) | O_OTU91 (Aspergillus_subflavus) | 0.162073 | 0.10538957 | positive | 0.16207339 | 0 | 0 | 0 | 0.9772 |
| F_OTU6998 (Calosphaeria_pulchella) | O_OTU91 (Aspergillus_subflavus) | 0.027163 | 0.78743745 | positive | 0.02716331 | 0 | 0 | 0 | 0.10378 |
| F_OTU69 (Apiotrichum_domesticum) | F_OTU17 (Acrodictys_fluminicola) | -0.02264 | 0.82219537 | negative | 0.02263963 | 0 | 0 | 0 | 0.08502 |
| F_OTU3128 (Sporothrix_dombeyi) | F_OTU17 (Acrodictys_fluminicola) | -0.101956 | 0.31032808 | negative | 0.10195561 | 0 | 0 | 0 | 0.50818 |
| F_OTU5539 (Fungi_spe_Incertae_sedis) | F_OTU17 (Acrodictys_fluminicola) | 0.021457 | 0.8313413 | positive | 0.02145711 | 0 | 0 | 0 | 0.08022 |
| F_OTU7821 (Acrodictys_fluminicola) | F_OTU17 (Acrodictys_fluminicola) | -0.301675 | 0.00217072 | negative | 0.30167529 | 0.3 | 1 | 2 | 2.66339 |
| F_OTU7828 (Acrodictys_fluminicola) | F_OTU17 (Acrodictys_fluminicola) | -0.203618 | 0.04112103 | negative | 0.20361751 | 0 | 1 | 1 | 1.38594 |
| O_OTU13 (Cryptococcus_longus) | F_OTU17 (Acrodictys_fluminicola) | 0.274413 | 0.00548616 | positive | 0.27441296 | 0 | 1 | 2 | 2.26073 |
| O_OTU29 (Blumeria_spe_Incertae_sedis) | F_OTU17 (Acrodictys_fluminicola) | 0.2765 | 0.00512629 | positive | 0.27649994 | 0 | 1 | 2 | 2.2902 |
| O_OTU30 (Aspergillus_subflavus) | F_OTU17 (Acrodictys_fluminicola) | 0.224784 | 0.02382529 | positive | 0.22478387 | 0 | 1 | 1 | 1.62296 |
| O_OTU33 (Aspergillus_subflavus) | F_OTU17 (Acrodictys_fluminicola) | 0.308816 | 0.00167738 | positive | 0.30881585 | 0.3 | 1 | 2 | 2.77537 |
| O_OTU44 (Zanclospora_jonesii) | F_OTU17 (Acrodictys_fluminicola) | 0.071497 | 0.47739166 | positive | 0.07149718 | 0 | 0 | 0 | 0.32113 |
| O_OTU95 (Zanclospora_jonesii) | F_OTU17 (Acrodictys_fluminicola) | 0.233301 | 0.01887666 | positive | 0.23330072 | 0 | 1 | 1 | 1.72407 |
| O_OTU154 (Diversispora_spurca) | F_OTU17 (Acrodictys_fluminicola) | -0.085783 | 0.39368755 | negative | 0.08578277 | 0 | 0 | 0 | 0.40485 |
| O_OTU599 (Aspergillus_subflavus) | F_OTU17 (Acrodictys_fluminicola) | 0.340085 | 0.00050217 | positive | 0.34008549 | 0.3 | 1 | 2 | 3.29914 |
| O_OTU1734 (Acrodictys_fluminicola) | F_OTU17 (Acrodictys_fluminicola) | 0.069566 | 0.48939673 | positive | 0.06956632 | 0 | 0 | 0 | 0.31034 |
| O_OTU5905 (Blumeria_spe_Incertae_sedis) | F_OTU17 (Acrodictys_fluminicola) | 0.222105 | 0.02559508 | positive | 0.22210468 | 0 | 1 | 1 | 1.59184 |
| O_OTU5962 (Acrodictys_fluminicola) | F_OTU17 (Acrodictys_fluminicola) | 0.213607 | 0.03196662 | positive | 0.21360743 | 0 | 1 | 1 | 1.4953 |
| O_OTU7942 (Fungi_spe_Incertae_sedis) | F_OTU17 (Acrodictys_fluminicola) | 0.033061 | 0.74275358 | positive | 0.03306061 | 0 | 0 | 0 | 0.12916 |
| O_OTU8123 (Heitmania_litseae) | F_OTU17 (Acrodictys_fluminicola) | 0.121461 | 0.226294 | positive | 0.12146111 | 0 | 0 | 0 | 0.64533 |
| Neutralizing_antibody | F_OTU17 (Acrodictys_fluminicola) | -0.293887 | 0.00285499 | negative | 0.29388738 | 0 | 1 | 2 | 2.54439 |
| lgM | F_OTU17 (Acrodictys_fluminicola) | -0.145463 | 0.14666294 | negative | 0.14546294 | 0 | 0 | 0 | 0.83368 |
| lgG | F_OTU17 (Acrodictys_fluminicola) | -0.304872 | 0.00193561 | negative | 0.30487192 | 0.3 | 1 | 2 | 2.71318 |
| MON# | F_OTU17 (Acrodictys_fluminicola) | 0.419629 | 1.2532E-05 | positive | 0.41962913 | 0.3 | 1 | 2 | 4.90164 |
| MON_p.c. | F_OTU17 (Acrodictys_fluminicola) | 0.417255 | 1.4192E-05 | positive | 0.41725472 | 0.3 | 1 | 2 | 4.84766 |
| IBIL | F_OTU17 (Acrodictys_fluminicola) | -0.325037 | 0.00091168 | negative | 0.32503671 | 0.3 | 1 | 2 | 3.04015 |
| TP | F_OTU17 (Acrodictys_fluminicola) | -0.010449 | 0.91739836 | negative | 0.01044946 | 0 | 0 | 0 | 0.03744 |
| ALB | F_OTU17 (Acrodictys_fluminicola) | -0.154482 | 0.12295909 | negative | 0.1544816 | 0 | 0 | 0 | 0.91024 |
| GLOB | F_OTU17 (Acrodictys_fluminicola) | 0.133218 | 0.18414959 | positive | 0.13321787 | 0 | 0 | 0 | 0.73483 |
| TBA | F_OTU17 (Acrodictys_fluminicola) | 0.062308 | 0.53591985 | positive | 0.06230795 | 0 | 0 | 0 | 0.2709 |
| F_OTU3924 (Saccharomyces_cerevisiae) | F_OTU17 (Acrodictys_fluminicola) | 0.389837 | 5.5852E-05 | positive | 0.38983715 | 0.3 | 1 | 2 | 4.25289 |
| F_OTU6998 (Calosphaeria_pulchella) | F_OTU17 (Acrodictys_fluminicola) | -0.449468 | 2.4171E-06 | negative | 0.44946808 | 0.3 | 1 | 2 | 5.61492 |
| F_OTU3128 (Sporothrix_dombeyi) | F_OTU69 (Apiotrichum_domesticum) | 0.042874 | 0.6703208 | positive | 0.04287368 | 0 | 0 | 0 | 0.17372 |
| F_OTU5539 (Fungi_spe_Incertae_sedis) | F_OTU69 (Apiotrichum_domesticum) | 0.062512 | 0.53458109 | positive | 0.06251224 | 0 | 0 | 0 | 0.27199 |
| F_OTU7821 (Acrodictys_fluminicola) | F_OTU69 (Apiotrichum_domesticum) | -0.129009 | 0.19852482 | negative | 0.12900891 | 0 | 0 | 0 | 0.70219 |
| F_OTU7828 (Acrodictys_fluminicola) | F_OTU69 (Apiotrichum_domesticum) | 0.040323 | 0.6888891 | positive | 0.04032332 | 0 | 0 | 0 | 0.16185 |
| O_OTU13 (Cryptococcus_longus) | F_OTU69 (Apiotrichum_domesticum) | 0.09987 | 0.32038178 | positive | 0.09986987 | 0 | 0 | 0 | 0.49433 |
| O_OTU29 (Blumeria_spe_Incertae_sedis) | F_OTU69 (Apiotrichum_domesticum) | 0.148907 | 0.1372339 | positive | 0.14890724 | 0 | 0 | 0 | 0.86254 |
| O_OTU30 (Aspergillus_subflavus) | F_OTU69 (Apiotrichum_domesticum) | 0.135461 | 0.1768074 | positive | 0.13546051 | 0 | 0 | 0 | 0.7525 |
| O_OTU33 (Aspergillus_subflavus) | F_OTU69 (Apiotrichum_domesticum) | 0.124939 | 0.21317682 | positive | 0.12493908 | 0 | 0 | 0 | 0.67126 |
| O_OTU44 (Zanclospora_jonesii) | F_OTU69 (Apiotrichum_domesticum) | -0.075995 | 0.45005251 | negative | 0.07599505 | 0 | 0 | 0 | 0.34674 |
| O_OTU95 (Zanclospora_jonesii) | F_OTU69 (Apiotrichum_domesticum) | 0.001889 | 0.98503993 | positive | 0.00188929 | 0 | 0 | 0 | 0.00655 |
| O_OTU154 (Diversispora_spurca) | F_OTU69 (Apiotrichum_domesticum) | 0.213076 | 0.03240637 | positive | 0.21307565 | 0 | 1 | 1 | 1.48937 |
| O_OTU599 (Aspergillus_subflavus) | F_OTU69 (Apiotrichum_domesticum) | -0.118157 | 0.23927093 | negative | 0.1181571 | 0 | 0 | 0 | 0.62111 |
| O_OTU1734 (Acrodictys_fluminicola) | F_OTU69 (Apiotrichum_domesticum) | 0.031961 | 0.75102501 | positive | 0.03196111 | 0 | 0 | 0 | 0.12435 |
| O_OTU5905 (Blumeria_spe_Incertae_sedis) | F_OTU69 (Apiotrichum_domesticum) | 0.118304 | 0.23868381 | positive | 0.11830385 | 0 | 0 | 0 | 0.62218 |
| O_OTU5962 (Acrodictys_fluminicola) | F_OTU69 (Apiotrichum_domesticum) | 0.064674 | 0.52051765 | positive | 0.06467391 | 0 | 0 | 0 | 0.28356 |
| O_OTU7942 (Fungi_spe_Incertae_sedis) | F_OTU69 (Apiotrichum_domesticum) | 0.141556 | 0.1579396 | positive | 0.14155615 | 0 | 0 | 0 | 0.80151 |
| O_OTU8123 (Heitmania_litseae) | F_OTU69 (Apiotrichum_domesticum) | 0.123581 | 0.21823359 | positive | 0.12358084 | 0 | 0 | 0 | 0.66108 |
| Neutralizing_antibody | F_OTU69 (Apiotrichum_domesticum) | -0.329704 | 0.00076019 | negative | 0.3297036 | 0.3 | 1 | 2 | 3.11907 |
| lgM | F_OTU69 (Apiotrichum_domesticum) | -0.158989 | 0.11227433 | negative | 0.15898931 | 0 | 0 | 0 | 0.94972 |
| lgG | F_OTU69 (Apiotrichum_domesticum) | -0.158162 | 0.11418032 | negative | 0.15816157 | 0 | 0 | 0 | 0.94241 |
| MON# | F_OTU69 (Apiotrichum_domesticum) | 0.015914 | 0.87449626 | positive | 0.01591369 | 0 | 0 | 0 | 0.05824 |
| MON_p.c. | F_OTU69 (Apiotrichum_domesticum) | 0.097923 | 0.32995516 | positive | 0.0979226 | 0 | 0 | 0 | 0.48155 |
| IBIL | F_OTU69 (Apiotrichum_domesticum) | -0.113437 | 0.25869036 | negative | 0.11343743 | 0 | 0 | 0 | 0.58722 |
| TP | F_OTU69 (Apiotrichum_domesticum) | -0.297595 | 0.00250818 | negative | 0.29759477 | 0 | 1 | 2 | 2.60064 |
| ALB | F_OTU69 (Apiotrichum_domesticum) | -0.299797 | 0.00232063 | negative | 0.29979666 | 0 | 1 | 2 | 2.63439 |
| GLOB | F_OTU69 (Apiotrichum_domesticum) | -0.233077 | 0.01899459 | negative | 0.23307651 | 0 | 1 | 1 | 1.72137 |
| TBA | F_OTU69 (Apiotrichum_domesticum) | 0.126921 | 0.20594815 | positive | 0.12692106 | 0 | 0 | 0 | 0.68624 |
| F_OTU3924 (Saccharomyces_cerevisiae) | F_OTU69 (Apiotrichum_domesticum) | -0.06997 | 0.48687333 | negative | 0.06997006 | 0 | 0 | 0 | 0.31258 |
| F_OTU6998 (Calosphaeria_pulchella) | F_OTU69 (Apiotrichum_domesticum) | -0.040795 | 0.68544243 | negative | 0.04079475 | 0 | 0 | 0 | 0.16403 |
| F_OTU5539 (Fungi_spe_Incertae_sedis) | F_OTU3128 (Sporothrix_dombeyi) | 0.700474 | 4.4409E-16 | positive | 0.70047396 | 0.5 | 1 | 2 | 8 |
| F_OTU7821 (Acrodictys_fluminicola) | F_OTU3128 (Sporothrix_dombeyi) | 0.510063 | 5.0828E-08 | positive | 0.51006343 | 0.5 | 1 | 2 | 7.2159 |
| F_OTU7828 (Acrodictys_fluminicola) | F_OTU3128 (Sporothrix_dombeyi) | 0.393211 | 4.7491E-05 | positive | 0.39321068 | 0.3 | 1 | 2 | 4.3233 |
| O_OTU13 (Cryptococcus_longus) | F_OTU3128 (Sporothrix_dombeyi) | 0.237801 | 0.01663929 | positive | 0.2378012 | 0 | 1 | 1 | 1.77886 |
| O_OTU29 (Blumeria_spe_Incertae_sedis) | F_OTU3128 (Sporothrix_dombeyi) | 0.09877 | 0.3257654 | positive | 0.0987703 | 0 | 0 | 0 | 0.4871 |
| O_OTU30 (Aspergillus_subflavus) | F_OTU3128 (Sporothrix_dombeyi) | 0.141369 | 0.15849439 | positive | 0.1413694 | 0 | 0 | 0 | 0.79999 |
| O_OTU33 (Aspergillus_subflavus) | F_OTU3128 (Sporothrix_dombeyi) | 0.084157 | 0.40274411 | positive | 0.08415749 | 0 | 0 | 0 | 0.39497 |
| O_OTU44 (Zanclospora_jonesii) | F_OTU3128 (Sporothrix_dombeyi) | 0.305224 | 0.00191116 | positive | 0.30522426 | 0.3 | 1 | 2 | 2.7187 |
| O_OTU95 (Zanclospora_jonesii) | F_OTU3128 (Sporothrix_dombeyi) | 0.18115 | 0.06984639 | positive | 0.18115012 | 0 | 0 | 0 | 1.15586 |
| O_OTU154 (Diversispora_spurca) | F_OTU3128 (Sporothrix_dombeyi) | 0.34447 | 0.00041968 | positive | 0.34446985 | 0.3 | 1 | 2 | 3.37707 |
| O_OTU599 (Aspergillus_subflavus) | F_OTU3128 (Sporothrix_dombeyi) | 0.12602 | 0.20921362 | positive | 0.12601968 | 0 | 0 | 0 | 0.67941 |
| O_OTU1734 (Acrodictys_fluminicola) | F_OTU3128 (Sporothrix_dombeyi) | 0.368584 | 0.00014911 | positive | 0.36858353 | 0.3 | 1 | 2 | 3.82646 |
| O_OTU5905 (Blumeria_spe_Incertae_sedis) | F_OTU3128 (Sporothrix_dombeyi) | 0.064577 | 0.52114269 | positive | 0.06457722 | 0 | 0 | 0 | 0.28304 |
| O_OTU5962 (Acrodictys_fluminicola) | F_OTU3128 (Sporothrix_dombeyi) | 0.207081 | 0.03772662 | positive | 0.20708095 | 0 | 1 | 1 | 1.42335 |
| O_OTU7942 (Fungi_spe_Incertae_sedis) | F_OTU3128 (Sporothrix_dombeyi) | 0.177214 | 0.07624967 | positive | 0.17721427 | 0 | 0 | 0 | 1.11776 |
| O_OTU8123 (Heitmania_litseae) | F_OTU3128 (Sporothrix_dombeyi) | 0.200554 | 0.04433178 | positive | 0.20055449 | 0 | 1 | 1 | 1.35328 |
| Neutralizing_antibody | F_OTU3128 (Sporothrix_dombeyi) | -0.051429 | 0.60951789 | negative | 0.0514292 | 0 | 0 | 0 | 0.21501 |
| lgM | F_OTU3128 (Sporothrix_dombeyi) | -0.187082 | 0.06102404 | negative | 0.18708175 | 0 | 0 | 0 | 1.2145 |
| lgG | F_OTU3128 (Sporothrix_dombeyi) | -0.12117 | 0.22741739 | negative | 0.12116993 | 0 | 0 | 0 | 0.64318 |
| MON# | F_OTU3128 (Sporothrix_dombeyi) | 0.02628 | 0.79419453 | positive | 0.02627994 | 0 | 0 | 0 | 0.10007 |
| MON_p.c. | F_OTU3128 (Sporothrix_dombeyi) | 0.008241 | 0.93480912 | positive | 0.00824143 | 0 | 0 | 0 | 0.02928 |
| IBIL | F_OTU3128 (Sporothrix_dombeyi) | -0.266865 | 0.00698237 | negative | 0.2668648 | 0 | 1 | 2 | 2.156 |
| TP | F_OTU3128 (Sporothrix_dombeyi) | -0.123124 | 0.21995394 | negative | 0.12312384 | 0 | 0 | 0 | 0.65767 |
| ALB | F_OTU3128 (Sporothrix_dombeyi) | -0.195737 | 0.04980087 | negative | 0.19573738 | 0 | 1 | 1 | 1.30276 |
| GLOB | F_OTU3128 (Sporothrix_dombeyi) | -0.042289 | 0.67455838 | negative | 0.04228932 | 0 | 0 | 0 | 0.17098 |
| TBA | F_OTU3128 (Sporothrix_dombeyi) | 0.144399 | 0.1496707 | positive | 0.14439946 | 0 | 0 | 0 | 0.82486 |
| F_OTU3924 (Saccharomyces_cerevisiae) | F_OTU3128 (Sporothrix_dombeyi) | -0.182674 | 0.06748708 | negative | 0.18267394 | 0 | 0 | 0 | 1.17078 |
| F_OTU6998 (Calosphaeria_pulchella) | F_OTU3128 (Sporothrix_dombeyi) | 0.45554 | 1.6959E-06 | positive | 0.45553976 | 0.3 | 1 | 2 | 5.76805 |
| F_OTU7821 (Acrodictys_fluminicola) | F_OTU5539 (Fungi_spe_Incertae_sedis) | 0.530849 | 1.1268E-08 | positive | 0.53084857 | 0.5 | 1 | 2 | 7.67228 |
| F_OTU7828 (Acrodictys_fluminicola) | F_OTU5539 (Fungi_spe_Incertae_sedis) | 0.5554 | 1.6593E-09 | positive | 0.55540038 | 0.5 | 1 | 2 | 7.93333 |
| O_OTU13 (Cryptococcus_longus) | F_OTU5539 (Fungi_spe_Incertae_sedis) | 0.369616 | 0.00014239 | positive | 0.36961563 | 0.3 | 1 | 2 | 3.8465 |
| O_OTU29 (Blumeria_spe_Incertae_sedis) | F_OTU5539 (Fungi_spe_Incertae_sedis) | 0.291276 | 0.00312455 | positive | 0.29127594 | 0 | 1 | 2 | 2.50521 |
| O_OTU30 (Aspergillus_subflavus) | F_OTU5539 (Fungi_spe_Incertae_sedis) | 0.258719 | 0.00899172 | positive | 0.25871915 | 0 | 1 | 2 | 2.04616 |
| O_OTU33 (Aspergillus_subflavus) | F_OTU5539 (Fungi_spe_Incertae_sedis) | 0.055346 | 0.58251312 | positive | 0.05534589 | 0 | 0 | 0 | 0.23469 |
| O_OTU44 (Zanclospora_jonesii) | F_OTU5539 (Fungi_spe_Incertae_sedis) | 0.352837 | 0.00029581 | positive | 0.35283663 | 0.3 | 1 | 2 | 3.52898 |
| O_OTU95 (Zanclospora_jonesii) | F_OTU5539 (Fungi_spe_Incertae_sedis) | 0.26276 | 0.00793894 | positive | 0.26276013 | 0 | 1 | 2 | 2.10024 |
| O_OTU154 (Diversispora_spurca) | F_OTU5539 (Fungi_spe_Incertae_sedis) | 0.386962 | 6.4039E-05 | positive | 0.38696243 | 0.3 | 1 | 2 | 4.19348 |
| O_OTU599 (Aspergillus_subflavus) | F_OTU5539 (Fungi_spe_Incertae_sedis) | -0.009968 | 0.92119314 | negative | 0.00996782 | 0 | 0 | 0 | 0.03565 |
| O_OTU1734 (Acrodictys_fluminicola) | F_OTU5539 (Fungi_spe_Incertae_sedis) | 0.356871 | 0.00024903 | positive | 0.35687092 | 0.3 | 1 | 2 | 3.60374 |
| O_OTU5905 (Blumeria_spe_Incertae_sedis) | F_OTU5539 (Fungi_spe_Incertae_sedis) | 0.234264 | 0.01837732 | positive | 0.23426362 | 0 | 1 | 1 | 1.73572 |
| O_OTU5962 (Acrodictys_fluminicola) | F_OTU5539 (Fungi_spe_Incertae_sedis) | 0.281576 | 0.00433716 | positive | 0.28157624 | 0 | 1 | 2 | 2.36279 |
| O_OTU7942 (Fungi_spe_Incertae_sedis) | F_OTU5539 (Fungi_spe_Incertae_sedis) | 0.292194 | 0.00302725 | positive | 0.29219433 | 0 | 1 | 2 | 2.51895 |
| O_OTU8123 (Heitmania_litseae) | F_OTU5539 (Fungi_spe_Incertae_sedis) | 0.321548 | 0.0010424 | positive | 0.32154794 | 0.3 | 1 | 2 | 2.98196 |
| Neutralizing_antibody | F_OTU5539 (Fungi_spe_Incertae_sedis) | -0.272572 | 0.00582213 | negative | 0.27257165 | 0 | 1 | 2 | 2.23492 |
| lgM | F_OTU5539 (Fungi_spe_Incertae_sedis) | -0.270291 | 0.00626349 | negative | 0.2702911 | 0 | 1 | 2 | 2.20318 |
| lgG | F_OTU5539 (Fungi_spe_Incertae_sedis) | -0.243966 | 0.01394868 | negative | 0.24396624 | 0 | 1 | 1 | 1.85547 |
| MON# | F_OTU5539 (Fungi_spe_Incertae_sedis) | -0.010148 | 0.91977371 | negative | 0.01014795 | 0 | 0 | 0 | 0.03632 |
| MON_p.c. | F_OTU5539 (Fungi_spe_Incertae_sedis) | -0.013248 | 0.89538684 | negative | 0.01324818 | 0 | 0 | 0 | 0.04799 |
| IBIL | F_OTU5539 (Fungi_spe_Incertae_sedis) | -0.305404 | 0.00189882 | negative | 0.30540354 | 0.3 | 1 | 2 | 2.72151 |
| TP | F_OTU5539 (Fungi_spe_Incertae_sedis) | -0.3242 | 0.00094158 | negative | 0.32420025 | 0.3 | 1 | 2 | 3.02614 |
| ALB | F_OTU5539 (Fungi_spe_Incertae_sedis) | -0.39044 | 5.4263E-05 | negative | 0.39044024 | 0.3 | 1 | 2 | 4.26542 |
| GLOB | F_OTU5539 (Fungi_spe_Incertae_sedis) | -0.184968 | 0.06405696 | negative | 0.1849683 | 0 | 0 | 0 | 1.19343 |
| TBA | F_OTU5539 (Fungi_spe_Incertae_sedis) | 0.238451 | 0.01633605 | positive | 0.23845087 | 0 | 1 | 1 | 1.78685 |
| F_OTU3924 (Saccharomyces_cerevisiae) | F_OTU5539 (Fungi_spe_Incertae_sedis) | -0.166764 | 0.09555607 | negative | 0.16676406 | 0 | 0 | 0 | 1.01974 |
| F_OTU6998 (Calosphaeria_pulchella) | F_OTU5539 (Fungi_spe_Incertae_sedis) | 0.436366 | 5.0753E-06 | positive | 0.4363658 | 0.3 | 1 | 2 | 5.29368 |
| F_OTU7828 (Acrodictys_fluminicola) | F_OTU7821 (Acrodictys_fluminicola) | 0.66931 | 1.9984E-14 | positive | 0.66930985 | 0.5 | 1 | 2 | 8 |
| O_OTU13 (Cryptococcus_longus) | F_OTU7821 (Acrodictys_fluminicola) | -0.06995 | 0.48699973 | negative | 0.06994981 | 0 | 0 | 0 | 0.31247 |
| O_OTU29 (Blumeria_spe_Incertae_sedis) | F_OTU7821 (Acrodictys_fluminicola) | -0.16102 | 0.10770353 | negative | 0.16101977 | 0 | 0 | 0 | 0.96777 |
| O_OTU30 (Aspergillus_subflavus) | F_OTU7821 (Acrodictys_fluminicola) | -0.098236 | 0.32840372 | negative | 0.09823569 | 0 | 0 | 0 | 0.48359 |
| O_OTU33 (Aspergillus_subflavus) | F_OTU7821 (Acrodictys_fluminicola) | -0.23694 | 0.01704881 | negative | 0.23693989 | 0 | 1 | 1 | 1.76831 |
| O_OTU44 (Zanclospora_jonesii) | F_OTU7821 (Acrodictys_fluminicola) | 0.283071 | 0.00412646 | positive | 0.28307089 | 0 | 1 | 2 | 2.38442 |
| O_OTU95 (Zanclospora_jonesii) | F_OTU7821 (Acrodictys_fluminicola) | 0.122019 | 0.22415143 | positive | 0.1220193 | 0 | 0 | 0 | 0.64946 |
| O_OTU154 (Diversispora_spurca) | F_OTU7821 (Acrodictys_fluminicola) | 0.194065 | 0.05182482 | positive | 0.19406537 | 0 | 0 | 0 | 1.28546 |
| O_OTU599 (Aspergillus_subflavus) | F_OTU7821 (Acrodictys_fluminicola) | -0.247885 | 0.01244225 | negative | 0.24788535 | 0 | 1 | 1 | 1.9051 |
| O_OTU1734 (Acrodictys_fluminicola) | F_OTU7821 (Acrodictys_fluminicola) | 0.149005 | 0.13697207 | positive | 0.14900541 | 0 | 0 | 0 | 0.86337 |
| O_OTU5905 (Blumeria_spe_Incertae_sedis) | F_OTU7821 (Acrodictys_fluminicola) | -0.166282 | 0.09653239 | negative | 0.16628182 | 0 | 0 | 0 | 1.01533 |
| O_OTU5962 (Acrodictys_fluminicola) | F_OTU7821 (Acrodictys_fluminicola) | 0.03459 | 0.73129761 | positive | 0.03458989 | 0 | 0 | 0 | 0.13591 |
| O_OTU7942 (Fungi_spe_Incertae_sedis) | F_OTU7821 (Acrodictys_fluminicola) | 0.112397 | 0.26311361 | positive | 0.11239662 | 0 | 0 | 0 | 0.57986 |
| O_OTU8123 (Heitmania_litseae) | F_OTU7821 (Acrodictys_fluminicola) | -0.064431 | 0.52208843 | negative | 0.06443102 | 0 | 0 | 0 | 0.28226 |
| Neutralizing_antibody | F_OTU7821 (Acrodictys_fluminicola) | 0.215779 | 0.0302224 | positive | 0.2157795 | 0 | 1 | 1 | 1.51967 |
| lgM | F_OTU7821 (Acrodictys_fluminicola) | -0.077505 | 0.44107648 | negative | 0.07750466 | 0 | 0 | 0 | 0.35549 |
| lgG | F_OTU7821 (Acrodictys_fluminicola) | 0.18084 | 0.07033501 | positive | 0.18083975 | 0 | 0 | 0 | 1.15283 |
| MON# | F_OTU7821 (Acrodictys_fluminicola) | -0.363218 | 0.00018906 | negative | 0.36321805 | 0.3 | 1 | 2 | 3.72337 |
| MON_p.c. | F_OTU7821 (Acrodictys_fluminicola) | -0.386526 | 6.5375E-05 | negative | 0.38652636 | 0.3 | 1 | 2 | 4.18452 |
| IBIL | F_OTU7821 (Acrodictys_fluminicola) | 0.02171 | 0.82938298 | positive | 0.02171006 | 0 | 0 | 0 | 0.08124 |
| TP | F_OTU7821 (Acrodictys_fluminicola) | -0.06983 | 0.48775003 | negative | 0.06982966 | 0 | 0 | 0 | 0.3118 |
| ALB | F_OTU7821 (Acrodictys_fluminicola) | -0.020635 | 0.8377116 | negative | 0.02063518 | 0 | 0 | 0 | 0.07691 |
| GLOB | F_OTU7821 (Acrodictys_fluminicola) | -0.105539 | 0.29353743 | negative | 0.10553947 | 0 | 0 | 0 | 0.53234 |
| TBA | F_OTU7821 (Acrodictys_fluminicola) | 0.06898 | 0.49307361 | positive | 0.06897999 | 0 | 0 | 0 | 0.30709 |
| F_OTU3924 (Saccharomyces_cerevisiae) | F_OTU7821 (Acrodictys_fluminicola) | -0.353077 | 0.00029281 | negative | 0.35307691 | 0.3 | 1 | 2 | 3.5334 |
| F_OTU6998 (Calosphaeria_pulchella) | F_OTU7821 (Acrodictys_fluminicola) | 0.638016 | 7.1787E-13 | positive | 0.63801553 | 0.5 | 1 | 2 | 7.99997 |
| O_OTU13 (Cryptococcus_longus) | F_OTU7828 (Acrodictys_fluminicola) | -0.045068 | 0.65450225 | negative | 0.04506788 | 0 | 0 | 0 | 0.18409 |
| O_OTU29 (Blumeria_spe_Incertae_sedis) | F_OTU7828 (Acrodictys_fluminicola) | -0.099801 | 0.32071875 | negative | 0.0998007 | 0 | 0 | 0 | 0.49388 |
| O_OTU30 (Aspergillus_subflavus) | F_OTU7828 (Acrodictys_fluminicola) | -0.087198 | 0.38590017 | negative | 0.08719821 | 0 | 0 | 0 | 0.41353 |
| O_OTU33 (Aspergillus_subflavus) | F_OTU7828 (Acrodictys_fluminicola) | -0.286791 | 0.00364125 | negative | 0.28679076 | 0 | 1 | 2 | 2.43875 |
| O_OTU44 (Zanclospora_jonesii) | F_OTU7828 (Acrodictys_fluminicola) | 0.073739 | 0.46365254 | positive | 0.07373937 | 0 | 0 | 0 | 0.33381 |
| O_OTU95 (Zanclospora_jonesii) | F_OTU7828 (Acrodictys_fluminicola) | 0.039978 | 0.69141853 | positive | 0.03997791 | 0 | 0 | 0 | 0.16026 |
| O_OTU154 (Diversispora_spurca) | F_OTU7828 (Acrodictys_fluminicola) | 0.256003 | 0.00976643 | positive | 0.25600348 | 0 | 1 | 2 | 2.01026 |
| O_OTU599 (Aspergillus_subflavus) | F_OTU7828 (Acrodictys_fluminicola) | -0.315366 | 0.00131662 | negative | 0.31536637 | 0.3 | 1 | 2 | 2.88054 |
| O_OTU1734 (Acrodictys_fluminicola) | F_OTU7828 (Acrodictys_fluminicola) | 0.136524 | 0.17340296 | positive | 0.1365236 | 0 | 0 | 0 | 0.76094 |
| O_OTU5905 (Blumeria_spe_Incertae_sedis) | F_OTU7828 (Acrodictys_fluminicola) | -0.156664 | 0.11769311 | negative | 0.15666368 | 0 | 0 | 0 | 0.92925 |
| O_OTU5962 (Acrodictys_fluminicola) | F_OTU7828 (Acrodictys_fluminicola) | 0.14397 | 0.15089884 | positive | 0.14396984 | 0 | 0 | 0 | 0.82131 |
| O_OTU7942 (Fungi_spe_Incertae_sedis) | F_OTU7828 (Acrodictys_fluminicola) | 0.117866 | 0.240437 | positive | 0.11786639 | 0 | 0 | 0 | 0.619 |
| O_OTU8123 (Heitmania_litseae) | F_OTU7828 (Acrodictys_fluminicola) | -0.124276 | 0.21563578 | negative | 0.12427578 | 0 | 0 | 0 | 0.66628 |
| Neutralizing_antibody | F_OTU7828 (Acrodictys_fluminicola) | 0.128294 | 0.20104547 | positive | 0.12829378 | 0 | 0 | 0 | 0.69671 |
| lgM | F_OTU7828 (Acrodictys_fluminicola) | 0.107833 | 0.28311112 | positive | 0.10783343 | 0 | 0 | 0 | 0.54804 |
| lgG | F_OTU7828 (Acrodictys_fluminicola) | 0.21883 | 0.02790902 | positive | 0.21883027 | 0 | 1 | 1 | 1.55426 |
| MON# | F_OTU7828 (Acrodictys_fluminicola) | -0.278854 | 0.00474563 | negative | 0.27885449 | 0 | 1 | 2 | 2.32371 |
| MON_p.c. | F_OTU7828 (Acrodictys_fluminicola) | -0.20879 | 0.03614037 | negative | 0.20878971 | 0 | 1 | 1 | 1.44201 |
| IBIL | F_OTU7828 (Acrodictys_fluminicola) | 0.088678 | 0.37785941 | positive | 0.08867784 | 0 | 0 | 0 | 0.42267 |
| TP | F_OTU7828 (Acrodictys_fluminicola) | -0.086428 | 0.39012796 | negative | 0.08642766 | 0 | 0 | 0 | 0.40879 |
| ALB | F_OTU7828 (Acrodictys_fluminicola) | -0.029109 | 0.77260946 | negative | 0.02910916 | 0 | 0 | 0 | 0.11204 |
| GLOB | F_OTU7828 (Acrodictys_fluminicola) | -0.118768 | 0.23683441 | negative | 0.11876775 | 0 | 0 | 0 | 0.62556 |
| TBA | F_OTU7828 (Acrodictys_fluminicola) | 0.162839 | 0.10373165 | positive | 0.16283947 | 0 | 0 | 0 | 0.98409 |
| F_OTU3924 (Saccharomyces_cerevisiae) | F_OTU7828 (Acrodictys_fluminicola) | -0.271364 | 0.00605232 | negative | 0.2713638 | 0 | 1 | 2 | 2.21808 |
| F_OTU6998 (Calosphaeria_pulchella) | F_OTU7828 (Acrodictys_fluminicola) | 0.666278 | 2.8866E-14 | positive | 0.66627789 | 0.5 | 1 | 2 | 8 |
| O_OTU29 (Blumeria_spe_Incertae_sedis) | O_OTU13 (Cryptococcus_longus) | 0.447475 | 2.7112E-06 | positive | 0.44747505 | 0.3 | 1 | 2 | 5.56524 |
| O_OTU30 (Aspergillus_subflavus) | O_OTU13 (Cryptococcus_longus) | 0.379215 | 9.2011E-05 | positive | 0.37921528 | 0.3 | 1 | 2 | 4.03611 |
| O_OTU33 (Aspergillus_subflavus) | O_OTU13 (Cryptococcus_longus) | 0.288922 | 0.00338683 | positive | 0.28892242 | 0 | 1 | 2 | 2.4702 |
| O_OTU44 (Zanclospora_jonesii) | O_OTU13 (Cryptococcus_longus) | 0.137862 | 0.16918556 | positive | 0.13786208 | 0 | 0 | 0 | 0.77164 |
| O_OTU95 (Zanclospora_jonesii) | O_OTU13 (Cryptococcus_longus) | 0.371559 | 0.00013048 | positive | 0.37155922 | 0.3 | 1 | 2 | 3.88441 |
| O_OTU154 (Diversispora_spurca) | O_OTU13 (Cryptococcus_longus) | 0.199638 | 0.04533183 | positive | 0.19963817 | 0 | 1 | 1 | 1.3436 |
| O_OTU599 (Aspergillus_subflavus) | O_OTU13 (Cryptococcus_longus) | 0.144791 | 0.14855905 | positive | 0.14479063 | 0 | 0 | 0 | 0.8281 |
| O_OTU1734 (Acrodictys_fluminicola) | O_OTU13 (Cryptococcus_longus) | 0.146722 | 0.14316143 | positive | 0.1467219 | 0 | 0 | 0 | 0.84417 |
| O_OTU5905 (Blumeria_spe_Incertae_sedis) | O_OTU13 (Cryptococcus_longus) | 0.454914 | 1.7595E-06 | positive | 0.4549143 | 0.3 | 1 | 2 | 5.75216 |
| O_OTU5962 (Acrodictys_fluminicola) | O_OTU13 (Cryptococcus_longus) | 0.148698 | 0.1377936 | positive | 0.14869787 | 0 | 0 | 0 | 0.86077 |
| O_OTU7942 (Fungi_spe_Incertae_sedis) | O_OTU13 (Cryptococcus_longus) | 0.127574 | 0.20360499 | positive | 0.12757417 | 0 | 0 | 0 | 0.69121 |
| O_OTU8123 (Heitmania_litseae) | O_OTU13 (Cryptococcus_longus) | 0.309479 | 0.00163719 | positive | 0.3094785 | 0.3 | 1 | 2 | 2.7859 |
| Neutralizing_antibody | O_OTU13 (Cryptococcus_longus) | -0.509887 | 5.1462E-08 | negative | 0.50988663 | 0.5 | 1 | 2 | 7.2114 |
| lgM | O_OTU13 (Cryptococcus_longus) | -0.429318 | 7.47E-06 | negative | 0.42931772 | 0.3 | 1 | 2 | 5.1261 |
| lgG | O_OTU13 (Cryptococcus_longus) | -0.388542 | 5.941E-05 | negative | 0.38854244 | 0.3 | 1 | 2 | 4.22606 |
| MON# | O_OTU13 (Cryptococcus_longus) | 0.382236 | 7.9971E-05 | positive | 0.38223643 | 0.3 | 1 | 2 | 4.09702 |
| MON_p.c. | O_OTU13 (Cryptococcus_longus) | 0.388904 | 5.8396E-05 | positive | 0.38890393 | 0.3 | 1 | 2 | 4.23354 |
| IBIL | O_OTU13 (Cryptococcus_longus) | -0.333649 | 0.00065046 | negative | 0.33364936 | 0.3 | 1 | 2 | 3.18677 |
| TP | O_OTU13 (Cryptococcus_longus) | -0.245725 | 0.0132541 | negative | 0.24572496 | 0 | 1 | 1 | 1.87765 |
| ALB | O_OTU13 (Cryptococcus_longus) | -0.37036 | 0.00013771 | negative | 0.37036011 | 0.3 | 1 | 2 | 3.86099 |
| GLOB | O_OTU13 (Cryptococcus_longus) | -0.078996 | 0.4323091 | negative | 0.07899601 | 0 | 0 | 0 | 0.36421 |
| TBA | O_OTU13 (Cryptococcus_longus) | 0.25391 | 0.01040304 | positive | 0.25391002 | 0 | 1 | 1 | 1.98284 |
| F_OTU3924 (Saccharomyces_cerevisiae) | O_OTU13 (Cryptococcus_longus) | 0.254381 | 0.0102568 | positive | 0.25438077 | 0 | 1 | 1 | 1.98899 |
| F_OTU6998 (Calosphaeria_pulchella) | O_OTU13 (Cryptococcus_longus) | -0.299876 | 0.00231411 | negative | 0.29987609 | 0 | 1 | 2 | 2.63561 |
| O_OTU30 (Aspergillus_subflavus) | O_OTU29 (Blumeria_spe_Incertae_sedis) | 0.833739 | 0 | positive | 0.83373869 | 0.8 | 1 | 2 | 8 |
| O_OTU33 (Aspergillus_subflavus) | O_OTU29 (Blumeria_spe_Incertae_sedis) | 0.292011 | 0.00304643 | positive | 0.29201126 | 0 | 1 | 2 | 2.51621 |
| O_OTU44 (Zanclospora_jonesii) | O_OTU29 (Blumeria_spe_Incertae_sedis) | 0.24811 | 0.01236023 | positive | 0.24811039 | 0 | 1 | 1 | 1.90797 |
| O_OTU95 (Zanclospora_jonesii) | O_OTU29 (Blumeria_spe_Incertae_sedis) | 0.337475 | 0.0005581 | positive | 0.33747539 | 0.3 | 1 | 2 | 3.25328 |
| O_OTU154 (Diversispora_spurca) | O_OTU29 (Blumeria_spe_Incertae_sedis) | 0.272641 | 0.00580909 | positive | 0.27264135 | 0 | 1 | 2 | 2.23589 |
| O_OTU599 (Aspergillus_subflavus) | O_OTU29 (Blumeria_spe_Incertae_sedis) | 0.218166 | 0.02839982 | positive | 0.21816558 | 0 | 1 | 1 | 1.54668 |
| O_OTU1734 (Acrodictys_fluminicola) | O_OTU29 (Blumeria_spe_Incertae_sedis) | 0.214818 | 0.03098431 | positive | 0.214818 | 0 | 1 | 1 | 1.50886 |
| O_OTU5905 (Blumeria_spe_Incertae_sedis) | O_OTU29 (Blumeria_spe_Incertae_sedis) | 0.896466 | 0 | positive | 0.8964659 | 0.8 | 1 | 2 | 8 |
| O_OTU5962 (Acrodictys_fluminicola) | O_OTU29 (Blumeria_spe_Incertae_sedis) | 0.204066 | 0.04066713 | positive | 0.20406647 | 0 | 1 | 1 | 1.39076 |
| O_OTU7942 (Fungi_spe_Incertae_sedis) | O_OTU29 (Blumeria_spe_Incertae_sedis) | 0.171936 | 0.08556754 | positive | 0.17193618 | 0 | 0 | 0 | 1.06769 |
| O_OTU8123 (Heitmania_litseae) | O_OTU29 (Blumeria_spe_Incertae_sedis) | 0.432091 | 6.4228E-06 | positive | 0.43209091 | 0.3 | 1 | 2 | 5.1916 |
| Neutralizing_antibody | O_OTU29 (Blumeria_spe_Incertae_sedis) | -0.505541 | 6.9625E-08 | negative | 0.50554066 | 0.5 | 1 | 2 | 7.09895 |
| lgM | O_OTU29 (Blumeria_spe_Incertae_sedis) | -0.202022 | 0.04276791 | negative | 0.20202247 | 0 | 1 | 1 | 1.36888 |
| lgG | O_OTU29 (Blumeria_spe_Incertae_sedis) | -0.434535 | 5.6159E-06 | negative | 0.43453531 | 0.3 | 1 | 2 | 5.24981 |
| MON# | O_OTU29 (Blumeria_spe_Incertae_sedis) | 0.204684 | 0.04004964 | positive | 0.20468401 | 0 | 1 | 1 | 1.3974 |
| MON_p.c. | O_OTU29 (Blumeria_spe_Incertae_sedis) | 0.256859 | 0.00951619 | positive | 0.25685933 | 0 | 1 | 2 | 2.02154 |
| IBIL | O_OTU29 (Blumeria_spe_Incertae_sedis) | -0.274543 | 0.00546302 | negative | 0.27454343 | 0 | 1 | 2 | 2.26257 |
| TP | O_OTU29 (Blumeria_spe_Incertae_sedis) | -0.307906 | 0.001734 | negative | 0.30790622 | 0.3 | 1 | 2 | 2.76095 |
| ALB | O_OTU29 (Blumeria_spe_Incertae_sedis) | -0.442027 | 3.6975E-06 | negative | 0.4420267 | 0.3 | 1 | 2 | 5.43092 |
| GLOB | O_OTU29 (Blumeria_spe_Incertae_sedis) | -0.116916 | 0.24427581 | negative | 0.11691618 | 0 | 0 | 0 | 0.61212 |
| TBA | O_OTU29 (Blumeria_spe_Incertae_sedis) | 0.267398 | 0.00686595 | positive | 0.26739784 | 0 | 1 | 2 | 2.1633 |
| F_OTU3924 (Saccharomyces_cerevisiae) | O_OTU29 (Blumeria_spe_Incertae_sedis) | 0.317245 | 0.00122704 | positive | 0.31724511 | 0.3 | 1 | 2 | 2.91114 |
| F_OTU6998 (Calosphaeria_pulchella) | O_OTU29 (Blumeria_spe_Incertae_sedis) | -0.236345 | 0.01733685 | negative | 0.23634472 | 0 | 1 | 1 | 1.76103 |
| O_OTU33 (Aspergillus_subflavus) | O_OTU30 (Aspergillus_subflavus) | 0.253259 | 0.01060838 | positive | 0.25325869 | 0 | 1 | 1 | 1.97435 |
| O_OTU44 (Zanclospora_jonesii) | O_OTU30 (Aspergillus_subflavus) | 0.189191 | 0.05811438 | positive | 0.18919144 | 0 | 0 | 0 | 1.23572 |
| O_OTU95 (Zanclospora_jonesii) | O_OTU30 (Aspergillus_subflavus) | 0.287546 | 0.00354916 | positive | 0.28754646 | 0 | 1 | 2 | 2.44987 |
| O_OTU154 (Diversispora_spurca) | O_OTU30 (Aspergillus_subflavus) | 0.208274 | 0.03661273 | positive | 0.2082744 | 0 | 1 | 1 | 1.43637 |
| O_OTU599 (Aspergillus_subflavus) | O_OTU30 (Aspergillus_subflavus) | 0.213648 | 0.03193332 | positive | 0.21364796 | 0 | 1 | 1 | 1.49576 |
| O_OTU1734 (Acrodictys_fluminicola) | O_OTU30 (Aspergillus_subflavus) | 0.143211 | 0.15308767 | positive | 0.1432106 | 0 | 0 | 0 | 0.81506 |
| O_OTU5905 (Blumeria_spe_Incertae_sedis) | O_OTU30 (Aspergillus_subflavus) | 0.872491 | 0 | positive | 0.87249144 | 0.8 | 1 | 2 | 8 |
| O_OTU5962 (Acrodictys_fluminicola) | O_OTU30 (Aspergillus_subflavus) | 0.161678 | 0.10625329 | positive | 0.16167802 | 0 | 0 | 0 | 0.97366 |
| O_OTU7942 (Fungi_spe_Incertae_sedis) | O_OTU30 (Aspergillus_subflavus) | 0.148522 | 0.1382646 | positive | 0.14852217 | 0 | 0 | 0 | 0.85929 |
| O_OTU8123 (Heitmania_litseae) | O_OTU30 (Aspergillus_subflavus) | 0.345069 | 0.00040943 | positive | 0.3450687 | 0.3 | 1 | 2 | 3.38781 |
| Neutralizing_antibody | O_OTU30 (Aspergillus_subflavus) | -0.405001 | 2.6571E-05 | negative | 0.40500134 | 0.3 | 1 | 2 | 4.57543 |
| lgM | O_OTU30 (Aspergillus_subflavus) | -0.186514 | 0.06182695 | negative | 0.18651407 | 0 | 0 | 0 | 1.20882 |
| lgG | O_OTU30 (Aspergillus_subflavus) | -0.413027 | 1.7669E-05 | negative | 0.41302706 | 0.3 | 1 | 2 | 4.75254 |
| MON# | O_OTU30 (Aspergillus_subflavus) | 0.153039 | 0.12653944 | positive | 0.15303875 | 0 | 0 | 0 | 0.89777 |
| MON_p.c. | O_OTU30 (Aspergillus_subflavus) | 0.243052 | 0.01432191 | positive | 0.24305242 | 0 | 1 | 1 | 1.844 |
| IBIL | O_OTU30 (Aspergillus_subflavus) | -0.184585 | 0.06461966 | negative | 0.18458518 | 0 | 0 | 0 | 1.18964 |
| TP | O_OTU30 (Aspergillus_subflavus) | -0.240054 | 0.01560824 | negative | 0.2400538 | 0 | 1 | 1 | 1.80665 |
| ALB | O_OTU30 (Aspergillus_subflavus) | -0.340293 | 0.00049796 | negative | 0.34029276 | 0.3 | 1 | 2 | 3.3028 |
| GLOB | O_OTU30 (Aspergillus_subflavus) | -0.086799 | 0.3880862 | negative | 0.08679916 | 0 | 0 | 0 | 0.41107 |
| TBA | O_OTU30 (Aspergillus_subflavus) | 0.270305 | 0.00626074 | positive | 0.27030485 | 0 | 1 | 2 | 2.20337 |
| F_OTU3924 (Saccharomyces_cerevisiae) | O_OTU30 (Aspergillus_subflavus) | 0.309091 | 0.00166057 | positive | 0.30909126 | 0.3 | 1 | 2 | 2.77974 |
| F_OTU6998 (Calosphaeria_pulchella) | O_OTU30 (Aspergillus_subflavus) | -0.221855 | 0.0257655 | negative | 0.22185511 | 0 | 1 | 1 | 1.58896 |
| O_OTU44 (Zanclospora_jonesii) | O_OTU33 (Aspergillus_subflavus) | 0.044462 | 0.65885269 | positive | 0.04446237 | 0 | 0 | 0 | 0.18121 |
| O_OTU95 (Zanclospora_jonesii) | O_OTU33 (Aspergillus_subflavus) | 0.161153 | 0.10740981 | positive | 0.16115252 | 0 | 0 | 0 | 0.96896 |
| O_OTU154 (Diversispora_spurca) | O_OTU33 (Aspergillus_subflavus) | -0.072984 | 0.46825502 | negative | 0.07298428 | 0 | 0 | 0 | 0.32952 |
| O_OTU599 (Aspergillus_subflavus) | O_OTU33 (Aspergillus_subflavus) | 0.591304 | 7.4932E-11 | positive | 0.59130355 | 0.5 | 1 | 2 | 7.99676 |
| O_OTU1734 (Acrodictys_fluminicola) | O_OTU33 (Aspergillus_subflavus) | 0.22196 | 0.02569361 | positive | 0.22196022 | 0 | 1 | 1 | 1.59017 |
| O_OTU5905 (Blumeria_spe_Incertae_sedis) | O_OTU33 (Aspergillus_subflavus) | 0.280671 | 0.00446934 | positive | 0.28067133 | 0 | 1 | 2 | 2.34976 |
| O_OTU5962 (Acrodictys_fluminicola) | O_OTU33 (Aspergillus_subflavus) | 0.147507 | 0.14101005 | positive | 0.147507 | 0 | 0 | 0 | 0.85075 |
| O_OTU7942 (Fungi_spe_Incertae_sedis) | O_OTU33 (Aspergillus_subflavus) | 0.152013 | 0.12913316 | positive | 0.15201294 | 0 | 0 | 0 | 0.88896 |
| O_OTU8123 (Heitmania_litseae) | O_OTU33 (Aspergillus_subflavus) | 0.383071 | 7.6914E-05 | positive | 0.38307064 | 0.3 | 1 | 2 | 4.11394 |
| Neutralizing_antibody | O_OTU33 (Aspergillus_subflavus) | -0.268268 | 0.00667968 | negative | 0.26826755 | 0 | 1 | 2 | 2.17524 |
| lgM | O_OTU33 (Aspergillus_subflavus) | -0.301846 | 0.00215757 | negative | 0.30184557 | 0.3 | 1 | 2 | 2.66603 |
| lgG | O_OTU33 (Aspergillus_subflavus) | -0.257492 | 0.00933477 | negative | 0.25749224 | 0 | 1 | 2 | 2.0299 |
| MON# | O_OTU33 (Aspergillus_subflavus) | 0.39608 | 4.1316E-05 | positive | 0.39607979 | 0.3 | 1 | 2 | 4.38378 |
| MON_p.c. | O_OTU33 (Aspergillus_subflavus) | 0.422108 | 1.0994E-05 | positive | 0.42210841 | 0.3 | 1 | 2 | 4.95843 |
| IBIL | O_OTU33 (Aspergillus_subflavus) | -0.300573 | 0.00225756 | negative | 0.30057326 | 0.3 | 1 | 2 | 2.64636 |
| TP | O_OTU33 (Aspergillus_subflavus) | -0.077815 | 0.43924215 | negative | 0.07781529 | 0 | 0 | 0 | 0.3573 |
| ALB | O_OTU33 (Aspergillus_subflavus) | -0.193833 | 0.05211118 | negative | 0.19383317 | 0 | 0 | 0 | 1.28307 |
| GLOB | O_OTU33 (Aspergillus_subflavus) | 0.061456 | 0.54151917 | positive | 0.06145624 | 0 | 0 | 0 | 0.26639 |
| TBA | O_OTU33 (Aspergillus_subflavus) | 0.113783 | 0.25723265 | positive | 0.11378309 | 0 | 0 | 0 | 0.58967 |
| F_OTU3924 (Saccharomyces_cerevisiae) | O_OTU33 (Aspergillus_subflavus) | 0.24026 | 0.01551649 | positive | 0.24026044 | 0 | 1 | 1 | 1.80921 |
| F_OTU6998 (Calosphaeria_pulchella) | O_OTU33 (Aspergillus_subflavus) | -0.329581 | 0.00076385 | negative | 0.32958128 | 0.3 | 1 | 2 | 3.11699 |
| O_OTU95 (Zanclospora_jonesii) | O_OTU44 (Zanclospora_jonesii) | 0.528065 | 1.3869E-08 | positive | 0.52806479 | 0.5 | 1 | 2 | 7.62217 |
| O_OTU154 (Diversispora_spurca) | O_OTU44 (Zanclospora_jonesii) | 0.360747 | 0.00021061 | positive | 0.36074651 | 0.3 | 1 | 2 | 3.67649 |
| O_OTU599 (Aspergillus_subflavus) | O_OTU44 (Zanclospora_jonesii) | 0.020732 | 0.83695888 | positive | 0.02073223 | 0 | 0 | 0 | 0.0773 |
| O_OTU1734 (Acrodictys_fluminicola) | O_OTU44 (Zanclospora_jonesii) | 0.249623 | 0.01182111 | positive | 0.24962288 | 0 | 1 | 1 | 1.92734 |
| O_OTU5905 (Blumeria_spe_Incertae_sedis) | O_OTU44 (Zanclospora_jonesii) | 0.189937 | 0.05711364 | positive | 0.18993702 | 0 | 0 | 0 | 1.24326 |
| O_OTU5962 (Acrodictys_fluminicola) | O_OTU44 (Zanclospora_jonesii) | 0.323544 | 0.00096566 | positive | 0.32354403 | 0.3 | 1 | 2 | 3.01517 |
| O_OTU7942 (Fungi_spe_Incertae_sedis) | O_OTU44 (Zanclospora_jonesii) | 0.438869 | 4.4151E-06 | positive | 0.43886897 | 0.3 | 1 | 2 | 5.35407 |
| O_OTU8123 (Heitmania_litseae) | O_OTU44 (Zanclospora_jonesii) | 0.387701 | 6.1836E-05 | positive | 0.38770068 | 0.3 | 1 | 2 | 4.20869 |
| Neutralizing_antibody | O_OTU44 (Zanclospora_jonesii) | -0.123389 | 0.21895572 | negative | 0.1233887 | 0 | 0 | 0 | 0.65964 |
| lgM | O_OTU44 (Zanclospora_jonesii) | -0.386073 | 6.6791E-05 | negative | 0.38607298 | 0.3 | 1 | 2 | 4.17522 |
| lgG | O_OTU44 (Zanclospora_jonesii) | -0.359923 | 0.00021829 | negative | 0.35992314 | 0.3 | 1 | 2 | 3.66096 |
| MON# | O_OTU44 (Zanclospora_jonesii) | -0.202185 | 0.04259783 | negative | 0.20218481 | 0 | 1 | 1 | 1.37061 |
| MON_p.c. | O_OTU44 (Zanclospora_jonesii) | -0.25802 | 0.00918574 | negative | 0.25802026 | 0 | 1 | 2 | 2.03689 |
| IBIL | O_OTU44 (Zanclospora_jonesii) | -0.436833 | 4.9455E-06 | negative | 0.43683281 | 0.3 | 1 | 2 | 5.30491 |
| TP | O_OTU44 (Zanclospora_jonesii) | -0.521662 | 2.2203E-08 | negative | 0.52166232 | 0.5 | 1 | 2 | 7.4921 |
| ALB | O_OTU44 (Zanclospora_jonesii) | -0.509965 | 5.118E-08 | negative | 0.50996503 | 0.5 | 1 | 2 | 7.21339 |
| GLOB | O_OTU44 (Zanclospora_jonesii) | -0.459344 | 1.3535E-06 | negative | 0.45934381 | 0.3 | 1 | 2 | 5.86535 |
| TBA | O_OTU44 (Zanclospora_jonesii) | 0.125461 | 0.21125545 | positive | 0.12546115 | 0 | 0 | 0 | 0.67519 |
| F_OTU3924 (Saccharomyces_cerevisiae) | O_OTU44 (Zanclospora_jonesii) | -0.149395 | 0.13593773 | negative | 0.14939462 | 0 | 0 | 0 | 0.86666 |
| F_OTU6998 (Calosphaeria_pulchella) | O_OTU44 (Zanclospora_jonesii) | 0.049113 | 0.62574355 | positive | 0.04911262 | 0 | 0 | 0 | 0.2036 |
| O_OTU154 (Diversispora_spurca) | O_OTU95 (Zanclospora_jonesii) | 0.383384 | 7.5794E-05 | positive | 0.38338419 | 0.3 | 1 | 2 | 4.12031 |
| O_OTU599 (Aspergillus_subflavus) | O_OTU95 (Zanclospora_jonesii) | 0.086742 | 0.38839806 | positive | 0.08674234 | 0 | 0 | 0 | 0.41072 |
| O_OTU1734 (Acrodictys_fluminicola) | O_OTU95 (Zanclospora_jonesii) | 0.16398 | 0.10130043 | positive | 0.16398039 | 0 | 0 | 0 | 0.99439 |
| O_OTU5905 (Blumeria_spe_Incertae_sedis) | O_OTU95 (Zanclospora_jonesii) | 0.291532 | 0.00309719 | positive | 0.29153156 | 0 | 1 | 2 | 2.50903 |
| O_OTU5962 (Acrodictys_fluminicola) | O_OTU95 (Zanclospora_jonesii) | 0.178879 | 0.07348638 | positive | 0.17887878 | 0 | 0 | 0 | 1.13379 |
| O_OTU7942 (Fungi_spe_Incertae_sedis) | O_OTU95 (Zanclospora_jonesii) | 0.281879 | 0.00429372 | positive | 0.28187901 | 0 | 1 | 2 | 2.36717 |
| O_OTU8123 (Heitmania_litseae) | O_OTU95 (Zanclospora_jonesii) | 0.29649 | 0.00260729 | positive | 0.29649041 | 0 | 1 | 2 | 2.58381 |
| Neutralizing_antibody | O_OTU95 (Zanclospora_jonesii) | -0.211296 | 0.03391516 | negative | 0.21129619 | 0 | 1 | 1 | 1.46961 |
| lgM | O_OTU95 (Zanclospora_jonesii) | -0.292222 | 0.00302442 | negative | 0.29222152 | 0 | 1 | 2 | 2.51936 |
| lgG | O_OTU95 (Zanclospora_jonesii) | -0.495118 | 1.4135E-07 | negative | 0.49511767 | 0.3 | 1 | 2 | 6.82002 |
| MON# | O_OTU95 (Zanclospora_jonesii) | -0.102863 | 0.30602055 | negative | 0.10286268 | 0 | 0 | 0 | 0.51425 |
| MON_p.c. | O_OTU95 (Zanclospora_jonesii) | -0.059191 | 0.55655206 | negative | 0.05919069 | 0 | 0 | 0 | 0.25449 |
| IBIL | O_OTU95 (Zanclospora_jonesii) | -0.349666 | 0.00033812 | negative | 0.34966606 | 0.3 | 1 | 2 | 3.47092 |
| TP | O_OTU95 (Zanclospora_jonesii) | -0.43259 | 6.2498E-06 | negative | 0.43258964 | 0.3 | 1 | 2 | 5.20344 |
| ALB | O_OTU95 (Zanclospora_jonesii) | -0.439716 | 4.2108E-06 | negative | 0.43971574 | 0.3 | 1 | 2 | 5.37461 |
| GLOB | O_OTU95 (Zanclospora_jonesii) | -0.362671 | 0.00019365 | negative | 0.36267081 | 0.3 | 1 | 2 | 3.71296 |
| TBA | O_OTU95 (Zanclospora_jonesii) | 0.258365 | 0.00908961 | positive | 0.25836489 | 0 | 1 | 2 | 2.04145 |
| F_OTU3924 (Saccharomyces_cerevisiae) | O_OTU95 (Zanclospora_jonesii) | 0.053357 | 0.59615912 | positive | 0.05335676 | 0 | 0 | 0 | 0.22464 |
| F_OTU6998 (Calosphaeria_pulchella) | O_OTU95 (Zanclospora_jonesii) | -0.096685 | 0.33613211 | negative | 0.09668525 | 0 | 0 | 0 | 0.47349 |
| O_OTU599 (Aspergillus_subflavus) | O_OTU154 (Diversispora_spurca) | -0.169729 | 0.08972313 | negative | 0.16972915 | 0 | 0 | 0 | 1.0471 |
| O_OTU1734 (Acrodictys_fluminicola) | O_OTU154 (Diversispora_spurca) | 0.275463 | 0.00530231 | positive | 0.27546339 | 0 | 1 | 2 | 2.27553 |
| O_OTU5905 (Blumeria_spe_Incertae_sedis) | O_OTU154 (Diversispora_spurca) | 0.251046 | 0.01133255 | positive | 0.25104649 | 0 | 1 | 1 | 1.94567 |
| O_OTU5962 (Acrodictys_fluminicola) | O_OTU154 (Diversispora_spurca) | 0.1746 | 0.08075717 | positive | 0.17460021 | 0 | 0 | 0 | 1.09282 |
| O_OTU7942 (Fungi_spe_Incertae_sedis) | O_OTU154 (Diversispora_spurca) | 0.265146 | 0.0073698 | positive | 0.26514587 | 0 | 1 | 2 | 2.13254 |
| O_OTU8123 (Heitmania_litseae) | O_OTU154 (Diversispora_spurca) | 0.305112 | 0.0019189 | positive | 0.30511224 | 0.3 | 1 | 2 | 2.71694 |
| Neutralizing_antibody | O_OTU154 (Diversispora_spurca) | -0.17925 | 0.0728812 | negative | 0.17925002 | 0 | 0 | 0 | 1.13738 |
| lgM | O_OTU154 (Diversispora_spurca) | -0.085393 | 0.3958468 | negative | 0.08539328 | 0 | 0 | 0 | 0.40247 |
| lgG | O_OTU154 (Diversispora_spurca) | -0.254067 | 0.01035402 | negative | 0.25406717 | 0 | 1 | 1 | 1.98489 |
| MON# | O_OTU154 (Diversispora_spurca) | -0.224642 | 0.02391628 | negative | 0.22464205 | 0 | 1 | 1 | 1.62131 |
| MON_p.c. | O_OTU154 (Diversispora_spurca) | -0.149185 | 0.13649443 | negative | 0.14918486 | 0 | 0 | 0 | 0.86489 |
| IBIL | O_OTU154 (Diversispora_spurca) | -0.338758 | 0.00052995 | negative | 0.33875775 | 0.3 | 1 | 2 | 3.27576 |
| TP | O_OTU154 (Diversispora_spurca) | -0.462135 | 1.1451E-06 | negative | 0.46213484 | 0.3 | 1 | 2 | 5.93738 |
| ALB | O_OTU154 (Diversispora_spurca) | -0.466698 | 8.6847E-07 | negative | 0.46669758 | 0.3 | 1 | 2 | 6.05627 |
| GLOB | O_OTU154 (Diversispora_spurca) | -0.38688 | 6.429E-05 | negative | 0.38687991 | 0.3 | 1 | 2 | 4.19179 |
| TBA | O_OTU154 (Diversispora_spurca) | 0.235534 | 0.01773601 | positive | 0.23553391 | 0 | 1 | 1 | 1.75114 |
| F_OTU3924 (Saccharomyces_cerevisiae) | O_OTU154 (Diversispora_spurca) | -0.101642 | 0.31182612 | negative | 0.10164208 | 0 | 0 | 0 | 0.50609 |
| F_OTU6998 (Calosphaeria_pulchella) | O_OTU154 (Diversispora_spurca) | 0.146676 | 0.1432877 | positive | 0.14667609 | 0 | 0 | 0 | 0.84379 |
| O_OTU1734 (Acrodictys_fluminicola) | O_OTU599 (Aspergillus_subflavus) | 0.153829 | 0.12456753 | positive | 0.15382948 | 0 | 0 | 0 | 0.9046 |
| O_OTU5905 (Blumeria_spe_Incertae_sedis) | O_OTU599 (Aspergillus_subflavus) | 0.209805 | 0.03522472 | positive | 0.20980494 | 0 | 1 | 1 | 1.45315 |
| O_OTU5962 (Acrodictys_fluminicola) | O_OTU599 (Aspergillus_subflavus) | 0.243086 | 0.014308 | positive | 0.2430861 | 0 | 1 | 1 | 1.84442 |
| O_OTU7942 (Fungi_spe_Incertae_sedis) | O_OTU599 (Aspergillus_subflavus) | 0.074871 | 0.45680305 | positive | 0.07487077 | 0 | 0 | 0 | 0.34027 |
| O_OTU8123 (Heitmania_litseae) | O_OTU599 (Aspergillus_subflavus) | 0.146428 | 0.14397412 | positive | 0.14642764 | 0 | 0 | 0 | 0.84172 |
| Neutralizing_antibody | O_OTU599 (Aspergillus_subflavus) | -0.237358 | 0.01684916 | negative | 0.23735754 | 0 | 1 | 1 | 1.77342 |
| lgM | O_OTU599 (Aspergillus_subflavus) | -0.188852 | 0.05857418 | negative | 0.18885239 | 0 | 0 | 0 | 1.23229 |
| lgG | O_OTU599 (Aspergillus_subflavus) | -0.190828 | 0.0559366 | negative | 0.19082776 | 0 | 0 | 0 | 1.2523 |
| MON# | O_OTU599 (Aspergillus_subflavus) | 0.476783 | 4.6457E-07 | positive | 0.47678328 | 0.3 | 1 | 2 | 6.3237 |
| MON_p.c. | O_OTU599 (Aspergillus_subflavus) | 0.488245 | 2.2261E-07 | positive | 0.48824467 | 0.3 | 1 | 2 | 6.63337 |
| IBIL | O_OTU599 (Aspergillus_subflavus) | -0.222923 | 0.02504321 | negative | 0.22292271 | 0 | 1 | 1 | 1.60131 |
| TP | O_OTU599 (Aspergillus_subflavus) | 0.094551 | 0.34695874 | positive | 0.09455075 | 0 | 0 | 0 | 0.45972 |
| ALB | O_OTU599 (Aspergillus_subflavus) | -0.028542 | 0.77692098 | negative | 0.0285423 | 0 | 0 | 0 | 0.10962 |
| GLOB | O_OTU599 (Aspergillus_subflavus) | 0.195536 | 0.05004055 | positive | 0.19553648 | 0 | 0 | 0 | 1.30068 |
| TBA | O_OTU599 (Aspergillus_subflavus) | 0.007312 | 0.94215048 | positive | 0.00731163 | 0 | 0 | 0 | 0.02588 |
| F_OTU3924 (Saccharomyces_cerevisiae) | O_OTU599 (Aspergillus_subflavus) | 0.340795 | 0.00048789 | positive | 0.34079462 | 0.3 | 1 | 2 | 3.31167 |
| F_OTU6998 (Calosphaeria_pulchella) | O_OTU599 (Aspergillus_subflavus) | -0.350102 | 0.00033198 | negative | 0.35010222 | 0.3 | 1 | 2 | 3.47887 |
| O_OTU5905 (Blumeria_spe_Incertae_sedis) | O_OTU1734 (Acrodictys_fluminicola) | 0.16239 | 0.10470291 | positive | 0.16238953 | 0 | 0 | 0 | 0.98004 |
| O_OTU5962 (Acrodictys_fluminicola) | O_OTU1734 (Acrodictys_fluminicola) | 0.485463 | 2.6679E-07 | positive | 0.48546266 | 0.3 | 1 | 2 | 6.55785 |
| O_OTU7942 (Fungi_spe_Incertae_sedis) | O_OTU1734 (Acrodictys_fluminicola) | 0.500691 | 9.7081E-08 | positive | 0.50069077 | 0.5 | 1 | 2 | 6.97029 |
| O_OTU8123 (Heitmania_litseae) | O_OTU1734 (Acrodictys_fluminicola) | 0.16214 | 0.10524522 | positive | 0.16213972 | 0 | 0 | 0 | 0.9778 |
| Neutralizing_antibody | O_OTU1734 (Acrodictys_fluminicola) | -0.146417 | 0.14400436 | negative | 0.14641672 | 0 | 0 | 0 | 0.84162 |
| lgM | O_OTU1734 (Acrodictys_fluminicola) | -0.159445 | 0.11123469 | negative | 0.15944541 | 0 | 0 | 0 | 0.95376 |
| lgG | O_OTU1734 (Acrodictys_fluminicola) | -0.199339 | 0.04566216 | negative | 0.19933918 | 0 | 1 | 1 | 1.34044 |
| MON# | O_OTU1734 (Acrodictys_fluminicola) | -0.049078 | 0.62598736 | negative | 0.04907801 | 0 | 0 | 0 | 0.20343 |
| MON_p.c. | O_OTU1734 (Acrodictys_fluminicola) | 0.031822 | 0.75207324 | positive | 0.03182204 | 0 | 0 | 0 | 0.12374 |
| IBIL | O_OTU1734 (Acrodictys_fluminicola) | -0.427802 | 8.1083E-06 | negative | 0.42780213 | 0.3 | 1 | 2 | 5.09054 |
| TP | O_OTU1734 (Acrodictys_fluminicola) | -0.222289 | 0.02546993 | negative | 0.22228887 | 0 | 1 | 1 | 1.59397 |
| ALB | O_OTU1734 (Acrodictys_fluminicola) | -0.249799 | 0.01175969 | negative | 0.24979901 | 0 | 1 | 1 | 1.9296 |
| GLOB | O_OTU1734 (Acrodictys_fluminicola) | -0.12756 | 0.20365405 | negative | 0.12756044 | 0 | 0 | 0 | 0.69111 |
| TBA | O_OTU1734 (Acrodictys_fluminicola) | 0.172165 | 0.0851457 | positive | 0.17216496 | 0 | 0 | 0 | 1.06984 |
| F_OTU3924 (Saccharomyces_cerevisiae) | O_OTU1734 (Acrodictys_fluminicola) | -0.071072 | 0.4800211 | negative | 0.07107207 | 0 | 0 | 0 | 0.31874 |
| F_OTU6998 (Calosphaeria_pulchella) | O_OTU1734 (Acrodictys_fluminicola) | 0.097055 | 0.33427865 | positive | 0.097055 | 0 | 0 | 0 | 0.47589 |
| O_OTU5962 (Acrodictys_fluminicola) | O_OTU5905 (Blumeria_spe_Incertae_sedis) | 0.121199 | 0.22730554 | positive | 0.12119888 | 0 | 0 | 0 | 0.64339 |
| O_OTU7942 (Fungi_spe_Incertae_sedis) | O_OTU5905 (Blumeria_spe_Incertae_sedis) | 0.107463 | 0.28477943 | positive | 0.10746264 | 0 | 0 | 0 | 0.54549 |
| O_OTU8123 (Heitmania_litseae) | O_OTU5905 (Blumeria_spe_Incertae_sedis) | 0.34879 | 0.00035075 | positive | 0.34879039 | 0.3 | 1 | 2 | 3.45499 |
| Neutralizing_antibody | O_OTU5905 (Blumeria_spe_Incertae_sedis) | -0.484621 | 2.8173E-07 | negative | 0.48462077 | 0.3 | 1 | 2 | 6.53502 |
| lgM | O_OTU5905 (Blumeria_spe_Incertae_sedis) | -0.174407 | 0.08109894 | negative | 0.17440681 | 0 | 0 | 0 | 1.09098 |
| lgG | O_OTU5905 (Blumeria_spe_Incertae_sedis) | -0.398479 | 3.6737E-05 | negative | 0.39847857 | 0.3 | 1 | 2 | 4.43477 |
| MON# | O_OTU5905 (Blumeria_spe_Incertae_sedis) | 0.224361 | 0.02409731 | positive | 0.22436126 | 0 | 1 | 1 | 1.61803 |
| MON_p.c. | O_OTU5905 (Blumeria_spe_Incertae_sedis) | 0.289525 | 0.00331782 | positive | 0.28952545 | 0 | 1 | 2 | 2.47915 |
| IBIL | O_OTU5905 (Blumeria_spe_Incertae_sedis) | -0.217076 | 0.02922009 | negative | 0.21707612 | 0 | 1 | 1 | 1.53432 |
| TP | O_OTU5905 (Blumeria_spe_Incertae_sedis) | -0.265423 | 0.00730603 | negative | 0.26542322 | 0 | 1 | 2 | 2.13632 |
| ALB | O_OTU5905 (Blumeria_spe_Incertae_sedis) | -0.382311 | 7.9692E-05 | negative | 0.38231117 | 0.3 | 1 | 2 | 4.09853 |
| GLOB | O_OTU5905 (Blumeria_spe_Incertae_sedis) | -0.088626 | 0.37814022 | negative | 0.08862585 | 0 | 0 | 0 | 0.42235 |
| TBA | O_OTU5905 (Blumeria_spe_Incertae_sedis) | 0.254603 | 0.01018829 | positive | 0.25460333 | 0 | 1 | 1 | 1.9919 |
| F_OTU3924 (Saccharomyces_cerevisiae) | O_OTU5905 (Blumeria_spe_Incertae_sedis) | 0.286578 | 0.00366751 | positive | 0.28657837 | 0 | 1 | 2 | 2.43563 |
| F_OTU6998 (Calosphaeria_pulchella) | O_OTU5905 (Blumeria_spe_Incertae_sedis) | -0.274034 | 0.00555379 | negative | 0.27403438 | 0 | 1 | 2 | 2.25541 |
| O_OTU7942 (Fungi_spe_Incertae_sedis) | O_OTU5962 (Acrodictys_fluminicola) | 0.726264 | 0 | positive | 0.72626401 | 0.5 | 1 | 2 | 8 |
| O_OTU8123 (Heitmania_litseae) | O_OTU5962 (Acrodictys_fluminicola) | 0.232585 | 0.01925532 | positive | 0.23258506 | 0 | 1 | 1 | 1.71545 |
| Neutralizing_antibody | O_OTU5962 (Acrodictys_fluminicola) | -0.153801 | 0.12463845 | negative | 0.15380088 | 0 | 0 | 0 | 0.90435 |
| lgM | O_OTU5962 (Acrodictys_fluminicola) | -0.210124 | 0.0349412 | negative | 0.2101238 | 0 | 1 | 1 | 1.45666 |
| lgG | O_OTU5962 (Acrodictys_fluminicola) | -0.237396 | 0.0168309 | negative | 0.23739593 | 0 | 1 | 1 | 1.77389 |
| MON# | O_OTU5962 (Acrodictys_fluminicola) | 0.072917 | 0.4686644 | positive | 0.07291731 | 0 | 0 | 0 | 0.32914 |
| MON_p.c. | O_OTU5962 (Acrodictys_fluminicola) | 0.180745 | 0.07048537 | positive | 0.18074459 | 0 | 0 | 0 | 1.1519 |
| IBIL | O_OTU5962 (Acrodictys_fluminicola) | -0.414303 | 1.6544E-05 | negative | 0.41430269 | 0.3 | 1 | 2 | 4.78111 |
| TP | O_OTU5962 (Acrodictys_fluminicola) | -0.31289 | 0.00144374 | negative | 0.31289048 | 0.3 | 1 | 2 | 2.84051 |
| ALB | O_OTU5962 (Acrodictys_fluminicola) | -0.35307 | 0.00029289 | negative | 0.3530701 | 0.3 | 1 | 2 | 3.53328 |
| GLOB | O_OTU5962 (Acrodictys_fluminicola) | -0.181643 | 0.06907551 | negative | 0.18164337 | 0 | 0 | 0 | 1.16068 |
| TBA | O_OTU5962 (Acrodictys_fluminicola) | 0.08515 | 0.39719899 | positive | 0.08515 | 0 | 0 | 0 | 0.40099 |
| F_OTU3924 (Saccharomyces_cerevisiae) | O_OTU5962 (Acrodictys_fluminicola) | 0.065847 | 0.51296394 | positive | 0.06584719 | 0 | 0 | 0 | 0.28991 |
| F_OTU6998 (Calosphaeria_pulchella) | O_OTU5962 (Acrodictys_fluminicola) | -0.04575 | 0.64961609 | negative | 0.04574988 | 0 | 0 | 0 | 0.18734 |
| O_OTU8123 (Heitmania_litseae) | O_OTU7942 (Fungi_spe_Incertae_sedis) | 0.375168 | 0.00011079 | positive | 0.37516782 | 0.3 | 1 | 2 | 3.95544 |
| Neutralizing_antibody | O_OTU7942 (Fungi_spe_Incertae_sedis) | -0.139825 | 0.16313799 | negative | 0.13982511 | 0 | 0 | 0 | 0.78744 |
| lgM | O_OTU7942 (Fungi_spe_Incertae_sedis) | -0.255197 | 0.01000751 | negative | 0.25519699 | 0 | 1 | 1 | 1.99967 |
| lgG | O_OTU7942 (Fungi_spe_Incertae_sedis) | -0.20739 | 0.03743592 | negative | 0.20738953 | 0 | 1 | 1 | 1.42671 |
| MON# | O_OTU7942 (Fungi_spe_Incertae_sedis) | -0.039506 | 0.69488096 | negative | 0.03950583 | 0 | 0 | 0 | 0.15809 |
| MON_p.c. | O_OTU7942 (Fungi_spe_Incertae_sedis) | -0.013261 | 0.89528411 | negative | 0.01326127 | 0 | 0 | 0 | 0.04804 |
| IBIL | O_OTU7942 (Fungi_spe_Incertae_sedis) | -0.425924 | 8.9703E-06 | negative | 0.4259243 | 0.3 | 1 | 2 | 5.04671 |
| TP | O_OTU7942 (Fungi_spe_Incertae_sedis) | -0.408449 | 2.2328E-05 | negative | 0.4084489 | 0.3 | 1 | 2 | 4.65096 |
| ALB | O_OTU7942 (Fungi_spe_Incertae_sedis) | -0.411744 | 1.8873E-05 | negative | 0.41174425 | 0.3 | 1 | 2 | 4.72393 |
| GLOB | O_OTU7942 (Fungi_spe_Incertae_sedis) | -0.277598 | 0.00494552 | negative | 0.27759795 | 0 | 1 | 2 | 2.30579 |
| TBA | O_OTU7942 (Fungi_spe_Incertae_sedis) | 0.157903 | 0.11478011 | positive | 0.15790331 | 0 | 0 | 0 | 0.94013 |
| F_OTU3924 (Saccharomyces_cerevisiae) | O_OTU7942 (Fungi_spe_Incertae_sedis) | -0.158718 | 0.11289669 | negative | 0.15871784 | 0 | 0 | 0 | 0.94732 |
| F_OTU6998 (Calosphaeria_pulchella) | O_OTU7942 (Fungi_spe_Incertae_sedis) | -0.012058 | 0.90473929 | negative | 0.01205793 | 0 | 0 | 0 | 0.04348 |
| Neutralizing_antibody | O_OTU8123 (Heitmania_litseae) | -0.37666 | 0.00010349 | negative | 0.3766601 | 0.3 | 1 | 2 | 3.98506 |
| lgM | O_OTU8123 (Heitmania_litseae) | -0.312691 | 0.00145447 | negative | 0.31269079 | 0.3 | 1 | 2 | 2.83729 |
| lgG | O_OTU8123 (Heitmania_litseae) | -0.51079 | 4.8302E-08 | negative | 0.51078998 | 0.5 | 1 | 2 | 7.23432 |
| MON# | O_OTU8123 (Heitmania_litseae) | 0.065476 | 0.51534471 | positive | 0.06547645 | 0 | 0 | 0 | 0.2879 |
| MON_p.c. | O_OTU8123 (Heitmania_litseae) | 0.08811 | 0.38093216 | positive | 0.08811018 | 0 | 0 | 0 | 0.41915 |
| IBIL | O_OTU8123 (Heitmania_litseae) | -0.334428 | 0.0006306 | negative | 0.33442827 | 0.3 | 1 | 2 | 3.20024 |
| TP | O_OTU8123 (Heitmania_litseae) | -0.415957 | 1.5184E-05 | negative | 0.41595687 | 0.3 | 1 | 2 | 4.81832 |
| ALB | O_OTU8123 (Heitmania_litseae) | -0.503293 | 8.1276E-08 | negative | 0.50329261 | 0.5 | 1 | 2 | 7.03964 |
| GLOB | O_OTU8123 (Heitmania_litseae) | -0.276953 | 0.00505099 | negative | 0.27695302 | 0 | 1 | 2 | 2.29662 |
| TBA | O_OTU8123 (Heitmania_litseae) | 0.175971 | 0.07836751 | positive | 0.17597106 | 0 | 0 | 0 | 1.10586 |
| F_OTU3924 (Saccharomyces_cerevisiae) | O_OTU8123 (Heitmania_litseae) | 0.012537 | 0.90096993 | positive | 0.01253743 | 0 | 0 | 0 | 0.04529 |
| F_OTU6998 (Calosphaeria_pulchella) | O_OTU8123 (Heitmania_litseae) | -0.196075 | 0.04940076 | negative | 0.19607454 | 0 | 1 | 1 | 1.30627 |
| lgM | Neutralizing_antibody | 0.382918 | 7.7466E-05 | positive | 0.38291789 | 0.3 | 1 | 2 | 4.11084 |
| lgG | Neutralizing_antibody | 0.498467 | 1.1287E-07 | positive | 0.49846731 | 0.3 | 1 | 2 | 6.91056 |
| MON# | Neutralizing_antibody | -0.352387 | 0.00030149 | negative | 0.35238698 | 0.3 | 1 | 2 | 3.52071 |
| MON_p.c. | Neutralizing_antibody | -0.348261 | 0.0003586 | negative | 0.34826082 | 0.3 | 1 | 2 | 3.44538 |
| IBIL | Neutralizing_antibody | 0.300115 | 0.00229456 | positive | 0.30011535 | 0.3 | 1 | 2 | 2.6393 |
| TP | Neutralizing_antibody | 0.342321 | 0.0004584 | positive | 0.34232146 | 0.3 | 1 | 2 | 3.33874 |
| ALB | Neutralizing_antibody | 0.503365 | 8.0872E-08 | positive | 0.50336531 | 0.5 | 1 | 2 | 7.04157 |
| GLOB | Neutralizing_antibody | 0.130373 | 0.19378013 | positive | 0.13037299 | 0 | 0 | 0 | 0.71269 |
| TBA | Neutralizing_antibody | -0.273219 | 0.00570201 | negative | 0.27321889 | 0 | 1 | 2 | 2.24397 |
| F_OTU3924 (Saccharomyces_cerevisiae) | Neutralizing_antibody | -0.304804 | 0.00194039 | negative | 0.30480354 | 0.3 | 1 | 2 | 2.71211 |
| F_OTU6998 (Calosphaeria_pulchella) | Neutralizing_antibody | 0.352897 | 0.00029504 | positive | 0.35289744 | 0.3 | 1 | 2 | 3.5301 |
| lgG | lgM | 0.386439 | 6.5645E-05 | positive | 0.38643925 | 0.3 | 1 | 2 | 4.18273 |
| MON# | lgM | -0.18153 | 0.06925264 | negative | 0.18152964 | 0 | 0 | 0 | 1.15956 |
| MON_p.c. | lgM | -0.089739 | 0.3721583 | negative | 0.08973855 | 0 | 0 | 0 | 0.42927 |
| IBIL | lgM | 0.34309 | 0.00044419 | positive | 0.34308993 | 0.3 | 1 | 2 | 3.35242 |
| TP | lgM | 0.283681 | 0.00404316 | positive | 0.28368073 | 0 | 1 | 2 | 2.39328 |
| ALB | lgM | 0.332015 | 0.00069401 | positive | 0.33201542 | 0.3 | 1 | 2 | 3.15863 |
| GLOB | lgM | 0.189769 | 0.0573385 | positive | 0.18976856 | 0 | 0 | 0 | 1.24155 |
| TBA | lgM | -0.031379 | 0.7554149 | negative | 0.0313791 | 0 | 0 | 0 | 0.12181 |
| F_OTU3924 (Saccharomyces_cerevisiae) | lgM | -0.104886 | 0.29655369 | negative | 0.10488593 | 0 | 0 | 0 | 0.5279 |
| F_OTU6998 (Calosphaeria_pulchella) | lgM | 0.189595 | 0.0575712 | positive | 0.18959481 | 0 | 0 | 0 | 1.23979 |
| MON# | lgG | -0.212801 | 0.03263589 | negative | 0.21280051 | 0 | 1 | 1 | 1.4863 |
| MON_p.c. | lgG | -0.207463 | 0.03736686 | negative | 0.20746313 | 0 | 1 | 1 | 1.42751 |
| IBIL | lgG | 0.333964 | 0.00064237 | positive | 0.33396392 | 0.3 | 1 | 2 | 3.19221 |
| TP | lgG | 0.376836 | 0.00010266 | positive | 0.3768357 | 0.3 | 1 | 2 | 3.98856 |
| ALB | lgG | 0.446827 | 2.8139E-06 | positive | 0.44682725 | 0.3 | 1 | 2 | 5.54916 |
| GLOB | lgG | 0.274505 | 0.00546988 | positive | 0.2745047 | 0 | 1 | 2 | 2.26202 |
| TBA | lgG | -0.065679 | 0.51404463 | negative | 0.06567879 | 0 | 0 | 0 | 0.289 |
| F_OTU3924 (Saccharomyces_cerevisiae) | lgG | -0.134481 | 0.17998867 | negative | 0.1344806 | 0 | 0 | 0 | 0.74475 |
| F_OTU6998 (Calosphaeria_pulchella) | lgG | 0.338559 | 0.00053423 | positive | 0.33855876 | 0.3 | 1 | 2 | 3.27226 |
| MON_p.c. | MON# | 0.817086 | 0 | positive | 0.81708647 | 0.8 | 1 | 2 | 8 |
| IBIL | MON# | -0.159297 | 0.11157183 | negative | 0.15929715 | 0 | 0 | 0 | 0.95245 |
| TP | MON# | 0.235507 | 0.01774931 | positive | 0.23550717 | 0 | 1 | 1 | 1.75082 |
| ALB | MON# | 0.030796 | 0.75982424 | positive | 0.03079555 | 0 | 0 | 0 | 0.11929 |
| GLOB | MON# | 0.402244 | 3.0496E-05 | positive | 0.40224374 | 0.3 | 1 | 2 | 4.51561 |
| TBA | MON# | 0.006914 | 0.94529473 | positive | 0.00691359 | 0 | 0 | 0 | 0.02443 |
| F_OTU3924 (Saccharomyces_cerevisiae) | MON# | 0.423057 | 1.0455E-05 | positive | 0.42305688 | 0.3 | 1 | 2 | 4.98027 |
| F_OTU6998 (Calosphaeria_pulchella) | MON# | -0.438866 | 4.4159E-06 | negative | 0.43886578 | 0.3 | 1 | 2 | 5.354 |
| IBIL | MON_p.c. | -0.053924 | 0.59225449 | negative | 0.0539238 | 0 | 0 | 0 | 0.22749 |
| TP | MON_p.c. | 0.206646 | 0.03813946 | positive | 0.20664611 | 0 | 1 | 1 | 1.41863 |
| ALB | MON_p.c. | 0.028684 | 0.77583891 | positive | 0.02868448 | 0 | 0 | 0 | 0.11023 |
| GLOB | MON_p.c. | 0.359102 | 0.00022619 | positive | 0.35910175 | 0.3 | 1 | 2 | 3.6455 |
| TBA | MON_p.c. | 0.035195 | 0.72677772 | positive | 0.03519541 | 0 | 0 | 0 | 0.1386 |
| F_OTU3924 (Saccharomyces_cerevisiae) | MON_p.c. | 0.487386 | 2.3544E-07 | positive | 0.4873862 | 0.3 | 1 | 2 | 6.61005 |
| F_OTU6998 (Calosphaeria_pulchella) | MON_p.c. | -0.4059 | 2.5398E-05 | negative | 0.40589987 | 0.3 | 1 | 2 | 4.59504 |
| TP | IBIL | 0.470282 | 6.9694E-07 | positive | 0.47028171 | 0.3 | 1 | 2 | 6.15062 |
| ALB | IBIL | 0.605835 | 1.9134E-11 | positive | 0.6058348 | 0.5 | 1 | 2 | 7.99917 |
| GLOB | IBIL | 0.250354 | 0.01156797 | positive | 0.25035395 | 0 | 1 | 1 | 1.93674 |
| TBA | IBIL | -0.097148 | 0.33381556 | negative | 0.09714759 | 0 | 0 | 0 | 0.47649 |
| F_OTU3924 (Saccharomyces_cerevisiae) | IBIL | 0.025494 | 0.80022176 | positive | 0.02549366 | 0 | 0 | 0 | 0.09679 |
| F_OTU6998 (Calosphaeria_pulchella) | IBIL | 0.200017 | 0.04491664 | positive | 0.20001654 | 0 | 1 | 1 | 1.34759 |
| ALB | TP | 0.890786 | 0 | positive | 0.89078629 | 0.8 | 1 | 2 | 8 |
| GLOB | TP | 0.890049 | 0 | positive | 0.89004914 | 0.8 | 1 | 2 | 8 |
| TBA | TP | -0.265637 | 0.0072571 | negative | 0.26563747 | 0 | 1 | 2 | 2.13924 |
| F_OTU3924 (Saccharomyces_cerevisiae) | TP | 0.165687 | 0.09774645 | positive | 0.16568748 | 0 | 0 | 0 | 1.0099 |
| F_OTU6998 (Calosphaeria_pulchella) | TP | 0.047452 | 0.63748443 | positive | 0.04745226 | 0 | 0 | 0 | 0.19553 |
| GLOB | ALB | 0.616181 | 6.9349E-12 | positive | 0.61618142 | 0.5 | 1 | 2 | 7.9997 |
| TBA | ALB | -0.259812 | 0.00869551 | negative | 0.25981209 | 0 | 1 | 2 | 2.0607 |
| F_OTU3924 (Saccharomyces_cerevisiae) | ALB | 0.072103 | 0.47365787 | positive | 0.07210302 | 0 | 0 | 0 | 0.32454 |
| F_OTU6998 (Calosphaeria_pulchella) | ALB | 0.145735 | 0.14590063 | positive | 0.14573508 | 0 | 0 | 0 | 0.83594 |
| TBA | GLOB | -0.177653 | 0.07551401 | negative | 0.1776526 | 0 | 0 | 0 | 1.12197 |
| F_OTU3924 (Saccharomyces_cerevisiae) | GLOB | 0.199558 | 0.04541988 | positive | 0.1995583 | 0 | 1 | 1 | 1.34275 |
| F_OTU6998 (Calosphaeria_pulchella) | GLOB | -0.048361 | 0.63104606 | negative | 0.04836115 | 0 | 0 | 0 | 0.19994 |
| F_OTU3924 (Saccharomyces_cerevisiae) | TBA | 0.086025 | 0.39234753 | positive | 0.08602514 | 0 | 0 | 0 | 0.40633 |
| F_OTU6998 (Calosphaeria_pulchella) | TBA | -0.069374 | 0.49060404 | negative | 0.06937354 | 0 | 0 | 0 | 0.30927 |
| F_OTU6998 (Calosphaeria_pulchella) | F_OTU3924 (Saccharomyces_cerevisiae) | -0.513714 | 3.9295E-08 | negative | 0.51371406 | 0.5 | 1 | 2 | 7.3072 |

| Data S19. Correlation analysis between oral bacteria and oral fungi | | | | | | | |
| --- | --- | --- | --- | --- | --- | --- | --- |
| rho | I_OTU5.Actinomucor_elegans. | I_OTU7.Acrodictys_fluminicola. | I_OTU13.Cryptococcus_longus. | I_OTU27.Candida_albicans. | I_OTU29.Blumeria_spe_Incertae_sedis. | I_OTU30.Aspergillus_subflavus. | I_OTU33.Aspergillus_subflavus. |
| B_OTU5 (Veillonella_unclassified) | 0.2228501 | -0.13099 | 0.1224745 | 0.2770032 | 0.0970748 | 0.1851684 | -0.088462 |
| B_OTU7 (Porphyromonas_pasteri) | -0.070937 | 0.0360893 | -0.193967 | -0.224207 | 0.0976663 | -0.00222 | 0.105611 |
| B_OTU9 (Halomonas_unclassified) | -0.264979 | -0.103543 | -0.029768 | -0.251543 | -0.097169 | -0.090629 | 0.221486 |
| B_OTU10 (Rothia_unclassified) | -0.128807 | 0.208946 | -0.153093 | -0.125275 | 0.1426281 | -0.035987 | 0.3023254 |
| B_OTU14 (Leptotrichia_unclassified) | -0.085261 | 0.0279907 | 0.122584 | -0.088134 | -0.099921 | 0.1350335 | 0.1013148 |
| B_OTU15 (Peptostreptococcus_unclassified) | -0.40214 | 0.2566247 | -0.061239 | -0.091008 | -0.107012 | -0.023939 | 0.1431604 |
| B_OTU20 (Lachnoanaerobaculum_unclassified) | 0.2641548 | 0.0886789 | -0.112268 | 0.3396409 | -0.328694 | -0.192302 | -0.158376 |
| B_OTU24 (Veillonella_unclassified) | 0.0775821 | 0.0085513 | 0.1225057 | 0.3159606 | 0.0322658 | 0.2278723 | -0.015937 |
| B_OTU30 (Enterobacteriaceae_unclassified) | -0.400432 | -0.054175 | -0.058706 | -0.193502 | -0.020671 | -0.17873 | 0.1189362 |
| B_OTU43 (Prevotella_shahii) | 0.1154239 | -0.004929 | -0.091903 | 0.0368249 | -0.40573 | -0.09168 | 0.0225234 |
| B_OTU46 (Capnocytophaga_leadbetteri) | -0.077982 | -0.006521 | 0.1122797 | -0.240791 | -0.215626 | -0.22863 | 0.0807023 |
| B_OTU48 (Capnocytophaga_sputigena) | 0.0139127 | -0.05364 | 0.1838055 | -0.122905 | 0.0148899 | -0.12943 | -0.016496 |
| B_OTU54 (Leptotrichia_unclassified) | 0.0537851 | 0.1238995 | 0.1605212 | 0.0970768 | 0.1251914 | -0.009392 | 0.1604918 |
| B_OTU56 (Lactobacillus_unclassified) | -0.129742 | 0.1552768 | -0.048566 | 0.0670021 | 0.2031052 | -0.14786 | -0.14786 |
| B_OTU59 (Capnocytophaga_gingivalis) | -0.023897 | 0.0994015 | 0.2143304 | -0.180148 | -0.146028 | -0.336886 | 0.0545359 |
| B_OTU62 (Campylobacter_showae) | -0.008119 | -0.070994 | 0.2145275 | -0.070312 | -0.414516 | -0.203904 | 0.0379246 |
| B_OTU66 (Amnipila_unclassified) | -0.351527 | 0.3432727 | 0.2013036 | -0.253385 | -0.200738 | -0.053504 | 0.0826886 |
| B_OTU67 (Actinomyces_unclassified) | -0.51468 | 0.0893063 | 0.2144453 | -0.465476 | 0.0575202 | 0.0873994 | 0.1675816 |
| B_OTU73 (Megasphaera_micronuciformis) | -0.002426 | 0.0490114 | 0.1841161 | 0.1927882 | -0.035736 | 0.1862115 | -0.026851 |
| B_OTU108 (Leptotrichia_hongkongensis) | 0.290513 | -0.155703 | -0.06124 | 0.4645257 | 0.1062966 | -0.062228 | -0.222911 |
| B_OTU115 (Prevotella_unclassified) | 0.0531295 | -0.000953 | -0.180728 | 0.0541095 | -0.022397 | -0.097693 | -0.148786 |
| B_OTU138 (Prevotella_unclassified) | -0.312155 | 0.1486676 | -0.08165 | 0.1083993 | 0.022059 | -0.064682 | 0.1609126 |
| B_OTU156 (Neisseria_unclassified) | -0.180109 | 0.2996744 | 0.1336594 | -0.252503 | 0.2378182 | -0.001437 | 0.0010381 |
| B_OTU179 (Porphyromonas_unclassified) | -0.093267 | 0.1275772 | -0.173594 | -0.238248 | -0.044745 | -0.134823 | 0.112617 |
| B_OTU188 (Actinomyces_unclassified) | -0.144116 | 0.0710353 | 0 | -0.046173 | 0.0699034 | -0.118705 | 0.0798047 |
| B_OTU200 (Leptotrichia_unclassified) | 0.0119848 | -0.075028 | 0.2056299 | 0.1183249 | -0.373241 | -0.157947 | 0.1285617 |
| B_OTU246 (Haemophilus_unclassified) | -0.316953 | 0.0833197 | 0.0714453 | -0.209712 | 0.2375182 | 0.1620265 | 0.073879 |
| B_OTU316 (Bacteria_unclassified) | 0.2521465 | -0.092849 | -0.127257 | 0.1736005 | -0.121633 | -0.022345 | -0.055262 |
| B_OTU317 (Actinomyces_unclassified) | -0.249267 | 0.1310821 | 0.244905 | -0.168066 | -0.093719 | 0.0315744 | 0.2539268 |
| B_OTU403 (Actinomyces_unclassified) | 0.1689584 | -0.121218 | 0.2232877 | 0.0337333 | 0.1392234 | -0.008258 | -0.058136 |
| B_OTU544 (Porphyromonas_unclassified) | -0.244245 | 0.1894812 | -0.163383 | -0.287866 | 0.0370357 | -0.081211 | 0.1424367 |
| B_OTU899 (Streptococcus_unclassified) | 0.280853 | -0.191708 | -0.031415 | 0.1681288 | 0.1001418 | -0.035053 | 0.0374112 |
| B_OTU1066 (Streptococcus_unclassified) | -0.560756 | 0.2758899 | -0.010206 | -0.201424 | 0.0697276 | 0.0714991 | 0.2091071 |
| B_OTU1226 (Burkholderiales_unclassified) | 0.1802422 | -0.073866 | 0 | 0.1815924 | 0.0436635 | -0.011139 | -0.186432 |
| B_OTU1247 (Streptococcus_unclassified) | -0.248943 | 0.218711 | -0.114622 | 0.1828408 | -0.085859 | -0.009258 | 0.1940676 |
| B_OTU1823 (Streptococcus_unclassified) | 0.4089768 | -0.108509 | -0.204768 | 0.2851467 | 0.0148534 | 0.0485055 | -0.171439 |
| B_OTU1912 (Neisseria_unclassified) | -0.387927 | 0.1122448 | 0.2323693 | -0.436192 | 0.0230966 | -0.011945 | 0.0006016 |
| B_OTU1966 (Veillonella_unclassified) | 0.4458546 | -0.285308 | -0.183712 | 0.2390583 | -0.03883 | -0.062146 | -0.139986 |

| Data S19. Correlation analysis between oral bacteria and oral fungi | | | | | | | |
| --- | --- | --- | --- | --- | --- | --- | --- |
| rho | I_OTU35.Zanclospora_jonesii. | I_OTU36.Candida_parapsilosis. | I_OTU38.Zanclospora_jonesii. | I_OTU44.Zanclospora_jonesii. | I_OTU50.Mucor_circinelloides. | I_OTU51.Talaromyces_scorteus. | I_OTU54.Rhodotorula_mucilaginosa. |
| B_OTU5 (Veillonella_unclassified) | -0.183929 | 0.2138667 | -0.043506 | 0.0650572 | 0.1064986 | -0.061237 | -0.018319 |
| B_OTU7 (Porphyromonas_pasteri) | 0.0270902 | -0.227712 | -0.02953 | 0.0109435 | -0.096991 | -0.071462 | 0.0276465 |
| B_OTU9 (Halomonas_unclassified) | -0.042535 | 0.205796 | -0.069395 | -0.083872 | -0.187906 | -0.029768 | -0.200202 |
| B_OTU10 (Rothia_unclassified) | -0.141071 | 0.0841566 | -0.074776 | 0.1525826 | -0.072314 | 0.1530931 | -0.320467 |
| B_OTU14 (Leptotrichia_unclassified) | -0.081621 | -0.141168 | 0.1748609 | -0.042459 | -0.188239 | 0.0102153 | -0.028635 |
| B_OTU15 (Peptostreptococcus_unclassified) | 0.1422655 | -0.275594 | 0.0033505 | 0.1063913 | -0.341089 | -0.040826 | -0.18055 |
| B_OTU20 (Lachnoanaerobaculum_unclassified) | 0.0904762 | -0.13039 | 0.0921594 | -0.022552 | 0.1612818 | 0 | 0.1136827 |
| B_OTU24 (Veillonella_unclassified) | -0.212852 | 0.2237823 | -0.047451 | 0.0410066 | -0.090032 | 0.1327146 | -0.184148 |
| B_OTU30 (Enterobacteriaceae_unclassified) | -0.083883 | 0.2901425 | -0.136854 | 0.0094379 | -0.370572 | 0.3186893 | -0.39482 |
| B_OTU43 (Prevotella_shahii) | 0.2775226 | -0.150864 | -0.13282 | 0.0920996 | 0.2991595 | -0.153171 | 0.2641373 |
| B_OTU46 (Capnocytophaga_leadbetteri) | 0.0208355 | -0.026368 | -0.047201 | 0.0509778 | 0.0017532 | -0.010207 | 0.0351321 |
| B_OTU48 (Capnocytophaga_sputigena) | -0.075336 | -0.047088 | -0.157693 | 0.121709 | 0.0531668 | 0.0102114 | -0.151798 |
| B_OTU54 (Leptotrichia_unclassified) | -0.045405 | 0.0634868 | -0.075604 | 0.0025053 | 0.0613473 | 0.2140283 | 0.1367677 |
| B_OTU56 (Lactobacillus_unclassified) | -0.069395 | 0.1844123 | -0.113216 | -0.136835 | -0.168611 | 0.4079558 | -0.030971 |
| B_OTU59 (Capnocytophaga_gingivalis) | 0.277381 | -0.102816 | 0.0728341 | 0.1603794 | 0.0653016 | -0.071443 | 0.0064654 |
| B_OTU62 (Campylobacter_showae) | 0.1784378 | -0.135538 | 0.0899599 | 0.0214399 | 0.1368098 | -0.071509 | 0.2201325 |
| B_OTU66 (Amnipila_unclassified) | 0.3029641 | -0.258352 | 0.1988829 | -0.058242 | -0.312632 | 0.0559177 | -0.109337 |
| B_OTU67 (Actinomyces_unclassified) | -0.043178 | 0.0141344 | -0.038963 | 0.0930246 | -0.448696 | -0.020423 | -0.416862 |
| B_OTU73 (Megasphaera_micronuciformis) | -0.151374 | 0.0952442 | -0.019611 | 0.0711659 | -0.130451 | 0.0920581 | -0.239043 |
| B_OTU108 (Leptotrichia_hongkongensis) | -0.110125 | 0.2143309 | -0.072595 | 0.0132888 | 0.2507008 | 0.112274 | 0.1076 |
| B_OTU115 (Prevotella_unclassified) | 0.2273687 | 0.2263417 | 0.0599413 | -0.095817 | 0.0911877 | 0.2272009 | 0.0870643 |
| B_OTU138 (Prevotella_unclassified) | -0.147917 | 0.1475385 | 0.2471503 | 0.2577137 | -0.435965 | 0.1428869 | -0.279088 |
| B_OTU156 (Neisseria_unclassified) | 0.1286204 | 0.0797928 | 0.0370281 | -0.072378 | -0.154138 | 0.1542224 | -0.289288 |
| B_OTU179 (Porphyromonas_unclassified) | 0.0786115 | -0.166132 | 0.0515929 | 0.050747 | -0.091973 | 0.0408457 | 0.0213466 |
| B_OTU188 (Actinomyces_unclassified) | -0.036137 | 0.3072083 | -0.018965 | -0.189551 | -0.028381 | -0.030981 | -0.133782 |
| B_OTU200 (Leptotrichia_unclassified) | -0.154704 | 0.041095 | -0.01531 | 0.0023647 | 0.0920525 | -0.190208 | -0.033597 |
| B_OTU246 (Haemophilus_unclassified) | -0.233042 | -0.048425 | 0.0502082 | 0.068622 | -0.31923 | -0.040826 | -0.267889 |
| B_OTU316 (Bacteria_unclassified) | 0.0233948 | 0.0253504 | -0.180365 | -0.137392 | 0.2219093 | 0.1881198 | 0.1305601 |
| B_OTU317 (Actinomyces_unclassified) | 0.0921263 | -0.019487 | -0.057092 | -0.159831 | -0.264621 | 0.1102072 | -0.305757 |
| B_OTU403 (Actinomyces_unclassified) | 0.1389057 | 0.0374227 | -0.178465 | -0.092406 | 0.2601375 | -0.148858 | -0.015155 |
| B_OTU544 (Porphyromonas_unclassified) | 0.057172 | -0.113526 | 0.1209663 | 0.0882411 | -0.223849 | 0.0510571 | -0.071263 |
| B_OTU899 (Streptococcus_unclassified) | -0.125655 | 0.1459498 | -0.251087 | -0.221837 | 0.2573198 | 0.1989609 | -0.026866 |
| B_OTU1066 (Streptococcus_unclassified) | -0.115476 | 0.1487472 | 0.1753844 | 0.1447858 | -0.553639 | 0.0816497 | -0.427145 |
| B_OTU1226 (Burkholderiales_unclassified) | 0.0641064 | 0.1457904 | -0.09418 | -0.324833 | 0.1169903 | 0.2512449 | -0.114063 |
| B_OTU1247 (Streptococcus_unclassified) | 0.053479 | 0.1053725 | -0.025303 | -0.057716 | -0.260865 | 0.2407056 | -0.253045 |
| B_OTU1823 (Streptococcus_unclassified) | 0.0111959 | 0.2079099 | -0.007306 | 0.0638326 | 0.2908817 | 0.2252444 | 0.2150026 |
| B_OTU1912 (Neisseria_unclassified) | -0.015327 | -0.108767 | -0.018478 | -0.045992 | -0.344545 | 0.2213041 | -0.472383 |
| B_OTU1966 (Veillonella_unclassified) | -0.110714 | -0.024854 | 0.1421721 | 0.1929918 | 0.316647 | -0.204124 | 0.1364192 |

| Data S19. Correlation analysis between oral bacteria and oral fungi | | | | | | | |
| --- | --- | --- | --- | --- | --- | --- | --- |
| rho | I_OTU67.Zanclospora_jonesii. | I_OTU79.Phaeosphaeria_oryzae. | I_OTU91.Aspergillus_subflavus. | I_OTU95.Zanclospora_jonesii. | I_OTU119.Nigrospora_oryzae. | I_OTU130.Ascomycota_spe_Incertae_sedis. | I_OTU154.Diversispora_spurca. |
| B_OTU5 (Veillonella_unclassified) | 0.3025366 | 0.2064603 | 0 | -0.085714 | 0.3358461 | -0.093452 | 0.2464286 |
| B_OTU7 (Porphyromonas_pasteri) | -0.07374 | -0.082809 | 0.0816705 | 0.2834056 | -0.218448 | -0.083057 | -0.241431 |
| B_OTU9 (Halomonas_unclassified) | -0.212693 | -0.069395 | -0.029768 | -0.042535 | 0.1584061 | -0.042535 | -0.042535 |
| B_OTU10 (Rothia_unclassified) | -0.26852 | -0.084488 | -0.091856 | 0.0181548 | 0.0846511 | 0.1056548 | -0.225893 |
| B_OTU14 (Leptotrichia_unclassified) | -0.076074 | -0.036498 | 0.1736606 | -0.00283 | 0.0359875 | -0.000894 | 0.2037534 |
| B_OTU15 (Peptostreptococcus_unclassified) | -0.393967 | -0.236474 | 0.2143358 | -0.032441 | -0.365047 | 0.1220269 | -0.173219 |
| B_OTU20 (Lachnoanaerobaculum_unclassified) | 0.337829 | 0.1614974 | -0.071443 | 0.0678571 | 0.1083586 | -0.195833 | -0.124702 |
| B_OTU24 (Veillonella_unclassified) | 0.1501373 | 0.0980111 | -0.010209 | 0.0116101 | 0.1940115 | -0.17564 | 0.2536361 |
| B_OTU30 (Enterobacteriaceae_unclassified) | -0.419454 | -0.136854 | 0.3690086 | 0.246269 | -0.062867 | 0.1684998 | -0.083883 |
| B_OTU43 (Prevotella_shahii) | 0.1569825 | 0.2520374 | 0.1531713 | -0.170921 | 0.106837 | 0.0092309 | -0.081291 |
| B_OTU46 (Capnocytophaga_leadbetteri) | -0.026578 | 0.0016511 | -0.030622 | 0.0184543 | -0.027651 | 0.0395874 | 0.1247151 |
| B_OTU48 (Capnocytophaga_sputigena) | 0.007445 | 0.1709074 | 0.0714799 | 0.0279905 | -0.194028 | -0.013697 | 0.0768249 |
| B_OTU54 (Leptotrichia_unclassified) | 0.0410174 | 0.0298344 | -0.144469 | -0.093306 | -0.005199 | 0.1504127 | 0.2743003 |
| B_OTU56 (Lactobacillus_unclassified) | -0.165915 | -0.113216 | -0.048566 | -0.069395 | -0.018375 | 0.2492544 | -0.069395 |
| B_OTU59 (Capnocytophaga_gingivalis) | -0.068671 | 0.0087401 | 0.0204124 | 0.0261905 | -0.069087 | -0.01875 | 0.1919643 |
| B_OTU62 (Campylobacter_showae) | 0.1546526 | 0.1639302 | 0.0306468 | -0.24591 | 0.0545249 | -0.012511 | 0.1134971 |
| B_OTU66 (Amnipila_unclassified) | -0.357482 | -0.128864 | 0.2460378 | -0.175778 | -0.282877 | -0.003587 | 0.060658 |
| B_OTU67 (Actinomyces_unclassified) | -0.553707 | -0.245922 | 0.1531752 | 0.1709249 | -0.157139 | 0.01608 | -0.000893 |
| B_OTU73 (Megasphaera_micronuciformis) | 0.164066 | 0.0278352 | -0.010229 | -0.102755 | 0.2577701 | -0.262332 | 0.2702364 |
| B_OTU108 (Leptotrichia_hongkongensis) | 0.3271092 | 0.2275453 | 0.1531009 | -0.249715 | 0.0832762 | 0.030954 | 0.0982193 |
| B_OTU115 (Prevotella_unclassified) | -0.020222 | 0.0217656 | -0.020655 | -0.117298 | 0.0543238 | -0.258237 | -0.258237 |
| B_OTU138 (Prevotella_unclassified) | -0.209628 | 0.0432149 | 0.0306186 | -0.291071 | -0.268073 | 0.2133929 | -0.130952 |
| B_OTU156 (Neisseria_unclassified) | -0.340535 | -0.268784 | -0.010281 | 0.0131918 | -0.142302 | -0.014991 | -0.035678 |
| B_OTU179 (Porphyromonas_unclassified) | -0.102209 | -0.096773 | 0.1021142 | 0.1619874 | -0.281219 | 0.0023822 | -0.23375 |
| B_OTU188 (Actinomyces_unclassified) | -0.295255 | -0.002063 | 0.0619623 | 0.1090137 | -0.017642 | 0.0542057 | -0.12648 |
| B_OTU200 (Leptotrichia_unclassified) | -0.016277 | 0.0551263 | 0.1542224 | -0.0811 | 0.1160052 | 0.0203874 | 0.3226004 |
| B_OTU246 (Haemophilus_unclassified) | -0.329918 | -0.020394 | 0.0816517 | 0.0285722 | -0.075525 | 0.1041693 | -0.00506 |
| B_OTU316 (Bacteria_unclassified) | 0.1478737 | -0.128087 | 0.088527 | -0.0192 | 0.3205221 | -0.068894 | -0.038561 |
| B_OTU317 (Actinomyces_unclassified) | -0.271915 | -0.067811 | 0.195924 | 0.1085519 | 0.0479844 | -0.139975 | 0.1099803 |
| B_OTU403 (Actinomyces_unclassified) | -0.014618 | 0.2247 | 0.0584801 | 0.1413861 | 0.3126634 | 0.0741037 | 0.0586008 |
| B_OTU544 (Porphyromonas_unclassified) | -0.221435 | -0.088028 | 0.0919028 | 0.106602 | -0.377806 | 0.0461545 | -0.219159 |
| B_OTU899 (Streptococcus_unclassified) | 0.1196464 | -0.01933 | 0.0471223 | -0.23192 | 0.2819843 | -0.23192 | -0.008245 |
| B_OTU1066 (Streptococcus_unclassified) | -0.528854 | -0.292405 | -0.030619 | -0.180952 | -0.168186 | 0.2142857 | -0.021726 |
| B_OTU1226 (Burkholderiales_unclassified) | -0.022352 | -0.175609 | 0.0942168 | -0.030832 | 0.1452615 | -0.231852 | 0.1025703 |
| B_OTU1247 (Streptococcus_unclassified) | -0.190537 | -0.116915 | 0.2177813 | -0.163779 | -0.039679 | -0.038438 | 0.0127013 |
| B_OTU1823 (Streptococcus_unclassified) | 0.4221247 | 0.0978566 | 0.0819071 | -0.047471 | 0.3110145 | -0.236607 | -0.041798 |
| B_OTU1912 (Neisseria_unclassified) | -0.492287 | -0.296642 | 0.1770433 | 0.1516539 | -0.010395 | 0.1716594 | 0.0803443 |
| B_OTU1966 (Veillonella_unclassified) | 0.4804869 | 0.2716225 | 0.1122683 | -0.106548 | 0.5417275 | -0.000298 | -0.019643 |

| Data S19. Correlation analysis between oral bacteria and oral fungi | | | | | | | |
| --- | --- | --- | --- | --- | --- | --- | --- |
| rho | I_OTU157.Aspergillus_penicillioides. | I_OTU190.Aspergillus_versicolor. | I_OTU389.Malassezia_arunalokei. | I_OTU599.Aspergillus_subflavus. | I_OTU1847.Cladosporium_coloradense. | I_OTU1938.Simplicillium_sympodiophorum. | I_OTU3923.Acrodictys_fluminicola. |
| B_OTU5 (Veillonella_unclassified) | 0.0893863 | -0.13467 | -0.093059 | 0.0860119 | -0.062529 | 0.0751353 | 0.180622 |
| B_OTU7 (Porphyromonas_pasteri) | -0.054448 | 0.3114113 | 0.0107338 | -0.153313 | -0.032025 | 0.0707336 | 0.0007169 |
| B_OTU9 (Halomonas_unclassified) | -0.163779 | -0.139975 | -0.083872 | -0.042535 | 0.3302845 | -0.052638 | -0.076827 |
| B_OTU10 (Rothia_unclassified) | -0.322364 | -0.264075 | 0.0319417 | 0.085119 | -0.012248 | 0.0332707 | -0.358019 |
| B_OTU14 (Leptotrichia_unclassified) | 0.0129611 | -0.31161 | -0.139964 | 0.1346441 | 0.0348413 | 0.1520024 | 0.1033048 |
| B_OTU15 (Peptostreptococcus_unclassified) | -0.185882 | 0.0222823 | 0.0016768 | -0.008631 | 0.1295744 | 0.0994463 | -0.04892 |
| B_OTU20 (Lachnoanaerobaculum_unclassified) | 0.2507401 | 0.1982093 | 0.0321932 | -0.105655 | -0.156216 | 0.0847114 | 0.1275822 |
| B_OTU24 (Veillonella_unclassified) | -0.082703 | -0.114866 | -0.20801 | -0.035128 | -0.083609 | 0.0799437 | 0.1480474 |
| B_OTU30 (Enterobacteriaceae_unclassified) | -0.322991 | -0.178263 | -0.165404 | -0.083883 | 0.0907556 | -0.103807 | -0.151511 |
| B_OTU43 (Prevotella_shahii) | 0.3101454 | 0.2225634 | 0.2940811 | -0.210226 | 0.1059887 | -0.042991 | 0.1461132 |
| B_OTU46 (Capnocytophaga_leadbetteri) | 0.0458438 | 0.2754889 | 0.1967007 | -0.223832 | 0.0722062 | -0.048622 | -0.018458 |
| B_OTU48 (Capnocytophaga_sputigena) | -0.081635 | 0.3869443 | -0.082873 | -0.246554 | -0.147051 | -0.155998 | -0.204558 |
| B_OTU54 (Leptotrichia_unclassified) | 0.0489044 | -0.091076 | -0.12166 | 0.073178 | -0.298076 | -0.04248 | -0.021325 |
| B_OTU56 (Lactobacillus_unclassified) | -0.133602 | -0.228367 | -0.136835 | 0.2636998 | -0.100204 | -0.085877 | -0.125342 |
| B_OTU59 (Capnocytophaga_gingivalis) | -0.025326 | 0.3151271 | -0.029427 | -0.083333 | -0.118183 | -0.039409 | 0.1320619 |
| B_OTU62 (Campylobacter_showae) | 0.0355007 | 0.370621 | 0.1074095 | -0.101581 | 0.008603 | -0.08952 | -0.052371 |
| B_OTU66 (Amnipila_unclassified) | -0.196896 | -0.029379 | -0.058977 | 0.0280462 | 0.063808 | 0.1925072 | 0.0775572 |
| B_OTU67 (Actinomyces_unclassified) | -0.583845 | -0.212708 | -0.119951 | 0.0047645 | 0.117171 | 0.1388046 | 0.1233478 |
| B_OTU73 (Megasphaera_micronuciformis) | -0.148846 | -0.114108 | -0.165522 | -0.093062 | 0.0351021 | 0.2428816 | 0.144205 |
| B_OTU108 (Leptotrichia_hongkongensis) | 0.1894399 | 0.0217319 | 0.0982196 | -0.046729 | -0.261519 | -0.176921 | -0.136011 |
| B_OTU115 (Prevotella_unclassified) | 0.0257426 | -0.018334 | 0.0249828 | -0.153436 | -0.026526 | -0.122301 | 0.0960968 |
| B_OTU138 (Prevotella_unclassified) | -0.372901 | -0.199311 | -0.040996 | -0.041071 | -0.200696 | 0.0912182 | -0.149264 |
| B_OTU156 (Neisseria_unclassified) | -0.195099 | -0.13554 | -0.084666 | 0.1130301 | -0.043076 | 0.1869977 | 0.0316796 |
| B_OTU179 (Porphyromonas_unclassified) | -0.018345 | 0.3010792 | 0.0018453 | -0.149779 | 0.0249385 | 0.2101669 | 0.0265334 |
| B_OTU188 (Actinomyces_unclassified) | -0.262405 | -0.184824 | -0.030242 | 0.0240914 | 0.1838299 | 0.0555901 | 0.0455088 |
| B_OTU200 (Leptotrichia_unclassified) | 0.012237 | -0.022569 | 0.1427712 | -0.103136 | -0.022729 | 0.1284991 | 0.0393512 |
| B_OTU246 (Haemophilus_unclassified) | -0.324034 | -0.124083 | -0.002851 | 0.0395843 | -0.214238 | 0.1179851 | -0.389566 |
| B_OTU316 (Bacteria_unclassified) | -0.003231 | -0.056348 | -0.020816 | 0.0621173 | -0.262564 | -0.225024 | -0.010977 |
| B_OTU317 (Actinomyces_unclassified) | -0.246937 | -0.142039 | -0.276008 | 0.1278342 | 0.1010602 | -0.015466 | 0.3166766 |
| B_OTU403 (Actinomyces_unclassified) | 0.07402 | 0.0280596 | 0.0645445 | 0.16247 | 0.0179086 | -0.26322 | 0.0795245 |
| B_OTU544 (Porphyromonas_unclassified) | -0.17015 | 0.1945133 | -0.042024 | -0.13489 | 0.0406326 | 0.2758824 | -0.016852 |
| B_OTU899 (Streptococcus_unclassified) | 0.0360377 | -0.143259 | 0.1264877 | 0.1797037 | -0.184089 | -0.287006 | -0.23983 |
| B_OTU1066 (Streptococcus_unclassified) | -0.580209 | -0.350508 | -0.03999 | 0.1625 | 0.0032232 | 0.2233188 | -0.077947 |
| B_OTU1226 (Burkholderiales_unclassified) | 0.0634734 | -0.123878 | -0.125204 | 0.0355638 | -0.134885 | -0.180389 | 0.0820641 |
| B_OTU1247 (Streptococcus_unclassified) | -0.340798 | -0.311009 | -0.124847 | 0.0701912 | -0.073361 | -0.062045 | 0.0402478 |
| B_OTU1823 (Streptococcus_unclassified) | 0.1459981 | -0.057231 | -0.046382 | 0.1107648 | -0.214046 | -0.135965 | 0.2554295 |
| B_OTU1912 (Neisseria_unclassified) | -0.374095 | -0.166976 | 0.0030903 | 0.0853457 | -0.158065 | 0.030414 | -0.044488 |
| B_OTU1966 (Veillonella_unclassified) | 0.1368298 | 0.0099166 | 0.0740277 | 0.1377976 | -0.160299 | 0.0137503 | -0.001075 |

| Data S19. Correlation analysis between oral bacteria and oral fungi | | | | | | | |
| --- | --- | --- | --- | --- | --- | --- | --- |
| rho | I_OTU4271.Fungi_spe_Incertae_sedis. | I_OTU4508.Blumeria_spe_Incertae_sedis. | I_OTU4684.Acrodictys_fluminicola. | I_OTU5463.Fungi_spe_Incertae_sedis. | I_OTU5606.Cladosporium_cladosporioides. | I_OTU5670.Fungi_spe_Incertae_sedis. | I_OTU5905.Blumeria_spe_Incertae_sedis. |
| B_OTU5 (Veillonella_unclassified) | -0.045124 | 0.19375 | -0.061117 | -0.099682 | -0.174732 | 0.0252976 | 0.085178 |
| B_OTU7 (Porphyromonas_pasteri) | 0.1072512 | -0.093774 | 0.0288471 | 0.15824 | 0.109218 | -0.09169 | 0.1679675 |
| B_OTU9 (Halomonas_unclassified) | -0.061419 | -0.042535 | -0.083872 | -0.083872 | -0.10979 | 0.4782986 | -0.083872 |
| B_OTU10 (Rothia_unclassified) | -0.073918 | 0.0571429 | 0.2884817 | 0.0149229 | 0.2630759 | 0.0485119 | 0.0975019 |
| B_OTU14 (Leptotrichia_unclassified) | 0.0597893 | -0.069109 | -0.017663 | -0.056682 | -0.134277 | 0.186774 | 0.0691431 |
| B_OTU15 (Peptostreptococcus_unclassified) | 0.1837249 | -0.235125 | 0.1331359 | 0.2356706 | 0.1163514 | 0.0258935 | 0.0180253 |
| B_OTU20 (Lachnoanaerobaculum_unclassified) | 0.1100173 | -0.174107 | -0.285799 | 0.2262749 | 0.0030728 | -0.290774 | -0.2277 |
| B_OTU24 (Veillonella_unclassified) | 0.0406222 | 0.1937994 | 0.0285956 | 0.0483861 | -0.190005 | -0.060283 | 0.0327046 |
| B_OTU30 (Enterobacteriaceae_unclassified) | 0.077513 | -0.083883 | 0.0317581 | -0.001102 | 0.0010903 | 0.4607455 | -0.165404 |
| B_OTU43 (Prevotella_shahii) | 0.0481571 | -0.256679 | -0.244005 | 0.0482306 | 0.1677642 | 0.0842692 | -0.245683 |
| B_OTU46 (Capnocytophaga_leadbetteri) | 0.0887534 | -0.128287 | 0.0792337 | 0.0276689 | 0.1398279 | 0.0122036 | -0.096925 |
| B_OTU48 (Capnocytophaga_sputigena) | -0.017844 | 0.0586609 | 0.2329331 | 0.0191245 | 0.0306042 | -0.144121 | -0.024996 |
| B_OTU54 (Leptotrichia_unclassified) | 0.007773 | 0.047121 | 0.2038941 | 0.0074719 | 0.316993 | 0.0393195 | 0.0929149 |
| B_OTU56 (Lactobacillus_unclassified) | 0.1400815 | 0.3059031 | 0.2209306 | 0.0619148 | 0.1441599 | -0.069395 | 0.045 |
| B_OTU59 (Capnocytophaga_gingivalis) | 0.0595211 | -0.057738 | 0.1658288 | 0.1347254 | 0.0537745 | -0.060417 | -0.111838 |
| B_OTU62 (Campylobacter_showae) | 0.0724803 | -0.235633 | -0.214609 | 0.127339 | 0.1580815 | 0.0551102 | -0.263237 |
| B_OTU66 (Amnipila_unclassified) | 0.26724 | -0.175778 | 0.0111156 | 0.2879956 | 0.0449964 | 0.0035873 | 0.0044095 |
| B_OTU67 (Actinomyces_unclassified) | 0.1440451 | 0.231374 | 0.2623814 | 0.0104013 | -0.215632 | 0.0202489 | 0.2249703 |
| B_OTU73 (Megasphaera_micronuciformis) | 0.1451462 | 0.0775513 | -0.046968 | 0.2049695 | -0.163323 | 0.0117818 | 0.0843152 |
| B_OTU108 (Leptotrichia_hongkongensis) | -0.146554 | 0.1339354 | -0.050305 | -0.093189 | 0.1666744 | -0.158341 | -0.035129 |
| B_OTU115 (Prevotella_unclassified) | 0.1092572 | 0.0335783 | -0.282955 | -0.008653 | -0.005795 | -0.258237 | -0.093569 |
| B_OTU138 (Prevotella_unclassified) | -0.004512 | 0.0979167 | 0.1129279 | 0.1198025 | 0.0988193 | 0.1666667 | 0.0581826 |
| B_OTU156 (Neisseria_unclassified) | 0.1114782 | 0.1178271 | 0.1768488 | 0.1686145 | 0.1043674 | -0.06476 | 0.1474585 |
| B_OTU179 (Porphyromonas_unclassified) | 0.2698089 | -0.109878 | -0.096042 | 0.3068308 | 0.2474888 | 0.0336481 | 0.0080524 |
| B_OTU188 (Actinomyces_unclassified) | 0.027069 | 0.2740399 | 0.143022 | -0.014591 | 0.0922163 | -0.073931 | 0.211055 |
| B_OTU200 (Leptotrichia_unclassified) | -0.09362 | -0.208071 | -0.133354 | -0.091465 | 0.0104825 | 0.1460097 | -0.284909 |
| B_OTU246 (Haemophilus_unclassified) | -0.107227 | 0.0413701 | 0.3237851 | 0.0249839 | 0.2782725 | -0.128575 | 0.0705922 |
| B_OTU316 (Bacteria_unclassified) | -0.096569 | -0.181834 | -0.296191 | -0.221246 | -0.046113 | -0.181834 | -0.250152 |
| B_OTU317 (Actinomyces_unclassified) | 0.2090812 | 0.1342616 | -0.149269 | 0.1717 | -0.085298 | 0.0314229 | 0.1190936 |
| B_OTU403 (Actinomyces_unclassified) | -0.145955 | 0.2530067 | 0.1953803 | -0.301499 | -0.055513 | -0.212699 | 0.2482212 |
| B_OTU544 (Porphyromonas_unclassified) | 0.3024869 | -0.088438 | -0.031539 | 0.3490221 | 0.2740404 | 0.070274 | 0.0618191 |
| B_OTU899 (Streptococcus_unclassified) | -0.144736 | 0.040002 | -0.205623 | -0.16726 | -0.035218 | -0.23192 | 0.0902317 |
| B_OTU1066 (Streptococcus_unclassified) | 0.1555713 | 0.1110119 | 0.2561208 | 0.0876931 | 0.0893913 | 0.1014881 | 0.1625591 |
| B_OTU1226 (Burkholderiales_unclassified) | -0.066892 | -0.023048 | -0.250665 | -0.085604 | -0.146846 | -0.231852 | -0.092011 |
| B_OTU1247 (Streptococcus_unclassified) | 0.2265995 | -0.163779 | -0.041239 | 0.1239059 | 0.1152152 | -0.163779 | 0.0608231 |
| B_OTU1823 (Streptococcus_unclassified) | 0.1168307 | 0.1143475 | -0.386402 | -0.010723 | -0.112337 | -0.292586 | 0.0411253 |
| B_OTU1912 (Neisseria_unclassified) | 0.0253929 | -0.007583 | 0.1220688 | 0.0539448 | 0.0597011 | 0.0561442 | -0.106981 |
| B_OTU1966 (Veillonella_unclassified) | -0.163522 | -0.15 | -0.239521 | -0.141013 | -0.001886 | 0.1002976 | -0.049547 |

| Data S19. Correlation analysis between oral bacteria and oral fungi | | | | | | | |
| --- | --- | --- | --- | --- | --- | --- | --- |
| rho | I_OTU5962.Acrodictys_fluminicola. | I_OTU6461.Acrodictys_fluminicola. | I_OTU6608.Acrodictys_fluminicola. | I_OTU6857.Tuber_alcaracense. | I_OTU6998.Calosphaeria_pulchella. | I_OTU7264.Acrodictys_fluminicola. | I_OTU7306.Calosphaeria_pulchella. |
| B_OTU5 (Veillonella_unclassified) | 0.0582362 | -0.12112 | 0.0575195 | 0.1089286 | -0.105105 | -0.10377 | 0.0809915 |
| B_OTU7 (Porphyromonas_pasteri) | -0.016848 | 0.1173455 | 0.0003585 | 0.0756145 | -0.060878 | -0.02025 | 0.0699386 |
| B_OTU9 (Halomonas_unclassified) | -0.076827 | -0.090629 | -0.076827 | -0.042535 | 0.0906426 | 0.0079054 | -0.069395 |
| B_OTU10 (Rothia_unclassified) | 0.183489 | 0.2441432 | 0.1125303 | -0.06875 | 0.0671832 | 0.2781767 | -0.091382 |
| B_OTU14 (Leptotrichia_unclassified) | 0.160517 | 0.0358608 | 0.0435817 | 0.1456658 | 0.0947486 | -0.115821 | 0.0172042 |
| B_OTU15 (Peptostreptococcus_unclassified) | -0.06433 | 0.1458555 | 0.142996 | 0.1229198 | 0.028145 | 0.1866082 | 0.082256 |
| B_OTU20 (Lachnoanaerobaculum_unclassified) | -0.186714 | 0.0743527 | -0.036017 | 0.0261905 | -0.261749 | -0.150345 | -0.106435 |
| B_OTU24 (Veillonella_unclassified) | 0.1421327 | -0.027909 | 0.0917679 | 0.094667 | 0.0082429 | 0.0467527 | 0.1611501 |
| B_OTU30 (Enterobacteriaceae_unclassified) | 0.0393134 | -0.038039 | 0.0181107 | -0.083883 | 0.0871691 | 0.131358 | -0.136854 |
| B_OTU43 (Prevotella_shahii) | -0.218722 | -0.114996 | -0.029402 | 0.0270972 | -0.179363 | -0.279758 | 0.0116594 |
| B_OTU46 (Capnocytophaga_leadbetteri) | 0.0333325 | 0.0459797 | 0.2066254 | -0.02024 | 0.0982008 | 0.0765631 | 0.0908091 |
| B_OTU48 (Capnocytophaga_sputigena) | 0.1769493 | 0.006186 | 0.1717502 | -0.108984 | 0.1454748 | 0.2388593 | 0.103963 |
| B_OTU54 (Leptotrichia_unclassified) | 0.0767501 | 0.1191845 | -0.007046 | -0.056639 | 0.1473296 | 0.2918478 | 0.0112515 |
| B_OTU56 (Lactobacillus_unclassified) | 0.0825382 | 0.3396244 | -0.125342 | -0.069395 | 0.1361842 | 0.2435779 | -0.113216 |
| B_OTU59 (Capnocytophaga_gingivalis) | 0.0904902 | 0.0515237 | 0.1800844 | -0.016667 | 0.0262587 | 0.1652244 | 0.0165091 |
| B_OTU62 (Campylobacter_showae) | -0.15263 | -0.057522 | 0.0661813 | -0.01549 | -0.277263 | -0.27846 | -0.207719 |
| B_OTU66 (Amnipila_unclassified) | 0.0905161 | 0.2425069 | 0.2715482 | 0.200889 | -0.002066 | 0.0129102 | 0.0993882 |
| B_OTU67 (Actinomyces_unclassified) | 0.1653003 | 0.0958062 | 0.089463 | 0.2179739 | 0.2068981 | 0.170377 | 0.1452604 |
| B_OTU73 (Megasphaera_micronuciformis) | 0.1194226 | 0.0523521 | 0.0869181 | 0.2496555 | -0.000105 | 0.0088421 | 0.1769384 |
| B_OTU108 (Leptotrichia_hongkongensis) | -0.094796 | -0.089577 | -0.121854 | -0.220547 | -0.128995 | -0.047545 | -0.237645 |
| B_OTU115 (Prevotella_unclassified) | -0.28965 | -0.01147 | -0.087938 | -0.106457 | -0.176311 | -0.033443 | -0.155454 |
| B_OTU138 (Prevotella_unclassified) | -0.088161 | 0.1704246 | -0.060566 | -0.013393 | -0.044067 | 0.0866225 | -0.009614 |
| B_OTU156 (Neisseria_unclassified) | 0.1630008 | 0.2513741 | 0.3036183 | 0.0767525 | 0.0708094 | 0.3723659 | 0.1884176 |
| B_OTU179 (Porphyromonas_unclassified) | -0.047509 | 0.2542607 | 0.039083 | 0.1759827 | -0.038011 | 0.0021584 | 0.1250471 |
| B_OTU188 (Actinomyces_unclassified) | -0.098361 | 0.2073319 | 0.0364433 | -0.035384 | 0.0456488 | 0.11706 | -0.129165 |
| B_OTU200 (Leptotrichia_unclassified) | 0.0602904 | -0.060847 | 0.0694965 | 0.0857469 | -0.003166 | -0.164632 | -0.093818 |
| B_OTU246 (Haemophilus_unclassified) | 0.1983667 | 0.2165637 | 0.2193323 | 0.0038691 | 0.143798 | 0.3529985 | -0.062056 |
| B_OTU316 (Bacteria_unclassified) | -0.219441 | -0.216922 | -0.162905 | -0.181834 | -0.304997 | -0.255757 | -0.29666 |
| B_OTU317 (Actinomyces_unclassified) | -0.102334 | 0.1285801 | 0.0206388 | 0.0399928 | -0.056809 | 0.0380938 | 0.0593054 |
| B_OTU403 (Actinomyces_unclassified) | 0.0050403 | -0.073001 | -0.010267 | -0.212699 | 0.064898 | 0.0627838 | -0.242304 |
| B_OTU544 (Porphyromonas_unclassified) | -0.030657 | 0.3200861 | 0.0982454 | 0.212013 | -0.010551 | 0.072665 | 0.1587622 |
| B_OTU899 (Streptococcus_unclassified) | -0.184676 | -0.135738 | -0.218044 | -0.23192 | -0.156526 | -0.035613 | -0.206997 |
| B_OTU1066 (Streptococcus_unclassified) | 0.0197107 | 0.2798135 | 0.2204018 | 0.1815476 | 0.0825473 | 0.2779554 | 0.1588754 |
| B_OTU1226 (Burkholderiales_unclassified) | -0.165782 | -0.149194 | -0.0533 | -0.231852 | -0.124747 | 0.036453 | -0.098363 |
| B_OTU1247 (Streptococcus_unclassified) | -0.212106 | 0.1271232 | 0.0704337 | -0.003342 | -0.147215 | -0.00087 | -0.067837 |
| B_OTU1823 (Streptococcus_unclassified) | -0.343329 | -0.09725 | -0.253632 | -0.034633 | -0.325275 | -0.209998 | -0.08378 |
| B_OTU1912 (Neisseria_unclassified) | 0.123847 | 0.0627353 | 0.0961635 | -0.024523 | 0.0426653 | 0.1818291 | -0.004422 |
| B_OTU1966 (Veillonella_unclassified) | -0.287239 | -0.168205 | -0.154102 | -0.002679 | -0.364549 | -0.398707 | -0.233457 |

| Data S19. Correlation analysis between oral bacteria and oral fungi | | | | | | | |  |
| --- | --- | --- | --- | --- | --- | --- | --- | --- |
| rho | I_OTU7440.Dothiora_spartii. | I_OTU7653.Acrodictys_fluminicola. | I_OTU7810.Heitmania_litseae. | I_OTU7828.Acrodictys_fluminicola. | I_OTU7839.Parafuscosporella_mucosa. | I_OTU7942.Fungi_spe_Incertae_sedis. | I_OTU8025.Entorrhiza_citriformis. | I_OTU8123.Heitmania_litseae. |
| B_OTU5 (Veillonella_unclassified) | -0.04671 | 0.016583 | 0.022992 | 0.127998 | -0.09758 | 0.007636 | -0.01264 | 0.157865 |
| B_OTU7 (Porphyromonas_pasteri) | 0.031258 | -0.11202 | 0.035464 | -0.16947 | -0.07046 | 0.003423 | -0.02342 | -0.11274 |
| B_OTU9 (Halomonas_unclassified) | 0.033944 | 0.097045 | -0.06142 | -0.04066 | 0.033394 | -0.00742 | 0.107933 | -0.07683 |
| B_OTU10 (Rothia_unclassified) | 0.149354 | 0.173167 | 0.231423 | 0.116548 | 0.281001 | 0.241249 | 0.144785 | -0.12937 |
| B_OTU14 (Leptotrichia_unclassified) | -0.07383 | -0.13034 | 0.082802 | 0.065163 | -0.08903 | -0.05701 | -0.02261 | -0.09586 |
| B_OTU15 (Peptostreptococcus_unclassified) | 0.172365 | 0.011268 | 0.200271 | -0.08342 | 0.094415 | 0.085415 | -0.00714 | 0.027417 |
| B_OTU20 (Lachnoanaerobaculum_unclassified) | -0.11158 | -0.23078 | -0.04813 | -0.20876 | -0.29408 | -0.15572 | -0.21838 | 0.286522 |
| B_OTU24 (Veillonella_unclassified) | 0.041713 | 0.10213 | 0.055453 | 0.224551 | 0.033194 | 0.197744 | 0.04596 | 0.166509 |
| B_OTU30 (Enterobacteriaceae_unclassified) | 0.180282 | 0.194878 | 0.08228 | 0.179129 | 0.174884 | 0.170674 | 0.109933 | 0.020761 |
| B_OTU43 (Prevotella_shahii) | -0.11994 | -0.23079 | -0.01569 | -0.20327 | -0.25277 | -0.29802 | -0.17186 | 0.190216 |
| B_OTU46 (Capnocytophaga_leadbetteri) | 0.216456 | 0.043695 | 0.133667 | -0.04027 | 0.064925 | 0.046388 | 0.06086 | 0.055554 |
| B_OTU48 (Capnocytophaga_sputigena) | 0.152557 | 0.207608 | 0.196498 | 0.165309 | 0.138043 | 0.180817 | 0.042733 | 0.048764 |
| B_OTU54 (Leptotrichia_unclassified) | 0.210044 | 0.246717 | -0.10916 | 0.196063 | 0.299084 | 0.247053 | 0.150274 | 0.002724 |
| B_OTU56 (Lactobacillus_unclassified) | 0.074454 | 0.162071 | -0.1002 | 0.118551 | 0.283026 | 0.163866 | 0.174184 | 0.08305 |
| B_OTU59 (Capnocytophaga_gingivalis) | 0.247954 | 0.16328 | 0.030513 | 0.023232 | 0.160987 | 0.020363 | 0.155989 | 0.029566 |
| B_OTU62 (Campylobacter_showae) | -0.12743 | -0.27483 | 0.045381 | -0.28735 | -0.33853 | -0.26446 | -0.25123 | 0.175407 |
| B_OTU66 (Amnipila_unclassified) | 0.065888 | -0.01736 | 0.13774 | -0.14286 | 0.071198 | 0.014194 | 0.024724 | 0.007658 |
| B_OTU67 (Actinomyces_unclassified) | 0.101078 | 0.040842 | 0.060628 | 0.087112 | 0.189916 | 0.17635 | 0.113262 | -0.10076 |
| B_OTU73 (Megasphaera_micronuciformis) | 0.017333 | 0.097374 | -0.02778 | 0.137621 | 0.027463 | 0.105952 | 0.119838 | 0.158123 |
| B_OTU108 (Leptotrichia_hongkongensis) | -0.10076 | 0.077711 | -0.17535 | 0 | -0.07402 | -0.03886 | -0.11295 | 0.140491 |
| B_OTU115 (Prevotella_unclassified) | 0.109256 | -0.06336 | 0.074251 | -0.40296 | -0.04343 | 0.098532 | -0.12437 | 0.306513 |
| B_OTU138 (Prevotella_unclassified) | 0.155443 | 0.203569 | -0.02192 | 0.140278 | 0.088688 | 0.316141 | 0.165857 | 0.122027 |
| B_OTU156 (Neisseria_unclassified) | 0.336979 | 0.246406 | 0.283782 | 0.078234 | 0.345924 | 0.2026 | 0.348374 | -0.08448 |
| B_OTU179 (Porphyromonas_unclassified) | 0.09175 | -0.0235 | -0.00365 | -0.05152 | -0.01983 | 0.028806 | 0.04952 | 0.041952 |
| B_OTU188 (Actinomyces_unclassified) | 0.159464 | 0.055878 | -0.18416 | 0.031203 | 0.108233 | 0.131038 | -0.03409 | 0.058563 |
| B_OTU200 (Leptotrichia_unclassified) | -0.12891 | -0.15629 | 0.04221 | 0.17026 | -0.17845 | -0.17562 | -0.09731 | -0.07049 |
| B_OTU246 (Haemophilus_unclassified) | 0.227162 | 0.150953 | 0.228421 | 0.138732 | 0.360807 | 0.473855 | 0.175345 | -0.16638 |
| B_OTU316 (Bacteria_unclassified) | -0.3326 | -0.35349 | -0.06407 | -0.25153 | -0.23611 | -0.22649 | -0.30836 | -0.1122 |
| B_OTU317 (Actinomyces_unclassified) | 0.06988 | -0.07691 | 0.005672 | -0.15848 | -0.00056 | -0.00665 | -0.02362 | 0.103839 |
| B_OTU403 (Actinomyces_unclassified) | 0.023856 | -0.01755 | -0.20237 | -0.09621 | 0.035511 | -0.00203 | -0.12468 | -0.02259 |
| B_OTU544 (Porphyromonas_unclassified) | 0.167543 | 0.053816 | 0.038698 | 0.034589 | 0.06216 | 0.125468 | 0.099761 | 0.045358 |
| B_OTU899 (Streptococcus_unclassified) | -0.20965 | -0.13868 | -0.01676 | -0.24688 | -0.01398 | -0.08337 | -0.24007 | -0.10617 |
| B_OTU1066 (Streptococcus_unclassified) | 0.244667 | 0.105452 | 0.255919 | 0.139669 | 0.270772 | 0.358564 | 0.097757 | -0.08028 |
| B_OTU1226 (Burkholderiales_unclassified) | 0.02509 | -0.01248 | 0.003526 | -0.12153 | 0.056393 | 0.031533 | -0.05469 | 0.037953 |
| B_OTU1247 (Streptococcus_unclassified) | 0.015006 | -0.10995 | 0.056952 | -0.20916 | -0.01371 | 0.126098 | -0.28693 | 0.105852 |
| B_OTU1823 (Streptococcus_unclassified) | -0.2176 | -0.22906 | -0.13602 | -0.30133 | -0.22904 | -0.21429 | -0.31247 | 0.196201 |
| B_OTU1912 (Neisseria_unclassified) | 0.084905 | 0.052842 | 0.141408 | 0.115802 | 0.240602 | 0.172969 | 0.233868 | -0.16931 |
| B_OTU1966 (Veillonella_unclassified) | -0.20372 | -0.22345 | -0.07607 | -0.293 | -0.34749 | -0.22824 | -0.21129 | 0.107513 |

| Data S20. Correlation analysis between fecal bacteria and fecal fungi | | | | | | | |
| --- | --- | --- | --- | --- | --- | --- | --- |
| rho | I_OTU3..Saccharomyces_cerevisiae. | I_OTU4..Zanclospora_jonesii. | I_OTU5..Actinomucor_elegans. | I_OTU11..Hydnobolites_roseus. | I_OTU12..Acrodictys_fluminicola. | I_OTU17..Acrodictys_fluminicola. | I_OTU18..Aspergillus_subflavus. |
| B_OTU1 (Faecalibacterium_unclassified) | 0.0138834 | -0.531061 | -0.256358 | 0.198025 | -0.34847 | 0.0593173 | 0.2650663 |
| B_OTU4 (Blautia_unclassified) | -0.128354 | -0.373339 | -0.193265 | 0.0135432 | -0.332462 | -0.063943 | 0.2761157 |
| B_OTU8 (Agathobacter_unclassified) | 0.1387966 | -0.291853 | -0.261213 | 0.2437568 | -0.333144 | 0.0668984 | 0.2016524 |
| B_OTU20 (Lachnospira_unclassified) | -0.156105 | -0.369956 | -0.373517 | 0.1341323 | -0.552972 | 0.1622091 | 0.3385204 |
| B_OTU21 (Bifidobacterium_unclassified) | -0.191146 | -0.260052 | -0.260219 | 0.2663287 | -0.468828 | 0.0087785 | 0.2587349 |
| B_OTU24 (Dialister_unclassified) | -0.025469 | -0.281008 | -0.002874 | 0.4118175 | -0.168828 | -0.110609 | 0.0484032 |
| B_OTU26 (Lachnoclostridium_unclassified) | 0.1305622 | -0.353248 | 0.0770644 | 0.2134871 | -0.298575 | 0.2742541 | 0.014131 |
| B_OTU34 ([Eubacterium]_eligens_group_unclassified) | 0.0634114 | -0.579325 | -0.132424 | 0.4459174 | -0.482372 | 0.145333 | 0.3479594 |
| B_OTU43 ([Eubacterium]_ruminantium_group_unclassified) | -0.013893 | -0.178983 | -0.043177 | 0.247261 | -0.242158 | -0.074312 | 0.1168703 |
| B_OTU48 (Lachnospiraceae_unclassified) | 0.2403136 | -0.401785 | -0.188611 | 0.1764617 | -0.432443 | 0.0694145 | 0.3087231 |
| B_OTU60 ([Eubacterium]_coprostanoligenes_group_unclassified) | -0.089683 | -0.254566 | -0.0794 | 0.4493042 | -0.229451 | -0.102136 | -0.182789 |
| B_OTU61 (Lachnospiraceae_NK4A136_group) | -0.022342 | -0.466209 | -0.106341 | 0.4281873 | -0.457836 | -0.197657 | 0.1749809 |
| B_OTU63 (Lachnospiraceae_ND3007_group) | 0.1990737 | -0.381359 | -0.063291 | 0.1442754 | 0.015235 | -0.02182 | 0.0947975 |
| B_OTU64 (Intestinibacter_unclassified) | 0.1395777 | -0.345196 | -0.275788 | 0.0538077 | -0.11552 | -0.034149 | 0.4165097 |
| B_OTU66 (Lachnospiraceae_unclassified) | -0.217824 | -0.376985 | -0.158496 | 0.0641978 | -0.206272 | 0.0845942 | 0.2646207 |
| B_OTU73 (Blautia_unclassified) | -0.052717 | -0.432089 | -0.255885 | 0.2403819 | -0.20815 | 0.3352067 | 0.0651552 |
| B_OTU91 (Bacteroides_eggerthii) | -0.005422 | -0.41007 | -0.009829 | 0.1292114 | -0.059519 | -0.074312 | -0.0008 |
| B_OTU136 (Sutterella_unclassified) | 0.0535339 | -0.173727 | 0.0970543 | 0.1643887 | -0.214935 | -0.035795 | -0.110659 |
| B_OTU150 (Lachnospiraceae_unclassified) | 0.0340157 | -0.215349 | 0.0081462 | -0.066791 | -0.064472 | -0.018432 | 0.1293039 |
| B_OTU151 (Lachnospiraceae_CAG-56) | 0.0362854 | -0.212428 | 0.1036413 | 0.3458116 | -0.170435 | -0.052912 | 0.1777532 |
| B_OTU171 (Lachnospiraceae_unclassified) | -0.017133 | -0.248058 | 0.1513476 | 0.2178103 | -0.112012 | -0.12694 | -0.005059 |
| B_OTU184 (Lachnospiraceae_unclassified) | 0.137917 | -0.180827 | -0.002619 | 0.2981989 | -0.434862 | -0.102136 | 0.2357552 |
| B_OTU217 (Lachnospiraceae_unclassified) | 0.0540405 | -0.247264 | 0.009668 | 0.1903604 | -0.190828 | 0.08939 | -0.077932 |
| B_OTU223 ([Eubacterium]_xylanophilum_group_unclassified) | 0.1009953 | -0.232159 | 0.1204336 | 0.1664165 | -0.261721 | 0.1187023 | 0.0938276 |
| B_OTU246 (Clostridium_paraputrificum) | 0.0796528 | 0.1081594 | -0.09413 | -0.309473 | 0.2124706 | 0.0063671 | -0.136031 |
| B_OTU306 (Faecalibacterium_unclassified) | 0.1516509 | -0.220095 | -0.113068 | 0.189965 | -0.318106 | 0.0078571 | 0.10263 |
| B_OTU329 (Lachnospiraceae_bacterium) | 0.2614472 | -0.28512 | -0.27498 | 0.2184787 | -0.307367 | 0.2293619 | 0.2017528 |
| B_OTU331 (Clostridia_unclassified) | 0.0920521 | -0.267031 | -0.099474 | 0.3557717 | -0.243136 | 0.0169791 | 0.1425928 |
| B_OTU431 (Lachnospiraceae_unclassified) | -0.019679 | -0.282422 | -0.207576 | 0.3992318 | -0.402412 | 0.0803837 | 0.2497755 |
| B_OTU499 (Lachnospiraceae_unclassified) | 0.0084629 | -0.519904 | -0.187856 | 0.2590095 | -0.159324 | 0.0513413 | 0.1796294 |
| B_OTU651 (Lachnospiraceae_unclassified) | 0.0736867 | -0.337941 | -0.141698 | 0.277301 | -0.375215 | -0.051524 | 0.304298 |
| B_OTU1605 (Agathobacter_unclassified) | 0.1000396 | -0.281444 | -0.06885 | 0.2754311 | -0.402268 | -0.104448 | 0.3351062 |
| B_OTU1817 (Fusicatenibacter_unclassified) | 0.0549118 | -0.547406 | -0.248514 | 0.2617871 | -0.57392 | -0.116673 | 0.4224236 |
| B_OTU2121 (Fusicatenibacter_unclassified) | 0.064675 | -0.439593 | -0.156711 | 0.232528 | -0.493409 | -0.142792 | 0.3985038 |

| Data S20. Correlation analysis between fecal bacteria and fecal fungi | | | | | | | |
| --- | --- | --- | --- | --- | --- | --- | --- |
| rho | I_OTU19..Candida_solani. | I_OTU25..Zanclospora_jonesii. | I_OTU29..Blumeria_spe_Incertae_sedis. | I_OTU30..Aspergillus_subflavus. | I_OTU31..Cladosporium_kenpeggii. | I_OTU34..Diversispora_spurca. | I_OTU35..Zanclospora_jonesii. |
| B_OTU1 (Faecalibacterium_unclassified) | -0.308678 | -0.010692 | -0.251245 | -0.086651 | 0.0176322 | -0.242695 | -0.266039 |
| B_OTU4 (Blautia_unclassified) | -0.471777 | -0.414439 | -0.257268 | -0.294803 | -0.386841 | -0.387757 | -0.385194 |
| B_OTU8 (Agathobacter_unclassified) | -0.085629 | -0.059869 | 0.044523 | -0.082515 | -0.067004 | -0.14628 | -0.240154 |
| B_OTU20 (Lachnospira_unclassified) | -0.44505 | -0.242686 | 0.0194767 | -0.250809 | -0.225138 | -0.101623 | -0.306659 |
| B_OTU21 (Bifidobacterium_unclassified) | -0.368297 | -0.154659 | -0.201426 | -0.197334 | -0.093165 | 0.0081172 | -0.154338 |
| B_OTU24 (Dialister_unclassified) | -0.22331 | 0.1435161 | -0.034787 | 0.0236231 | 0.0775344 | -0.094869 | -0.227367 |
| B_OTU26 (Lachnoclostridium_unclassified) | -0.259454 | -0.266794 | 0.2014538 | 0.0852354 | 0.1585909 | -0.225841 | -0.322135 |
| B_OTU34 ([Eubacterium]_eligens_group_unclassified) | -0.438857 | 0.0840101 | -0.342593 | -0.203669 | -0.101075 | -0.241337 | -0.606898 |
| B_OTU43 ([Eubacterium]_ruminantium_group_unclassified) | -0.219636 | 0.1445465 | -0.357552 | -0.017964 | -0.283666 | 0.187441 | -0.165768 |
| B_OTU48 (Lachnospiraceae_unclassified) | -0.554147 | -0.212652 | -0.055516 | -0.23561 | -0.315061 | -0.298278 | -0.614677 |
| B_OTU60 ([Eubacterium]_coprostanoligenes_group_unclassified) | -0.406553 | -0.096562 | -0.159584 | -0.335591 | -0.311561 | -0.120413 | -0.281379 |
| B_OTU61 (Lachnospiraceae_NK4A136_group) | -0.209289 | 0.1247433 | -0.07364 | -0.055569 | -0.249718 | 0.0715868 | -0.320391 |
| B_OTU63 (Lachnospiraceae_ND3007_group) | 0.0400714 | -0.090458 | 0.1540602 | 0.1205569 | 0.1189807 | 0.0894531 | -0.210888 |
| B_OTU64 (Intestinibacter_unclassified) | -0.286911 | -0.057936 | -0.322586 | -0.156469 | -0.131163 | -0.054787 | -0.292589 |
| B_OTU66 (Lachnospiraceae_unclassified) | -0.383222 | -0.221707 | -0.002743 | -0.150614 | 0.0412278 | -0.308121 | -0.171164 |
| B_OTU73 (Blautia_unclassified) | -0.305471 | -0.217961 | -0.285549 | -0.171294 | 0.0400315 | -0.184358 | -0.323222 |
| B_OTU91 (Bacteroides_eggerthii) | -0.170563 | -0.303718 | -0.210345 | -0.357536 | -0.262348 | -0.182291 | -0.094113 |
| B_OTU136 (Sutterella_unclassified) | -0.208632 | -0.120901 | -0.172225 | -0.172217 | -0.19013 | -0.138043 | -0.154885 |
| B_OTU150 (Lachnospiraceae_unclassified) | -0.292256 | -0.290435 | 0.1109672 | -0.016599 | 0.0614581 | -0.30073 | -0.220672 |
| B_OTU151 (Lachnospiraceae_CAG-56) | -0.130508 | -0.101263 | 0.138609 | -0.06544 | -0.022834 | -0.120886 | -0.219383 |
| B_OTU171 (Lachnospiraceae_unclassified) | -0.012225 | 0.1196807 | 0.303419 | 0.183759 | 0.3115265 | 0.0502746 | -0.098407 |
| B_OTU184 (Lachnospiraceae_unclassified) | -0.120507 | -0.045142 | -0.117188 | 0.0160947 | -0.205772 | 0.0557643 | -0.120671 |
| B_OTU217 (Lachnospiraceae_unclassified) | -0.110883 | 0.0034855 | -0.045579 | -0.041891 | -0.082365 | -0.112045 | -0.246827 |
| B_OTU223 ([Eubacterium]_xylanophilum_group_unclassified) | -0.139363 | 0.1194702 | -0.048287 | 0.0983861 | 0.0765109 | 0.1169088 | -0.207204 |
| B_OTU246 (Clostridium_paraputrificum) | 0.2641704 | 0.0191164 | 0.0846557 | 0.2395574 | 0.3306543 | 0.0479255 | 0.0870361 |
| B_OTU306 (Faecalibacterium_unclassified) | -0.208702 | 0.0468223 | -0.199791 | 0.0856861 | 0.0764686 | -0.176395 | -0.128781 |
| B_OTU329 (Lachnospiraceae_bacterium) | -0.206845 | 0.0311366 | 0.0477562 | -0.094873 | -0.087166 | -0.145484 | -0.357701 |
| B_OTU331 (Clostridia_unclassified) | -0.034418 | 0.1417798 | -0.036354 | -0.137036 | 0.0805246 | -0.195794 | -0.224374 |
| B_OTU431 (Lachnospiraceae_unclassified) | -0.152486 | 0.0344771 | -0.008748 | -0.138492 | -0.039666 | -0.126254 | -0.241106 |
| B_OTU499 (Lachnospiraceae_unclassified) | -0.159121 | 0.0184393 | 0.0353807 | -0.006693 | 0.2165756 | -0.163038 | -0.247022 |
| B_OTU651 (Lachnospiraceae_unclassified) | -0.124131 | 0.0423644 | 0.019652 | -0.109478 | -0.031233 | -0.285503 | -0.199729 |
| B_OTU1605 (Agathobacter_unclassified) | -0.135618 | 0.0347162 | 0.0254203 | -0.07788 | -0.023546 | -0.290244 | -0.2479 |
| B_OTU1817 (Fusicatenibacter_unclassified) | -0.534895 | -0.130637 | -0.320617 | -0.336343 | -0.254555 | -0.366505 | -0.538254 |
| B_OTU2121 (Fusicatenibacter_unclassified) | -0.319443 | -0.042043 | -0.065241 | -0.163318 | 0.0037439 | -0.202882 | -0.303226 |

| Data S20. Correlation analysis between fecal bacteria and fecal fungi | | | | | | | |
| --- | --- | --- | --- | --- | --- | --- | --- |
| rho | I_OTU46..Zanclospora_jonesii. | I_OTU69..Apiotrichum_domesticum. | I_OTU4321..Hydnobolites_roseus. | I_OTU4657..Fungi_spe_Incertae_sedis. | I_OTU5539..Fungi_spe_Incertae_sedis. | I_OTU5910..Acrodictys_fluminicola. | I_OTU6859..Blumeria_spe_Incertae_sedis. |
| B_OTU1 (Faecalibacterium_unclassified) | 0.16369 | -0.236952 | 0.324931 | -0.375324 | 0.0331839 | -0.397198 | -0.183509 |
| B_OTU4 (Blautia_unclassified) | 0.0353462 | -0.414061 | -0.070806 | -0.25721 | 0.3214208 | -0.280885 | -0.290179 |
| B_OTU8 (Agathobacter_unclassified) | 0.2972765 | -0.405554 | 0.2494588 | -0.31307 | 0.0858094 | -0.366445 | -0.126906 |
| B_OTU20 (Lachnospira_unclassified) | 0.3439436 | -0.418609 | 0.2407827 | -0.435683 | 0.1890969 | -0.497182 | 0.0016445 |
| B_OTU21 (Bifidobacterium_unclassified) | 0.1182971 | -0.229666 | 0.1412165 | -0.479309 | -0.08753 | -0.38247 | -0.091873 |
| B_OTU24 (Dialister_unclassified) | -0.220432 | -0.072122 | 0.1257339 | -0.161263 | -0.021297 | -0.13994 | -0.039369 |
| B_OTU26 (Lachnoclostridium_unclassified) | -0.034931 | -0.067659 | 0.2681004 | -0.367791 | -0.019997 | -0.416305 | 0.1362916 |
| B_OTU34 ([Eubacterium]_eligens_group_unclassified) | 0.1842491 | -0.356518 | 0.143938 | -0.443772 | 0.0970414 | -0.406038 | -0.35652 |
| B_OTU43 ([Eubacterium]_ruminantium_group_unclassified) | -0.148097 | -0.019874 | -0.092154 | -0.286588 | -0.207048 | -0.263705 | -0.084255 |
| B_OTU48 (Lachnospiraceae_unclassified) | 0.1888393 | -0.391283 | 0.2361346 | -0.401809 | 0.0890601 | -0.377161 | -0.044966 |
| B_OTU60 ([Eubacterium]_coprostanoligenes_group_unclassified) | -0.016616 | -0.30018 | 0.3529035 | -0.208394 | -0.10692 | -0.160234 | -0.110437 |
| B_OTU61 (Lachnospiraceae_NK4A136_group) | 0.2011809 | -0.216088 | 0.2726954 | -0.401143 | -0.035818 | -0.4842 | -0.063606 |
| B_OTU63 (Lachnospiraceae_ND3007_group) | 0.1082059 | -0.227434 | -0.001363 | 0.0010815 | 0.1131051 | -0.032942 | -0.019011 |
| B_OTU64 (Intestinibacter_unclassified) | -0.039822 | -0.218817 | -0.006188 | -0.191708 | 0.0319327 | -0.107317 | -0.155399 |
| B_OTU66 (Lachnospiraceae_unclassified) | -0.210834 | -0.21756 | 0.0577647 | -0.134328 | 0.2018381 | -0.202241 | -0.169542 |
| B_OTU73 (Blautia_unclassified) | 0.0065793 | -0.252364 | 0.2777016 | -0.18251 | -0.004011 | -0.278181 | -0.065701 |
| B_OTU91 (Bacteroides_eggerthii) | -0.148097 | -0.218406 | -0.092154 | -0.088294 | 0.2255644 | -0.084749 | -0.252379 |
| B_OTU136 (Sutterella_unclassified) | -0.071335 | -0.105201 | -0.044388 | -0.138043 | -0.09973 | -0.127021 | -0.121565 |
| B_OTU150 (Lachnospiraceae_unclassified) | -0.173973 | -0.113844 | -0.013675 | -0.05722 | -0.020739 | -0.137669 | -0.150724 |
| B_OTU151 (Lachnospiraceae_CAG-56) | 0.1522811 | -0.203357 | 0.1343393 | -0.127045 | -0.11109 | -0.146881 | 0.0017033 |
| B_OTU171 (Lachnospiraceae_unclassified) | -0.096432 | -0.130193 | 0.0737171 | -0.037832 | -0.271729 | -0.103147 | -0.019058 |
| B_OTU184 (Lachnospiraceae_unclassified) | 0.3094225 | -0.30018 | 0.1326882 | -0.335742 | -0.163107 | -0.36244 | -0.173437 |
| B_OTU217 (Lachnospiraceae_unclassified) | 0.1421193 | -0.205373 | 0.3783808 | -0.103797 | -0.324528 | -0.170115 | -0.165592 |
| B_OTU223 ([Eubacterium]_xylanophilum_group_unclassified) | -0.0553 | -0.174282 | 0.1145824 | -0.142508 | -0.176638 | -0.298745 | 0.0631332 |
| B_OTU246 (Clostridium_paraputrificum) | -0.138824 | -0.060818 | -0.03597 | 0.1626235 | -0.002464 | 0.1589367 | 0.1337989 |
| B_OTU306 (Faecalibacterium_unclassified) | 0.2422656 | -0.194643 | 0.364348 | -0.316255 | -0.177895 | -0.299097 | -0.12013 |
| B_OTU329 (Lachnospiraceae_bacterium) | 0.1407833 | -0.43462 | 0.2959641 | -0.328664 | -0.015316 | -0.25125 | 0.023373 |
| B_OTU331 (Clostridia_unclassified) | 0.0942615 | -0.305409 | 0.429443 | -0.224765 | -0.013305 | -0.273792 | -0.125915 |
| B_OTU431 (Lachnospiraceae_unclassified) | 0.2165919 | -0.367031 | 0.3961584 | -0.271834 | 0.1497534 | -0.327798 | -0.147163 |
| B_OTU499 (Lachnospiraceae_unclassified) | 0.1326516 | -0.395643 | 0.3737893 | -0.125444 | 0.0788291 | -0.123246 | -0.178349 |
| B_OTU651 (Lachnospiraceae_unclassified) | 0.1568885 | -0.307411 | 0.2917799 | -0.298338 | 0.1229995 | -0.376917 | -0.166732 |
| B_OTU1605 (Agathobacter_unclassified) | 0.1027431 | -0.232481 | 0.2412354 | -0.335829 | 0.0308845 | -0.433949 | -0.186558 |
| B_OTU1817 (Fusicatenibacter_unclassified) | 0.1173884 | -0.472235 | 0.1805815 | -0.605421 | 0.1188578 | -0.560253 | -0.244282 |
| B_OTU2121 (Fusicatenibacter_unclassified) | 0.0469324 | -0.419669 | 0.2353783 | -0.406707 | 0.0902965 | -0.446614 | -0.16399 |

| Data S20. Correlation analysis between fecal bacteria and fecal fungi | | | | | |
| --- | --- | --- | --- | --- | --- |
| rho | I_OTU7039..Hydnobolites_roseus. | I_OTU7821..Acrodictys_fluminicola. | I_OTU7828..Acrodictys_fluminicola. | I_OTU8026..Saccharomyces_cerevisiae. | I_OTU8088..Zanclospora_jonesii. |
| B_OTU1 (Faecalibacterium_unclassified) | 0.4347648 | -0.020775 | -0.221631 | -0.057925 | -0.077047 |
| B_OTU4 (Blautia_unclassified) | 0.1779045 | 0.4163031 | 0.1039425 | -0.108907 | -0.461782 |
| B_OTU8 (Agathobacter_unclassified) | 0.3637662 | 0.2664052 | 0.0075103 | 0.0690403 | 0.0683941 |
| B_OTU20 (Lachnospira_unclassified) | 0.2933566 | 0.3469604 | 0.0520632 | -0.116542 | -0.295564 |
| B_OTU21 (Bifidobacterium_unclassified) | 0.3713923 | 0.0619381 | -0.062088 | -0.240186 | 0.0053417 |
| B_OTU24 (Dialister_unclassified) | 0.3266198 | -0.024868 | -0.036259 | -0.093559 | 0.0369998 |
| B_OTU26 (Lachnoclostridium_unclassified) | 0.3653929 | -0.04285 | -0.105849 | 0.1193424 | -0.056136 |
| B_OTU34 ([Eubacterium]_eligens_group_unclassified) | 0.5064084 | 0.3292422 | 0.0772008 | 0.0794187 | -0.172884 |
| B_OTU43 ([Eubacterium]_ruminantium_group_unclassified) | 0.2105304 | 0.0708958 | 0.0260523 | -0.202237 | -0.096498 |
| B_OTU48 (Lachnospiraceae_unclassified) | 0.4102329 | 0.245459 | -0.320295 | 0.2959689 | -0.189031 |
| B_OTU60 ([Eubacterium]_coprostanoligenes_group_unclassified) | 0.4282861 | -0.026799 | -0.2638 | -0.172784 | 0.0374871 |
| B_OTU61 (Lachnospiraceae_NK4A136_group) | 0.4583714 | 0.150331 | -0.064747 | -0.204873 | 0.0030449 |
| B_OTU63 (Lachnospiraceae_ND3007_group) | 0.2376486 | -0.076919 | 0.085157 | -0.007747 | -0.149483 |
| B_OTU64 (Intestinibacter_unclassified) | 0.1018238 | 0.1572443 | -0.034406 | 0.1192061 | -0.43405 |
| B_OTU66 (Lachnospiraceae_unclassified) | 0.2670304 | 0.0996577 | 0.0093352 | -0.208067 | -0.104785 |
| B_OTU73 (Blautia_unclassified) | 0.2720778 | 0.1465457 | -0.266068 | -0.005104 | -0.066031 |
| B_OTU91 (Bacteroides_eggerthii) | 0.119446 | 0.1976597 | 0.1018409 | -0.098385 | -0.277066 |
| B_OTU136 (Sutterella_unclassified) | 0.186702 | 0.0139661 | -0.053455 | -0.202395 | -0.184095 |
| B_OTU150 (Lachnospiraceae_unclassified) | 0.1307434 | 0.0182554 | -0.120158 | 0.0633326 | -0.185899 |
| B_OTU151 (Lachnospiraceae_CAG-56) | 0.2382533 | -0.018101 | -0.112159 | 0.0435773 | -0.032768 |
| B_OTU171 (Lachnospiraceae_unclassified) | 0.2925471 | 0.0090602 | -0.093051 | -0.060222 | 0.1454352 |
| B_OTU184 (Lachnospiraceae_unclassified) | 0.3149141 | 0.3485209 | 0.0162757 | 0.0386349 | -0.122358 |
| B_OTU217 (Lachnospiraceae_unclassified) | 0.3303589 | -0.118335 | -0.261892 | -0.047043 | 0.1118946 |
| B_OTU223 ([Eubacterium]_xylanophilum_group_unclassified) | 0.2016005 | 0.0299992 | 0.0993348 | 0.0071186 | -0.082864 |
| B_OTU246 (Clostridium_paraputrificum) | -0.147319 | -0.03654 | 0.1875951 | 0.0668037 | -0.115018 |
| B_OTU306 (Faecalibacterium_unclassified) | 0.3695097 | -0.156116 | -0.252629 | 0.0208223 | 0.0400472 |
| B_OTU329 (Lachnospiraceae_bacterium) | 0.424398 | 0.1104284 | -0.143791 | 0.1541731 | -0.045285 |
| B_OTU331 (Clostridia_unclassified) | 0.4882597 | -0.009213 | -0.1663 | 0.0436024 | 0.1269444 |
| B_OTU431 (Lachnospiraceae_unclassified) | 0.5370533 | 0.2047972 | 0.0102715 | -0.09397 | 0.1601367 |
| B_OTU499 (Lachnospiraceae_unclassified) | 0.3562816 | 0.1155612 | -0.024424 | 0.0274693 | -0.104212 |
| B_OTU651 (Lachnospiraceae_unclassified) | 0.3675017 | 0.1877817 | -0.068812 | -0.004414 | 0.0885085 |
| B_OTU1605 (Agathobacter_unclassified) | 0.3781972 | 0.1841642 | -0.1781 | 0.1120253 | 0.112995 |
| B_OTU1817 (Fusicatenibacter_unclassified) | 0.4800899 | 0.3126052 | -0.197497 | 0.1103594 | -0.349542 |
| B_OTU2121 (Fusicatenibacter_unclassified) | 0.4543745 | 0.2176457 | -0.18897 | 0.0630161 | -0.194046 |
